# Supplementary material for: Safety and immunogenicity of a hybrid-type vaccine booster in BBIBP-CorV recipients in a randomized phase 2 trial
Source: Nat Commun. 2022 Jun 27;13:3654. doi: 10.1038/s41467-022-31379-0 (PMC9237056; doi:10.1038/s41467-022-31379-0)
Supplement: Supplementary file 1 — Supplementary information [file 41467_2022_31379_MOESM1_ESM.pdf]

Safety and immunogenicity of a hybrid-type vaccine booster in BBIBP-CorV recipients in a randomized phase 2 trial

Supplementary Tables

Supplementary Table 1. Baseline characteristic for the nationality of the participants

	4-6 months			7-9 months			＞9 months		
	**NVSI-06-08 (N=285)**	**BBIBP-CorV (N=289)**	***p* value**	**NVSI-06-08 (N=270)**	**BBIBP-CorV (N=270)**	***p* value**	**NVSI-06-08 (N=286)**	**BBIBP-CorV (N=278)**	***p* value**
**Countries**, n(%)									
Afghanistan	6 (2.11)	8 (2.77)	0.7975	14 (5.19)	15 (5.56)	0.7707	1 (0.35)	3 (1.08)	0.8782
Bangladesh	118 (41.40)	108 (37.37)		74 (27.41)	70 (25.93)		104 (36.36)	101 (36.33)	
Cameroon	0 (0.00)	0 (0.00)		0 (0.00)	1 (0.37)		0 (0.00)	1 (0.36)	
Columbia	0 (0.00)	0 (0.00)		0 (0.00)	0 (0.00)		0 (0.00)	1 (0.36)	
Canada	0 (0.00)	0 (0.00)		0 (0.00)	0 (0.00)		0 (0.00)	0 (0.00)	
Egypt	2 (0.70)	2 (0.69)		7 (2.59)	4 (1.48)		7 (2.45)	7 (2.52)	
United Arab Emirates	0 (0.00)	0 (0.00)		0 (0.00)	0 (0.00)		5 (1.75)	4 (1.44)	
Eritrea	0 (0.00)	0 (0.00)		0 (0.00)	1 (0.37)		0 (0.00)	0 (0.00)	
Ethiopia	1 (0.35)	3 (1.04)		0 (0.00)	1 (0.37)		5 (1.75)	3 (1.08)	
Philippines	3 (1.05)	5 (1.73)		6 (2.22)	8 (2.96)		4 (1.40)	3 (1.08)	
Ghana	4 (1.40)	3 (1.04)		2 (0.74)	1 (0.37)		5 (1.75)	4 (1.44)	
India	31 (10.88)	34 (11.76)		36 (13.33)	49 (18.15)		35 (12.24)	24 (8.63)	
Jordan	0 (0.00)	0 (0.00)		2 (0.74)	0 (0.00)		1 (0.35)	1 (0.36)	
Mauritania	0 (0.00)	1 (0.35)		1 (0.37)	0 (0.00)		0 (0.00)	0 (0.00)	
Morocco	1 (0.35)	1 (0.35)		2 (0.74)	1 (0.37)		2 (0.70)	0 (0.00)	
Namibia	0 (0.00)	0 (0.00)		0 (0.00)	0 (0.00)		0 (0.00)	1 (0.36)	

	4-6 months			7-9 months			＞9 months		
	**NVSI-06-08 (N=285)**	**BBIBP-CorV (N=289)**	***p* value**	**NVSI-06-08 (N=270)**	**BBIBP-CorV (N=270)**	***p* value**	**NVSI-06-08 (N=286)**	**BBIBP-CorV (N=278)**	***p* value**
Nepal	23 (8.07)	31 (10.73)		15 (5.56)	15 (5.56)		11 (3.85)	10 (3.60)	
Nigeria	3 (1.05)	1 (0.35)		2 (0.74)	4 (1.48)		3 (1.05)	2 (0.72)	
Oman	0 (0.00)	0 (0.00)		0 (0.00)	0 (0.00)		1 (0.35)	0 (0.00)	
Pakistan	73 (25.61)	68 (23.53)		94 (34.81)	89 (32.96)		78 (27.27)	91 (32.73)	
Palestine	0 (0.00)	0 (0.00)		0 (0.00)	0 (0.00)		1 (0.35)	0 (0.00)	
Sierra Leone	0 (0.00)	0 (0.00)		0 (0.00)	0 (0.00)		1 (0.35)	0 (0.00)	
Sri Lanka	2 (0.70)	0 (0.00)		1 (0.37)	2 (0.74)		3 (1.05)	4 (1.44)	
Sudan	0 (0.00)	0 (0.00)		2 (0.74)	0 (0.00)		3 (1.05)	3 (1.08)	
Uganda	14 (4.91)	18 (6.23)		9 (3.33)	8 (2.96)		12 (4.20)	12 (4.32)	
Yemen	0 (0.00)	0 (0.00)		0 (0.00)	0 (0.00)		2 (0.70)	0 (0.00)	
Zimbabwe	3 (1.05)	6 (2.08)		1 (0.37)	0 (0.00)		0 (0.00)	0 (0.00)	
Syria	1 (0.35)	0 (0.00)		1 (0.37)	1 (0.37)		2 (0.70)	3 (1.08)	
Kazakhstan	0 (0.00)	0 (0.00)		1 (0.37)	0 (0.00)		0 (0.00)	0 (0.00)	

Results were obtained from the participants who had no protocol deviations.

Comparisons between NVSI-06-08 and BBIBP-CorV booster groups were carried out using Chi-square test. All the tests were two-sided and a *p*-value < 0.05 was considered statistically significant.

N = the number of participants.

Supplementary Table 2. Adverse reactions within 7 days of booster vaccination

	NVSI-06-08 (N=896)	BBIBP-CorV (N=904)	Total (N=1800)	*p* value
**Solicited adverse reactions within 0-7 days**	146 (16.29)	115 (12.72)	261 (14.50)	
Grade 1	121 (13.50)	94 (10.40)	215 (11.94)	
Grade 2	44 (4.91)	35 (3.87)	79 (4.39)	
Grade 3	4 (0.45)	2 (0.22)	6 (0.33)	
**Injection site adverse reactions**	46 (5.13)	42 (4.65)	88 (4.89)	
Grade 1	40 (4.46)	37 (4.09)	77 (4.28)	
Grade 2	6 (0.67)	8 (0.88)	14 (0.78)	
Pain	42 (4.69)	36 (3.98)	78 (4.33)	0.4888
Grade 1	36 (4.02)	32 (3.54)	68 (3.78)	0.6225
Grade 2	6 (0.67)	4 (0.44)	10 (0.56)	0.5460
Swelling	0	1 (0.11)	1 (0.06)	1.0000
Grade 1	0	1 (0.11)	1 (0.06)	1.0000
Erythra	0	2 (0.22)	2 (0.11)	0.4997
Grade 1	0	2 (0.22)	2 (0.11)	0.4997
Redness	2 (0.22)	0	2 (0.11)	0.2476
Grade 1	2 (0.22)	0	2 (0.11)	0.2476
Pruritus	3 (0.33)	6 (0.66)	9 (0.50)	0.5068
Grade 1	3 (0.33)	2 (0.22)	5 (0.28)	0.6858
Grade 2	0	4 (0.44)	4 (0.22)	0.1246
**Systemic adverse reactions**	111 (12.39)	84 (9.29)	195 (10.83)	
Grade 1	89 (9.93)	65 (7.19)	154 (8.56)	
Grade 2	39 (4.35)	30 (3.32)	69 (3.83)	
Grade 3	4 (0.45)	2 (0.22)	6 (0.33)	
Fever	24 (2.68)	22 (2.43)	46 (2.56)	0.7671
Grade 1	18 (2.01)	19 (2.10)	37 (2.06)	1.0000
Grade 2	3 (0.33)	1 (0.11)	4 (0.22)	0.3724
Grade 3	3 (0.33)	2 (0.22)	5 (0.28)	0.6858
Diarrhea	2 (0.22)	2 (0.22)	4 (0.22)	1.0000
Grade 1	2 (0.22)	2 (0.22)	4 (0.22)	1.0000
Vomiting	2 (0.22)	1 (0.11)	3 (0.17)	0.6231
Grade 1	1 (0.11)	1 (0.11)	2 (0.11)	1.0000
Grade 2	1 (0.11)	0	1 (0.06)	0.4978
Nausea	5 (0.56)	2 (0.22)	7 (0.39)	0.2861
Grade 1	4 (0.45)	2 (0.22)	6 (0.33)	0.4504
Grade 2	1 (0.11)	0	1 (0.06)	0.4978
Muscle pain (non-inoculation site)	30 (3.35)	25 (2.77)	55 (3.06)	0.4961
Grade 1	19 (2.12)	16 (1.77)	35 (1.94)	0.6128
Grade 2	10 (1.12)	8 (0.88)	18 (1.00)	0.6446
Grade 3	1 (0.11)	1 (0.11)	2 (0.11)	1.0000
Joint pain	2 (0.22)	2 (0.22)	4 (0.22)	1.0000

	NVSI-06-08 (N=896)	BBIBP-CorV (N=904)	Total (N=1800)	*p* value
Grade 1	1 (0.11)	2 (0.22)	3 (0.17)	1.0000
Grade 2	1 (0.11)	0	1 (0.06)	0.4978
Headache	52 (5.80)	38 (4.20)	90 (5.00)	0.1304
Grade 1	30 (3.35)	18 (1.99)	48 (2.67)	0.0801
Grade 2	23 (2.57)	20 (2.21)	43 (2.39)	0.6463
Cough	20 (2.23)	13 (1.44)	33 (1.83)	0.2233
Grade 1	14 (1.56)	9 (1.00)	23 (1.28)	0.3024
Grade 2	6 (0.67)	5 (0.55)	11 (0.61)	0.7728
Breathing trouble	0	1 (0.11)	1 (0.06)	1.0000
Grade 1	0	1 (0.11)	1 (0.06)	1.0000
Fatigue	27 (3.01)	22 (2.43)	49 (2.72)	0.4721
Grade 1	19 (2.12)	17 (1.88)	36 (2.00)	0.7393
Grade 2	9 (1.00)	5 (0.55)	14 (0.78)	0.2979
Dizziness	1 (0.11)	2 (0.22)	3 (0.17)	1.0000
Grade 1	1 (0.11)	1 (0.11)	2 (0.11)	1.0000
Grade 2	0	1 (0.11)	1 (0.06)	1.0000
**Unsolicited adverse reactions within 0-30 days**	48 (5.36)	48 (5.31)	96 (5.33)	
Grade 1	40 (4.46)	32 (3.54)	72 (4.00)	
Grade 2	13 (1.45)	22 (2.43)	35 (1.94)	
Grade 3	2 (0.22)	1 (0.11)	3 (0.17)	

p-values were calculated using two-sided Fisher's exact test.

N = the number of participants.

Data are presented as n (%).

Supplementary Table 3. Live-virus neutralizing antibody response results (PPS)

	15 days after boosting			30 days after boosting		
	**NVSI-06-08**	**BBIBP-CorV**	***p* value**	**NVSI-06-08**	**BBIBP-CorV**	***p* value**
**4-6 months**						
N(missing)	285 (0)	289 (0)		285 (0)	289 (0)	
Pre-booster antibody GMT[1] (95%CI)	78.35 (67.10-91.48)	67.28 (57.41-78.84)	0.1771	78.35 (67.10-91.48)	67.28 (57.41-78.84)	0.1771
Post-booster antibody GMT (95%CI)	3141.92 (2731.70-3613.74)	196.89 (176.01-220.24)	<0.0001	3730.18 (3216.15-4326.36)	294.96 (266.23-326.80)	<0.0001
Ratio of GMT between two groups(95%CI)[2]	15.96 (13.34-19.08)			12.65 (10.56-15.14)		
Rate of 4-fold rise[3] (%, 95%CI)	93.68 (90.20-96.21)	22.84 (18.13-28.12)	<0.0001	96.84 (94.09-98.55)	38.75 (33.11-44.64)	<0.0001
Rate difference between two groups (%, 95%CI[4])	70.85 (64.82-76.03)			58.09 (51.92-63.84)		
Post-booster antibody GMT fold rise (95%CI）	40.10 (34.61-46.47)	2.93 (2.54-3.37)	<0.0001	47.61 (41.17-55.06)	4.38 (3.81-5.05)	<0.0001
**7-9 months**						
N(missing)	270 (0)	270 (0)		270 (0)	270 (0)	
Pre-booster antibody GMT[1] (95%CI)	51.98 (44.89-60.19)	48.80 (42.03-56.66)	0.5526	51.98 (44.89-60.19)	48.80 (42.03-56.66)	0.5526
Post-booster antibody GMT (95%CI)	4908.34 (4259.82-5655.59)	504.76 (443.94-573.92)	<0.0001	7719.35 (6828.68-8726.20)	1093.26 (958.59-1246.84)	
Ratio of GMT between two groups(95%CI)[2]	9.72 (8.04-11.77)			7.06 (5.90-8.45)		
Rate of 4-fold rise[3] (%, 95%CI)	98.15 (95.73-99.40)	75.19 (69.59-80.22)	<0.0001	99.26 (97.35-99.91)	92.96 (89.23-95.71)	0.0002
Rate difference between two groups (%, 95%CI[4])	22.96 (17.80-28.63)			6.30 (3.08-9.51)		
Post-booster antibody GMT fold rise (95%CI）	94.42 (79.36-112.34)	10.34 (8.78-12.19)	<0.0001	148.50 (126.60-174.19)	22.40 (19.19-26.15)	<0.0001
**>9 months**						
N(missing)	286 (0)	278 (0)		286 (0)	278 (0)	
Pre-booster antibody GMT[1] (95%CI)	16.96 (14.56-19.74)	17.47 (14.94-20.44)	0.7870	16.96 (14.56-19.74)	17.47 (14.94-20.44)	0.7870

	15 days after boosting			30 days after boosting		
	**NVSI-06-08**	**BBIBP-CorV**	***p* value**	**NVSI-06-08**	**BBIBP-CorV**	***p* value**
Post-booster antibody GMT (95%CI)	4184.82 (3651.45-4796.10)	374.63 (339.20-413.76)	<0.0001	7479.16 (6540.06-8553.10)	808.29 (714.16-914.82)	<0.0001
Ratio of GMT between two groups(95%CI)[2]	11.17 (9.44-13.22)			9.25 (7.71-11.10)		
Rate of 4-fold rise[3] (%, 95%CI)	99.65 (98.07-99.99)	94.24 (90.82-96.67)	<0.0001	100.00 (98.72-100.00)	98.20 (95.85-99.41)	0.0286
Rate difference between two groups (%, 95%CI[4])	5.41 (2.96-8.84)			1.80 (0.46-4.14)		
Post-booster antibody GMT fold rise (95%CI）	246.81 (207.02-294.26)	21.44(18.56-24.77)	<0.0001	441.11 (373.91-520.38)	46.26 (39.76-53.83)	<0.0001

Notes: [1] GMT represent geometric mean titer.

[2] The ratio of GMT between two groups was calculated by "NVSI-06-08/ BBIBP-CorV", and the non-inferiority threshold of ratio between groups on day 15 post-booster was set to 0·67.

[3] Rate of 4-fold rise was defined as percentage of participants with a ≥4-fold rise from baseline in neutralizing antibody titer.

[4] Rate difference=(NVSI-06-08)-(BBIBP-CorV). Rate difference and 95%CI were estimated by Miettnen-Nurminen method without considering stratification factors.

Differences in neutralizing antibody titers between heterologous and homologous booster groups were tested with two-sided grouped t-test after log transformation. The 4-fold rise rates and the post-booster antibody GMT fold rise between heterologous and homologous booster groups were compared by two-sided Fisher's exact test and two-sided grouped t-test, respectively. A two-sided *p*-value < 0.05 was considered significant.

Supplementary Table 4. RBD-specific IgG response results (PPS)

	15 days after boosting			30 days after boosting		
	**NVSI-06-08**	**BBIBP-CorV**	***p* value**	**NVSI-06-08**	**BBIBP-CorV**	***p* value**
**4-6 months**						
N(missing)	285 (0)	289 (0)		285 (0)	289 (0)	
Pre-booster antibody GMC[1] (95%CI)	113.71 (96.45-134.05)	95.53 (81.53-111.94)	0.1341	113.71 (96.45-134.05)	95.53 (81.53-111.94)	0.1341
Post-booster antibody GMC (95%CI)	7119.99 (6235.45-8130.01)	254.00 (228.22-282.69)	<0.0001	5030.67 (4416.40-5730.38)	213.91 (191.73-238.66)	<0.0001
Ratio of GMC between two groups (95%CI)[2]	28.03(23.65-33.23)			23.52 (19.85-27.87)		
Rate of 4-fold rise[3] (%, 95%CI)	96.14 (93.20-98.06)	20.76 (16.23-25.90)	<0.0001	95.44 (92.33-97.55)	17.30 (13.12-22.16)	<0.0001
Rate difference between two groups (%, 95%CI[4])	75.38 (69.76-80.13)			78.14 (72.67-82.66)		
Post-booster antibody GMC fold rise (95%CI）	62.62 (53.00-73.98)	2.66(2.30-3.08)	<0.0001	44.24 (37.82-51.75)	2.24 (1.94-2.58)	<0.0001
**7-9 months**						
N(missing)	270 (0)	270 (0)		270 (0)	270 (0)	
Pre-booster antibody GMC[1] (95%CI)	133.93 (111.27-161.20)	118.77 (97.65-144.47)	0.3810	133.93 (111.27-161.20)	118.77 (97.65-144.47)	0.3810
Post-booster antibody GMC (95%CI)	6583.20 (5734.58-7557.39)	303.96 (270.91-341.04)	<0.0001	4947.49 (4395.20-5569.19)	290.61 (258.00-327.35)	<0.0001
Ratio of GMC between two groups (95%CI)[2]	21.66 (18.10-25.91)			17.02 (14.40-20.13)		
Rate of 4-fold rise[3] (%, 95%CI)	92.59 (88.79-95.42)	22.96 (18.08-28.45)	<0.0001	92.22 (88.36-95.12)	22.96 (18.08-28.45)	<0.0001
Rate difference between two groups (%, 95%CI[4])	69.6 3 (63.27-75.10)			69.26 (62.87-74.76)		
Post-booster antibody GMC fold rise (95%CI）	49.15 (40.43-59.76)	2.56 (2.13-3.07)	<.0001	36.94 (30.65-44.52)	2.45 (2.04-2.93)	<.0001
**>9 months**						
N(missing)	286(0)	278(0)		286(0)	278(0)	
Pre-booster antibody GMC[1] (95%CI)	120.45 (96.96-149.63)	116.05 (91.23-147.62)	0.8210	120.45 (96.96-149.63)	116.05 (91.23-147.62)	0.8210

	15 days after boosting			30 days after boosting		
	**NVSI-06-08**	**BBIBP-CorV**	***p* value**	**NVSI-06-08**	**BBIBP-CorV**	***p* value**
Post-booster antibody GMC (95%CI)	7440.80 (6509.03-8505.96)	414.87 (362.17-475.25)	<0.0001	4960.67 (4379.76-5618.63)	267.88 (233.41-307.44)	<0.0001
Ratio of GMC between two groups (95%CI)[2]	17.94 (14.83-21.69)			18.52 (15.39-22.29)		
Rate of 4-fold rise[3] (%, 95%CI)	90.56 (86.56-93.69)	31.65 (26.23-37.48)	<0.0001	88.11 (83.79-91.62)	21.94 (17.22-27.27)	<0.0001
Rate difference between two groups (%, 95%CI[4])	58.90 (52.14-65.00)			66.17 (59.59-71.88)		
Post-booster antibody GMC fold rise (95%CI)	61.77 (49.74-76.72)	3.58 (2.93-4.36)	<0.0001	41.18 (33.25-51.01)	2.31 (1.89-2.82)	<0.0001

Notes: [1] GMC represents geometric mean concentration, and the unit of GMC is BAU/ml.

[2] The ratio of GMC between two groups was calculated by "NVSI-06-08/ BBIBP-CorV".

[3] Rate of 4-fold rise was defined as percentage of participants with a ≥4-fold rise from baseline in IgG concentration.

[4] Rate difference=(NVSI-06-08)-(BBIBP-CorV). Rate difference and 95%CI were estimated by Miettnen-Nurminen method without considering stratification factors.

Differences in RBD-binding IgG antibody concentrations between heterologous and homologous booster groups were tested with two-sided grouped t-test after log transformation. The 4-fold rise rates and the post-booster antibody GMC fold rise between heterologous and homologous booster groups were compared by two-sided Fisher's exact test and two-sided grouped t-test, respectively. A two-sided *p*-value < 0.05 was considered significant.

Supplementary Table 5. Live-virus neutralizing antibody responses against main SARS-CoV-2 VOC variants

	NVSI-06-08	BBIBP-CorV	GMT ratio and 95%CI	*p* value
**Prototype strain**				
n	99	101		
GMT (95%CI)	3797.54 (2991.48-4820.80)	460.39 (369.08-574.29)	8.25 (5.97-11.39)	<0.0001
**Alpha strain**				
n	99	101		
GMT (95%CI)	3263.18 (2522.75-4220.92)	295.59 (230.60-378.89)	11.04 (7.74-15.75)	<0.0001
**Beta strain**				
n	99	101		
GMT (95%CI)	4002.56 (3003.56-5333.67)	267.12 (209.87-340.00)	14.98 (10.33-21.74)	<0.0001
**Delta strain**				
n	99	101		
GMT (95%CI)	2609.91 (2036.72-3344.41)	275.34 (217.85-348.01)	9.48 (6.75-13.30)	<0.0001
**Omicron strain**				
n	99	101		
GMT (95%CI)	367.67 (295.50-457.47)	45.03 (36.37-55.74)	8.17 (6.03-11.06)	<0.0001

If the titer is less than 1:10, it shall be calculated as 5. “n” is the number of serum samples.

Differences between NVSI-06-08 and BBIBP-CorV booster groups were tested with two-sided grouped t-test after log transformation. A two-sided p-value < 0.05 was considered significant.

Supplementary Notes

Supplementary Note 1. Safety reports for phase 1 trial.

Open-label Phase I Clinical Trial Safety Summary Report for Recombinant COVID-19 Vaccine (CHO cells, NVSI-06-08) in Subjects Aged 18 and Above（30 Days after Full Vaccination)

Name of test vaccine: Recombinant COVID-19 vaccine (CHO cells, NVSI-06-08)

Study Number: CNBG-REC-2021003

Study start time: October 23, 2021

Study Completion Time: Ongoing

Main researcher: Nawal Al Kaabi

Study Responsible: Sheikh Khalifa Medical City, SEHA, Abu Dhabi, United Arab Emirates

Reporting date: February 09, 2022

Source data stored at Sheikh Khalifa Medical City

Version number: V1.0

1. Safety Assessment

1.1. Exposure degree

48 Subjects were immunized with the recombinant COVID-19 vaccine according to a 3-dose immunization schedule. Intramuscular injection into the lateral deltoid muscle of the upper arm. one dose per person per time, and 0.5 ml per dose.

1.2. Adverse events

1.2.1. Total adverse events

48 subjects in this trial, 22 participants (45.83%) with 101 cases were observed, of which 20 participants (41.67%) with 60 cases were related to the study vaccine. No adverse events of grade 3 and above were observed.

The incidence of adverse events of severity grade 1 and grade 2 was 41.67% and 27.08%, and the incidence of adverse events of grade 1 and grade 2 related to the study vaccine was 35.42% and 20.83%. The adverse events related to the study vaccine mainly was general disorders and administration site conditions. There were 13 participants (27.08%) with 22 cases, of which the main adverse reactions were vaccination site pain (18.75%) and fatigue (10.42%).

See Table 1-1, Table 1-2, Table 1-3 for details.

Table 1-1 Total incidence of adverse events in the whole population during the period of full immunization (SS)

	NVSI-06-08 (N=48)		
	**Cases**	**Number of participants**	**Percentage (%)**
Adverse events occurring during the Immunization period	101	22	45.83
Adverse events related to study vaccine	60	20	41.67
Adverse events unrelated to study vaccine	41	8	16.67
Adverse events of grade 3 and above	0	0	0
Study vaccine related adverse events of grade 3 and above	0	0	0

(1) Adverse events were coded using MedDRA version 24.1.
(2) Study vaccine related means that the relationship between adverse events and study vaccine is "definitely related", "most likely related" or "likely related".
(3) Not related to the study vaccine means that the relationship between the adverse event and the study vaccine is "definitely not related" or "may not be related".
(4) Adverse events occurring during full immunization are those that occur within 30 days after full vaccination.
(5) The data for this analysis is as of Jan. 25, 2022.

Table 1-2 Incidence and severity of various adverse events in the whole population during the period of full immunization (listed per SOC and PT) (SS)

Adverse Events	NVSI-06-08 (N=48)		
Severity	Cases	Number of participants	Percentage (%)
**TEAE**	101	22	45.83
Grade 1	44	20	41.67
Grade 2	57	13	27.08
**Cardiac disorders**	1	1	2.08
Grade 1	1	1	2.08
Grade 2	0	0	0
**Palpitations**	1	1	2.08
Grade 1	1	1	2.08
Grade 2	0	0	0
**Gastrointestinal disorders**	1	1	2.08
Grade 1	0	0	0
Grade 2	1	1	2.08
**Nausea**	1	1	2.08
Grade 1	0	0	0
Grade 2	1	1	2.08
**General disorders and administration site conditions**	31	17	35.42
Grade 1	21	15	31.25
Grade 2	10	9	18.75
**Chills**	1	1	2.08
Grade 1	1	1	2.08
Grade 2	0	0	0.00
**Fatigue**	10	10	20.83
Grade 1	3	3	6.25
Grade 2	7	7	14.58
**Pain**	13	9	18.75
Grade 1	10	7	14.58
Grade 2	3	3	6.25
**Pyrexia**	6	5	10.42
Grade 1	6	5	10.42
Grade 2	0	0	0.00
**Swelling**	1	1	2.08
Grade 1	1	1	2.08
Grade 2	0	0	0.00
**Immune system disorders**	1	1	2.08
Grade 1	0	0	0.00
Grade 2	1	1	2.08
**Hypersensitivity**	1	1	2.08
Grade 1	0	0	0.00
Grade 2	1	1	2.08
**Infections and infestations**	1	1	2.08
Grade 1	0	0	0.00
Grade 2	1	1	2.08
**Pharyngitis**	1	1	2.08
Grade 1	0	0	0.00
Grade 2	1	1	2.08
**Metabolism and nutrition disorders**	1	1	2.08
Grade 1	0	0	0.00
Grade 2	1	1	2.08
**Dehydration**	1	1	2.08

Adverse Events	NVSI-06-08 (N=48)		
Severity	Cases	Number of participants	Percentage (%)
Grade 1	0	0	0.00
Grade 2	1	1	2.08
**Musculoskeletal and connective tissue disorders**	12	10	20.83
Grade 1	4	4	8.33
Grade 2	8	6	12.50
**Myalgia**	12	10	20.83
Grade 1	4	4	8.33
Grade 2	8	6	12.50
**Nervous system disorders**	14	8	16.67
Grade 1	6	5	10.42
Grade 2	8	5	10.42
**Ageusia**	1	1	2.08
Grade 1	0	0	0.00
Grade 2	1	1	2.08
**Anosmia**	1	1	2.08
Grade 1	0	0	0.00
Grade 2	1	1	2.08
**Dizziness**	1	1	2.08
Grade 1	1	1	2.08
Grade 2	0	0	0.00
**Headache**	9	7	14.58
Grade 1	4	4	8.33
Grade 2	5	4	8.33
**Hypoaesthesia**	1	1	2.08
Grade 1	0	0	0.00
Grade 2	1	1	2.08
**Syncope**	1	1	2.08
Grade 1	1	1	2.08
Grade 2	0	0	0.00
**Reproductive system and breast disorders**	3	2	4.17
Grade 1	0	0	0.00
Grade 2	3	2	4.17
**Dysmenorrhoea**	1	1	2.08
Grade 1	0	0	0.00
Grade 2	1	1	2.08
**Oligomenorrhoea**	2	1	2.08
Grade 1	0	0	0.00
Grade 2	2	1	2.08
**Respiratory, thoracic and mediastinal disorders**	29	11	22.92
Grade 1	8	6	12.50
Grade 2	21	9	18.75
**Cough**	8	7	14.58
Grade 1	3	2	4.17
Grade 2	5	5	10.42
**Dyspnoea**	3	2	4.17
Grade 1	2	2	4.17
Grade 2	1	1	2.08
**Nasal congestion**	6	5	10.42
Grade 1	0	0	0.00
Grade 2	6	5	10.42
**Oropharyngeal pain**	6	5	10.42
Grade 1	1	1	2.08
Grade 2	5	5	10.42
**Rhinitis allergic**	1	1	2.08

Adverse Events	NVSI-06-08 (N=48)		
Severity	Cases	Number of participants	Percentage (%)
Grade 1	0	0	0.00
Grade 2	1	1	2.08
**Rhinorrhoea**	5	5	10.42
Grade 1	2	2	4.17
Grade 2	3	3	6.25
**Skin and subcutaneous tissue disorders**	7	4	8.33
Grade 1	4	2	4.17
Grade 2	3	3	6.25
**Erythema**	2	2	4.17
Grade 1	2	2	4.17
Grade 2	0	0	0.00
**Pruritus**	4	2	4.17
Grade 1	2	1	2.08
Grade 2	2	2	4.17
**Rash erythematous**	1	1	2.08
Grade 1	0	0	0.00
Grade 2	1	1	2.08

(1) Adverse events were coded using MedDRA version 24.1.
(2) The data for this analysis is as of Jan. 25, 2022.

Table 1-3 Incidence and severity of various adverse events related to the study vaccine in the whole population during full immunization period (listed by SOC and PT) (SS)

Adverse Events	NVSI-06-08 (N=48)		
Severity	Cases	Number of participants	Percentage (%)
**TEAE**	60	20	41.67
Grade 1	35	17	35.42
Grade 2	25	10	20.83
**General disorders and administration site conditions**	22	13	27.08
Grade 1	17	12	25.00
Grade 2	5	4	8.33
**Fatigue**	5	5	10.42
Grade 1	3	3	6.25
Grade 2	2	2	4.17
**Pain**	13	9	18.75
Grade 1	10	7	14.58
Grade 2	3	3	6.25
**Pyrexia**	3	2	4.17
Grade 1	3	2	4.17
Grade 2	0	0	0.00
**Swelling**	1	1	2.08
Grade 1	1	1	2.08
Grade 2	0	0	0.00
**Musculoskeletal and connective tissue disorders**	9	7	14.58
Grade 1	4	4	8.33
Grade 2	5	3	6.25
**Myalgia**	9	7	14.58
Grade 1	4	4	8.33
Grade 2	5	3	6.25

Adverse Events	NVSI-06-08 (N=48)		
Severity	Cases	Number of participants	Percentage (%)
**Nervous system disorders**	9	6	12.50
Grade 1	6	5	10.42
Grade 2	3	2	4.17
**Dizziness**	1	1	2.08
Grade 1	1	1	2.08
Grade 2	0	0	0.00
**Headache**	7	5	10.42
Grade 1	4	4	8.33
Grade 2	3	2	4.17
**Syncope**	1	1	2.08
Grade 1	1	1	2.08
Grade 2	0	0	0.00
**Respiratory, thoracic and mediastinal disorders**	14	8	16.67
Grade 1	4	4	8.33
Grade 2	10	5	10.42
**Cough**	3	3	6.25
Grade 1	1	1	2.08
Grade 2	2	2	4.17
**Nasal congestion**	5	5	10.42
Grade 1	0	0	0.00
Grade 2	5	5	10.42
**Oropharyngeal pain**	4	3	6.25
Grade 1	1	1	2.08
Grade 2	3	3	6.25
**Rhinitis allergic**	2	2	4.17
Grade 1	2	2	4.17
Grade 2	0	0	0.00
**Skin and subcutaneous tissue disorders**	6	3	6.25
Grade 1	4	2	4.17
Grade 2	2	2	4.17
**Erythema**	2	2	4.17
Grade 1	2	2	4.17
Grade 2	0	0	0.00
**Pruritus**	4	2	4.17
Grade 1	2	1	2.08
Grade 2	2	2	4.17

(1) Adverse events were coded using MedDRA version 24.1.
(2) Study vaccine related means that the relationship between adverse events and study vaccine is "definitely related", "most likely related" or "likely related".
(3) The data for this analysis is as of Jan 25, 2022.

1.2.2. Adverse events in each time period

1.2.2.1. Adverse events within 30 minutes

No adverse events were observed within 30 minutes

1.2.2.2. Adverse events within 0-7 days

There were 17 participants (35.42%) with 52 cases within 0-7 days after the whole population was immunized. Adverse events related to study vaccine occurred 17 participants (35.42%) with 45 cases.

According to the classification of SOC and PT, the adverse events related to study vaccine vaccination were mainly general disorders and administration site conditions. Among them, the most common adverse reactions were pain at the vaccination site (18.75%), followed by fatigue (10.42%).

See Table 1-4 and Table 1-5 for details.

Table 1-4 Adverse events within 0-7 days of the whole population during full immunization period (listed by SOC and PT) (SS)

Adverse Events	NVSI-06-08 (N=48) Cases	Number of participants	Percentage (%)
**TEAE**	52	17	35.42
**Cardiac disorders**	1	1	2.08
Palpitations	1	1	2.08
**Gastrointestinal disorders**	1	1	2.08
Nausea	1	1	2.08
**General disorders and administration site conditions**	21	13	27.08
Fatigue	6	6	12.50
Pain	13	9	18.75
Pyrexia	1	1	2.08
Swelling	1	1	2.08
**Musculoskeletal and connective tissue disorders**	8	7	14.58
Myalgia	8	7	14.58
**Nervous system disorders**	8	6	12.50
Dizziness	1	1	2.08
Headache	5	5	10.42
Hypoaesthesia	1	1	2.08
Syncope	1	1	2.08
**Reproductive system and breast disorders**	1	1	2.08
Oligomenorrhoea	1	1	2.08
**Respiratory, thoracic and mediastinal disorders**	6	3	6.25
Cough	1	1	2.08
Dyspnoea	1	1	2.08
Nasal congestion	2	2	4.17
Oropharyngeal pain	1	1	2.08
Rhinitis allergic	1	1	2.08
**Skin and subcutaneous tissue disorders**	6	3	6.25
Erythema	2	2	4.17
Pruritus	4	2	4.17

(1) Adverse events were coded using MedDRA version 24.1.
(2) Occurrence time (day) = occurrence date of adverse events-vaccination date.
(3) Study vaccine related means that the relationship between adverse events and study vaccine is "definitely related", "most likely related" or " likely related".

(4) The data for this analysis is as of Jan 25, 2022.

Table 1-5 Adverse events related to study vaccine within 0-7 days of the whole population during full immunization period (listed by SOC and PT) (SS)

	NVSI-06-08 (N=48)		
**Adverse Events**	**Cases**	**Number of participants**	**Percentage (%)**
**TEAE**	45	17	35.42
**General disorders and administration site conditions**	20	13	27.08
Fatigue	5	5	10.42
Pain	13	9	18.75
Pyrexia	1	1	2.08
Swelling	1	1	2.08
**Musculoskeletal and connective tissue disorders**	8	7	14.58
Myalgia	8	7	14.58
**Nervous system disorders**	7	6	12.50
Dizziness	1	1	2.08
Headache	5	5	10.42
Syncope	1	1	2.08
**Respiratory, thoracic and mediastinal disorders**	4	2	4.17
Cough	1	1	2.08
Nasal congestion	2	2	4.17
Oropharyngeal pain	1	1	2.08
**Skin and subcutaneous tissue disorders**	6	3	6.25
Erythema	2	2	4.17
Pruritus	4	2	4.17

(1) Adverse events were coded using MedDRA version 24.1.
(2) Occurrence time (day) = occurrence date of adverse events-vaccination date.
(3) Study vaccine related means that the relationship between adverse events and study vaccine is "definitely related", "most likely related" or " likely related".
(4) The data for this analysis is as of Jan 25, 2022.

1.2.2.3. Various kinds of adverse events within 8-30 days

There were 11 participants (22.92%) with 49 cases within 8-30 days after the whole population was immunized. Adverse events related to study vaccine occurred 9 participants (18.75%) with 18 cases.

According to the classification of SOC and PT, the adverse events related to study vaccine vaccination were mainly respiratory, thoracic and mediastinal disorders. Among them, the most common adverse reactions were Nasal congestion (6.25%) and cough (6.25%).

See Table 1-6 and Table 1-7 for details.

Table 1-6 Adverse events occurred within 8 - 30 days during full immunization period for the whole population (by SOC and PT) (SS)

	NVSI-06-08 (N=48)		
**Adverse Events**	**Cases**	**Number of participants**	**Percentage (%)**
**TEAE**	49	11	22.92
**General disorders and administration site conditions**	10	5	10.42
Chills	1	1	2.08
Fatigue	4	4	8.33
Pyrexia	5	4	8.33
**Immune system disorders**	1	1	2.08
Hypersensitivity	1	1	2.08
**Infections and infestations**	1	1	2.08
Pharyngitis	1	1	2.08
**Metabolism and nutrition disorders**	1	1	2.08
Dehydration	1	1	2.08
**Musculoskeletal and connective tissue disorders**	4	4	8.33
Myalgia	4	4	8.33
**Nervous system disorders**	6	3	6.25
Ageusia	1	1	2.08
Anosmia	1	1	2.08
Headache	4	3	6.25
**Reproductive system and breast disorders**	2	2	4.17
Oligomenorrhoea	1	1	2.08
Dysmenorrhoea	1	1	2.08
**Respiratory, thoracic and mediastinal disorders**	23	10	20.83
Cough	7	6	12.50
Dyspnoea	2	2	4.17
Nasal congestion	4	3	6.25
Oropharyngeal pain	5	4	8.33
Rhinorrhoea	5	5	10.42
**Skin and subcutaneous tissue disorders**	1	1	2.08
Rash erythematous	1	1	2.08

(1) Adverse events were coded using MedDRA version 24.1.
(2) Occurrence time (day) = occurrence date of adverse events-vaccination date.
(3) The data for this analysis is as of Jan 25, 2022.

Table 1-7 Adverse events occurred within 8-30 days related to the study vaccine during full immunization period for the whole population(by SOC and PT) (SS)

	Inoculator (N=48)		
**Adverse Events**	**Cases**	**Number of participants**	**Percentage (%)**
**TEAE**	18	9	18.75
**General disorders and administration site conditions**	2	1	2.08

Adverse Events	Inoculator (N=48)		
	Cases	Number of participants	Percentage (%)
Pyrexia	2	1	2.08
**Musculoskeletal and connective tissue disorders**	1	1	2.08
Myalgia	1	1	2.08
**Nervous system disorders**	2	1	2.08
Headache	2	1	2.08
**Respiratory, thoracic and mediastinal disorders**	13	8	16.67
Cough	3	3	6.25
Dyspnoea	2	2	4.17
Nasal congestion	3	3	6.25
Oropharyngeal pain	3	2	4.17
Rhinorrhoea	2	2	4.17

(1) Adverse events were coded using MedDRA version 24.1.
(2) Occurrence time (day) = occurrence date of adverse events-vaccination date.
(3) Study vaccine related means that the relationship between adverse events and study vaccine is "definitely related", "most likely related" or " likely related".
(4) The data for this analysis is as of Jan 25, 2022.

1.2.3. Adverse events in each dose

The incidence of related to vaccine adverse events was 25.00% for the first dose, 22.92% for the second dose and 20.83% for the third dose.

See Table 1-8 and Table 1-9 for details.

Table 1-8 Incidence of adverse events of each dose in the whole population (SS)

Adverse Events	Inoculator			
	N	Cases	Number of participants	Percentage (%)
**TEAE**	48	101	22	45.83
First-dose	48	39	12	25.00
Second-dose	48	20	13	27.08
Third-dose	48	42	12	25.00

(1) The data for this analysis is as of Jan 25, 2022.

Table 1-9 Incidence of adverse events of related to the study vaccine each dose in the whole population (SS)

Adverse Events	Inoculator			
	N	Cases	Number of participants	Percentage (%)
**TEAE**	48	60	20	41.67
First-dose	48	26	12	25.00
Second-dose	48	15	11	22.92
Third-dose	48	19	10	20.83

(1) Vaccine-related means that the relationship between adverse events and vaccine-related is "definitely related", "likely related" or "likely related".

(2) The data for this analysis is as of January 25, 2022.

1.2.4 Solicited and unsolicited adverse events

There were 16 participants (33.33%) with 42 cases solicitation adverse events in this trial. Among them, the incidence of systemic adverse events related to vaccine was 20.83%. The incidence of local adverse events related to vaccine was 20.83%. The incidence of non-solicitation adverse events related to vaccine was 18.75%. See Table 1-10 for details.

Table 1-10 The incidence of adverse events in all subjects during full immunization period (Listed in terms of solicitation and non-solicitation)（SS）

	**NVSI-06-08** (N=48)		
Name of Adverse event	**Cases**	**Number of participants**	**Percentage (%)**
Solicited Adverse Events	42	16	33.33
Related	39	16	33.33
Systemic adverse events	24	11	22.92
Related	21	10	20.83
Local adverse events	18	10	20.83
Related	18	10	20.83
Unsolicited adverse events	59	12	25.00
Related	21	9	18.75

(1)Non-solicitation adverse events are coded by MedDRA version 24.1, and solicitation adverse events are listed according to the event names specified in the plan.
(2)The data of this analysis is up to January 25, 2022.

1.2.4.1 Solicited Adverse Events

Systemic Adverse Events Related to test vaccine:

After immunization of all subjects, the most common systemic adverse reaction related to test vaccine (systemic adverse reaction) was myalgia (14.58%), followed by fatigue (10.42%) and headache (10.42%). The severity of all kinds of systemic adverse reactions were grade 1 or grade 2.

See Table 1-11 for details.

Table 1-11 Distribution of severity of systemic adverse events related to test vaccine in all subjects during full immunization period (SS)

Adverse Event Name	**Inoculator (N=48)**		
Severity	**Cases**	**Number of participants**	**Percentage (%)**
Systemic Adverse Events Related to Test Vaccine	21	10	20.83
Grade 1	11	7	14.58
Grade 2	10	4	8.33
**Cough**	1	1	2.08

Adverse Event Name<br>Severity	Inoculator (N=48)		
	Cases	Number of participants	Percentage (%)
Grade 1	0	0	0.00
Grade 2	1	1	2.08
**Fatigue**	5	5	10.42
Grade 1	3	3	6.25
Grade 2	2	2	4.17
**Fever**	1	1	2.08
Grade 1	1	1	2.08
Grade 2	0	0	0.00
**Headache**	5	5	10.42
Grade 1	3	3	6.25
Grade 2	2	2	4.17
**Myalgia**	8	7	14.58
Grade 1	4	4	8.33
Grade 2	4	3	6.25
**Pruritus**	1	1	2.08
Grade 1	0	0	0.00
Grade 2	1	1	2.08

(1) Unsolicited adverse events are coded using MedDRA version 24.1, and solicited adverse events are listed according to the event name specified in the protocol.
(2) Vaccine-related means that the relationship between adverse events and vaccine-related is "definitely related", "likely related" or "likely related".
(3) The data for this analysis is as of January 25, 2022.

Local adverse events related to test vaccine:

After immunization of all subjects, the most common local adverse events (local adverse reactions) related to test vaccine were pain, occurred 9 participants (18.75%) with 13 cases. The incidence of all other local adverse reactions was 2.08%. The severity of all kinds of local adverse reactions were mainly grade 1. No local adverse reactions with grade 3 occurred in this trial. See Table 1-12 for details.

Table 1-12 Distribution of severity of local adverse events related to test vaccine in all subjects during full immunization period (SS)

Name of Adverse Event<br>Severity	Inoculator (N=48)		
	Cases	Number of participants	Percentage (%)
Local adverse events related to test vaccine	18	10	20.83
Grade 1	14	8	16.67
Grade 2	4	4	8.33
**Pain**	13	9	18.75
Grade 1	10	7	14.58
Grade 2	3	3	6.25
**Pruritus**	3	1	2.08
Grade 1	2	1	2.08

Name of Adverse Event / Severity	Inoculator (N=48) Cases	Number of participants	Percentage (%)
Grade 2	1	1	2.08
**Redness**	1	1	2.08
Grade 1	1	1	2.08
Grade 2	0	0	0.00
**Swelling**	1	1	2.08
Grade 1	1	1	2.08
Grade 2	0	0	0.00

(1) Unsolicited adverse events are coded using MedDRA version 24.1, and solicited adverse events are listed according to the event name specified in the protocol.
(2) Vaccine-related means that the relationship between adverse events and vaccine-related is "definitely related", "likely related" or "likely related".
(3) The data for this analysis is as of January 25, 2022.

1.2.4.2 Unsolicited adverse events Related to test vaccine

Unsolicited adverse events related to test vaccine occurred in 9 participants (18.75%) with 21 cases. The main diseases are Respiratory, thoracic and mediastinal disorders. The incidence of Unsolicited adverse reaction was 16.67%. The severity of all kinds of non-solicitation adverse events related to test vaccine was Grade 1 or Grade 2. See Table 1-13 for details.

Table 1-13 Severity distribution of unsolicited adverse events related to test vaccine during full immunization period for all subjects (SS)

Name of Adverse Event / Severity	Inoculator (N=48) Cases	Number of participants	Percentage (%)
**Unsolicited adverse events related to test vaccine**	21	9	18.75
Grade 1	10	6	12.50
Grade 2	11	5	10.42
**General disorders and administration site conditions**	2	1	2.08
Grade 1	2	1	2.08
Grade 2	0	0	0.00
**Pyrexia**	2	1	2.08
Grade 1	2	1	2.08
Grade 2	0	0	0.00
**Musculoskeletal and connective tissue disorders**	1	1	2.08
Grade 1	0	0	0.00
Grade 2	1	1	2.08
**Myalgia**	1	1	2.08
Grade 1	0	0	0.00
Grade 2	1	1	2.08
**Nervous system disorders**	4	2	4.17
Grade 1	3	2	4.17
Grade 2	1	1	2.08
**Dizziness**	1	1	2.08

Name of Adverse Event	Inoculator (N=48)		
Severity	Cases	Number of participants	Percentage (%)
Grade 1	1	1	2.08
Grade 2	0	0	0.00
**Headache**	2	1	2.08
Grade 1	1	1	2.08
Grade 2	1	1	2.08
**Syncope**	1	1	2.08
Grade 1	1	1	2.08
Grade 2	0	0	0.00
**Respiratory, thoracic and mediastinal disorders**	13	8	16.67
Grade 1	4	4	8.33
Grade 2	9	5	10.42
**Cough**	2	2	4.17
Grade 1	1	1	2.08
Grade 2	1	1	2.08
**Nasal Congestion**	5	5	10.42
Grade 1	0	0	0.00
Grade 2	5	5	10.42
**Oropharyngeal pain**	4	3	6.25
Grade 1	1	1	2.08
Grade 2	3	3	6.25
**Rhinorrhoea**	2	2	4.17
Grade 1	2	2	4.17
Grade 2	0	0	0.00
**Skin and subcutaneous tissue disorders**	1	1	2.08
Grade 1	1	1	2.08
Grade 2	0	0	0.00
**Erythema**	1	1	2.08
Grade 1	1	1	2.08
Grade 2	0	0	0.00

(1) Unsolicited adverse events are coded using MedDRA version 24.1.
(2) Vaccine-related means that the relationship between adverse events and vaccine-related is "definitely related", "most likely related" or "likely related".
(3) The data for this analysis is as of January 25, 2022.

1.3 Deaths, other serious adverse event, and other significant adverse events

1.3.1 List and narrative of death

No deaths occurred in this clinical trial.

1.3.2 List and narrative of serious adverse event other than death

In this clinical trial, no serious adverse events have been observed.

1.4 Safety Summary

1) The incidence of total adverse events related to the test vaccine was 41.67%, the severity of grade 1 was 35.42%, grade 2 was 20.83%,

2) No adverse events were observed within 30 minutes; Adverse events related to test vaccine are mainly concentrated in 0-7 days; Within 0-7 days, the incidence of adverse reactions was 35.42%; The incidence of adverse reactions in the 8-30 days was 18.75%.

3）The incidence of adverse events related to vaccination was 25.00%, 22.92% and 20.83% for the first, second and third doses, respectively.

4) The incidence of solicited systemic adverse events related to the test vaccine was 20.83%. Myalgia (14.58%), headache (10.42%) and fatigue (10.42%) were the main ones. The severity was grade 1 or grade 2. The incidence of solicited local adverse reactions was 20.83%, of which mainly pain (18.75%). The incidence of all other local adverse reactions was 2.08%. There were no local adverse reactions of severity grade 3 in this trial.

5) The incidence of unsolicited adverse events related to the study vaccine was 18.75%; Mainly in respiratory, thoracic and mediastinal disorders (16.67%), and the severity was grade 1 or grade 2.

6) During the clinical trial, no subjects died, no adverse events of special interest occurred, and no serious adverse event occurred.

2. Discussion And Overall Conclusions (30 Days After Vaccination)

This clinical study evaluated the safety of the NVSI-06-08 in subjects 18 years of age and above.

Security:

A total of 48 subjects were included in the SS set of this clinical study.

Adverse reactions mainly occur within 0-7 days after vaccination, mainly pain at the vaccination site, myalgia (non-vaccination site), fatigue and headache, and the severity is grade 1 or 2. There were no adverse events of severity grade 3 in this trial. which is consistent with the adverse reactions of conventional vaccination consistent.

Supplementary Notes

Supplementary Note 2. Safety reports for phase 2 trial.

Clinical Trial of Booster Immunization of Recombinant COVID-19 Vaccine (CHO cells, NVSI-06-08) and Inactivated COVID-19 Vaccine (Vero cells) in Subjects Aged 18 and Above

Safety Summary Report (30 Days after Full Vaccination)

Name of test vaccine: Recombinant COVID-19 vaccine (CHO cells, NVSI-06-08)

Study Number: CNBG-REC-2021003

Study start time: October 23, 2021

Study Completion Time: Ongoing

Main researcher: Nawal Al Kaabi

Study Responsible: Sheikh Khalifa Medical City, SEHA, Abu Dhabi, United Arab Emirates

Reporting date: January 28, 2022

Source data stored at Sheikh Khalifa Medical City

Version number: V1.0

1. Safety Assessment

1.1. Exposure degree

896 subjects in the recombinant vaccine group received a booster dose of the study vaccine NVSI-06-08 and 904 subjects in the inactivated vaccine group were vaccinated with a booster dose of BBIBP-CorV as control according to the trial protocol

1.2. Adverse events

1.2.1. Total adverse events

In the recombinant vaccine group of 896 subjects, 179 cases (19.98%) with 353 adverse events were observed, of which 157 cases (17.52%) with 289 adverse events were related to the study vaccine, and 5 cases (0.56%) with 6 adverse events related to the study vaccine were of grade 3 and above. In the inactivated vaccine group of 904 subjects, a total of 153 cases (16.92%) with 282 adverse events were reported, of which 135 cases (14.93%) with 250 adverse events were related to the study vaccine, and 2 cases (0.22%) with 4 adverse events related to the study vaccine were of grade 3 and above. There was no statistically significant difference in the incidence of adverse events between these two groups.

In the recombinant vaccine group, the incidence of adverse events of severity grade 1, grade 2, grade 3, and grade 4 was 16.85%, 7.14%, 0.67%, and 0.11%, respectively, and the incidence of adverse events of grade 1, grade 2, grade 3, and grade 4 related to the study vaccine was 15.07%, 5.58%, 0.56%, and 0.00% respectively. In the inactivated vaccine group, the incidence of adverse events of grade 1, 2, 3, and 4 were 13.27%, 6.75%, 0.33%, and 0.00%, respectively, and the incidence of adverse events of grade 1, grade 2, grade 3, and grade 4 related to the study vaccine was 12.06%, 5.42%, 0.22%, and 0.00% respectively. There was a statistically significant difference in the incidence of adverse events of grade 1 between these two groups (P=0.0350). For other adverse events, there was no statistically significant difference between two groups.

In the recombinant vaccine group, there were 3 cases (0.33%) with grade 3 vaccine-related adverse events of fever, 2 cases (0.22%) with grade 3 vaccine-related adverse events of myalgia, and 1 case (0.11%) with grade 3 adverse events of pharyngalgia. While, in the inactivated vaccine group, there were 2 cases (0.22%) with grade 3 vaccine-related adverse events of fever, 1 case (0.11%) with grade 3 adverse events of chest pain, and 1 case (0.11%) with grade 3 adverse events of myalgia.

The adverse events related to the study vaccine were mainly systemic diseases and

various reactions at the administration site. There were 170 cases (9.44%) with 212 adverse events, including 91 cases (10.16%) with 112 adverse events in the recombinant vaccine group and 79 cases (8.74%) with 100 adverse events in the inactivated vaccine group. There was no significant difference between these two groups. The main adverse reactions at the vaccination site were pain, whose incidence rate was 4.69% and 3.98% in the recombinant vaccine group and the inactivated vaccine group, respectively. The other most common adverse reaction on the vaccination site was fatigue, followed by fever. For the adverse reaction fatigue, the incidence rate in the recombinant vaccine group and inactivated vaccine group were 3.13% and 2.88%, respectively. For the fever, the incidence rate in the recombinant vaccine group and inactivated vaccine group were 3.01% and 2.88%, respectively.

No adverse events leading to dropout were observed. See Table 1-1, Table 1-2, and Table 1-3 for details.

Table 1-1 Total incidence of adverse events in the whole population during the period of booster dose immunization (SS)

	Recombinant vaccine group (N=896)			Inactivated vaccine group (N=904)			Total (N=1800)			
	Adverse events	Cases	Incidence (%)	Adverse events	Cases	Incidence (%)	Adverse events	Cases	Incidence (%)	P Value[1]
Adverse events occurring during the booster period	353	179	19.98	282	153	16.92	635	332	18.44	0.1009
Adverse events related to study vaccine	289	157	17.52	250	135	14.93	539	292	16.22	0.1417
Adverse events unrelated to study vaccine	64	39	4.35	32	27	2.99	96	66	3.67	0.1333
Adverse events of grade 3 and above	20	6	0.67	5	3	0.33	25	9	0.50	0.3405
Study vaccine related adverse events of grade 3 and above	6	5	0.56	4	2	0.22	10	7	0.39	0.2861

[1]P value was calculated by Fisher exact test.

(1) Adverse events were coded using MedDRA version 24.1.

(2) Study vaccine-related means that the relationship between adverse events and study vaccine is "definitely related," "most likely related," or "likely related."

(3) Not related to the study vaccine means that the relationship between the adverse event and the study vaccine is "definitely not related" or "may not be related."

(4) Adverse events during the period of booster dose immunization are defined as adverse events that occurred within 30 days after the booster dose vaccination.

(5) This analysis data is up to January 25, 2022.

Table 1-2 Incidence and severity of various adverse events in the whole population during the period of booster dose immunization (listed per SOC and PT) (SS)

Adverse Events	Recombinant vaccine group (N=896)			Inactivated vaccine group (N=904)			Total (N=1800)			
Severity	Adverse events	Cases	Incidence (%)	Adverse events	Cases	Incidence (%)	Adverse events	Cases	Incidence (%)	P Value[1]
**TEAE**	353	179	19.98	282	153	16.92	635	332	18.44	0.1009
Grade 1	230	151	16.85	183	120	13.27	413	271	15.06	0.0350
Grade 2	103	64	7.14	94	61	6.75	197	125	6.94	0.7812
Grade 3	19	6	0.67	5	3	0.33	24	9	0.50	0.3405
Grade 4	1	1	0.11	0	0	0.00	1	1	0.06	0.4978
Grade 5	0	0	0.00	0	0	0.00	0	0	0.00	1.0000
Grade 2 and above	123	68	7.59	99	62	6.86	222	130	7.22	0.5853
Grade 3 and above	20	6	0.67	5	3	0.33	25	9	0.50	0.3405
**Systemic diseases and various reactions at the administration site**	131	102	11.38	106	83	9.18	237	185	10.28	0.1401
Grade 1	101	86	9.60	87	74	8.19	188	160	8.89	0.3204
Grade 2	24	22	2.46	15	14	1.55	39	36	2.00	0.1816
Grade 3	6	4	0.45	4	3	0.33	10	7	0.39	0.7249
Grade 4	0	0	0.00	0	0	0.00	0	0	0.00	1.0000
Grade 5	0	0	0.00	0	0	0.00	0	0	0.00	1.0000
Grade 2 and above	30	26	2.90	19	16	1.77	49	42	2.33	0.1204
Grade 3 and above	6	4	0.45	4	3	0.33	10	7	0.39	0.7249
Pain at vaccination site	42	42	4.69	36	36	3.98	78	78	4.33	0.4888
Grade 1	36	36	4.02	32	32	3.54	68	68	3.78	0.6225
Grade 2	6	6	0.67	4	4	0.44	10	10	0.56	0.5460
Grade 3	0	0	0.00	0	0	0.00	0	0	0.00	1.0000
Grade 4	0	0	0.00	0	0	0.00	0	0	0.00	1.0000
Grade 5	0	0	0.00	0	0	0.00	0	0	0.00	1.0000
Grade 2 and above	6	6	0.67	4	4	0.44	10	10	0.56	0.5460
Grade 3 and above	0	0	0.00	0	0	0.00	0	0	0.00	1.0000
Fever	39	37	4.13	30	29	3.21	69	66	3.67	0.3176
Grade 1	31	30	3.35	26	25	2.77	57	55	3.06	0.4961
Grade 2	5	5	0.56	2	2	0.22	7	7	0.39	0.2861
Grade 3	3	3	0.33	2	2	0.22	5	5	0.28	0.6858
Grade 4	0	0	0.00	0	0	0.00	0	0	0.00	1.0000
Grade 5	0	0	0.00	0	0	0.00	0	0	0.00	1.0000
Grade 2 and above	8	8	0.89	4	4	0.44	12	12	0.67	0.2633
Grade 3 and above	3	3	0.33	2	2	0.22	5	5	0.28	0.6858
Fatigue	35	34	3.79	27	27	2.99	62	61	3.39	0.3641
Grade 1	23	23	2.57	18	18	1.99	41	41	2.28	0.4337
Grade 2	10	10	1.12	9	9	1.00	19	19	1.06	0.8222
Grade 3	2	2	0.22	0	0	0.00	2	2	0.11	0.2476
Grade 4	0	0	0.00	0	0	0.00	0	0	0.00	1.0000
Grade 5	0	0	0.00	0	0	0.00	0	0	0.00	1.0000
Grade 2 and above	12	12	1.34	9	9	1.00	21	21	1.17	0.5193
Grade 3 and above	2	2	0.22	0	0	0.00	2	2	0.11	0.2476
Chest pain	2	2	0.22	5	5	0.55	7	7	0.39	0.4522
Grade 1	1	1	0.11	3	3	0.33	4	4	0.22	0.6246
Grade 2	1	1	0.11	0	0	0.00	1	1	0.06	0.4978
Grade 3	0	0	0.00	2	2	0.22	2	2	0.11	0.4997

Adverse Events<br>Severity	Recombinant vaccine group (N=896)			Inactivated vaccine group (N=904)			Total (N=1800)			P Value[1]
	Adverse events	Cases	Incidence (%)	Adverse events	Cases	Incidence (%)	Adverse events	Cases	Incidence (%)	
Grade 4	0	0	0.00	0	0	0.00	0	0	0.00	1.0000
Grade 5	0	0	0.00	0	0	0.00	0	0	0.00	1.0000
Grade 2 and above	1	1	0.11	2	2	0.22	3	3	0.17	1.0000
Grade 3 and above	0	0	0.00	2	2	0.22	2	2	0.11	0.4997
Feeling hot	4	4	0.45	0	0	0.00	4	4	0.22	0.0612
Grade 1	3	3	0.33	0	0	0.00	3	3	0.17	0.1231
Grade 2	1	1	0.11	0	0	0.00	1	1	0.06	0.4978
Grade 3	0	0	0.00	0	0	0.00	0	0	0.00	1.0000
Grade 4	0	0	0.00	0	0	0.00	0	0	0.00	1.0000
Grade 5	0	0	0.00	0	0	0.00	0	0	0.00	1.0000
Grade 2 and above	1	1	0.11	0	0	0.00	1	1	0.06	0.4978
Grade 3 and above	0	0	0.00	0	0	0.00	0	0	0.00	1.0000
Pain	3	3	0.33	1	1	0.11	4	4	0.22	0.3724
Grade 1	3	3	0.33	1	1	0.11	4	4	0.22	0.3724
Grade 2	0	0	0.00	0	0	0.00	0	0	0.00	1.0000
Grade 3	0	0	0.00	0	0	0.00	0	0	0.00	1.0000
Grade 4	0	0	0.00	0	0	0.00	0	0	0.00	1.0000
Grade 5	0	0	0.00	0	0	0.00	0	0	0.00	1.0000
Grade 2 and above	0	0	0.00	0	0	0.00	0	0	0.00	1.0000
Grade 3 and above	0	0	0.00	0	0	0.00	0	0	0.00	1.0000
Itching at vaccination site	2	2	0.22	2	2	0.22	4	4	0.22	1.0000
Grade 1	1	1	0.11	2	2	0.22	3	3	0.17	1.0000
Grade 2	1	1	0.11	0	0	0.00	1	1	0.06	0.4978
Grade 3	0	0	0.00	0	0	0.00	0	0	0.00	1.0000
Grade 4	0	0	0.00	0	0	0.00	0	0	0.00	1.0000
Grade 5	0	0	0.00	0	0	0.00	0	0	0.00	1.0000
Grade 2 and above	1	1	0.11	0	0	0.00	1	1	0.06	0.4978
Grade 3 and above	0	0	0.00	0	0	0.00	0	0	0.00	1.0000
Spot erythema at vaccination site	2	2	0.22	0	0	0.00	2	2	0.11	0.2476
Grade 1	2	2	0.22	0	0	0.00	2	2	0.11	0.2476
Grade 2	0	0	0.00	0	0	0.00	0	0	0.00	1.0000
Grade 3	0	0	0.00	0	0	0.00	0	0	0.00	1.0000
Grade 4	0	0	0.00	0	0	0.00	0	0	0.00	1.0000
Grade 5	0	0	0.00	0	0	0.00	0	0	0.00	1.0000
Grade 2 and above	0	0	0.00	0	0	0.00	0	0	0.00	1.0000
Grade 3 and above	0	0	0.00	0	0	0.00	0	0	0.00	1.0000
Swelling of vaccination site	1	1	0.11	1	1	0.11	2	2	0.11	1.0000
Grade 1	1	1	0.11	1	1	0.11	2	2	0.11	1.0000
Grade 2	0	0	0.00	0	0	0.00	0	0	0.00	1.0000
Grade 3	0	0	0.00	0	0	0.00	0	0	0.00	1.0000
Grade 4	0	0	0.00	0	0	0.00	0	0	0.00	1.0000
Grade 5	0	0	0.00	0	0	0.00	0	0	0.00	1.0000
Grade 2 and above	0	0	0.00	0	0	0.00	0	0	0.00	1.0000
Grade 3 and above	0	0	0.00	0	0	0.00	0	0	0.00	1.0000
Chills	1	1	0.11	0	0	0.00	1	1	0.06	0.4978
Grade 1	0	0	0.00	0	0	0.00	0	0	0.00	1.0000
Grade 2	0	0	0.00	0	0	0.00	0	0	0.00	1.0000
Grade 3	1	1	0.11	0	0	0.00	1	1	0.06	0.4978
Grade 4	0	0	0.00	0	0	0.00	0	0	0.00	1.0000
Grade 5	0	0	0.00	0	0	0.00	0	0	0.00	1.0000

Adverse Events	Recombinant vaccine group (N=896)			Inactivated vaccine group (N=904)			Total (N=1800)			
Severity	Adverse events	Cases	Incidence (%)	Adverse events	Cases	Incidence (%)	Adverse events	Cases	Incidence (%)	P Value[1]
Grade 2 and above	1	1	0.11	0	0	0.00	1	1	0.06	0.4978
Grade 3 and above	1	1	0.11	0	0	0.00	1	1	0.06	0.4978
Nodule	0	0	0.00	1	1	0.11	1	1	0.06	1.0000
Grade 1	0	0	0.00	1	1	0.11	1	1	0.06	1.0000
Grade 2	0	0	0.00	0	0	0.00	0	0	0.00	1.0000
Grade 3	0	0	0.00	0	0	0.00	0	0	0.00	1.0000
Grade 4	0	0	0.00	0	0	0.00	0	0	0.00	1.0000
Grade 5	0	0	0.00	0	0	0.00	0	0	0.00	1.0000
Grade 2 and above	0	0	0.00	0	0	0.00	0	0	0.00	1.0000
Grade 3 and above	0	0	0.00	0	0	0.00	0	0	0.00	1.0000
Influenza-like disease	0	0	0.00	1	1	0.11	1	1	0.06	1.0000
Grade 1	0	0	0.00	1	1	0.11	1	1	0.06	1.0000
Grade 2	0	0	0.00	0	0	0.00	0	0	0.00	1.0000
Grade 3	0	0	0.00	0	0	0.00	0	0	0.00	1.0000
Grade 4	0	0	0.00	0	0	0.00	0	0	0.00	1.0000
Grade 5	0	0	0.00	0	0	0.00	0	0	0.00	1.0000
Grade 2 and above	0	0	0.00	0	0	0.00	0	0	0.00	1.0000
Grade 3 and above	0	0	0.00	0	0	0.00	0	0	0.00	1.0000
Rash at vaccination site	0	0	0.00	1	1	0.11	1	1	0.06	1.0000
Grade 1	0	0	0.00	1	1	0.11	1	1	0.06	1.0000
Grade 2	0	0	0.00	0	0	0.00	0	0	0.00	1.0000
Grade 3	0	0	0.00	0	0	0.00	0	0	0.00	1.0000
Grade 4	0	0	0.00	0	0	0.00	0	0	0.00	1.0000
Grade 5	0	0	0.00	0	0	0.00	0	0	0.00	1.0000
Grade 2 and above	0	0	0.00	0	0	0.00	0	0	0.00	1.0000
Grade 3 and above	0	0	0.00	0	0	0.00	0	0	0.00	1.0000
Swelling of vaccination site	0	0	0.00	1	1	0.11	1	1	0.06	1.0000
Grade 1	0	0	0.00	1	1	0.11	1	1	0.06	1.0000
Grade 2	0	0	0.00	0	0	0.00	0	0	0.00	1.0000
Grade 3	0	0	0.00	0	0	0.00	0	0	0.00	1.0000
Grade 4	0	0	0.00	0	0	0.00	0	0	0.00	1.0000
Grade 5	0	0	0.00	0	0	0.00	0	0	0.00	1.0000
Grade 2 and above	0	0	0.00	0	0	0.00	0	0	0.00	1.0000
Grade 3 and above	0	0	0.00	0	0	0.00	0	0	0.00	1.0000
**Various nervous system diseases**	72	63	7.03	52	48	5.31	124	111	6.17	0.1418
Grade 1	41	39	4.35	24	23	2.54	65	62	3.44	0.0387
Grade 2	28	27	3.01	28	26	2.88	56	53	2.94	0.8899
Grade 3	3	1	0.11	0	0	0.00	3	1	0.06	0.4978
Grade 4	0	0	0.00	0	0	0.00	0	0	0.00	1.0000
Grade 5	0	0	0.00	0	0	0.00	0	0	0.00	1.0000
Grade 2 and above	31	28	3.13	28	26	2.88	59	54	3.00	0.7837
Grade 3 and above	3	1	0.11	0	0	0.00	3	1	0.06	0.4978
Headache	61	57	6.36	44	43	4.76	105	100	5.56	0.1500
Grade 1	34	33	3.68	18	18	1.99	52	51	2.83	0.0332
Grade 2	26	25	2.79	26	25	2.77	52	50	2.78	1.0000
Grade 3	1	1	0.11	0	0	0.00	1	1	0.06	0.4978
Grade 4	0	0	0.00	0	0	0.00	0	0	0.00	1.0000
Grade 5	0	0	0.00	0	0	0.00	0	0	0.00	1.0000
Grade 2 and above	27	26	2.90	26	25	2.77	53	51	2.83	0.8879
Grade 3 and above	1	1	0.11	0	0	0.00	1	1	0.06	0.4978

Adverse Events	Recombinant vaccine group (N=896)			Inactivated vaccine group (N=904)			Total (N=1800)			
Severity	Adverse events	Cases	Incidence (%)	Adverse events	Cases	Incidence (%)	Adverse events	Cases	Incidence (%)	P Value[1]
Dizziness	4	4	0.45	4	4	0.44	8	8	0.44	1.0000
Grade 1	3	3	0.33	3	3	0.33	6	6	0.33	1.0000
Grade 2	1	1	0.11	1	1	0.11	2	2	0.11	1.0000
Grade 3	0	0	0.00	0	0	0.00	0	0	0.00	1.0000
Grade 4	0	0	0.00	0	0	0.00	0	0	0.00	1.0000
Grade 5	0	0	0.00	0	0	0.00	0	0	0.00	1.0000
Grade 2 and above	1	1	0.11	1	1	0.11	2	2	0.11	1.0000
Grade 3 and above	0	0	0.00	0	0	0.00	0	0	0.00	1.0000
Sleepiness	3	3	0.33	2	2	0.22	5	5	0.28	0.6858
Grade 1	3	3	0.33	2	2	0.22	5	5	0.28	0.6858
Grade 2	0	0	0.00	0	0	0.00	0	0	0.00	1.0000
Grade 3	0	0	0.00	0	0	0.00	0	0	0.00	1.0000
Grade 4	0	0	0.00	0	0	0.00	0	0	0.00	1.0000
Grade 5	0	0	0.00	0	0	0.00	0	0	0.00	1.0000
Grade 2 and above	0	0	0.00	0	0	0.00	0	0	0.00	1.0000
Grade 3 and above	0	0	0.00	0	0	0.00	0	0	0.00	1.0000
Hypoaesthesia	2	2	0.22	0	0	0.00	2	2	0.11	0.2476
Grade 1	1	1	0.11	0	0	0.00	1	1	0.06	0.4978
Grade 2	1	1	0.11	0	0	0.00	1	1	0.06	0.4978
Grade 3	0	0	0.00	0	0	0.00	0	0	0.00	1.0000
Grade 4	0	0	0.00	0	0	0.00	0	0	0.00	1.0000
Grade 5	0	0	0.00	0	0	0.00	0	0	0.00	1.0000
Grade 2 and above	1	1	0.11	0	0	0.00	1	1	0.06	0.4978
Grade 3 and above	0	0	0.00	0	0	0.00	0	0	0.00	1.0000
Dysarthria	0	0	0.00	1	1	0.11	1	1	0.06	1.0000
Grade 1	0	0	0.00	0	0	0.00	0	0	0.00	1.0000
Grade 2	0	0	0.00	1	1	0.11	1	1	0.06	1.0000
Grade 3	0	0	0.00	0	0	0.00	0	0	0.00	1.0000
Grade 4	0	0	0.00	0	0	0.00	0	0	0.00	1.0000
Grade 5	0	0	0.00	0	0	0.00	0	0	0.00	1.0000
Grade 2 and above	0	0	0.00	1	1	0.11	1	1	0.06	1.0000
Grade 3 and above	0	0	0.00	0	0	0.00	0	0	0.00	1.0000
Muscle weakness	0	0	0.00	1	1	0.11	1	1	0.06	1.0000
Grade 1	0	0	0.00	1	1	0.11	1	1	0.06	1.0000
Grade 2	0	0	0.00	0	0	0.00	0	0	0.00	1.0000
Grade 3	0	0	0.00	0	0	0.00	0	0	0.00	1.0000
Grade 4	0	0	0.00	0	0	0.00	0	0	0.00	1.0000
Grade 5	0	0	0.00	0	0	0.00	0	0	0.00	1.0000
Grade 2 and above	0	0	0.00	0	0	0.00	0	0	0.00	1.0000
Grade 3 and above	0	0	0.00	0	0	0.00	0	0	0.00	1.0000
Loss of taste	1	1	0.11	0	0	0.00	1	1	0.06	0.4978
Grade 1	0	0	0.00	0	0	0.00	0	0	0.00	1.0000
Grade 2	0	0	0.00	0	0	0.00	0	0	0.00	1.0000
Grade 3	1	1	0.11	0	0	0.00	1	1	0.06	0.4978
Grade 4	0	0	0.00	0	0	0.00	0	0	0.00	1.0000
Grade 5	0	0	0.00	0	0	0.00	0	0	0.00	1.0000
Grade 2 and above	1	1	0.11	0	0	0.00	1	1	0.06	0.4978
Grade 3 and above	1	1	0.11	0	0	0.00	1	1	0.06	0.4978
Loss of smell	1	1	0.11	0	0	0.00	1	1	0.06	0.4978
Grade 1	0	0	0.00	0	0	0.00	0	0	0.00	1.0000
Grade 2	0	0	0.00	0	0	0.00	0	0	0.00	1.0000

Adverse Events	Recombinant vaccine group (N=896)			Inactivated vaccine group (N=904)			Total (N=1800)			
Severity	Adverse events	Cases	Incidence (%)	Adverse events	Cases	Incidence (%)	Adverse events	Cases	Incidence (%)	P Value[1]
Grade 3	1	1	0.11	0	0	0.00	1	1	0.06	0.4978
Grade 4	0	0	0.00	0	0	0.00	0	0	0.00	1.0000
Grade 5	0	0	0.00	0	0	0.00	0	0	0.00	1.0000
Grade 2 and above	1	1	0.11	0	0	0.00	1	1	0.06	0.4978
Grade 3 and above	1	1	0.11	0	0	0.00	1	1	0.06	0.4978
**Respiratory, thoracic and mediastinal disorders**	61	40	4.46	60	39	4.31	121	79	4.39	0.9087
Grade 1	33	28	3.13	33	28	3.10	66	56	3.11	1.0000
Grade 2	23	15	1.67	27	18	1.99	50	33	1.83	0.7260
Grade 3	4	2	0.22	0	0	0.00	4	2	0.11	0.2476
Grade 4	1	1	0.11	0	0	0.00	1	1	0.06	0.4978
Grade 5	0	0	0.00	0	0	0.00	0	0	0.00	1.0000
Grade 2 and above	28	16	1.79	27	18	1.99	55	34	1.89	0.8629
Grade 3 and above	5	2	0.22	0	0	0.00	5	2	0.11	0.2476
Cough	33	30	3.35	28	26	2.88	61	56	3.11	0.5894
Grade 1	20	19	2.12	16	15	1.66	36	34	1.89	0.4937
Grade 2	11	11	1.23	12	12	1.33	23	23	1.28	1.0000
Grade 3	1	1	0.11	0	0	0.00	1	1	0.06	0.4978
Grade 4	1	1	0.11	0	0	0.00	1	1	0.06	0.4978
Grade 5	0	0	0.00	0	0	0.00	0	0	0.00	1.0000
Grade 2 and above	13	13	1.45	12	12	1.33	25	25	1.39	0.8433
Grade 3 and above	2	2	0.22	0	0	0.00	2	2	0.11	0.2476
Runny nose	10	10	1.12	10	10	1.11	20	20	1.11	1.0000
Grade 1	4	4	0.45	6	6	0.66	10	10	0.56	0.7532
Grade 2	6	6	0.67	4	4	0.44	10	10	0.56	0.5460
Grade 3	0	0	0.00	0	0	0.00	0	0	0.00	1.0000
Grade 4	0	0	0.00	0	0	0.00	0	0	0.00	1.0000
Grade 5	0	0	0.00	0	0	0.00	0	0	0.00	1.0000
Grade 2 and above	6	6	0.67	4	4	0.44	10	10	0.56	0.5460
Grade 3 and above	0	0	0.00	0	0	0.00	0	0	0.00	1.0000
Oropharyngeal Pain	10	9	1.00	7	7	0.77	17	16	0.89	0.6261
Grade 1	4	4	0.45	3	3	0.33	7	7	0.39	0.7249
Grade 2	4	4	0.45	4	4	0.44	8	8	0.44	1.0000
Grade 3	2	2	0.22	0	0	0.00	2	2	0.11	0.2476
Grade 4	0	0	0.00	0	0	0.00	0	0	0.00	1.0000
Grade 5	0	0	0.00	0	0	0.00	0	0	0.00	1.0000
Grade 2 and above	6	6	0.67	4	4	0.44	10	10	0.56	0.5460
Grade 3 and above	2	2	0.22	0	0	0.00	2	2	0.11	0.2476
Nasal congestion	5	5	0.56	8	8	0.88	13	13	0.72	0.5797
Grade 1	4	4	0.45	4	4	0.44	8	8	0.44	1.0000
Grade 2	1	1	0.11	4	4	0.44	5	5	0.28	0.3743
Grade 3	0	0	0.00	0	0	0.00	0	0	0.00	1.0000
Grade 4	0	0	0.00	0	0	0.00	0	0	0.00	1.0000
Grade 5	0	0	0.00	0	0	0.00	0	0	0.00	1.0000
Grade 2 and above	1	1	0.11	4	4	0.44	5	5	0.28	0.3743
Grade 3 and above	0	0	0.00	0	0	0.00	0	0	0.00	1.0000
Difficulty breathing	1	1	0.11	4	4	0.44	5	5	0.28	0.3743
Grade 1	0	0	0.00	2	2	0.22	2	2	0.11	0.4997

Adverse Events	Recombinant vaccine group (N=896)			Inactivated vaccine group (N=904)			Total (N=1800)			
Severity	Adverse events	Cases	Incidence (%)	Adverse events	Cases	Incidence (%)	Adverse events	Cases	Incidence (%)	P Value[1]
Grade 2	1	1	0.11	2	2	0.22	3	3	0.17	1.0000
Grade 3	0	0	0.00	0	0	0.00	0	0	0.00	1.0000
Grade 4	0	0	0.00	0	0	0.00	0	0	0.00	1.0000
Grade 5	0	0	0.00	0	0	0.00	0	0	0.00	1.0000
Grade 2 and above	1	1	0.11	2	2	0.22	3	3	0.17	1.0000
Grade 3 and above	0	0	0.00	0	0	0.00	0	0	0.00	1.0000
Sneezing	0	0	0.00	2	2	0.22	2	2	0.11	0.4997
Grade 1	0	0	0.00	1	1	0.11	1	1	0.06	1.0000
Grade 2	0	0	0.00	1	1	0.11	1	1	0.06	1.0000
Grade 3	0	0	0.00	0	0	0.00	0	0	0.00	1.0000
Grade 4	0	0	0.00	0	0	0.00	0	0	0.00	1.0000
Grade 5	0	0	0.00	0	0	0.00	0	0	0.00	1.0000
Grade 2 and above	0	0	0.00	1	1	0.11	1	1	0.06	1.0000
Grade 3 and above	0	0	0.00	0	0	0.00	0	0	0.00	1.0000
Epistaxis	0	0	0.00	1	1	0.11	1	1	0.06	1.0000
Grade 1	0	0	0.00	1	1	0.11	1	1	0.06	1.0000
Grade 2	0	0	0.00	0	0	0.00	0	0	0.00	1.0000
Grade 3	0	0	0.00	0	0	0.00	0	0	0.00	1.0000
Grade 4	0	0	0.00	0	0	0.00	0	0	0.00	1.0000
Grade 5	0	0	0.00	0	0	0.00	0	0	0.00	1.0000
Grade 2 and above	0	0	0.00	0	0	0.00	0	0	0.00	1.0000
Grade 3 and above	0	0	0.00	0	0	0.00	0	0	0.00	1.0000
Allergic coryza	1	1	0.11	0	0	0.00	1	1	0.06	0.4978
Grade 1	1	1	0.11	0	0	0.00	1	1	0.06	0.4978
Grade 2	0	0	0.00	0	0	0.00	0	0	0.00	1.0000
Grade 3	0	0	0.00	0	0	0.00	0	0	0.00	1.0000
Grade 4	0	0	0.00	0	0	0.00	0	0	0.00	1.0000
Grade 5	0	0	0.00	0	0	0.00	0	0	0.00	1.0000
Grade 2 and above	0	0	0.00	0	0	0.00	0	0	0.00	1.0000
Grade 3 and above	0	0	0.00	0	0	0.00	0	0	0.00	1.0000
Hemoptysis	1	1	0.11	0	0	0.00	1	1	0.06	0.4978
Grade 1	0	0	0.00	0	0	0.00	0	0	0.00	1.0000
Grade 2	0	0	0.00	0	0	0.00	0	0	0.00	1.0000
Grade 3	1	1	0.11	0	0	0.00	1	1	0.06	0.4978
Grade 4	0	0	0.00	0	0	0.00	0	0	0.00	1.0000
Grade 5	0	0	0.00	0	0	0.00	0	0	0.00	1.0000
Grade 2 and above	1	1	0.11	0	0	0.00	1	1	0.06	0.4978
Grade 3 and above	1	1	0.11	0	0	0.00	1	1	0.06	0.4978
**Musculoskeletal and connective tissue disorders**	47	44	4.91	36	33	3.65	83	77	4.28	0.2011
Grade 1	23	23	2.57	21	19	2.10	44	42	2.33	0.5360
Grade 2	20	18	2.01	14	14	1.55	34	32	1.78	0.4812
Grade 3	4	4	0.45	1	1	0.11	5	5	0.28	0.2162
Grade 4	0	0	0.00	0	0	0.00	0	0	0.00	1.0000
Grade 5	0	0	0.00	0	0	0.00	0	0	0.00	1.0000
Grade 2 and above	24	22	2.46	15	15	1.66	39	37	2.06	0.2491
Grade 3 and above	4	4	0.45	1	1	0.11	5	5	0.28	0.2162
Myalgia	45	42	4.69	33	32	3.54	78	74	4.11	0.2365
Grade 1	22	22	2.46	19	19	2.10	41	41	2.28	0.6388
Grade 2	19	17	1.90	13	13	1.44	32	30	1.67	0.4676
Grade 3	4	4	0.45	1	1	0.11	5	5	0.28	0.2162

Adverse Events	Recombinant vaccine group (N=896)			Inactivated vaccine group (N=904)			Total (N=1800)			
Severity	Adverse events	Cases	Incidence (%)	Adverse events	Cases	Incidence (%)	Adverse events	Cases	Incidence (%)	P Value[1]
Grade 4	0	0	0.00	0	0	0.00	0	0	0.00	1.0000
Grade 5	0	0	0.00	0	0	0.00	0	0	0.00	1.0000
Grade 2 and above	23	21	2.34	14	14	1.55	37	35	1.94	0.2365
Grade 3 and above	4	4	0.45	1	1	0.11	5	5	0.28	0.2162
Arthralgia	2	2	0.22	3	3	0.33	5	5	0.28	1.0000
Grade 1	1	1	0.11	2	2	0.22	3	3	0.17	1.0000
Grade 2	1	1	0.11	1	1	0.11	2	2	0.11	1.0000
Grade 3	0	0	0.00	0	0	0.00	0	0	0.00	1.0000
Grade 4	0	0	0.00	0	0	0.00	0	0	0.00	1.0000
Grade 5	0	0	0.00	0	0	0.00	0	0	0.00	1.0000
Grade 2 and above	1	1	0.11	1	1	0.11	2	2	0.11	1.0000
Grade 3 and above	0	0	0.00	0	0	0.00	0	0	0.00	1.0000
**Gastrointestinal disorders**	16	13	1.45	13	10	1.11	29	23	1.28	0.5370
Grade 1	11	10	1.12	9	8	0.88	20	18	1.00	0.6446
Grade 2	4	2	0.22	4	4	0.44	8	6	0.33	0.6870
Grade 3	1	1	0.11	0	0	0.00	1	1	0.06	0.4978
Grade 4	0	0	0.00	0	0	0.00	0	0	0.00	1.0000
Grade 5	0	0	0.00	0	0	0.00	0	0	0.00	1.0000
Grade 2 and above	5	3	0.33	4	4	0.44	9	7	0.39	1.0000
Grade 3 and above	1	1	0.11	0	0	0.00	1	1	0.06	0.4978
Nausea	6	6	0.67	3	3	0.33	9	9	0.50	0.3405
Grade 1	4	4	0.45	2	2	0.22	6	6	0.33	0.4504
Grade 2	1	1	0.11	1	1	0.11	2	2	0.11	1.0000
Grade 3	1	1	0.11	0	0	0.00	1	1	0.06	0.4978
Grade 4	0	0	0.00	0	0	0.00	0	0	0.00	1.0000
Grade 5	0	0	0.00	0	0	0.00	0	0	0.00	1.0000
Grade 2 and above	2	2	0.22	1	1	0.11	3	3	0.17	0.6231
Grade 3 and above	1	1	0.11	0	0	0.00	1	1	0.06	0.4978
Diarrhoea	2	2	0.22	2	2	0.22	4	4	0.22	1.0000
Grade 1	2	2	0.22	2	2	0.22	4	4	0.22	1.0000
Grade 2	0	0	0.00	0	0	0.00	0	0	0.00	1.0000
Grade 3	0	0	0.00	0	0	0.00	0	0	0.00	1.0000
Grade 4	0	0	0.00	0	0	0.00	0	0	0.00	1.0000
Grade 5	0	0	0.00	0	0	0.00	0	0	0.00	1.0000
Grade 2 and above	0	0	0.00	0	0	0.00	0	0	0.00	1.0000
Grade 3 and above	0	0	0.00	0	0	0.00	0	0	0.00	1.0000
Abdominal pain	0	0	0.00	3	3	0.33	3	3	0.17	0.2496
Grade 1	0	0	0.00	2	2	0.22	2	2	0.11	0.4997
Grade 2	0	0	0.00	1	1	0.11	1	1	0.06	1.0000
Grade 3	0	0	0.00	0	0	0.00	0	0	0.00	1.0000
Grade 4	0	0	0.00	0	0	0.00	0	0	0.00	1.0000
Grade 5	0	0	0.00	0	0	0.00	0	0	0.00	1.0000
Grade 2 and above	0	0	0.00	1	1	0.11	1	1	0.06	1.0000
Grade 3 and above	0	0	0.00	0	0	0.00	0	0	0.00	1.0000
Vomiting	2	2	0.22	1	1	0.11	3	3	0.17	0.6231
Grade 1	1	1	0.11	1	1	0.11	2	2	0.11	1.0000
Grade 2	1	1	0.11	0	0	0.00	1	1	0.06	0.4978
Grade 3	0	0	0.00	0	0	0.00	0	0	0.00	1.0000
Grade 4	0	0	0.00	0	0	0.00	0	0	0.00	1.0000
Grade 5	0	0	0.00	0	0	0.00	0	0	0.00	1.0000
Grade 2 and above	1	1	0.11	0	0	0.00	1	1	0.06	0.4978

Adverse Events	Recombinant vaccine group (N=896)			Inactivated vaccine group (N=904)			Total (N=1800)			
Severity	Adverse events	Cases	Incidence (%)	Adverse events	Cases	Incidence (%)	Adverse events	Cases	Incidence (%)	P Value[1]
Grade 3 and above	0	0	0.00	0	0	0.00	0	0	0.00	1.0000
Abdominal pain upper	2	2	0.22	1	1	0.11	3	3	0.17	0.6231
Grade 1	0	0	0.00	0	0	0.00	0	0	0.00	1.0000
Grade 2	2	2	0.22	1	1	0.11	3	3	0.17	0.6231
Grade 3	0	0	0.00	0	0	0.00	0	0	0.00	1.0000
Grade 4	0	0	0.00	0	0	0.00	0	0	0.00	1.0000
Grade 5	0	0	0.00	0	0	0.00	0	0	0.00	1.0000
Grade 2 and above	2	2	0.22	1	1	0.11	3	3	0.17	0.6231
Grade 3 and above	0	0	0.00	0	0	0.00	0	0	0.00	1.0000
Dysphagia	0	0	0.00	2	2	0.22	2	2	0.11	0.4997
Grade 1	0	0	0.00	2	2	0.22	2	2	0.11	0.4997
Grade 2	0	0	0.00	0	0	0.00	0	0	0.00	1.0000
Grade 3	0	0	0.00	0	0	0.00	0	0	0.00	1.0000
Grade 4	0	0	0.00	0	0	0.00	0	0	0.00	1.0000
Grade 5	0	0	0.00	0	0	0.00	0	0	0.00	1.0000
Grade 2 and above	0	0	0.00	0	0	0.00	0	0	0.00	1.0000
Grade 3 and above	0	0	0.00	0	0	0.00	0	0	0.00	1.0000
Abdominal distension	1	1	0.11	0	0	0.00	1	1	0.06	0.4978
Grade 1	1	1	0.11	0	0	0.00	1	1	0.06	0.4978
Grade 2	0	0	0.00	0	0	0.00	0	0	0.00	1.0000
Grade 3	0	0	0.00	0	0	0.00	0	0	0.00	1.0000
Grade 4	0	0	0.00	0	0	0.00	0	0	0.00	1.0000
Grade 5	0	0	0.00	0	0	0.00	0	0	0.00	1.0000
Grade 2 and above	0	0	0.00	0	0	0.00	0	0	0.00	1.0000
Grade 3 and above	0	0	0.00	0	0	0.00	0	0	0.00	1.0000
Dry mouth	1	1	0.11	0	0	0.00	1	1	0.06	0.4978
Grade 1	1	1	0.11	0	0	0.00	1	1	0.06	0.4978
Grade 2	0	0	0.00	0	0	0.00	0	0	0.00	1.0000
Grade 3	0	0	0.00	0	0	0.00	0	0	0.00	1.0000
Grade 4	0	0	0.00	0	0	0.00	0	0	0.00	1.0000
Grade 5	0	0	0.00	0	0	0.00	0	0	0.00	1.0000
Grade 2 and above	0	0	0.00	0	0	0.00	0	0	0.00	1.0000
Grade 3 and above	0	0	0.00	0	0	0.00	0	0	0.00	1.0000
Oral pain	1	1	0.11	0	0	0.00	1	1	0.06	0.4978
Grade 1	1	1	0.11	0	0	0.00	1	1	0.06	0.4978
Grade 2	0	0	0.00	0	0	0.00	0	0	0.00	1.0000
Grade 3	0	0	0.00	0	0	0.00	0	0	0.00	1.0000
Grade 4	0	0	0.00	0	0	0.00	0	0	0.00	1.0000
Grade 5	0	0	0.00	0	0	0.00	0	0	0.00	1.0000
Grade 2 and above	0	0	0.00	0	0	0.00	0	0	0.00	1.0000
Grade 3 and above	0	0	0.00	0	0	0.00	0	0	0.00	1.0000
Poor digestion	1	1	0.11	0	0	0.00	1	1	0.06	0.4978
Grade 1	1	1	0.11	0	0	0.00	1	1	0.06	0.4978
Grade 2	0	0	0.00	0	0	0.00	0	0	0.00	1.0000
Grade 3	0	0	0.00	0	0	0.00	0	0	0.00	1.0000
Grade 4	0	0	0.00	0	0	0.00	0	0	0.00	1.0000
Grade 5	0	0	0.00	0	0	0.00	0	0	0.00	1.0000
Grade 2 and above	0	0	0.00	0	0	0.00	0	0	0.00	1.0000
Grade 3 and above	0	0	0.00	0	0	0.00	0	0	0.00	1.0000

Adverse Events<br>Severity	Recombinant vaccine group (N=896)			Inactivated vaccine group (N=904)			Total (N=1800)			
	Adverse events	Cases	Incidence (%)	Adverse events	Cases	Incidence (%)	Adverse events	Cases	Incidence (%)	P Value[1]
Toothache	0	0	0.00	1	1	0.11	1	1	0.06	1.0000
Grade 1	0	0	0.00	0	0	0.00	0	0	0.00	1.0000
Grade 2	0	0	0.00	1	1	0.11	1	1	0.06	1.0000
Grade 3	0	0	0.00	0	0	0.00	0	0	0.00	1.0000
Grade 4	0	0	0.00	0	0	0.00	0	0	0.00	1.0000
Grade 5	0	0	0.00	0	0	0.00	0	0	0.00	1.0000
Grade 2 and above	0	0	0.00	1	1	0.11	1	1	0.06	1.0000
Grade 3 and above	0	0	0.00	0	0	0.00	0	0	0.00	1.0000
**Infections and infestations**	7	7	0.78	5	4	0.44	12	11	0.61	0.3842
Grade 1	7	7	0.78	4	4	0.44	11	11	0.61	0.3842
Grade 2	0	0	0.00	1	1	0.11	1	1	0.06	1.0000
Grade 3	0	0	0.00	0	0	0.00	0	0	0.00	1.0000
Grade 4	0	0	0.00	0	0	0.00	0	0	0.00	1.0000
Grade 5	0	0	0.00	0	0	0.00	0	0	0.00	1.0000
Grade 2 and above	0	0	0.00	1	1	0.11	1	1	0.06	1.0000
Grade 3 and above	0	0	0.00	0	0	0.00	0	0	0.00	1.0000
Nasopharyngitis	7	7	0.78	3	3	0.33	10	10	0.56	0.2233
Grade 1	7	7	0.78	3	3	0.33	10	10	0.56	0.2233
Grade 2	0	0	0.00	0	0	0.00	0	0	0.00	1.0000
Grade 3	0	0	0.00	0	0	0.00	0	0	0.00	1.0000
Grade 4	0	0	0.00	0	0	0.00	0	0	0.00	1.0000
Grade 5	0	0	0.00	0	0	0.00	0	0	0.00	1.0000
Grade 2 and above	0	0	0.00	0	0	0.00	0	0	0.00	1.0000
Grade 3 and above	0	0	0.00	0	0	0.00	0	0	0.00	1.0000
Influenza	0	0	0.00	2	1	0.11	2	1	0.06	1.0000
Grade 1	0	0	0.00	1	1	0.11	1	1	0.06	1.0000
Grade 2	0	0	0.00	1	1	0.11	1	1	0.06	1.0000
Grade 3	0	0	0.00	0	0	0.00	0	0	0.00	1.0000
Grade 4	0	0	0.00	0	0	0.00	0	0	0.00	1.0000
Grade 5	0	0	0.00	0	0	0.00	0	0	0.00	1.0000
Grade 2 and above	0	0	0.00	1	1	0.11	1	1	0.06	1.0000
Grade 3 and above	0	0	0.00	0	0	0.00	0	0	0.00	1.0000
**Skin and subcutaneous tissue disorders**	3	3	0.33	5	4	0.44	8	7	0.39	1.0000
Grade 1	3	3	0.33	1	1	0.11	4	4	0.22	0.3724
Grade 2	0	0	0.00	4	4	0.44	4	4	0.22	0.1246
Grade 3	0	0	0.00	0	0	0.00	0	0	0.00	1.0000
Grade 4	0	0	0.00	0	0	0.00	0	0	0.00	1.0000
Grade 5	0	0	0.00	0	0	0.00	0	0	0.00	1.0000
Grade 2 and above	0	0	0.00	4	4	0.44	4	4	0.22	0.1246
Grade 3 and above	0	0	0.00	0	0	0.00	0	0	0.00	1.0000
Pruritus	2	2	0.22	4	4	0.44	6	6	0.33	0.6870
Grade 1	2	2	0.22	0	0	0.00	2	2	0.11	0.2476
Grade 2	0	0	0.00	4	4	0.44	4	4	0.22	0.1246
Grade 3	0	0	0.00	0	0	0.00	0	0	0.00	1.0000
Grade 4	0	0	0.00	0	0	0.00	0	0	0.00	1.0000
Grade 5	0	0	0.00	0	0	0.00	0	0	0.00	1.0000
Grade 2 and above	0	0	0.00	4	4	0.44	4	4	0.22	0.1246
Grade 3 and above	0	0	0.00	0	0	0.00	0	0	0.00	1.0000

Adverse Events	Recombinant vaccine group (N=896)			Inactivated vaccine group (N=904)			Total (N=1800)			
Severity	Adverse events	Cases	Incidence (%)	Adverse events	Cases	Incidence (%)	Adverse events	Cases	Incidence (%)	P Value[1]
Erythema	1	1	0.11	0	0	0.00	1	1	0.06	0.4978
Grade 1	1	1	0.11	0	0	0.00	1	1	0.06	0.4978
Grade 2	0	0	0.00	0	0	0.00	0	0	0.00	1.0000
Grade 3	0	0	0.00	0	0	0.00	0	0	0.00	1.0000
Grade 4	0	0	0.00	0	0	0.00	0	0	0.00	1.0000
Grade 5	0	0	0.00	0	0	0.00	0	0	0.00	1.0000
Grade 2 and above	0	0	0.00	0	0	0.00	0	0	0.00	1.0000
Grade 3 and above	0	0	0.00	0	0	0.00	0	0	0.00	1.0000
Rash	0	0	0.00	1	1	0.11	1	1	0.06	1.0000
Grade 1	0	0	0.00	1	1	0.11	1	1	0.06	1.0000
Grade 2	0	0	0.00	0	0	0.00	0	0	0.00	1.0000
Grade 3	0	0	0.00	0	0	0.00	0	0	0.00	1.0000
Grade 4	0	0	0.00	0	0	0.00	0	0	0.00	1.0000
Grade 5	0	0	0.00	0	0	0.00	0	0	0.00	1.0000
Grade 2 and above	0	0	0.00	0	0	0.00	0	0	0.00	1.0000
Grade 3 and above	0	0	0.00	0	0	0.00	0	0	0.00	1.0000
**Psychiatric disorders**	3	3	0.33	1	1	0.11	4	4	0.22	0.3724
Grade 1	2	2	0.22	1	1	0.11	3	3	0.17	0.6231
Grade 2	1	1	0.11	0	0	0.00	1	1	0.06	0.4978
Grade 3	0	0	0.00	0	0	0.00	0	0	0.00	1.0000
Grade 4	0	0	0.00	0	0	0.00	0	0	0.00	1.0000
Grade 5	0	0	0.00	0	0	0.00	0	0	0.00	1.0000
Grade 2 and above	1	1	0.11	0	0	0.00	1	1	0.06	0.4978
Grade 3 and above	0	0	0.00	0	0	0.00	0	0	0.00	1.0000
Insomnia	2	2	0.22	1	1	0.11	3	3	0.17	0.6231
Grade 1	2	2	0.22	1	1	0.11	3	3	0.17	0.6231
Grade 2	0	0	0.00	0	0	0.00	0	0	0.00	1.0000
Grade 3	0	0	0.00	0	0	0.00	0	0	0.00	1.0000
Grade 4	0	0	0.00	0	0	0.00	0	0	0.00	1.0000
Grade 5	0	0	0.00	0	0	0.00	0	0	0.00	1.0000
Grade 2 and above	0	0	0.00	0	0	0.00	0	0	0.00	1.0000
Grade 3 and above	0	0	0.00	0	0	0.00	0	0	0.00	1.0000
Anxiety	1	1	0.11	0	0	0.00	1	1	0.06	0.4978
Grade 1	0	0	0.00	0	0	0.00	0	0	0.00	1.0000
Grade 2	1	1	0.11	0	0	0.00	1	1	0.06	0.4978
Grade 3	0	0	0.00	0	0	0.00	0	0	0.00	1.0000
Grade 4	0	0	0.00	0	0	0.00	0	0	0.00	1.0000
Grade 5	0	0	0.00	0	0	0.00	0	0	0.00	1.0000
Grade 2 and above	1	1	0.11	0	0	0.00	1	1	0.06	0.4978
Grade 3 and above	0	0	0.00	0	0	0.00	0	0	0.00	1.0000
**Immune system disease**	3	3	0.33	1	1	0.11	4	4	0.22	0.3724
Grade 1	3	3	0.33	1	1	0.11	4	4	0.22	0.3724
Grade 2	0	0	0.00	0	0	0.00	0	0	0.00	1.0000
Grade 3	0	0	0.00	0	0	0.00	0	0	0.00	1.0000
Grade 4	0	0	0.00	0	0	0.00	0	0	0.00	1.0000
Grade 5	0	0	0.00	0	0	0.00	0	0	0.00	1.0000
Grade 2 and above	0	0	0.00	0	0	0.00	0	0	0.00	1.0000
Grade 3 and above	0	0	0.00	0	0	0.00	0	0	0.00	1.0000

Adverse Events	Recombinant vaccine group (N=896)			Inactivated vaccine group (N=904)			Total (N=1800)			
Severity	Adverse events	Cases	Incidence (%)	Adverse events	Cases	Incidence (%)	Adverse events	Cases	Incidence (%)	P Value[1]
Hypersensitivity reaction	3	3	0.33	1	1	0.11	4	4	0.22	0.3724
Grade 1	3	3	0.33	1	1	0.11	4	4	0.22	0.3724
Grade 2	0	0	0.00	0	0	0.00	0	0	0.00	1.0000
Grade 3	0	0	0.00	0	0	0.00	0	0	0.00	1.0000
Grade 4	0	0	0.00	0	0	0.00	0	0	0.00	1.0000
Grade 5	0	0	0.00	0	0	0.00	0	0	0.00	1.0000
Grade 2 and above	0	0	0.00	0	0	0.00	0	0	0.00	1.0000
Grade 3 and above	0	0	0.00	0	0	0.00	0	0	0.00	1.0000
**Renal and urinary disorders**	4	3	0.33	1	1	0.11	5	4	0.22	0.3724
Grade 1	3	2	0.22	0	0	0.00	3	2	0.11	0.2476
Grade 2	0	0	0.00	1	1	0.11	1	1	0.06	1.0000
Grade 3	1	1	0.11	0	0	0.00	1	1	0.06	0.4978
Grade 4	0	0	0.00	0	0	0.00	0	0	0.00	1.0000
Grade 5	0	0	0.00	0	0	0.00	0	0	0.00	1.0000
Grade 2 and above	1	1	0.11	1	1	0.11	2	2	0.11	1.0000
Grade 3 and above	1	1	0.11	0	0	0.00	1	1	0.06	0.4978
Urinary frequency	1	1	0.11	1	1	0.11	2	2	0.11	1.0000
Grade 1	1	1	0.11	0	0	0.00	1	1	0.06	0.4978
Grade 2	0	0	0.00	1	1	0.11	1	1	0.06	1.0000
Grade 3	0	0	0.00	0	0	0.00	0	0	0.00	1.0000
Grade 4	0	0	0.00	0	0	0.00	0	0	0.00	1.0000
Grade 5	0	0	0.00	0	0	0.00	0	0	0.00	1.0000
Grade 2 and above	0	0	0.00	1	1	0.11	1	1	0.06	1.0000
Grade 3 and above	0	0	0.00	0	0	0.00	0	0	0.00	1.0000
Polyuria	1	1	0.11	0	0	0.00	1	1	0.06	0.4978
Grade 1	1	1	0.11	0	0	0.00	1	1	0.06	0.4978
Grade 2	0	0	0.00	0	0	0.00	0	0	0.00	1.0000
Grade 3	0	0	0.00	0	0	0.00	0	0	0.00	1.0000
Grade 4	0	0	0.00	0	0	0.00	0	0	0.00	1.0000
Grade 5	0	0	0.00	0	0	0.00	0	0	0.00	1.0000
Grade 2 and above	0	0	0.00	0	0	0.00	0	0	0.00	1.0000
Grade 3 and above	0	0	0.00	0	0	0.00	0	0	0.00	1.0000
Dysuria	1	1	0.11	0	0	0.00	1	1	0.06	0.4978
Grade 1	1	1	0.11	0	0	0.00	1	1	0.06	0.4978
Grade 2	0	0	0.00	0	0	0.00	0	0	0.00	1.0000
Grade 3	0	0	0.00	0	0	0.00	0	0	0.00	1.0000
Grade 4	0	0	0.00	0	0	0.00	0	0	0.00	1.0000
Grade 5	0	0	0.00	0	0	0.00	0	0	0.00	1.0000
Grade 2 and above	0	0	0.00	0	0	0.00	0	0	0.00	1.0000
Grade 3 and above	0	0	0.00	0	0	0.00	0	0	0.00	1.0000
Dysuria Hematuria	1	1	0.11	0	0	0.00	1	1	0.06	0.4978
Grade 1	0	0	0.00	0	0	0.00	0	0	0.00	1.0000
Grade 2	0	0	0.00	0	0	0.00	0	0	0.00	1.0000
Grade 3	1	1	0.11	0	0	0.00	1	1	0.06	0.4978
Grade 4	0	0	0.00	0	0	0.00	0	0	0.00	1.0000
Grade 5	0	0	0.00	0	0	0.00	0	0	0.00	1.0000
Grade 2 and above	1	1	0.11	0	0	0.00	1	1	0.06	0.4978
Grade 3 and above	1	1	0.11	0	0	0.00	1	1	0.06	0.4978
**Various inspections**	1	1	0.11	2	2	0.22	3	3	0.17	1.0000

Adverse Events	Recombinant vaccine group (N=896)			Inactivated vaccine group (N=904)			Total (N=1800)			
Severity	Adverse events	Cases	Incidence (%)	Adverse events	Cases	Incidence (%)	Adverse events	Cases	Incidence (%)	P Value[1]
Grade 1	1	1	0.11	2	2	0.22	3	3	0.17	1.0000
Grade 2	0	0	0.00	0	0	0.00	0	0	0.00	1.0000
Grade 3	0	0	0.00	0	0	0.00	0	0	0.00	1.0000
Grade 4	0	0	0.00	0	0	0.00	0	0	0.00	1.0000
Grade 5	0	0	0.00	0	0	0.00	0	0	0.00	1.0000
Grade 2 and above	0	0	0.00	0	0	0.00	0	0	0.00	1.0000
Grade 3 and above	0	0	0.00	0	0	0.00	0	0	0.00	1.0000
Blood pressure increased	1	1	0.11	1	1	0.11	2	2	0.11	1.0000
Grade 1	1	1	0.11	1	1	0.11	2	2	0.11	1.0000
Grade 2	0	0	0.00	0	0	0.00	0	0	0.00	1.0000
Grade 3	0	0	0.00	0	0	0.00	0	0	0.00	1.0000
Grade 4	0	0	0.00	0	0	0.00	0	0	0.00	1.0000
Grade 5	0	0	0.00	0	0	0.00	0	0	0.00	1.0000
Grade 2 and above	0	0	0.00	0	0	0.00	0	0	0.00	1.0000
Grade 3 and above	0	0	0.00	0	0	0.00	0	0	0.00	1.0000
Heart rate increased	0	0	0.00	1	1	0.11	1	1	0.06	1.0000
Grade 1	0	0	0.00	1	1	0.11	1	1	0.06	1.0000
Grade 2	0	0	0.00	0	0	0.00	0	0	0.00	1.0000
Grade 3	0	0	0.00	0	0	0.00	0	0	0.00	1.0000
Grade 4	0	0	0.00	0	0	0.00	0	0	0.00	1.0000
Grade 5	0	0	0.00	0	0	0.00	0	0	0.00	1.0000
Grade 2 and above	0	0	0.00	0	0	0.00	0	0	0.00	1.0000
Grade 3 and above	0	0	0.00	0	0	0.00	0	0	0.00	1.0000
**Eye disorders**	3	3	0.33	0	0	0.00	3	3	0.17	0.1231
Grade 1	1	1	0.11	0	0	0.00	1	1	0.06	0.4978
Grade 2	2	2	0.22	0	0	0.00	2	2	0.11	0.2476
Grade 3	0	0	0.00	0	0	0.00	0	0	0.00	1.0000
Grade 4	0	0	0.00	0	0	0.00	0	0	0.00	1.0000
Grade 5	0	0	0.00	0	0	0.00	0	0	0.00	1.0000
Grade 2 and above	2	2	0.22	0	0	0.00	2	2	0.11	0.2476
Grade 3 and above	0	0	0.00	0	0	0.00	0	0	0.00	1.0000
Ocular hyperemia	1	1	0.11	0	0	0.00	1	1	0.06	0.4978
Grade 1	1	1	0.11	0	0	0.00	1	1	0.06	0.4978
Grade 2	0	0	0.00	0	0	0.00	0	0	0.00	1.0000
Grade 3	0	0	0.00	0	0	0.00	0	0	0.00	1.0000
Grade 4	0	0	0.00	0	0	0.00	0	0	0.00	1.0000
Grade 5	0	0	0.00	0	0	0.00	0	0	0.00	1.0000
Grade 2 and above	0	0	0.00	0	0	0.00	0	0	0.00	1.0000
Grade 3 and above	0	0	0.00	0	0	0.00	0	0	0.00	1.0000
Eye irritation	1	1	0.11	0	0	0.00	1	1	0.06	0.4978
Grade 1	0	0	0.00	0	0	0.00	0	0	0.00	1.0000
Grade 2	1	1	0.11	0	0	0.00	1	1	0.06	0.4978
Grade 3	0	0	0.00	0	0	0.00	0	0	0.00	1.0000
Grade 4	0	0	0.00	0	0	0.00	0	0	0.00	1.0000
Grade 5	0	0	0.00	0	0	0.00	0	0	0.00	1.0000
Grade 2 and above	1	1	0.11	0	0	0.00	1	1	0.06	0.4978
Grade 3 and above	0	0	0.00	0	0	0.00	0	0	0.00	1.0000
Eye pruritus	1	1	0.11	0	0	0.00	1	1	0.06	0.4978
Grade 1	0	0	0.00	0	0	0.00	0	0	0.00	1.0000
Grade 2	1	1	0.11	0	0	0.00	1	1	0.06	0.4978

Adverse Events	Recombinant vaccine group (N=896)			Inactivated vaccine group (N=904)			Total (N=1800)			
Severity	Adverse events	Cases	Incidence (%)	Adverse events	Cases	Incidence (%)	Adverse events	Cases	Incidence (%)	P Value[1]
Grade 3	0	0	0.00	0	0	0.00	0	0	0.00	1.0000
Grade 4	0	0	0.00	0	0	0.00	0	0	0.00	1.0000
Grade 5	0	0	0.00	0	0	0.00	0	0	0.00	1.0000
Grade 2 and above	1	1	0.11	0	0	0.00	1	1	0.06	0.4978
Grade 3 and above	0	0	0.00	0	0	0.00	0	0	0.00	1.0000
**Ear and labyrinth disorders**	1	1	0.11	0	0	0.00	1	1	0.06	0.4978
Grade 1	0	0	0.00	0	0	0.00	0	0	0.00	1.0000
Grade 2	1	1	0.11	0	0	0.00	1	1	0.06	0.4978
Grade 3	0	0	0.00	0	0	0.00	0	0	0.00	1.0000
Grade 4	0	0	0.00	0	0	0.00	0	0	0.00	1.0000
Grade 5	0	0	0.00	0	0	0.00	0	0	0.00	1.0000
Grade 2 and above	1	1	0.11	0	0	0.00	1	1	0.06	0.4978
Grade 3 and above	0	0	0.00	0	0	0.00	0	0	0.00	1.0000
Ear pain	1	1	0.11	0	0	0.00	1	1	0.06	0.4978
Grade 1	0	0	0.00	0	0	0.00	0	0	0.00	1.0000
Grade 2	1	1	0.11	0	0	0.00	1	1	0.06	0.4978
Grade 3	0	0	0.00	0	0	0.00	0	0	0.00	1.0000
Grade 4	0	0	0.00	0	0	0.00	0	0	0.00	1.0000
Grade 5	0	0	0.00	0	0	0.00	0	0	0.00	1.0000
Grade 2 and above	1	1	0.11	0	0	0.00	1	1	0.06	0.4978
Grade 3 and above	0	0	0.00	0	0	0.00	0	0	0.00	1.0000
**Reproductive system and breast disorders**	1	1	0.11	0	0	0.00	1	1	0.06	0.4978
Grade 1	1	1	0.11	0	0	0.00	1	1	0.06	0.4978
Grade 2	0	0	0.00	0	0	0.00	0	0	0.00	1.0000
Grade 3	0	0	0.00	0	0	0.00	0	0	0.00	1.0000
Grade 4	0	0	0.00	0	0	0.00	0	0	0.00	1.0000
Grade 5	0	0	0.00	0	0	0.00	0	0	0.00	1.0000
Grade 2 and above	0	0	0.00	0	0	0.00	0	0	0.00	1.0000
Grade 3 and above	0	0	0.00	0	0	0.00	0	0	0.00	1.0000
Erectile dysfunctiom	1	1	0.11	0	0	0.00	1	1	0.06	0.4978
Grade 1	1	1	0.11	0	0	0.00	1	1	0.06	0.4978
Grade 2	0	0	0.00	0	0	0.00	0	0	0.00	1.0000
Grade 3	0	0	0.00	0	0	0.00	0	0	0.00	1.0000
Grade 4	0	0	0.00	0	0	0.00	0	0	0.00	1.0000
Grade 5	0	0	0.00	0	0	0.00	0	0	0.00	1.0000
Grade 2 and above	0	0	0.00	0	0	0.00	0	0	0.00	1.0000
Grade 3 and above	0	0	0.00	0	0	0.00	0	0	0.00	1.0000

[1]P value was calculated by Fisher exact test.

(1) Adverse events were coded using MedDRA version 24.1.

(2) This analysis data is up to January 25, 2022.

Table 1-3 Incidence and severity of various adverse events related to the study vaccine in the whole population during the booster dose immunization period (listed by SOC and PT) (SS)

Adverse Events / Severity	Recombinant vaccine group (N=896)			Inactivated vaccine group (N=904)			Total (N=1800)			
	Adverse events	Cases	Incidence (%)	Adverse events	Cases	Incidence (%)	Adverse events	Cases	Incidence (%)	P Value[1]
**TEAE**	289	157	17.52	250	135	14.93	539	292	16.22	0.1417
Grade 1	200	135	15.07	167	109	12.06	367	244	13.56	0.0634
Grade 2	83	50	5.58	79	49	5.42	162	99	5.50	0.9179
Grade 3	6	5	0.56	4	2	0.22	10	7	0.39	0.2861
Grade 4	0	0	0.00	0	0	0.00	0	0	0.00	1.0000
Grade 5	0	0	0.00	0	0	0.00	0	0	0.00	1.0000
Grade 2 and above	89	54	6.03	83	49	5.42	172	103	5.72	0.6126
Grade 3 and above	6	5	0.56	4	2	0.22	10	7	0.39	0.2861
**General disorders and administration site conditions**	112	91	10.16	100	79	8.74	212	170	9.44	0.3336
Grade 1	88	78	8.71	82	70	7.74	170	148	8.22	0.4927
Grade 2	21	19	2.12	15	14	1.55	36	33	1.83	0.3852
Grade 3	3	3	0.33	3	2	0.22	6	5	0.28	0.6858
Grade 4	0	0	0.00	0	0	0.00	0	0	0.00	1.0000
Grade 5	0	0	0.00	0	0	0.00	0	0	0.00	1.0000
Grade 2 and above	24	22	2.46	18	15	1.66	42	37	2.06	0.2491
Grade 3 and above	3	3	0.33	3	2	0.22	6	5	0.28	0.6858
Pain at vaccination site	42	42	4.69	36	36	3.98	78	78	4.33	0.4888
Grade 1	36	36	4.02	32	32	3.54	68	68	3.78	0.6225
Grade 2	6	6	0.67	4	4	0.44	10	10	0.56	0.5460
Grade 3	0	0	0.00	0	0	0.00	0	0	0.00	1.0000
Grade 4	0	0	0.00	0	0	0.00	0	0	0.00	1.0000
Grade 5	0	0	0.00	0	0	0.00	0	0	0.00	1.0000
Grade 2 and above	6	6	0.67	4	4	0.44	10	10	0.56	0.5460
Grade 3 and above	0	0	0.00	0	0	0.00	0	0	0.00	1.0000
Fatigue	29	28	3.13	26	26	2.88	55	54	3.00	0.7837
Grade 1	20	20	2.23	17	17	1.88	37	37	2.06	0.6221
Grade 2	9	9	1.00	9	9	1.00	18	18	1.00	1.0000
Grade 3	0	0	0.00	0	0	0.00	0	0	0.00	1.0000
Grade 4	0	0	0.00	0	0	0.00	0	0	0.00	1.0000
Grade 5	0	0	0.00	0	0	0.00	0	0	0.00	1.0000
Grade 2 and above	9	9	1.00	9	9	1.00	18	18	1.00	1.0000
Grade 3 and above	0	0	0.00	0	0	0.00	0	0	0.00	1.0000
Fever	28	27	3.01	27	26	2.88	55	53	2.94	0.8899
Grade 1	21	21	2.34	23	22	2.43	44	43	2.39	1.0000
Grade 2	4	4	0.45	2	2	0.22	6	6	0.33	0.4504
Grade 3	3	3	0.33	2	2	0.22	5	5	0.28	0.6858
Grade 4	0	0	0.00	0	0	0.00	0	0	0.00	1.0000
Grade 5	0	0	0.00	0	0	0.00	0	0	0.00	1.0000
Grade 2 and above	7	7	0.78	4	4	0.44	11	11	0.61	0.3842
Grade 3 and above	3	3	0.33	2	2	0.22	5	5	0.28	0.6858
Chest pain	2	2	0.22	4	4	0.44	6	6	0.33	0.6870
Grade 1	1	1	0.11	3	3	0.33	4	4	0.22	0.6246
Grade 2	1	1	0.11	0	0	0.00	1	1	0.06	0.4978
Grade 3	0	0	0.00	1	1	0.11	1	1	0.06	1.0000
Grade 4	0	0	0.00	0	0	0.00	0	0	0.00	1.0000
Grade 5	0	0	0.00	0	0	0.00	0	0	0.00	1.0000
Grade 2 and above	1	1	0.11	1	1	0.11	2	2	0.11	1.0000
Grade 3 and above	0	0	0.00	1	1	0.11	1	1	0.06	1.0000

Adverse Events	Recombinant vaccine group (N=896)			Inactivated vaccine group (N=904)			Total (N=1800)			
Severity	Adverse events	Cases	Incidence (%)	Adverse events	Cases	Incidence (%)	Adverse events	Cases	Incidence (%)	P Value[1]
Feeling hot	4	4	0.45	0	0	0.00	4	4	0.22	0.0612
Grade 1	3	3	0.33	0	0	0.00	3	3	0.17	0.1231
Grade 2	1	1	0.11	0	0	0.00	1	1	0.06	0.4978
Grade 3	0	0	0.00	0	0	0.00	0	0	0.00	1.0000
Grade 4	0	0	0.00	0	0	0.00	0	0	0.00	1.0000
Grade 5	0	0	0.00	0	0	0.00	0	0	0.00	1.0000
Grade 2 and above	1	1	0.11	0	0	0.00	1	1	0.06	0.4978
Grade 3 and above	0	0	0.00	0	0	0.00	0	0	0.00	1.0000
Pain	3	3	0.33	1	1	0.11	4	4	0.22	0.3724
Grade 1	3	3	0.33	1	1	0.11	4	4	0.22	0.3724
Grade 2	0	0	0.00	0	0	0.00	0	0	0.00	1.0000
Grade 3	0	0	0.00	0	0	0.00	0	0	0.00	1.0000
Grade 4	0	0	0.00	0	0	0.00	0	0	0.00	1.0000
Grade 5	0	0	0.00	0	0	0.00	0	0	0.00	1.0000
Grade 2 and above	0	0	0.00	0	0	0.00	0	0	0.00	1.0000
Grade 3 and above	0	0	0.00	0	0	0.00	0	0	0.00	1.0000
Vaccination site pruritus	1	1	0.11	2	2	0.22	3	3	0.17	1.0000
Grade 1	1	1	0.11	2	2	0.22	3	3	0.17	1.0000
Grade 2	0	0	0.00	0	0	0.00	0	0	0.00	1.0000
Grade 3	0	0	0.00	0	0	0.00	0	0	0.00	1.0000
Grade 4	0	0	0.00	0	0	0.00	0	0	0.00	1.0000
Grade 5	0	0	0.00	0	0	0.00	0	0	0.00	1.0000
Grade 2 and above	0	0	0.00	0	0	0.00	0	0	0.00	1.0000
Grade 3 and above	0	0	0.00	0	0	0.00	0	0	0.00	1.0000
Vaccination site erythema	2	2	0.22	0	0	0.00	2	2	0.11	0.2476
Grade 1	2	2	0.22	0	0	0.00	2	2	0.11	0.2476
Grade 2	0	0	0.00	0	0	0.00	0	0	0.00	1.0000
Grade 3	0	0	0.00	0	0	0.00	0	0	0.00	1.0000
Grade 4	0	0	0.00	0	0	0.00	0	0	0.00	1.0000
Grade 5	0	0	0.00	0	0	0.00	0	0	0.00	1.0000
Grade 2 and above	0	0	0.00	0	0	0.00	0	0	0.00	1.0000
Grade 3 and above	0	0	0.00	0	0	0.00	0	0	0.00	1.0000
Swelling	1	1	0.11	1	1	0.11	2	2	0.11	1.0000
Grade 1	1	1	0.11	1	1	0.11	2	2	0.11	1.0000
Grade 2	0	0	0.00	0	0	0.00	0	0	0.00	1.0000
Grade 3	0	0	0.00	0	0	0.00	0	0	0.00	1.0000
Grade 4	0	0	0.00	0	0	0.00	0	0	0.00	1.0000
Grade 5	0	0	0.00	0	0	0.00	0	0	0.00	1.0000
Grade 2 and above	0	0	0.00	0	0	0.00	0	0	0.00	1.0000
Grade 3 and above	0	0	0.00	0	0	0.00	0	0	0.00	1.0000
Influenza-like illness	0	0	0.00	1	1	0.11	1	1	0.06	1.0000
Grade 1	0	0	0.00	1	1	0.11	1	1	0.06	1.0000
Grade 2	0	0	0.00	0	0	0.00	0	0	0.00	1.0000
Grade 3	0	0	0.00	0	0	0.00	0	0	0.00	1.0000
Grade 4	0	0	0.00	0	0	0.00	0	0	0.00	1.0000
Grade 5	0	0	0.00	0	0	0.00	0	0	0.00	1.0000
Grade 2 and above	0	0	0.00	0	0	0.00	0	0	0.00	1.0000
Grade 3 and above	0	0	0.00	0	0	0.00	0	0	0.00	1.0000

Adverse Events	Recombinant vaccine group (N=896)			Inactivated vaccine group (N=904)			Total (N=1800)			
Severity	Adverse events	Cases	Incidence (%)	Adverse events	Cases	Incidence (%)	Adverse events	Cases	Incidence (%)	P Value[1]
Vaccination site rash	0	0	0.00	1	1	0.11	1	1	0.06	1.0000
Grade 1	0	0	0.00	1	1	0.11	1	1	0.06	1.0000
Grade 2	0	0	0.00	0	0	0.00	0	0	0.00	1.0000
Grade 3	0	0	0.00	0	0	0.00	0	0	0.00	1.0000
Grade 4	0	0	0.00	0	0	0.00	0	0	0.00	1.0000
Grade 5	0	0	0.00	0	0	0.00	0	0	0.00	1.0000
Grade 2 and above	0	0	0.00	0	0	0.00	0	0	0.00	1.0000
Grade 3 and above	0	0	0.00	0	0	0.00	0	0	0.00	1.0000
Vaccination site swelling	0	0	0.00	1	1	0.11	1	1	0.06	1.0000
Grade 1	0	0	0.00	1	1	0.11	1	1	0.06	1.0000
Grade 2	0	0	0.00	0	0	0.00	0	0	0.00	1.0000
Grade 3	0	0	0.00	0	0	0.00	0	0	0.00	1.0000
Grade 4	0	0	0.00	0	0	0.00	0	0	0.00	1.0000
Grade 5	0	0	0.00	0	0	0.00	0	0	0.00	1.0000
Grade 2 and above	0	0	0.00	0	0	0.00	0	0	0.00	1.0000
Grade 3 and above	0	0	0.00	0	0	0.00	0	0	0.00	1.0000
**Nervous system disorders**	60	55	6.14	47	43	4.76	107	98	5.44	0.2131
Grade 1	36	34	3.79	23	22	2.43	59	56	3.11	0.1043
Grade 2	24	24	2.68	24	22	2.43	48	46	2.56	0.7671
Grade 3	0	0	0.00	0	0	0.00	0	0	0.00	1.0000
Grade 4	0	0	0.00	0	0	0.00	0	0	0.00	1.0000
Grade 5	0	0	0.00	0	0	0.00	0	0	0.00	1.0000
Grade 2 and above	24	24	2.68	24	22	2.43	48	46	2.56	0.7671
Grade 3 and above	0	0	0.00	0	0	0.00	0	0	0.00	1.0000
Headache	54	52	5.80	41	40	4.42	95	92	5.11	0.1997
Grade 1	31	30	3.35	18	18	1.99	49	48	2.67	0.0801
Grade 2	23	23	2.57	23	22	2.43	46	45	2.50	0.8811
Grade 3	0	0	0.00	0	0	0.00	0	0	0.00	1.0000
Grade 4	0	0	0.00	0	0	0.00	0	0	0.00	1.0000
Grade 5	0	0	0.00	0	0	0.00	0	0	0.00	1.0000
Grade 2 and above	23	23	2.57	23	22	2.43	46	45	2.50	0.8811
Grade 3 and above	0	0	0.00	0	0	0.00	0	0	0.00	1.0000
Dizziness	3	3	0.33	3	3	0.33	6	6	0.33	1.0000
Grade 1	2	2	0.22	2	2	0.22	4	4	0.22	1.0000
Grade 2	1	1	0.11	1	1	0.11	2	2	0.11	1.0000
Grade 3	0	0	0.00	0	0	0.00	0	0	0.00	1.0000
Grade 4	0	0	0.00	0	0	0.00	0	0	0.00	1.0000
Grade 5	0	0	0.00	0	0	0.00	0	0	0.00	1.0000
Grade 2 and above	1	1	0.11	1	1	0.11	2	2	0.11	1.0000
Grade 3 and above	0	0	0.00	0	0	0.00	0	0	0.00	1.0000
Sleepiness	2	2	0.22	2	2	0.22	4	4	0.22	1.0000
Grade 1	2	2	0.22	2	2	0.22	4	4	0.22	1.0000
Grade 2	0	0	0.00	0	0	0.00	0	0	0.00	1.0000
Grade 3	0	0	0.00	0	0	0.00	0	0	0.00	1.0000
Grade 4	0	0	0.00	0	0	0.00	0	0	0.00	1.0000
Grade 5	0	0	0.00	0	0	0.00	0	0	0.00	1.0000
Grade 2 and above	0	0	0.00	0	0	0.00	0	0	0.00	1.0000
Grade 3 and above	0	0	0.00	0	0	0.00	0	0	0.00	1.0000

Adverse Events<br>Severity	Recombinant vaccine group (N=896) Adverse events	Cases	Incidence (%)	Inactivated vaccine group (N=904) Adverse events	Cases	Incidence (%)	Total (N=1800) Adverse events	Cases	Incidence (%)	P Value[1]
Hypoaesthesia	1	1	0.11	0	0	0.00	1	1	0.06	0.4978
Grade 1	1	1	0.11	0	0	0.00	1	1	0.06	0.4978
Grade 2	0	0	0.00	0	0	0.00	0	0	0.00	1.0000
Grade 3	0	0	0.00	0	0	0.00	0	0	0.00	1.0000
Grade 4	0	0	0.00	0	0	0.00	0	0	0.00	1.0000
Grade 5	0	0	0.00	0	0	0.00	0	0	0.00	1.0000
Grade 2 and above	0	0	0.00	0	0	0.00	0	0	0.00	1.0000
Grade 3 and above	0	0	0.00	0	0	0.00	0	0	0.00	1.0000
Muscle weakness	0	0	0.00	1	1	0.11	1	1	0.06	1.0000
Grade 1	0	0	0.00	1	1	0.11	1	1	0.06	1.0000
Grade 2	0	0	0.00	0	0	0.00	0	0	0.00	1.0000
Grade 3	0	0	0.00	0	0	0.00	0	0	0.00	1.0000
Grade 4	0	0	0.00	0	0	0.00	0	0	0.00	1.0000
Grade 5	0	0	0.00	0	0	0.00	0	0	0.00	1.0000
Grade 2 and above	0	0	0.00	0	0	0.00	0	0	0.00	1.0000
Grade 3 and above	0	0	0.00	0	0	0.00	0	0	0.00	1.0000
**Musculoskeletal and connective tissue disorders**	38	36	4.02	33	30	3.32	71	66	3.67	0.4538
Grade 1	21	21	2.34	20	18	1.99	41	39	2.17	0.6308
Grade 2	15	14	1.56	12	12	1.33	27	26	1.44	0.6979
Grade 3	2	2	0.22	1	1	0.11	3	3	0.17	0.6231
Grade 4	0	0	0.00	0	0	0.00	0	0	0.00	1.0000
Grade 5	0	0	0.00	0	0	0.00	0	0	0.00	1.0000
Grade 2 and above	17	16	1.79	13	13	1.44	30	29	1.61	0.5801
Grade 3 and above	2	2	0.22	1	1	0.11	3	3	0.17	0.6231
Myalgia	36	34	3.79	31	30	3.32	67	64	3.56	0.6124
Grade 1	20	20	2.23	18	18	1.99	38	38	2.11	0.7457
Grade 2	14	13	1.45	12	12	1.33	26	25	1.39	0.8433
Grade 3	2	2	0.22	1	1	0.11	3	3	0.17	0.6231
Grade 4	0	0	0.00	0	0	0.00	0	0	0.00	1.0000
Grade 5	0	0	0.00	0	0	0.00	0	0	0.00	1.0000
Grade 2 and above	16	15	1.67	13	13	1.44	29	28	1.56	0.7078
Grade 3 and above	2	2	0.22	1	1	0.11	3	3	0.17	0.6231
Arthralgia	2	2	0.22	2	2	0.22	4	4	0.22	1.0000
Grade 1	1	1	0.11	2	2	0.22	3	3	0.17	1.0000
Grade 2	1	1	0.11	0	0	0.00	1	1	0.06	0.4978
Grade 3	0	0	0.00	0	0	0.00	0	0	0.00	1.0000
Grade 4	0	0	0.00	0	0	0.00	0	0	0.00	1.0000
Grade 5	0	0	0.00	0	0	0.00	0	0	0.00	1.0000
Grade 2 and above	1	1	0.11	0	0	0.00	1	1	0.06	0.4978
Grade 3 and above	0	0	0.00	0	0	0.00	0	0	0.00	1.0000
**Respiratory, thoracic and mediastinal diseases**	46	32	3.57	49	29	3.21	95	61	3.39	0.6975
Grade 1	29	24	2.68	27	22	2.43	56	46	2.56	0.7671
Grade 2	16	9	1.00	22	14	1.55	38	23	1.28	0.4019
Grade 3	1	1	0.11	0	0	0.00	1	1	0.06	0.4978
Grade 4	0	0	0.00	0	0	0.00	0	0	0.00	1.0000
Grade 5	0	0	0.00	0	0	0.00	0	0	0.00	1.0000
Grade 2 and above	17	10	1.12	22	14	1.55	39	24	1.33	0.5386

Adverse Events	Recombinant vaccine group (N=896)			Inactivated vaccine group (N=904)			Total (N=1800)			
Severity	Adverse events	Cases	Incidence (%)	Adverse events	Cases	Incidence (%)	Adverse events	Cases	Incidence (%)	P Value[1]
Grade 3 and above	1	1	0.11	0	0	0.00	1	1	0.06	0.4978
Coughing	23	22	2.46	22	20	2.21	45	42	2.33	0.7571
Grade 1	16	15	1.67	13	12	1.33	29	27	1.50	0.5671
Grade 2	7	7	0.78	9	9	1.00	16	16	0.89	0.8028
Grade 3	0	0	0.00	0	0	0.00	0	0	0.00	1.0000
Grade 4	0	0	0.00	0	0	0.00	0	0	0.00	1.0000
Grade 5	0	0	0.00	0	0	0.00	0	0	0.00	1.0000
Grade 2 and above	7	7	0.78	9	9	1.00	16	16	0.89	0.8028
Grade 3 and above	0	0	0.00	0	0	0.00	0	0	0.00	1.0000
Runny nose	8	8	0.89	9	9	1.00	17	17	0.94	1.0000
Grade 1	4	4	0.45	5	5	0.55	9	9	0.50	1.0000
Grade 2	4	4	0.45	4	4	0.44	8	8	0.44	1.0000
Grade 3	0	0	0.00	0	0	0.00	0	0	0.00	1.0000
Grade 4	0	0	0.00	0	0	0.00	0	0	0.00	1.0000
Grade 5	0	0	0.00	0	0	0.00	0	0	0.00	1.0000
Grade 2 and above	4	4	0.45	4	4	0.44	8	8	0.44	1.0000
Grade 3 and above	0	0	0.00	0	0	0.00	0	0	0.00	1.0000
Oropharyngeal pain	8	8	0.89	6	6	0.66	14	14	0.78	0.6043
Grade 1	4	4	0.45	3	3	0.33	7	7	0.39	0.7249
Grade 2	3	3	0.33	3	3	0.33	6	6	0.33	1.0000
Grade 3	1	1	0.11	0	0	0.00	1	1	0.06	0.4978
Grade 4	0	0	0.00	0	0	0.00	0	0	0.00	1.0000
Grade 5	0	0	0.00	0	0	0.00	0	0	0.00	1.0000
Grade 2 and above	4	4	0.45	3	3	0.33	7	7	0.39	0.7249
Grade 3 and above	1	1	0.11	0	0	0.00	1	1	0.06	0.4978
Nasal obstruction	5	5	0.56	6	6	0.66	11	11	0.61	1.0000
Grade 1	4	4	0.45	2	2	0.22	6	6	0.33	0.4504
Grade 2	1	1	0.11	4	4	0.44	5	5	0.28	0.3743
Grade 3	0	0	0.00	0	0	0.00	0	0	0.00	1.0000
Grade 4	0	0	0.00	0	0	0.00	0	0	0.00	1.0000
Grade 5	0	0	0.00	0	0	0.00	0	0	0.00	1.0000
Grade 2 and above	1	1	0.11	4	4	0.44	5	5	0.28	0.3743
Grade 3 and above	0	0	0.00	0	0	0.00	0	0	0.00	1.0000
Dyspnea	1	1	0.11	3	3	0.33	4	4	0.22	0.6246
Grade 1	0	0	0.00	2	2	0.22	2	2	0.11	0.4997
Grade 2	1	1	0.11	1	1	0.11	2	2	0.11	1.0000
Grade 3	0	0	0.00	0	0	0.00	0	0	0.00	1.0000
Grade 4	0	0	0.00	0	0	0.00	0	0	0.00	1.0000
Grade 5	0	0	0.00	0	0	0.00	0	0	0.00	1.0000
Grade 2 and above	1	1	0.11	1	1	0.11	2	2	0.11	1.0000
Grade 3 and above	0	0	0.00	0	0	0.00	0	0	0.00	1.0000
Sneeze	0	0	0.00	2	2	0.22	2	2	0.11	0.4997
Grade 1	0	0	0.00	1	1	0.11	1	1	0.06	1.0000
Grade 2	0	0	0.00	1	1	0.11	1	1	0.06	1.0000
Grade 3	0	0	0.00	0	0	0.00	0	0	0.00	1.0000
Grade 4	0	0	0.00	0	0	0.00	0	0	0.00	1.0000
Grade 5	0	0	0.00	0	0	0.00	0	0	0.00	1.0000
Grade 2 and above	0	0	0.00	1	1	0.11	1	1	0.06	1.0000
Grade 3 and above	0	0	0.00	0	0	0.00	0	0	0.00	1.0000
Epistaxis	0	0	0.00	1	1	0.11	1	1	0.06	1.0000

Adverse Events	Recombinant vaccine group (N=896)			Inactivated vaccine group (N=904)			Total (N=1800)			
Severity	Adverse events	Cases	Incidence (%)	Adverse events	Cases	Incidence (%)	Adverse events	Cases	Incidence (%)	P Value[1]
Grade 1	0	0	0.00	1	1	0.11	1	1	0.06	1.0000
Grade 2	0	0	0.00	0	0	0.00	0	0	0.00	1.0000
Grade 3	0	0	0.00	0	0	0.00	0	0	0.00	1.0000
Grade 4	0	0	0.00	0	0	0.00	0	0	0.00	1.0000
Grade 5	0	0	0.00	0	0	0.00	0	0	0.00	1.0000
Grade 2 and above	0	0	0.00	0	0	0.00	0	0	0.00	1.0000
Grade 3 and above	0	0	0.00	0	0	0.00	0	0	0.00	1.0000
Allergic coryza	1	1	0.11	0	0	0.00	1	1	0.06	0.4978
Grade 1	1	1	0.11	0	0	0.00	1	1	0.06	0.4978
Grade 2	0	0	0.00	0	0	0.00	0	0	0.00	1.0000
Grade 3	0	0	0.00	0	0	0.00	0	0	0.00	1.0000
Grade 4	0	0	0.00	0	0	0.00	0	0	0.00	1.0000
Grade 5	0	0	0.00	0	0	0.00	0	0	0.00	1.0000
Grade 2 and above	0	0	0.00	0	0	0.00	0	0	0.00	1.0000
Grade 3 and above	0	0	0.00	0	0	0.00	0	0	0.00	1.0000
**Gastrointestinal disorders**	15	12	1.34	8	7	0.77	23	19	1.06	0.2587
Grade 1	11	10	1.12	7	6	0.66	18	16	0.89	0.3281
Grade 2	4	2	0.22	1	1	0.11	5	3	0.17	0.6231
Grade 3	0	0	0.00	0	0	0.00	0	0	0.00	1.0000
Grade 4	0	0	0.00	0	0	0.00	0	0	0.00	1.0000
Grade 5	0	0	0.00	0	0	0.00	0	0	0.00	1.0000
Grade 2 and above	4	2	0.22	1	1	0.11	5	3	0.17	0.6231
Grade 3 and above	0	0	0.00	0	0	0.00	0	0	0.00	1.0000
Nausea	5	5	0.56	2	2	0.22	7	7	0.39	0.2861
Grade 1	4	4	0.45	2	2	0.22	6	6	0.33	0.4504
Grade 2	1	1	0.11	0	0	0.00	1	1	0.06	0.4978
Grade 3	0	0	0.00	0	0	0.00	0	0	0.00	1.0000
Grade 4	0	0	0.00	0	0	0.00	0	0	0.00	1.0000
Grade 5	0	0	0.00	0	0	0.00	0	0	0.00	1.0000
Grade 2 and above	1	1	0.11	0	0	0.00	1	1	0.06	0.4978
Grade 3 and above	0	0	0.00	0	0	0.00	0	0	0.00	1.0000
Diarrhoea	2	2	0.22	2	2	0.22	4	4	0.22	1.0000
Grade 1	2	2	0.22	2	2	0.22	4	4	0.22	1.0000
Grade 2	0	0	0.00	0	0	0.00	0	0	0.00	1.0000
Grade 3	0	0	0.00	0	0	0.00	0	0	0.00	1.0000
Grade 4	0	0	0.00	0	0	0.00	0	0	0.00	1.0000
Grade 5	0	0	0.00	0	0	0.00	0	0	0.00	1.0000
Grade 2 and above	0	0	0.00	0	0	0.00	0	0	0.00	1.0000
Grade 3 and above	0	0	0.00	0	0	0.00	0	0	0.00	1.0000
Abdominal pai	0	0	0.00	3	3	0.33	3	3	0.17	0.2496
Grade 1	0	0	0.00	2	2	0.22	2	2	0.11	0.4997
Grade 2	0	0	0.00	1	1	0.11	1	1	0.06	1.0000
Grade 3	0	0	0.00	0	0	0.00	0	0	0.00	1.0000
Grade 4	0	0	0.00	0	0	0.00	0	0	0.00	1.0000
Grade 5	0	0	0.00	0	0	0.00	0	0	0.00	1.0000
Grade 2 and above	0	0	0.00	1	1	0.11	1	1	0.06	1.0000
Grade 3 and above	0	0	0.00	0	0	0.00	0	0	0.00	1.0000
Vomiting	2	2	0.22	1	1	0.11	3	3	0.17	0.6231
Grade 1	1	1	0.11	1	1	0.11	2	2	0.11	1.0000
Grade 2	1	1	0.11	0	0	0.00	1	1	0.06	0.4978
Grade 3	0	0	0.00	0	0	0.00	0	0	0.00	1.0000

Adverse Events<br>Severity	Recombinant vaccine group (N=896)			Inactivated vaccine group (N=904)			Total (N=1800)			P Value[1]
	Adverse events	Cases	Incidence (%)	Adverse events	Cases	Incidence (%)	Adverse events	Cases	Incidence (%)	
Grade 4	0	0	0.00	0	0	0.00	0	0	0.00	1.0000
Grade 5	0	0	0.00	0	0	0.00	0	0	0.00	1.0000
Grade 2 and above	1	1	0.11	0	0	0.00	1	1	0.06	0.4978
Grade 3 and above	0	0	0.00	0	0	0.00	0	0	0.00	1.0000
Abdominal pain upper	2	2	0.22	0	0	0.00	2	2	0.11	0.2476
Grade 1	0	0	0.00	0	0	0.00	0	0	0.00	1.0000
Grade 2	2	2	0.22	0	0	0.00	2	2	0.11	0.2476
Grade 3	0	0	0.00	0	0	0.00	0	0	0.00	1.0000
Grade 4	0	0	0.00	0	0	0.00	0	0	0.00	1.0000
Grade 5	0	0	0.00	0	0	0.00	0	0	0.00	1.0000
Grade 2 and above	2	2	0.22	0	0	0.00	2	2	0.11	0.2476
Grade 3 and above	0	0	0.00	0	0	0.00	0	0	0.00	1.0000
Abdominal distension	1	1	0.11	0	0	0.00	1	1	0.06	0.4978
Grade 1	1	1	0.11	0	0	0.00	1	1	0.06	0.4978
Grade 2	0	0	0.00	0	0	0.00	0	0	0.00	1.0000
Grade 3	0	0	0.00	0	0	0.00	0	0	0.00	1.0000
Grade 4	0	0	0.00	0	0	0.00	0	0	0.00	1.0000
Grade 5	0	0	0.00	0	0	0.00	0	0	0.00	1.0000
Grade 2 and above	0	0	0.00	0	0	0.00	0	0	0.00	1.0000
Grade 3 and above	0	0	0.00	0	0	0.00	0	0	0.00	1.0000
Dry mouth	1	1	0.11	0	0	0.00	1	1	0.06	0.4978
Grade 1	1	1	0.11	0	0	0.00	1	1	0.06	0.4978
Grade 2	0	0	0.00	0	0	0.00	0	0	0.00	1.0000
Grade 3	0	0	0.00	0	0	0.00	0	0	0.00	1.0000
Grade 4	0	0	0.00	0	0	0.00	0	0	0.00	1.0000
Grade 5	0	0	0.00	0	0	0.00	0	0	0.00	1.0000
Grade 2 and above	0	0	0.00	0	0	0.00	0	0	0.00	1.0000
Grade 3 and above	0	0	0.00	0	0	0.00	0	0	0.00	1.0000
Oral pain	1	1	0.11	0	0	0.00	1	1	0.06	0.4978
Grade 1	1	1	0.11	0	0	0.00	1	1	0.06	0.4978
Grade 2	0	0	0.00	0	0	0.00	0	0	0.00	1.0000
Grade 3	0	0	0.00	0	0	0.00	0	0	0.00	1.0000
Grade 4	0	0	0.00	0	0	0.00	0	0	0.00	1.0000
Grade 5	0	0	0.00	0	0	0.00	0	0	0.00	1.0000
Grade 2 and above	0	0	0.00	0	0	0.00	0	0	0.00	1.0000
Grade 3 and above	0	0	0.00	0	0	0.00	0	0	0.00	1.0000
Poor digestion	1	1	0.11	0	0	0.00	1	1	0.06	0.4978
Grade 1	1	1	0.11	0	0	0.00	1	1	0.06	0.4978
Grade 2	0	0	0.00	0	0	0.00	0	0	0.00	1.0000
Grade 3	0	0	0.00	0	0	0.00	0	0	0.00	1.0000
Grade 4	0	0	0.00	0	0	0.00	0	0	0.00	1.0000
Grade 5	0	0	0.00	0	0	0.00	0	0	0.00	1.0000
Grade 2 and above	0	0	0.00	0	0	0.00	0	0	0.00	1.0000
Grade 3 and above	0	0	0.00	0	0	0.00	0	0	0.00	1.0000
**Infections and infestations**	6	6	0.67	5	4	0.44	11	10	0.56	0.5460
Grade 1	6	6	0.67	4	4	0.44	10	10	0.56	0.5460
Grade 2	0	0	0.00	1	1	0.11	1	1	0.06	1.0000
Grade 3	0	0	0.00	0	0	0.00	0	0	0.00	1.0000
Grade 4	0	0	0.00	0	0	0.00	0	0	0.00	1.0000

Adverse Events	Recombinant vaccine group (N=896)			Inactivated vaccine group (N=904)			Total (N=1800)			
Severity	Adverse events	Cases	Incidence (%)	Adverse events	Cases	Incidence (%)	Adverse events	Cases	Incidence (%)	P Value[1]
Grade 5	0	0	0.00	0	0	0.00	0	0	0.00	1.0000
Grade 2 and above	0	0	0.00	1	1	0.11	1	1	0.06	1.0000
Grade 3 and above	0	0	0.00	0	0	0.00	0	0	0.00	1.0000
Nasopharyngitis	6	6	0.67	3	3	0.33	9	9	0.50	0.3405
Grade 1	6	6	0.67	3	3	0.33	9	9	0.50	0.3405
Grade 2	0	0	0.00	0	0	0.00	0	0	0.00	1.0000
Grade 3	0	0	0.00	0	0	0.00	0	0	0.00	1.0000
Grade 4	0	0	0.00	0	0	0.00	0	0	0.00	1.0000
Grade 5	0	0	0.00	0	0	0.00	0	0	0.00	1.0000
Grade 2 and above	0	0	0.00	0	0	0.00	0	0	0.00	1.0000
Grade 3 and above	0	0	0.00	0	0	0.00	0	0	0.00	1.0000
Influenza	0	0	0.00	2	1	0.11	2	1	0.06	1.0000
Grade 1	0	0	0.00	1	1	0.11	1	1	0.06	1.0000
Grade 2	0	0	0.00	1	1	0.11	1	1	0.06	1.0000
Grade 3	0	0	0.00	0	0	0.00	0	0	0.00	1.0000
Grade 4	0	0	0.00	0	0	0.00	0	0	0.00	1.0000
Grade 5	0	0	0.00	0	0	0.00	0	0	0.00	1.0000
Grade 2 and above	0	0	0.00	1	1	0.11	1	1	0.06	1.0000
Grade 3 and above	0	0	0.00	0	0	0.00	0	0	0.00	1.0000
**Skin and subcutaneous tissue disorders**	3	3	0.33	5	4	0.44	8	7	0.39	1.0000
Grade 1	3	3	0.33	1	1	0.11	4	4	0.22	0.3724
Grade 2	0	0	0.00	4	4	0.44	4	4	0.22	0.1246
Grade 3	0	0	0.00	0	0	0.00	0	0	0.00	1.0000
Grade 4	0	0	0.00	0	0	0.00	0	0	0.00	1.0000
Grade 5	0	0	0.00	0	0	0.00	0	0	0.00	1.0000
Grade 2 and above	0	0	0.00	4	4	0.44	4	4	0.22	0.1246
Grade 3 and above	0	0	0.00	0	0	0.00	0	0	0.00	1.0000
Pruritus	2	2	0.22	4	4	0.44	6	6	0.33	0.6870
Grade 1	2	2	0.22	0	0	0.00	2	2	0.11	0.2476
Grade 2	0	0	0.00	4	4	0.44	4	4	0.22	0.1246
Grade 3	0	0	0.00	0	0	0.00	0	0	0.00	1.0000
Grade 4	0	0	0.00	0	0	0.00	0	0	0.00	1.0000
Grade 5	0	0	0.00	0	0	0.00	0	0	0.00	1.0000
Grade 2 and above	0	0	0.00	4	4	0.44	4	4	0.22	0.1246
Grade 3 and above	0	0	0.00	0	0	0.00	0	0	0.00	1.0000
Erythema	1	1	0.11	0	0	0.00	1	1	0.06	0.4978
Grade 1	1	1	0.11	0	0	0.00	1	1	0.06	0.4978
Grade 2	0	0	0.00	0	0	0.00	0	0	0.00	1.0000
Grade 3	0	0	0.00	0	0	0.00	0	0	0.00	1.0000
Grade 4	0	0	0.00	0	0	0.00	0	0	0.00	1.0000
Grade 5	0	0	0.00	0	0	0.00	0	0	0.00	1.0000
Grade 2 and above	0	0	0.00	0	0	0.00	0	0	0.00	1.0000
Grade 3 and above	0	0	0.00	0	0	0.00	0	0	0.00	1.0000
Rash	0	0	0.00	1	1	0.11	1	1	0.06	1.0000
Grade 1	0	0	0.00	1	1	0.11	1	1	0.06	1.0000
Grade 2	0	0	0.00	0	0	0.00	0	0	0.00	1.0000
Grade 3	0	0	0.00	0	0	0.00	0	0	0.00	1.0000
Grade 4	0	0	0.00	0	0	0.00	0	0	0.00	1.0000
Grade 5	0	0	0.00	0	0	0.00	0	0	0.00	1.0000
Grade 2 and above	0	0	0.00	0	0	0.00	0	0	0.00	1.0000

Adverse Events	Recombinant vaccine group (N=896)			Inactivated vaccine group (N=904)			Total (N=1800)			
Severity	Adverse events	Cases	Incidence (%)	Adverse events	Cases	Incidence (%)	Adverse events	Cases	Incidence (%)	P Value[1]
Grade 3 and above	0	0	0.00	0	0	0.00	0	0	0.00	1.0000
**Psychiatric disorders**	2	2	0.22	1	1	0.11	3	3	0.17	0.6231
Grade 1	2	2	0.22	1	1	0.11	3	3	0.17	0.6231
Grade 2	0	0	0.00	0	0	0.00	0	0	0.00	1.0000
Grade 3	0	0	0.00	0	0	0.00	0	0	0.00	1.0000
Grade 4	0	0	0.00	0	0	0.00	0	0	0.00	1.0000
Grade 5	0	0	0.00	0	0	0.00	0	0	0.00	1.0000
Grade 2 and above	0	0	0.00	0	0	0.00	0	0	0.00	1.0000
Grade 3 and above	0	0	0.00	0	0	0.00	0	0	0.00	1.0000
Insomnia	2	2	0.22	1	1	0.11	3	3	0.17	0.6231
Grade 1	2	2	0.22	1	1	0.11	3	3	0.17	0.6231
Grade 2	0	0	0.00	0	0	0.00	0	0	0.00	1.0000
Grade 3	0	0	0.00	0	0	0.00	0	0	0.00	1.0000
Grade 4	0	0	0.00	0	0	0.00	0	0	0.00	1.0000
Grade 5	0	0	0.00	0	0	0.00	0	0	0.00	1.0000
Grade 2 and above	0	0	0.00	0	0	0.00	0	0	0.00	1.0000
Grade 3 and above	0	0	0.00	0	0	0.00	0	0	0.00	1.0000
**Eye disorders**	3	3	0.33	0	0	0.00	3	3	0.17	0.1231
Grade 1	1	1	0.11	0	0	0.00	1	1	0.06	0.4978
Grade 2	2	2	0.22	0	0	0.00	2	2	0.11	0.2476
Grade 3	0	0	0.00	0	0	0.00	0	0	0.00	1.0000
Grade 4	0	0	0.00	0	0	0.00	0	0	0.00	1.0000
Grade 5	0	0	0.00	0	0	0.00	0	0	0.00	1.0000
Grade 2 and above	2	2	0.22	0	0	0.00	2	2	0.11	0.2476
Grade 3 and above	0	0	0.00	0	0	0.00	0	0	0.00	1.0000
Ocular hyperemia	1	1	0.11	0	0	0.00	1	1	0.06	0.4978
Grade 1	1	1	0.11	0	0	0.00	1	1	0.06	0.4978
Grade 2	0	0	0.00	0	0	0.00	0	0	0.00	1.0000
Grade 3	0	0	0.00	0	0	0.00	0	0	0.00	1.0000
Grade 4	0	0	0.00	0	0	0.00	0	0	0.00	1.0000
Grade 5	0	0	0.00	0	0	0.00	0	0	0.00	1.0000
Grade 2 and above	0	0	0.00	0	0	0.00	0	0	0.00	1.0000
Grade 3 and above	0	0	0.00	0	0	0.00	0	0	0.00	1.0000
Eye irritation	1	1	0.11	0	0	0.00	1	1	0.06	0.4978
Grade 1	0	0	0.00	0	0	0.00	0	0	0.00	1.0000
Grade 2	1	1	0.11	0	0	0.00	1	1	0.06	0.4978
Grade 3	0	0	0.00	0	0	0.00	0	0	0.00	1.0000
Grade 4	0	0	0.00	0	0	0.00	0	0	0.00	1.0000
Grade 5	0	0	0.00	0	0	0.00	0	0	0.00	1.0000
Grade 2 and above	1	1	0.11	0	0	0.00	1	1	0.06	0.4978
Grade 3 and above	0	0	0.00	0	0	0.00	0	0	0.00	1.0000
Eye pruritus	1	1	0.11	0	0	0.00	1	1	0.06	0.4978
Grade 1	0	0	0.00	0	0	0.00	0	0	0.00	1.0000
Grade 2	1	1	0.11	0	0	0.00	1	1	0.06	0.4978
Grade 3	0	0	0.00	0	0	0.00	0	0	0.00	1.0000
Grade 4	0	0	0.00	0	0	0.00	0	0	0.00	1.0000
Grade 5	0	0	0.00	0	0	0.00	0	0	0.00	1.0000
Grade 2 and above	1	1	0.11	0	0	0.00	1	1	0.06	0.4978
Grade 3 and above	0	0	0.00	0	0	0.00	0	0	0.00	1.0000

Adverse Events	Recombinant vaccine group (N=896)			Inactivated vaccine group (N=904)			Total (N=1800)			
Severity	Adverse events	Cases	Incidence (%)	Adverse events	Cases	Incidence (%)	Adverse events	Cases	Incidence (%)	P Value[1]
**Renal and urinary disorders**	3	2	0.22	0	0	0.00	3	2	0.11	0.2476
Grade 1	3	2	0.22	0	0	0.00	3	2	0.11	0.2476
Grade 2	0	0	0.00	0	0	0.00	0	0	0.00	1.0000
Grade 3	0	0	0.00	0	0	0.00	0	0	0.00	1.0000
Grade 4	0	0	0.00	0	0	0.00	0	0	0.00	1.0000
Grade 5	0	0	0.00	0	0	0.00	0	0	0.00	1.0000
Grade 2 and above	0	0	0.00	0	0	0.00	0	0	0.00	1.0000
Grade 3 and above	0	0	0.00	0	0	0.00	0	0	0.00	1.0000
Polyuria	1	1	0.11	0	0	0.00	1	1	0.06	0.4978
Grade 1	1	1	0.11	0	0	0.00	1	1	0.06	0.4978
Grade 2	0	0	0.00	0	0	0.00	0	0	0.00	1.0000
Grade 3	0	0	0.00	0	0	0.00	0	0	0.00	1.0000
Grade 4	0	0	0.00	0	0	0.00	0	0	0.00	1.0000
Grade 5	0	0	0.00	0	0	0.00	0	0	0.00	1.0000
Grade 2 and above	0	0	0.00	0	0	0.00	0	0	0.00	1.0000
Grade 3 and above	0	0	0.00	0	0	0.00	0	0	0.00	1.0000
Urinary frequency	1	1	0.11	0	0	0.00	1	1	0.06	0.4978
Grade 1	1	1	0.11	0	0	0.00	1	1	0.06	0.4978
Grade 2	0	0	0.00	0	0	0.00	0	0	0.00	1.0000
Grade 3	0	0	0.00	0	0	0.00	0	0	0.00	1.0000
Grade 4	0	0	0.00	0	0	0.00	0	0	0.00	1.0000
Grade 5	0	0	0.00	0	0	0.00	0	0	0.00	1.0000
Grade 2 and above	0	0	0.00	0	0	0.00	0	0	0.00	1.0000
Grade 3 and above	0	0	0.00	0	0	0.00	0	0	0.00	1.0000
Dysuria	1	1	0.11	0	0	0.00	1	1	0.06	0.4978
Grade 1	1	1	0.11	0	0	0.00	1	1	0.06	0.4978
Grade 2	0	0	0.00	0	0	0.00	0	0	0.00	1.0000
Grade 3	0	0	0.00	0	0	0.00	0	0	0.00	1.0000
Grade 4	0	0	0.00	0	0	0.00	0	0	0.00	1.0000
Grade 5	0	0	0.00	0	0	0.00	0	0	0.00	1.0000
Grade 2 and above	0	0	0.00	0	0	0.00	0	0	0.00	1.0000
Grade 3 and above	0	0	0.00	0	0	0.00	0	0	0.00	1.0000
**Ear and labyrinth disorders**	1	1	0.11	0	0	0.00	1	1	0.06	0.4978
Grade 1	0	0	0.00	0	0	0.00	0	0	0.00	1.0000
Grade 2	1	1	0.11	0	0	0.00	1	1	0.06	0.4978
Grade 3	0	0	0.00	0	0	0.00	0	0	0.00	1.0000
Grade 4	0	0	0.00	0	0	0.00	0	0	0.00	1.0000
Grade 5	0	0	0.00	0	0	0.00	0	0	0.00	1.0000
Grade 2 and above	1	1	0.11	0	0	0.00	1	1	0.06	0.4978
Grade 3 and above	0	0	0.00	0	0	0.00	0	0	0.00	1.0000
Ear pain	1	1	0.11	0	0	0.00	1	1	0.06	0.4978
Grade 1	0	0	0.00	0	0	0.00	0	0	0.00	1.0000
Grade 2	1	1	0.11	0	0	0.00	1	1	0.06	0.4978
Grade 3	0	0	0.00	0	0	0.00	0	0	0.00	1.0000
Grade 4	0	0	0.00	0	0	0.00	0	0	0.00	1.0000
Grade 5	0	0	0.00	0	0	0.00	0	0	0.00	1.0000
Grade 2 and above	1	1	0.11	0	0	0.00	1	1	0.06	0.4978
Grade 3 and above	0	0	0.00	0	0	0.00	0	0	0.00	1.0000
**Various inspections**	0	0	0.00	1	1	0.11	1	1	0.06	1.0000

Adverse Events<br>Severity	Recombinant vaccine group (N=896)			Inactivated vaccine group (N=904)			Total (N=1800)			P Value[1]
	Adverse events	Cases	Incidence (%)	Adverse events	Cases	Incidence (%)	Adverse events	Cases	Incidence (%)	
Grade 1	0	0	0.00	1	1	0.11	1	1	0.06	1.0000
Grade 2	0	0	0.00	0	0	0.00	0	0	0.00	1.0000
Grade 3	0	0	0.00	0	0	0.00	0	0	0.00	1.0000
Grade 4	0	0	0.00	0	0	0.00	0	0	0.00	1.0000
Grade 5	0	0	0.00	0	0	0.00	0	0	0.00	1.0000
Grade 2 and above	0	0	0.00	0	0	0.00	0	0	0.00	1.0000
Grade 3 and above	0	0	0.00	0	0	0.00	0	0	0.00	1.0000
Heart rate increased	0	0	0.00	1	1	0.11	1	1	0.06	1.0000
Grade 1	0	0	0.00	1	1	0.11	1	1	0.06	1.0000
Grade 2	0	0	0.00	0	0	0.00	0	0	0.00	1.0000
Grade 3	0	0	0.00	0	0	0.00	0	0	0.00	1.0000
Grade 4	0	0	0.00	0	0	0.00	0	0	0.00	1.0000
Grade 5	0	0	0.00	0	0	0.00	0	0	0.00	1.0000
Grade 2 and above	0	0	0.00	0	0	0.00	0	0	0.00	1.0000
Grade 3 and above	0	0	0.00	0	0	0.00	0	0	0.00	1.0000
**Immune system disease**	0	0	0.00	1	1	0.11	1	1	0.06	1.0000
Grade 1	0	0	0.00	1	1	0.11	1	1	0.06	1.0000
Grade 2	0	0	0.00	0	0	0.00	0	0	0.00	1.0000
Grade 3	0	0	0.00	0	0	0.00	0	0	0.00	1.0000
Grade 4	0	0	0.00	0	0	0.00	0	0	0.00	1.0000
Grade 5	0	0	0.00	0	0	0.00	0	0	0.00	1.0000
Grade 2 and above	0	0	0.00	0	0	0.00	0	0	0.00	1.0000
Grade 3 and above	0	0	0.00	0	0	0.00	0	0	0.00	1.0000
Hypersensitivity reaction	0	0	0.00	1	1	0.11	1	1	0.06	1.0000
Grade 1	0	0	0.00	1	1	0.11	1	1	0.06	1.0000
Grade 2	0	0	0.00	0	0	0.00	0	0	0.00	1.0000
Grade 3	0	0	0.00	0	0	0.00	0	0	0.00	1.0000
Grade 4	0	0	0.00	0	0	0.00	0	0	0.00	1.0000
Grade 5	0	0	0.00	0	0	0.00	0	0	0.00	1.0000
Grade 2 and above	0	0	0.00	0	0	0.00	0	0	0.00	1.0000
Grade 3 and above	0	0	0.00	0	0	0.00	0	0	0.00	1.0000

[1]P value was calculated by Fisher exact test.

(1) Adverse events were coded using MedDRA version 24.1.

(2) Study vaccine-related means that the relationship between adverse events and study vaccine is "definitely related," "most likely related," or "likely related."

(3) This analysis data is up to January 25, 2022.

1.2.2. Adverse events in each time period

1.2.2.1. Adverse events within 30 minutes

There were 2 cases (0.11%) with 2 adverse events within 30 minutes after the whole population was immunized with a booster dose, among which 0 cases (0.00%) with 0 adverse events occurred in the recombinant vaccine group and 2 cases (0.22%) with 2 adverse events occurred in the inactivated vaccine group. There was no significant difference between the groups. 2 cases that occurred in the inactivated vaccine group were

dysphagia. No adverse events related to the study vaccine were observed in the recombinant vaccine and the inactivated vaccine groups.

See Table 1-4, Table 1-5, Table 1-6, and Table 1-7 for details.

Table 1-4 Adverse events within 30 minutes of the whole population during the booster period (by SOC and PT) (SS)

	Recombinant vaccine group (N=896)			Inactivated vaccine group (N=904)			Total (N=1800)			
**Adverse Events**	**Adverse events**	**Cases**	**Incidence (%)**	**Adverse events**	**Cases**	**Incidence (%)**	**Adverse events**	**Cases**	**Incidence (%)**	**P Value[1]**
TEAE	0	0	0.00	2	2	0.22	2	2	0.11	0.4997
**Systemic diseases and various reactions at the administration site**	0	0	0.00	0	0	0.00	0	0	0.00	1.0000
**Nervous system disorders**	0	0	0.00	0	0	0.00	0	0	0.00	1.0000
**Respiratory, thoracic and mediastinal diseases**	0	0	0.00	0	0	0.00	0	0	0.00	1.0000
**Musculoskeletal and connective tissue disorders**	0	0	0.00	0	0	0.00	0	0	0.00	1.0000
**Gastrointestinal system diseases**	0	0	0.00	2	2	0.22	2	2	0.11	0.4997
Dysphagia	0	0	0.00	2	2	0.22	2	2	0.11	0.4997
**Infection and infectious diseases**	0	0	0.00	0	0	0.00	0	0	0.00	1.0000
**Skin and subcutaneous tissue disorders**	0	0	0.00	0	0	0.00	0	0	0.00	1.0000
**Psychiatric category**	0	0	0.00	0	0	0.00	0	0	0.00	1.0000
**Immune system disease**	0	0	0.00	0	0	0.00	0	0	0.00	1.0000
**Diseases of kidney and urinary system**	0	0	0.00	0	0	0.00	0	0	0.00	1.0000
**Various inspections**	0	0	0.00	0	0	0.00	0	0	0.00	1.0000
**Ocular organ disease**	0	0	0.00	0	0	0.00	0	0	0.00	1.0000
**Ear and labyrinth disorders**	0	0	0.00	0	0	0.00	0	0	0.00	1.0000
**Reproductive system and breast diseases**	0	0	0.00	0	0	0.00	0	0	0.00	1.0000

Adverse Events	Recombinant vaccine group (N=896)			Inactivated vaccine group (N=904)			Total (N=1800)			
	Adverse events	Cases	Incidence (%)	Adverse events	Cases	Incidence (%)	Adverse events	Cases	Incidence (%)	P Value[1]

[1]P value was calculated by Fisher exact test.
(1) Adverse events were coded using MedDRA version 24.1.
(2) Occurrence time (day) = occurrence date of adverse events-vaccination date of the booster dose
(3) This analysis data is up to January 25, 2022.

Table 1-5 Adverse events related to study vaccine within 30 minutes of the whole population during the booster period (by SOC and PT) (SS)

Adverse Events	Recombinant vaccine group (N=896)			Inactivated vaccine group (N=904)			Total (N=1800)			
	Adverse events	Cases	Incidence (%)	Adverse events	Cases	Incidence (%)	Adverse events	Cases	Incidence (%)	P Value[1]
**TEAE**	0	0	0.00	0	0	0.00	0	0	0.00	1.0000

[1]P value was calculated by Fisher exact test.
(1) Adverse events were coded using MedDRA version 24.1.
(2) Occurrence time (day) = occurrence date of adverse events-vaccination date of the booster dose
(3) Study vaccine-related means that the relationship between adverse events and study vaccine is "definitely related," "most likely related" or " likely related."
(4) This analysis data is up to January 25, 2022.

Table 1-6 Adverse events occurred within 30 minutes of the whole population during the booster period (listed by solicitation and non-solicitation events) (SS)

Adverse Events	Recombinant vaccine group (N=896)			Inactivated vaccine group (N=904)			Total (N=1800)			
	Adverse events	Cases	Incidence (%)	Adverse events	Cases	Incidence (%)	Adverse events	Cases	Incidence (%)	P Value[1]
**TEAE**	0	0	0.00	2	2	0.22	2	2	0.11	0.4997
**Solicited Adverse Events**	0	0	0.00	2	2	0.22	2	2	0.11	0.4997
**Non-inoculation site (whole body)**	0	0	0.00	2	2	0.22	2	2	0.11	0.4997
Fever	0	0	0.00	0	0	0.00	0	0	0.00	1.0000
Diarrhea	0	0	0.00	0	0	0.00	0	0	0.00	1.0000
Dysphagia	0	0	0.00	2	2	0.22	2	2	0.11	0.4997
Vomiting	0	0	0.00	0	0	0.00	0	0	0.00	1.0000
Nausea	0	0	0.00	0	0	0.00	0	0	0.00	1.0000
Muscle pain (non-inoculation site)	0	0	0.00	0	0	0.00	0	0	0.00	1.0000
Arthralgia	0	0	0.00	0	0	0.00	0	0	0.00	1.0000
Headache	0	0	0.00	0	0	0.00	0	0	0.00	1.0000
Cough	0	0	0.00	0	0	0.00	0	0	0.00	1.0000
Dyspnea	0	0	0.00	0	0	0.00	0	0	0.00	1.0000
Fatigue/Asthenia	0	0	0.00	0	0	0.00	0	0	0.00	1.0000
Vertigo	0	0	0.00	0	0	0.00	0	0	0.00	1.0000

Adverse Events	Recombinant vaccine group (N=896)			Inactivated vaccine group (N=904)			Total (N=1800)			P Value[1]
	Adverse events	Cases	Incidence (%)	Adverse events	Cases	Incidence (%)	Adverse events	Cases	Incidence (%)	
**Injection site (local)**	0	0	0.00	0	0	0.00	0	0	0.00	1.0000
Pain	0	0	0.00	0	0	0.00	0	0	0.00	1.0000
Swelling	0	0	0.00	0	0	0.00	0	0	0.00	1.0000
Skin rash	0	0	0.00	0	0	0.00	0	0	0.00	1.0000
Flushing	0	0	0.00	0	0	0.00	0	0	0.00	1.0000
Pruritus	0	0	0.00	0	0	0.00	0	0	0.00	1.0000
**Unsolicited adverse events**	0	0	0.00	0	0	0.00	0	0	0.00	1.0000

[1]P value was calculated by Fisher exact test.
(1) Unsolicited adverse events were coded using MedDRA version 24.1. and solicited adverse events are listed according to the event name specified in the protocol.
(2) Occurrence time (day) = occurrence date of adverse events-vaccination date of the booster dose
(3) This analysis data is up to January 25, 2022.

Table 1-7 Adverse events in the whole population within 30 minutes related to the study vaccine during the booster period (listed by solicitation and non-solicitation events) (SS)

Adverse Events	Recombinant vaccine group (N=896)			Inactivated vaccine group (N=904)			Total (N=1800)			P Value[1]
	Adverse events	Cases	Incidence (%)	Adverse events	Cases	Incidence (%)	Adverse events	Cases	Incidence (%)	
**TEAE**	0	0	0.00	0	0	0.00	0	0	0.00	1.0000

[1]P value was calculated by Fisher exact test.
(1) Unsolicited adverse events were coded using MedDRA version 24.1. and solicited adverse events are listed according to the event name specified in the protocol.
(2) Occurrence time (day) = occurrence date of adverse events-vaccination date of the booster dose
(3) Study vaccine related means that the relationship between adverse events and study vaccine is "definitely related," "most likely related," or " likely related."
(4) This analysis data is up to January 25, 2022.

1.2.2.2. Adverse events within 0-7 days

There were 284 cases (15.78%) with 508 adverse events within 0-7 days after the booster vaccinations, among which 155 cases (17.30%) with 283 adverse events occurred in the recombinant vaccine group, and 129 cases (14.27%) with 225 adverse events occurred in the inactivated vaccine group. There was no significant difference between these two groups. 276 cases (15.33%) with 485 adverse events were related to the study vaccines, of which 153 cases (17.08%) with 269 adverse events occurred in the recombinant vaccine group, and 123 cases (13.61%) with 216 adverse events occurred in the inactivated vaccine group. There was a statistically significant difference between

these two groups (P=0.0427).

According to the classification of SOC and PT, the adverse events related to study vaccines were mainly systemic diseases and various reactions at the administration site. There were 89 cases (9.93%) with 107 adverse events in the recombinant vaccine group and 74 cases (8.19%) with 91 adverse events in the inactivated vaccine group. There was no significant difference between these two groups. Among them, the most common adverse reactions were pain at the vaccination site (4.69% in the recombinant vaccine group and 3.98% in the inactivated vaccine group), followed by fatigue (3.01% in the recombinant vaccine group and 2.43% in the inactivated vaccine group).

By classification of solicited and unsolicited, the solicited adverse events related to the study vaccines occurred in 146 cases (16.29%) with 215 adverse events in the recombinant vaccine group, and 115 cases (12.72%) with 176 adverse events in the inactivated vaccine group. There was a statistically significant difference between these two groups (P=0.0323). Among them, the most common local adverse reactions were pain (the incidence in the recombinant vaccine group was 4.69%, and in inactivated vaccine group was 3.98%). The most common systemic adverse reactions were headache (the incidence in the recombinant vaccine group was 5.80%, and in inactivated vaccine group was 4.20%), followed by muscle pain (non-inoculation site) (the incidence in recombinant vaccine group was3.35%, and in inactivated vaccine group was 2.77%), fatigue/weary (3.01% in recombinant vaccine group and 2.43% in inactivated vaccine group) and fever (2.68% in recombinant vaccine group and 2.43% in inactivated vaccine group). For unsolicited adverse events related to the study vaccine, 41 cases (4.58%) with 54 adverse events occurred in the recombinant vaccine group, and 30 cases (3.32%) with 40 adverse events occurred in the -inactivated vaccine group. There was no statistically significant difference between the groups.

See Table 1-8, Table 1-9, Table 1-10, and Table 1-11 for details.

Table 1-8 Adverse events within 0-7 days of the whole population during the booster period (listed by SOC and PT) (SS)

	Recombinant vaccine group (N=896)			Inactivated vaccine group (N=904)			Total (N=1800)			
**Adverse Events**	**Adverse events**	**Cases**	**Incidence (%)**	**Adverse events**	**Cases**	**Incidence (%)**	**Adverse events**	**Cases**	**Incidence (%)**	**P Value[1]**
**TEAE**	283	155	17.30	225	129	14.27	508	284	15.78	0.0811

Adverse Events	Recombinant vaccine group (N=896)			Inactivated vaccine group (N=904)			Total (N=1800)			P Value[1]
	Adverse events	Cases	Incidence (%)	Adverse events	Cases	Incidence (%)	Adverse events	Cases	Incidence (%)	
**General disorders and administration site conditions**	109	89	9.93	91	74	8.19	200	163	9.06	0.2180
Pain at vaccination site	42	42	4.69	36	36	3.98	78	78	4.33	0.4888
Fever	24	24	2.68	22	22	2.43	46	46	2.56	0.7671
Fatigue	29	28	3.13	22	22	2.43	51	50	2.78	0.3926
Chest pain	2	2	0.22	4	4	0.44	6	6	0.33	0.6870
Feeling of fever	4	4	0.45	0	0	0.00	4	4	0.22	0.0612
Pain	3	3	0.33	1	1	0.11	4	4	0.22	0.3724
Itching at vaccination site	1	1	0.11	2	2	0.22	3	3	0.17	1.0000
Vaccination site erythema	2	2	0.22	0	0	0.00	2	2	0.11	0.2476
Swelling	1	1	0.11	1	1	0.11	2	2	0.11	1.0000
Chills	1	1	0.11	0	0	0.00	1	1	0.06	0.4978
Nodule	0	0	0.00	0	0	0.00	0	0	0.00	1.0000
Influenza-like illness	0	0	0.00	1	1	0.11	1	1	0.06	1.0000
Rash at vaccination site	0	0	0.00	1	1	0.11	1	1	0.06	1.0000
Vaccination site swelling	0	0	0.00	1	1	0.11	1	1	0.06	1.0000
**Various nervous system diseases**	63	56	6.25	44	41	4.54	107	97	5.39	0.1176
Headache	55	53	5.92	38	38	4.20	93	91	5.06	0.1068
Dizziness	2	2	0.22	3	3	0.33	5	5	0.28	1.0000
Sleepiness	3	3	0.33	2	2	0.22	5	5	0.28	0.6858
Hyposensation	1	1	0.11	0	0	0.00	1	1	0.06	0.4978
Dysarthria	0	0	0.00	0	0	0.00	0	0	0.00	1.0000
Myasthenia	0	0	0.00	1	1	0.11	1	1	0.06	1.0000
Loss of taste	1	1	0.11	0	0	0.00	1	1	0.06	0.4978
Loss of olfaction	1	1	0.11	0	0	0.00	1	1	0.06	0.4978
**Respiratory, thoracic and mediastinal disorders**	41	30	3.35	35	22	2.43	76	52	2.89	0.2628
Coughing	21	21	2.34	14	13	1.44	35	34	1.89	0.1697
Runny nose	6	6	0.67	7	7	0.77	13	13	0.72	1.0000
Oropharyngeal pain	7	7	0.78	4	4	0.44	11	11	0.61	0.3842
Nasal congestion	5	5	0.56	5	5	0.55	10	10	0.56	1.0000
Dyspnea	0	0	0.00	2	2	0.22	2	2	0.11	0.4997
Sneezing	0	0	0.00	2	2	0.22	2	2	0.11	0.4997
Epistaxis	0	0	0.00	1	1	0.11	1	1	0.06	1.0000
allergic coryza	1	1	0.11	0	0	0.00	1	1	0.06	0.4978
Hemoptysis	1	1	0.11	0	0	0.00	1	1	0.06	0.4978

Adverse Events	Recombinant vaccine group (N=896)			Inactivated vaccine group (N=904)			Total (N=1800)			P Value[1]
	Adverse events	Cases	Incidence (%)	Adverse events	Cases	Incidence (%)	Adverse events	Cases	Incidence (%)	
**Musculoskeletal and connective tissue disorders**	33	33	3.68	27	25	2.77	60	58	3.22	0.2879
Myalgia	31	31	3.46	25	25	2.77	56	56	3.11	0.4179
Arthralgia	2	2	0.22	2	2	0.22	4	4	0.22	1.0000
**Gastrointestinal system diseases**	16	13	1.45	13	10	1.11	29	23	1.28	0.5370
Nausea	6	6	0.67	3	3	0.33	9	9	0.50	0.3405
Diarrhea	2	2	0.22	2	2	0.22	4	4	0.22	1.0000
Abdominal pain	0	0	0.00	3	3	0.33	3	3	0.17	0.2496
Vomiting	2	2	0.22	1	1	0.11	3	3	0.17	0.6231
Epigastric pain	2	2	0.22	1	1	0.11	3	3	0.17	0.6231
Dysphagia	0	0	0.00	2	2	0.22	2	2	0.11	0.4997
Abdominal distension	1	1	0.11	0	0	0.00	1	1	0.06	0.4978
Dry mouth	1	1	0.11	0	0	0.00	1	1	0.06	0.4978
Oral pain	1	1	0.11	0	0	0.00	1	1	0.06	0.4978
Dyspepsia	1	1	0.11	0	0	0.00	1	1	0.06	0.4978
Toothache	0	0	0.00	1	1	0.11	1	1	0.06	1.0000
**Infection and infectious diseases**	6	6	0.67	5	4	0.44	11	10	0.56	0.5460
Nasopharyngitis	6	6	0.67	3	3	0.33	9	9	0.50	0.3405
Influenza	0	0	0.00	2	1	0.11	2	1	0.06	1.0000
**Skin and subcutaneous tissue disorders**	3	3	0.33	5	4	0.44	8	7	0.39	1.0000
Pruritus	2	2	0.22	4	4	0.44	6	6	0.33	0.6870
Erythema	1	1	0.11	0	0	0.00	1	1	0.06	0.4978
Skin rash	0	0	0.00	1	1	0.11	1	1	0.06	1.0000
**Psychiatric disorders**	3	3	0.33	1	1	0.11	4	4	0.22	0.3724
Insomnia	2	2	0.22	1	1	0.11	3	3	0.17	0.6231
Anxiety	1	1	0.11	0	0	0.00	1	1	0.06	0.4978
**Immune system disease**	0	0	0.00	1	1	0.11	1	1	0.06	1.0000
Hypersensitivity reaction	0	0	0.00	1	1	0.11	1	1	0.06	1.0000
**Diseases of kidney and urinary system**	4	3	0.33	1	1	0.11	5	4	0.22	0.3724
Urinary frequency	1	1	0.11	1	1	0.11	2	2	0.11	1.0000
Polyuria	1	1	0.11	0	0	0.00	1	1	0.06	0.4978
Dysuria	1	1	0.11	0	0	0.00	1	1	0.06	0.4978
Hematuria's disease	1	1	0.11	0	0	0.00	1	1	0.06	0.4978
**Various inspections**	1	1	0.11	2	2	0.22	3	3	0.17	1.0000

Adverse Events	Recombinant vaccine group (N=896)			Inactivated vaccine group (N=904)			Total (N=1800)			P Value[1]
	Adverse events	Cases	Incidence (%)	Adverse events	Cases	Incidence (%)	Adverse events	Cases	Incidence (%)	
Elevated blood pressure	1	1	0.11	1	1	0.11	2	2	0.11	1.0000
Heart rate increased	0	0	0.00	1	1	0.11	1	1	0.06	1.0000
**Ocular organ disease**	3	3	0.33	0	0	0.00	3	3	0.17	0.1231
Ocular hyperemia	1	1	0.11	0	0	0.00	1	1	0.06	0.4978
Ocular irritation	1	1	0.11	0	0	0.00	1	1	0.06	0.4978
Eye pruritus	1	1	0.11	0	0	0.00	1	1	0.06	0.4978
**Ear and labyrinthine diseases**	0	0	0.00	0	0	0.00	0	0	0.00	1.0000
Ear pain	0	0	0.00	0	0	0.00	0	0	0.00	1.0000
**Reproductive system and breast diseases**	1	1	0.11	0	0	0.00	1	1	0.06	0.4978
Erectile dysfunctiom	1	1	0.11	0	0	0.00	1	1	0.06	0.4978

[1]P value was calculated by Fisher exact test.
(1) Adverse events were coded using MedDRA version 24.1.
(2) Occurrence time (day) = occurrence date of adverse events-vaccination date of the booster dose
(3) This analysis data is up to January 25, 2022.

Table 1-9 Adverse events related to study vaccine within 0-7 days of the whole population during the booster period (listed by SOC and PT) (SS)

Adverse Events	Recombinant vaccine group (N=896)			Inactivated vaccine group (N=904)			Total (N=1800)			P Value[1]
	Adverse events	Cases	Incidence (%)	Adverse events	Cases	Incidence (%)	Adverse events	Cases	Incidence (%)	
**TEAE**	269	153	17.08	216	123	13.61	485	276	15.33	0.0427
**Systemic diseases and various reactions at the administration site**	107	89	9.93	91	74	8.19	198	163	9.06	0.2180
Vaccination site Pain	42	42	4.69	36	36	3.98	78	78	4.33	0.4888
Fatigue	28	27	3.01	22	22	2.43	50	49	2.72	0.4721
Fever	24	24	2.68	22	22	2.43	46	46	2.56	0.7671
Chest pain	2	2	0.22	4	4	0.44	6	6	0.33	0.6870
Feeling hot	4	4	0.45	0	0	0.00	4	4	0.22	0.0612
Pain	3	3	0.33	1	1	0.11	4	4	0.22	0.3724
Vaccination site pruritus	1	1	0.11	2	2	0.22	3	3	0.17	1.0000
Erythema at vaccination site	2	2	0.22	0	0	0.00	2	2	0.11	0.2476

Adverse Events	Recombinant vaccine group (N=896)			Inactivated vaccine group (N=904)			Total (N=1800)			P Value[1]
	Adverse events	Cases	Incidence (%)	Adverse events	Cases	Incidence (%)	Adverse events	Cases	Incidence (%)	
Swelling	1	1	0.11	1	1	0.11	2	2	0.11	1.0000
Influenza-like disease	0	0	0.00	1	1	0.11	1	1	0.06	1.0000
Vaccination site rash	0	0	0.00	1	1	0.11	1	1	0.06	1.0000
Swelling of vaccination site	0	0	0.00	1	1	0.11	1	1	0.06	1.0000
**Nervous system disorders**	59	54	6.03	44	41	4.54	103	95	5.28	0.1710
Headache	54	52	5.80	38	38	4.20	92	90	5.00	0.1304
Dizziness	2	2	0.22	3	3	0.33	5	5	0.28	1.0000
Sleepiness	2	2	0.22	2	2	0.22	4	4	0.22	1.0000
Hypoaesthesia	1	1	0.11	0	0	0.00	1	1	0.06	0.4978
Myasthenia	0	0	0.00	1	1	0.11	1	1	0.06	1.0000
**Musculoskeletal and connective tissue disorders**	32	32	3.57	27	25	2.77	59	57	3.17	0.3486
Myalgia	30	30	3.35	25	25	2.77	55	55	3.06	0.4961
Arthralgia	2	2	0.22	2	2	0.22	4	4	0.22	1.0000
**Respiratory, thoracic and mediastinal diseases**	39	30	3.35	33	20	2.21	72	50	2.78	0.1534
Cough	20	20	2.23	14	13	1.44	34	33	1.83	0.2233
Runny nose	6	6	0.67	7	7	0.77	13	13	0.72	1.0000
Oropharyngeal Pain	7	7	0.78	4	4	0.44	11	11	0.61	0.3842
Nasal obstruction	5	5	0.56	4	4	0.44	9	9	0.50	0.7523
Difficulty breathing	0	0	0.00	1	1	0.11	1	1	0.06	1.0000
Sneeze	0	0	0.00	2	2	0.22	2	2	0.11	0.4997
Epistaxis	0	0	0.00	1	1	0.11	1	1	0.06	1.0000
Allergic coryza	1	1	0.11	0	0	0.00	1	1	0.06	0.4978
**Gastrointestinal disorders**	15	12	1.34	8	7	0.77	23	19	1.06	0.2587
Nausea	5	5	0.56	2	2	0.22	7	7	0.39	0.2861
Diarrhoea	2	2	0.22	2	2	0.22	4	4	0.22	1.0000
Abdominal pain	0	0	0.00	3	3	0.33	3	3	0.17	0.2496
Vomiting	2	2	0.22	1	1	0.11	3	3	0.17	0.6231
Epigastric pain	2	2	0.22	0	0	0.00	2	2	0.11	0.2476
Abdominal distension	1	1	0.11	0	0	0.00	1	1	0.06	0.4978
Dry mouth	1	1	0.11	0	0	0.00	1	1	0.06	0.4978
Oral pain	1	1	0.11	0	0	0.00	1	1	0.06	0.4978
Dyspepsia	1	1	0.11	0	0	0.00	1	1	0.06	0.4978
**Infections and infestations**	6	6	0.67	5	4	0.44	11	10	0.56	0.5460
Nasopharyngitis	6	6	0.67	3	3	0.33	9	9	0.50	0.3405

Adverse Events	Recombinant vaccine group (N=896)			Inactivated vaccine group (N=904)			Total (N=1800)			P Value[1]
	Adverse events	Cases	Incidence (%)	Adverse events	Cases	Incidence (%)	Adverse events	Cases	Incidence (%)	
Influenza	0	0	0.00	2	1	0.11	2	1	0.06	1.0000
**Skin and subcutaneous tissue diseases**	3	3	0.33	5	4	0.44	8	7	0.39	1.0000
Pruritus	2	2	0.22	4	4	0.44	6	6	0.33	0.6870
Erythema	1	1	0.11	0	0	0.00	1	1	0.06	0.4978
Rash	0	0	0.00	1	1	0.11	1	1	0.06	1.0000
**Psychiatric category**	2	2	0.22	1	1	0.11	3	3	0.17	0.6231
Insomnia	2	2	0.22	1	1	0.11	3	3	0.17	0.6231
**Ocular organ disease**	3	3	0.33	0	0	0.00	3	3	0.17	0.1231
Ocular hyperemia	1	1	0.11	0	0	0.00	1	1	0.06	0.4978
Ocular irritation	1	1	0.11	0	0	0.00	1	1	0.06	0.4978
Eye pruritus	1	1	0.11	0	0	0.00	1	1	0.06	0.4978
**Diseases of kidney and urinary system**	3	2	0.22	0	0	0.00	3	2	0.11	0.2476
Polyuria	1	1	0.11	0	0	0.00	1	1	0.06	0.4978
Frequency of urination	1	1	0.11	0	0	0.00	1	1	0.06	0.4978
Dysuria	1	1	0.11	0	0	0.00	1	1	0.06	0.4978
**Ear and labyrinthine diseases**	0	0	0.00	0	0	0.00	0	0	0.00	1.0000
Ear pain	0	0	0.00	0	0	0.00	0	0	0.00	1.0000
**Various inspections**	0	0	0.00	1	1	0.11	1	1	0.06	1.0000
Heart rate increased	0	0	0.00	1	1	0.11	1	1	0.06	1.0000
**immune system disease**	0	0	0.00	1	1	0.11	1	1	0.06	1.0000
Hypersensitivity reaction	0	0	0.00	1	1	0.11	1	1	0.06	1.0000

[1]P value was calculated by Fisher exact test.

(1) Adverse events were coded using MedDRA version 24.1.

(2) Occurrence time (day) = occurrence date of adverse events-vaccination date of the booster dose

(3) Study vaccine-related means that the relationship between adverse events and study vaccine is "definitely related," "most likely related," or " likely related."

(4) This analysis data is up to January 25, 2022.

Table 1-10 Adverse events occurred within 0-7 days of the whole population during the booster period (listed per solicitation and non-solicitation events) (SS)

Adverse Events	Recombinant vaccine group (N=896)			Inactivated vaccine group (N=904)			Total (N=1800)			P Value[1]
	Adverse events	Cases	Incidence (%)	Adverse events	Cases	Incidence (%)	Adverse events	Cases	Incidence (%)	
**TEAE**	283	155	17.30	225	129	14.27	508	284	15.78	0.0811
**Solicited Adverse Events**	220	146	16.29	180	118	13.05	400	264	14.67	0.0536
**Non-inoculation site (whole body)**	173	111	12.39	135	87	9.62	308	198	11.00	0.0704
Fever	24	24	2.68	22	22	2.43	46	46	2.56	0.7671
Diarrhea	2	2	0.22	2	2	0.22	4	4	0.22	1.0000
Dysphagia	0	0	0.00	2	2	0.22	2	2	0.11	0.4997
Vomiting	2	2	0.22	1	1	0.11	3	3	0.17	0.6231
Nausea	6	6	0.67	3	3	0.33	9	9	0.50	0.3405
Muscle pain (non-inoculation site)	31	31	3.46	25	25	2.77	56	56	3.11	0.4179
Arthralgia	2	2	0.22	2	2	0.22	4	4	0.22	1.0000
Headache	55	53	5.92	38	38	4.20	93	91	5.06	0.1068
Cough	21	21	2.34	14	13	1.44	35	34	1.89	0.1697
Dyspnea	0	0	0.00	2	2	0.22	2	2	0.11	0.4997
Fatigue/Asthenia	29	28	3.13	22	22	2.43	51	50	2.78	0.3926
Vertigo	1	1	0.11	2	2	0.22	3	3	0.17	1.0000
**Injection site (local)**	47	46	5.13	45	42	4.65	92	88	4.89	0.6629
Pain	42	42	4.69	36	36	3.98	78	78	4.33	0.4888
Swelling	0	0	0.00	1	1	0.11	1	1	0.06	1.0000
Skin rash	0	0	0.00	2	2	0.22	2	2	0.11	0.4997
Flushing	2	2	0.22	0	0	0.00	2	2	0.11	0.2476
Pruritus	3	3	0.33	6	6	0.66	9	9	0.50	0.5068
**Unsolicited adverse events**	63	44	4.91	45	34	3.76	108	78	4.33	0.2482
**Respiratory, thoracic and mediastinal disorders**	20	15	1.67	19	15	1.66	39	30	1.67	1.0000
Coughing	0	0	0.00	0	0	0.00	0	0	0.00	1.0000
Runny nose	6	6	0.67	7	7	0.77	13	13	0.72	1.0000
Oropharyngeal pain	7	7	0.78	4	4	0.44	11	11	0.61	0.3842
Nasal congestion	5	5	0.56	5	5	0.55	10	10	0.56	1.0000
Dyspnea	0	0	0.00	0	0	0.00	0	0	0.00	1.0000
Sneezing	0	0	0.00	2	2	0.22	2	2	0.11	0.4997
Epistaxis	0	0	0.00	1	1	0.11	1	1	0.06	1.0000
Allergic coryza	1	1	0.11	0	0	0.00	1	1	0.06	0.4978
Hemoptysis	1	1	0.11	0	0	0.00	1	1	0.06	0.4978
**General disorders and administration site conditions**	11	10	1.12	7	6	0.66	18	16	0.89	0.3281
Pain	3	3	0.33	1	1	0.11	4	4	0.22	0.3724
Fever	0	0	0.00	0	0	0.00	0	0	0.00	1.0000

Adverse Events	Recombinant vaccine group (N=896)			Inactivated vaccine group (N=904)			Total (N=1800)			P Value[1]
	Adverse events	Cases	Incidence (%)	Adverse events	Cases	Incidence (%)	Adverse events	Cases	Incidence (%)	
Fatigue	0	0	0.00	0	0	0.00	0	0	0.00	1.0000
Chest pain	2	2	0.22	4	4	0.44	6	6	0.33	0.6870
Feeling of fever	4	4	0.45	0	0	0.00	4	4	0.22	0.0612
Swelling	1	1	0.11	1	1	0.11	2	2	0.11	1.0000
Chills	1	1	0.11	0	0	0.00	1	1	0.06	0.4978
Nodule	0	0	0.00	0	0	0.00	0	0	0.00	1.0000
Influenza-like disease	0	0	0.00	1	1	0.11	1	1	0.06	1.0000
Vaccination site pruritus	0	0	0.00	0	0	0.00	0	0	0.00	1.0000
**Various nervous system diseases**	7	6	0.67	4	4	0.44	11	10	0.56	0.5460
Headache	0	0	0.00	0	0	0.00	0	0	0.00	1.0000
Sleepiness	3	3	0.33	2	2	0.22	5	5	0.28	0.6858
Dizziness	1	1	0.11	1	1	0.11	2	2	0.11	1.0000
Hyposensation	1	1	0.11	0	0	0.00	1	1	0.06	0.4978
Dysarthria	0	0	0.00	0	0	0.00	0	0	0.00	1.0000
Myasthenia	0	0	0.00	1	1	0.11	1	1	0.06	1.0000
Loss of taste	1	1	0.11	0	0	0.00	1	1	0.06	0.4978
Loss of olfaction	1	1	0.11	0	0	0.00	1	1	0.06	0.4978
**Musculoskeletal and connective tissue disorders**	0	0	0.00	0	0	0.00	0	0	0.00	1.0000
Myalgia	0	0	0.00	0	0	0.00	0	0	0.00	1.0000
Arthralgia	0	0	0.00	0	0	0.00	0	0	0.00	1.0000
**Infection and infectious diseases**	6	6	0.67	5	4	0.44	11	10	0.56	0.5460
Nasopharyngitis	6	6	0.67	3	3	0.33	9	9	0.50	0.3405
Influenza	0	0	0.00	2	1	0.11	2	1	0.06	1.0000
**Gastrointestinal disorders**	6	6	0.67	5	4	0.44	11	10	0.56	0.5460
Abdominal pain	0	0	0.00	3	3	0.33	3	3	0.17	0.2496
Epigastric pain	2	2	0.22	1	1	0.11	3	3	0.17	0.6231
Abdominal distension	1	1	0.11	0	0	0.00	1	1	0.06	0.4978
Dry mouth	1	1	0.11	0	0	0.00	1	1	0.06	0.4978
Oral pain	1	1	0.11	0	0	0.00	1	1	0.06	0.4978
Poor digestion	1	1	0.11	0	0	0.00	1	1	0.06	0.4978
Toothache	0	0	0.00	1	1	0.11	1	1	0.06	1.0000
**Renal and urinary disorders**	4	3	0.33	1	1	0.11	5	4	0.22	0.3724
Frequency of urination	1	1	0.11	1	1	0.11	2	2	0.11	1.0000
Polyuria	1	1	0.11	0	0	0.00	1	1	0.06	0.4978
Dysuria	1	1	0.11	0	0	0.00	1	1	0.06	0.4978
Hematuria	1	1	0.11	0	0	0.00	1	1	0.06	0.4978

Adverse Events	Recombinant vaccine group (N=896)			Inactivated vaccine group (N=904)			Total (N=1800)			P Value[1]
	Adverse events	Cases	Incidence (%)	Adverse events	Cases	Incidence (%)	Adverse events	Cases	Incidence (%)	
**Psychiatric category**	3	3	0.33	1	1	0.11	4	4	0.22	0.3724
Insomnia	2	2	0.22	1	1	0.11	3	3	0.17	0.6231
Anxiety	1	1	0.11	0	0	0.00	1	1	0.06	0.4978
**Immune system disease**	0	0	0.00	1	1	0.11	1	1	0.06	1.0000
Hypersensitivity reaction	0	0	0.00	1	1	0.11	1	1	0.06	1.0000
**Various inspections**	1	1	0.11	2	2	0.22	3	3	0.17	1.0000
Elevated blood pressure	1	1	0.11	1	1	0.11	2	2	0.11	1.0000
Heart rate increased	0	0	0.00	1	1	0.11	1	1	0.06	1.0000
**Ocular organ disease**	3	3	0.33	0	0	0.00	3	3	0.17	0.1231
Ocular hyperemia	1	1	0.11	0	0	0.00	1	1	0.06	0.4978
Ocular irritation	1	1	0.11	0	0	0.00	1	1	0.06	0.4978
Eye pruritus	1	1	0.11	0	0	0.00	1	1	0.06	0.4978
**Ear and labyrinthine diseases**	0	0	0.00	0	0	0.00	0	0	0.00	1.0000
Ear pain	0	0	0.00	0	0	0.00	0	0	0.00	1.0000
**Skin and subcutaneous tissue diseases**	1	1	0.11	0	0	0.00	1	1	0.06	0.4978
Erythema	1	1	0.11	0	0	0.00	1	1	0.06	0.4978
**Reproductive system and breast diseases**	1	1	0.11	0	0	0.00	1	1	0.06	0.4978
Erectile dysfunctiom	1	1	0.11	0	0	0.00	1	1	0.06	0.4978

[1]P value was calculated by Fisher exact test.
(1) Unsolicited adverse events were coded using MedDRA version 24.1. and solicited adverse events are listed according to the event name specified in the protocol.
(2) Occurrence time (day) = occurrence date of adverse events-vaccination date of the booster dose
(3) This analysis data is up to January 25, 2022.

Table 1-11 Adverse events in the whole population within 0-7 days related to the study vaccine during the booster period (listed by solicitation and non-solicitation events) (SS)

Adverse events	Recombinant vaccine group (N=896)			Inactivated vaccine group (N=904)			Total (N=1800)			P Value[1]
	Adverse events	Cases	Incidence (%)	Adverse events	Cases	Incidence (%)	Adverse events	Cases	Incidence (%)	
**TEAE**	269	153	17.08	216	123	13.61	485	276	15.33	0.0427

Adverse events	Recombinant vaccine group (N=896)			Inactivated vaccine group (N=904)			Total (N=1800)			P Value[1]
	Adverse events	Cases	Incidence (%)	Adverse events	Cases	Incidence (%)	Adverse events	Cases	Incidence (%)	
**Solicitated adverse events**	215	146	16.29	176	115	12.72	391	261	14.50	0.0323
**Non-injection site (systemic)**	168	111	12.39	131	84	9.29	299	195	10.83	0.0404
Fever	24	24	2.68	22	22	2.43	46	46	2.56	0.7671
Diarrhoea	2	2	0.22	2	2	0.22	4	4	0.22	1.0000
Vomiting	2	2	0.22	1	1	0.11	3	3	0.17	0.6231
Nausea	5	5	0.56	2	2	0.22	7	7	0.39	0.2861
Muscle pain (non-inoculation site)	30	30	3.35	25	25	2.77	55	55	3.06	0.4961
Arthralgia	2	2	0.22	2	2	0.22	4	4	0.22	1.0000
Headache	54	52	5.80	38	38	4.20	92	90	5.00	0.1304
Cough	20	20	2.23	14	13	1.44	34	33	1.83	0.2233
Dyspnea	0	0	0.00	1	1	0.11	1	1	0.06	1.0000
Fatigue/Asthenia	28	27	3.01	22	22	2.43	50	49	2.72	0.4721
Vertigo	1	1	0.11	2	2	0.22	3	3	0.17	1.0000
**Injection site (local)**	47	46	5.13	45	42	4.65	92	88	4.89	0.6629
Pain	42	42	4.69	36	36	3.98	78	78	4.33	0.4888
Swelling	0	0	0.00	1	1	0.11	1	1	0.06	1.0000
Skin rash	0	0	0.00	2	2	0.22	2	2	0.11	0.4997
Flushing	2	2	0.22	0	0	0.00	2	2	0.11	0.2476
Pruritus	3	3	0.33	6	6	0.66	9	9	0.50	0.5068
**Unsolicited adverse events**	54	41	4.58	40	30	3.32	94	71	3.94	0.1840
**Respiratory, thoracic and mediastinal diseases**	19	15	1.67	18	14	1.55	37	29	1.61	0.8538
Cough	0	0	0.00	0	0	0.00	0	0	0.00	1.0000
Runny nose	6	6	0.67	7	7	0.77	13	13	0.72	1.0000
Oropharyngeal Pain	7	7	0.78	4	4	0.44	11	11	0.61	0.3842
Nasal obstruction	5	5	0.56	4	4	0.44	9	9	0.50	0.7523
Difficulty breathing	0	0	0.00	0	0	0.00	0	0	0.00	1.0000
Sneeze	0	0	0.00	2	2	0.22	2	2	0.11	0.4997
Epistaxis	0	0	0.00	1	1	0.11	1	1	0.06	1.0000
allergic coryza	1	1	0.11	0	0	0.00	1	1	0.06	0.4978
**General disorders and administration site conditions**	10	9	1.00	7	6	0.66	17	15	0.83	0.4507
Pain	3	3	0.33	1	1	0.11	4	4	0.22	0.3724
Fever	0	0	0.00	0	0	0.00	0	0	0.00	1.0000
Chest pain	2	2	0.22	4	4	0.44	6	6	0.33	0.6870
Fatigue	0	0	0.00	0	0	0.00	0	0	0.00	1.0000

	Recombinant vaccine group (N=896)			Inactivated vaccine group (N=904)			Total (N=1800)			
**Adverse events**	**Adverse events**	**Cases**	**Incidence (%)**	**Adverse events**	**Cases**	**Incidence (%)**	**Adverse events**	**Cases**	**Incidence (%)**	**P Value[1]**
Feeling of fever	4	4	0.45	0	0	0.00	4	4	0.22	0.0612
Swelling	1	1	0.11	1	1	0.11	2	2	0.11	1.0000
Influenza-like disease	0	0	0.00	1	1	0.11	1	1	0.06	1.0000
**Nervous system disorders**	4	4	0.45	4	4	0.44	8	8	0.44	1.0000
Headache	0	0	0.00	0	0	0.00	0	0	0.00	1.0000
Sleepiness	2	2	0.22	2	2	0.22	4	4	0.22	1.0000
Dizziness	1	1	0.11	1	1	0.11	2	2	0.11	1.0000
Hypoaesthesia	1	1	0.11	0	0	0.00	1	1	0.06	0.4978
Myasthenia	0	0	0.00	1	1	0.11	1	1	0.06	1.0000
	0	0	0.00	0	0	0.00	0	0	0.00	1.0000
**Musculoskeletal and connective tissue disorders**										
Myalgia	0	0	0.00	0	0	0.00	0	0	0.00	1.0000
	6	6	0.67	5	4	0.44	11	10	0.56	0.5460
**Infections and infestations**										
Nasopharyngitis	6	6	0.67	3	3	0.33	9	9	0.50	0.3405
Influenza	0	0	0.00	2	1	0.11	2	1	0.06	1.0000
**Gastrointestinal system diseases**	6	6	0.67	3	3	0.33	9	9	0.50	0.3405
Abdominal pain	0	0	0.00	3	3	0.33	3	3	0.17	0.2496
Epigastric pain	2	2	0.22	0	0	0.00	2	2	0.11	0.2476
Abdominal distension	1	1	0.11	0	0	0.00	1	1	0.06	0.4978
Dry mouth	1	1	0.11	0	0	0.00	1	1	0.06	0.4978
Oral pain	1	1	0.11	0	0	0.00	1	1	0.06	0.4978
Dyspepsia	1	1	0.11	0	0	0.00	1	1	0.06	0.4978
**Psychiatric disorders**	2	2	0.22	1	1	0.11	3	3	0.17	0.6231
Insomnia	2	2	0.22	1	1	0.11	3	3	0.17	0.6231
**Renal and urinary disorders**	3	2	0.22	0	0	0.00	3	2	0.11	0.2476
Polyuria	1	1	0.11	0	0	0.00	1	1	0.06	0.4978
Urinary frequency	1	1	0.11	0	0	0.00	1	1	0.06	0.4978
Dysuria	1	1	0.11	0	0	0.00	1	1	0.06	0.4978
**Eye disorders**	3	3	0.33	0	0	0.00	3	3	0.17	0.1231
Ocular congestion	1	1	0.11	0	0	0.00	1	1	0.06	0.4978
Eye irritation	1	1	0.11	0	0	0.00	1	1	0.06	0.4978
Eye pruritus	1	1	0.11	0	0	0.00	1	1	0.06	0.4978
**Ear and labyrinth disorders**	0	0	0.00	0	0	0.00	0	0	0.00	1.0000
Earache	0	0	0.00	0	0	0.00	0	0	0.00	1.0000
**Various inspections**	0	0	0.00	1	1	0.11	1	1	0.06	1.0000

Adverse events	Recombinant vaccine group (N=896) Adverse events	Cases	Incidence (%)	Inactivated vaccine group (N=904) Adverse events	Cases	Incidence (%)	Total (N=1800) Adverse events	Cases	Incidence (%)	P Value[1]
Elevated heart rate	0	0	0.00	1	1	0.11	1	1	0.06	1.0000
**Immune system disease**	0	0	0.00	1	1	0.11	1	1	0.06	1.0000
Hypersensitivity reaction	0	0	0.00	1	1	0.11	1	1	0.06	1.0000
**Skin and subcutaneous tissue disorders**	1	1	0.11	0	0	0.00	1	1	0.06	0.4978
Erythema	1	1	0.11	0	0	0.00	1	1	0.06	0.4978

[1]P value was calculated by Fisher exact test.
(1) Unsolicited adverse events were coded using MedDRA version 24.1. and solicited adverse events are listed according to the event name specified in the protocol.
(2) Occurrence time (day) = occurrence date of adverse events-vaccination date of the booster dose
(3) Study vaccine-related means that the relationship between adverse events and study vaccine is "definitely related," "most likely related," or " likely related."
(4) This analysis data is up to January 25, 2022.

1.2.2.3. Various kinds of adverse events within 8-30 days

There were 76 cases (4.22%) with 127 adverse events within 8-30 days after the whole population was immunized with a booster dose, among which 42 cases (4.69%) with 70 adverse events occurred in the recombinant vaccine, and 34 cases (3.76%) with 57 adverse events occurred in the inactivated vaccine group. There was no significant difference between the groups. Adverse events related to the study vaccine occurred in 29 cases (1.61%) with 54 adverse events, of which 9 cases (1.00%) with 20 adverse events occurred in the recombinant vaccine group, and 20 cases (2.21%) with 34 adverse events occurred in the inactivated vaccine group. There was no statistically significant difference between the groups.

According to the classification of SOC and PT, the adverse events related to the study vaccines were mainly respiratory, thoracic, and mediastinal disease reactions. There were 4 cases (0.45%) with 7 adverse events in the recombinant vaccine group and 11 cases (1.22%) with 16 in the inactivated vaccine group. There was no significant difference between the groups. The other vaccine-related adverse events include by systemic diseases and various reactions at the administration site (the incidence of recombinant vaccine group and inactivated vaccine group were 0.45% and 1.00%, respectively), and various musculoskeletal and connective tissue diseases (the incidence of recombinant vaccine group and inactivated vaccine group were 0.56% and 0.66%, respectively).

By classification of solicited and unsolicited, the unsolicited adverse events related to

the study vaccines occurred in 9 cases (1.00%) with 20 adverse events in the recombinant vaccine group and 20 cases (2.21%) with 34 adverse events in the inactivated vaccine group. There was no statistically significant difference between the groups. The most common unsolicited adverse reaction was cough (the incidence in the recombinant vaccine group was 1.34%, and in inactivated vaccine group was 1.55%).

See Table 1-12,Table 1-13 and Table 1-14 and Table 1-15 for details.

Table 1-12 Adverse events occurred within 8 - 30 days during the booster period for the whole population (by SOC and PT) (SS)

	Recombinant vaccine group (N=896)			Inactivated vaccine group (N=904)			Total (N=1800)			
**Adverse events**	**Adverse events**	**Cases**	**Incidence (%)**	**Adverse events**	**Cases**	**Incidence (%)**	**Adverse events**	**Cases**	**Incidence (%)**	**P Value[1]**
**TEAE**	70	42	4.69	57	34	3.76	127	76	4.22	0.3499
**General disorders and administration site conditions**	22	18	2.01	15	14	1.55	37	32	1.78	0.4812
Pain at vaccination site	0	0	0.00	0	0	0.00	0	0	0.00	1.0000
Fever	15	15	1.67	8	8	0.88	23	23	1.28	0.1473
Fatigue	6	6	0.67	5	5	0.55	11	11	0.61	0.7728
Chest pain	0	0	0.00	1	1	0.11	1	1	0.06	1.0000
Feeling of fever	0	0	0.00	0	0	0.00	0	0	0.00	1.0000
Pain	0	0	0.00	0	0	0.00	0	0	0.00	1.0000
Itching at vaccination site	1	1	0.11	0	0	0.00	1	1	0.06	0.4978
Vaccination site erythema	0	0	0.00	0	0	0.00	0	0	0.00	1.0000
Swelling	0	0	0.00	0	0	0.00	0	0	0.00	1.0000
Chills	0	0	0.00	0	0	0.00	0	0	0.00	1.0000
Nodule	0	0	0.00	1	1	0.11	1	1	0.06	1.0000
Influenza-like illness	0	0	0.00	0	0	0.00	0	0	0.00	1.0000
Rash at vaccination site	0	0	0.00	0	0	0.00	0	0	0.00	1.0000
Vaccination site swelling	0	0	0.00	0	0	0.00	0	0	0.00	1.0000
**Various nervous system diseases**	9	9	1.00	8	8	0.88	17	17	0.94	0.8129
Headache	6	6	0.67	6	6	0.66	12	12	0.67	1.0000
Dizziness	2	2	0.22	1	1	0.11	3	3	0.17	0.6231
Sleepiness	0	0	0.00	0	0	0.00	0	0	0.00	1.0000
Hyposensation	1	1	0.11	0	0	0.00	1	1	0.06	0.4978
Dysarthria	0	0	0.00	1	1	0.11	1	1	0.06	1.0000
Myasthenia	0	0	0.00	0	0	0.00	0	0	0.00	1.0000
Loss of taste	0	0	0.00	0	0	0.00	0	0	0.00	1.0000

Adverse events	Recombinant vaccine group (N=896)			Inactivated vaccine group (N=904)			Total (N=1800)			P Value[1]
	Adverse events	Cases	Incidence (%)	Adverse events	Cases	Incidence (%)	Adverse events	Cases	Incidence (%)	
Loss of olfaction	0	0	0.00	0	0	0.00	0	0	0.00	1.0000
**Respiratory, thoracic and mediastinal disorders**	20	14	1.56	25	19	2.10	45	33	1.83	0.4829
Coughing	12	12	1.34	14	14	1.55	26	26	1.44	0.8439
Runny nose	4	4	0.45	3	3	0.33	7	7	0.39	0.7249
Oropharyngeal pain	3	3	0.33	3	3	0.33	6	6	0.33	1.0000
Nasal congestion	0	0	0.00	3	3	0.33	3	3	0.17	0.2496
Dyspnea	1	1	0.11	2	2	0.22	3	3	0.17	1.0000
Sneezing	0	0	0.00	0	0	0.00	0	0	0.00	1.0000
Epistaxis	0	0	0.00	0	0	0.00	0	0	0.00	1.0000
Allergic coryza	0	0	0.00	0	0	0.00	0	0	0.00	1.0000
Hemoptysis	0	0	0.00	0	0	0.00	0	0	0.00	1.0000
**Musculoskeletal and connective tissue disorders**	14	13	1.45	9	9	1.00	23	22	1.22	0.4001
Myalgia	14	13	1.45	8	8	0.88	22	21	1.17	0.2815
Arthralgia	0	0	0.00	1	1	0.11	1	1	0.06	1.0000
**Gastrointestinal system diseases**	0	0	0.00	0	0	0.00	0	0	0.00	1.0000
Nausea	0	0	0.00	0	0	0.00	0	0	0.00	1.0000
Diarrhea	0	0	0.00	0	0	0.00	0	0	0.00	1.0000
Abdominal pain	0	0	0.00	0	0	0.00	0	0	0.00	1.0000
Vomiting	0	0	0.00	0	0	0.00	0	0	0.00	1.0000
Epigastric pain	0	0	0.00	0	0	0.00	0	0	0.00	1.0000
Dysphagia	0	0	0.00	0	0	0.00	0	0	0.00	1.0000
Abdominal distension	0	0	0.00	0	0	0.00	0	0	0.00	1.0000
Dry mouth	0	0	0.00	0	0	0.00	0	0	0.00	1.0000
Oral pain	0	0	0.00	0	0	0.00	0	0	0.00	1.0000
Poor digestion	0	0	0.00	0	0	0.00	0	0	0.00	1.0000
Toothache	0	0	0.00	0	0	0.00	0	0	0.00	1.0000
**Infections and infestations**	1	1	0.11	0	0	0.00	1	1	0.06	0.4978
Nasopharyngitis	1	1	0.11	0	0	0.00	1	1	0.06	0.4978
Influenza	0	0	0.00	0	0	0.00	0	0	0.00	1.0000
**Skin and subcutaneous tissue diseases**	0	0	0.00	0	0	0.00	0	0	0.00	1.0000
Pruritus	0	0	0.00	0	0	0.00	0	0	0.00	1.0000
Erythema	0	0	0.00	0	0	0.00	0	0	0.00	1.0000
Rash	0	0	0.00	0	0	0.00	0	0	0.00	1.0000
**Psychiatric category**	0	0	0.00	0	0	0.00	0	0	0.00	1.0000
Insomnia	0	0	0.00	0	0	0.00	0	0	0.00	1.0000
Anxiety	0	0	0.00	0	0	0.00	0	0	0.00	1.0000

Adverse events	Recombinant vaccine group (N=896)			Inactivated vaccine group (N=904)			Total (N=1800)			P Value[1]
	Adverse events	Cases	Incidence (%)	Adverse events	Cases	Incidence (%)	Adverse events	Cases	Incidence (%)	
**Immune system disease**	3	3	0.33	0	0	0.00	3	3	0.17	0.1231
Hypersensitivity reaction	3	3	0.33	0	0	0.00	3	3	0.17	0.1231
**Renal and urinary disorders**	0	0	0.00	0	0	0.00	0	0	0.00	1.0000
Frequency of urination	0	0	0.00	0	0	0.00	0	0	0.00	1.0000
Polyuria	0	0	0.00	0	0	0.00	0	0	0.00	1.0000
Dysuria	0	0	0.00	0	0	0.00	0	0	0.00	1.0000
Hematuria	0	0	0.00	0	0	0.00	0	0	0.00	1.0000
**Various inspections**	0	0	0.00	0	0	0.00	0	0	0.00	1.0000
Blood pressure increased	0	0	0.00	0	0	0.00	0	0	0.00	1.0000
Elevated heart rate	0	0	0.00	0	0	0.00	0	0	0.00	1.0000
**Eye disorders**	0	0	0.00	0	0	0.00	0	0	0.00	1.0000
Ocular congestion	0	0	0.00	0	0	0.00	0	0	0.00	1.0000
Eye irritation	0	0	0.00	0	0	0.00	0	0	0.00	1.0000
Eye pruritus	0	0	0.00	0	0	0.00	0	0	0.00	1.0000
**Ear and labyrinth disorders**	1	1	0.11	0	0	0.00	1	1	0.06	0.4978
Earache	1	1	0.11	0	0	0.00	1	1	0.06	0.4978
**Reproductive system and breast disorders**	0	0	0.00	0	0	0.00	0	0	0.00	1.0000
Erectile dysfunctiom	0	0	0.00	0	0	0.00	0	0	0.00	1.0000

[1]P value was calculated by Fisher exact test.
(1) Adverse events were coded using MedDRA version 24.1.
(2) Occurrence time (day) = occurrence date of adverse events-vaccination date of the booster dose
(3) This analysis data is up to January 25, 2022.

Table 1-13 Adverse events occurred within 8-30 days related to the study vaccine during the booster period for the whole population (by SOC and PT) (SS)

Adverse events	Recombinant vaccine group (N=896)			Inactivated vaccine group (N=904)			Total (N=1800)			P Value[1]
	Adverse events	Cases	Incidence (%)	Adverse events	Cases	Incidence (%)	Adverse events	Cases	Incidence (%)	
**TEAE**	20	9	1.00	34	20	2.21	54	29	1.61	0.0594
**General disorders and administration site conditions**	5	4	0.45	9	9	1.00	14	13	0.72	0.2652

Adverse events	Recombinant vaccine group (N=896)			Inactivated vaccine group (N=904)			Total (N=1800)			P Value[1]
	Adverse events	Cases	Incidence (%)	Adverse events	Cases	Incidence (%)	Adverse events	Cases	Incidence (%)	
Pain at vaccination site	0	0	0.00	0	0	0.00	0	0	0.00	1.0000
Fatigue	1	1	0.11	4	4	0.44	5	5	0.28	0.3743
Fever	4	4	0.45	5	5	0.55	9	9	0.50	1.0000
Chest pain	0	0	0.00	0	0	0.00	0	0	0.00	1.0000
Feeling of fever	0	0	0.00	0	0	0.00	0	0	0.00	1.0000
Pain	0	0	0.00	0	0	0.00	0	0	0.00	1.0000
Itching at vaccination site	0	0	0.00	0	0	0.00	0	0	0.00	1.0000
Vaccination site erythema	0	0	0.00	0	0	0.00	0	0	0.00	1.0000
Swelling	0	0	0.00	0	0	0.00	0	0	0.00	1.0000
Influenza-like illness	0	0	0.00	0	0	0.00	0	0	0.00	1.0000
Rash at vaccination site	0	0	0.00	0	0	0.00	0	0	0.00	1.0000
Vaccination site swelling	0	0	0.00	0	0	0.00	0	0	0.00	1.0000
**Various nervous system diseases**	1	1	0.11	3	3	0.33	4	4	0.22	0.6246
Headache	0	0	0.00	3	3	0.33	3	3	0.17	0.2496
Dizziness	1	1	0.11	0	0	0.00	1	1	0.06	0.4978
Sleepiness	0	0	0.00	0	0	0.00	0	0	0.00	1.0000
Hyposensation	0	0	0.00	0	0	0.00	0	0	0.00	1.0000
Myasthenia	0	0	0.00	0	0	0.00	0	0	0.00	1.0000
**Musculoskeletal and connective tissue disorders**	6	5	0.56	6	6	0.66	12	11	0.61	1.0000
Myalgia	6	5	0.56	6	6	0.66	12	11	0.61	1.0000
Arthralgia	0	0	0.00	0	0	0.00	0	0	0.00	1.0000
**Respiratory, thoracic and mediastinal disorders**	7	4	0.45	16	11	1.22	23	15	0.83	0.1170
Coughing	3	3	0.33	8	8	0.88	11	11	0.61	0.2252
Runny nose	2	2	0.22	2	2	0.22	4	4	0.22	1.0000
Oropharyngeal pain	1	1	0.11	2	2	0.22	3	3	0.17	1.0000
Nasal congestion	0	0	0.00	2	2	0.22	2	2	0.11	0.4997
Dyspnea	1	1	0.11	2	2	0.22	3	3	0.17	1.0000
Sneezing	0	0	0.00	0	0	0.00	0	0	0.00	1.0000
Epistaxis	0	0	0.00	0	0	0.00	0	0	0.00	1.0000
Allergic coryza	0	0	0.00	0	0	0.00	0	0	0.00	1.0000
**Gastrointestinal system diseases**	0	0	0.00	0	0	0.00	0	0	0.00	1.0000
Nausea	0	0	0.00	0	0	0.00	0	0	0.00	1.0000
Diarrhea	0	0	0.00	0	0	0.00	0	0	0.00	1.0000
Abdominal pain	0	0	0.00	0	0	0.00	0	0	0.00	1.0000

Adverse events	Recombinant vaccine group (N=896)			Inactivated vaccine group (N=904)			Total (N=1800)			P Value[1]
	Adverse events	Cases	Incidence (%)	Adverse events	Cases	Incidence (%)	Adverse events	Cases	Incidence (%)	
Vomiting	0	0	0.00	0	0	0.00	0	0	0.00	1.0000
Epigastric pain	0	0	0.00	0	0	0.00	0	0	0.00	1.0000
Abdominal distension	0	0	0.00	0	0	0.00	0	0	0.00	1.0000
Dry mouth	0	0	0.00	0	0	0.00	0	0	0.00	1.0000
Oral pain	0	0	0.00	0	0	0.00	0	0	0.00	1.0000
Poor digestion	0	0	0.00	0	0	0.00	0	0	0.00	1.0000
**Infections and infestations**	0	0	0.00	0	0	0.00	0	0	0.00	1.0000
Nasopharyngitis	0	0	0.00	0	0	0.00	0	0	0.00	1.0000
Influenza	0	0	0.00	0	0	0.00	0	0	0.00	1.0000
**Skin and subcutaneous tissue diseases**	0	0	0.00	0	0	0.00	0	0	0.00	1.0000
Pruritus	0	0	0.00	0	0	0.00	0	0	0.00	1.0000
Erythema	0	0	0.00	0	0	0.00	0	0	0.00	1.0000
Rash	0	0	0.00	0	0	0.00	0	0	0.00	1.0000
**Psychiatric category**	0	0	0.00	0	0	0.00	0	0	0.00	1.0000
Insomnia	0	0	0.00	0	0	0.00	0	0	0.00	1.0000
**Eye disorders**	0	0	0.00	0	0	0.00	0	0	0.00	1.0000
Ocular congestion	0	0	0.00	0	0	0.00	0	0	0.00	1.0000
Eye irritation	0	0	0.00	0	0	0.00	0	0	0.00	1.0000
Eye pruritus	0	0	0.00	0	0	0.00	0	0	0.00	1.0000
**Renal and urinary disorders**	0	0	0.00	0	0	0.00	0	0	0.00	1.0000
Polyuria	0	0	0.00	0	0	0.00	0	0	0.00	1.0000
Frequency of urination	0	0	0.00	0	0	0.00	0	0	0.00	1.0000
Dysuria	0	0	0.00	0	0	0.00	0	0	0.00	1.0000
**Ear and labyrinth disorders**	1	1	0.11	0	0	0.00	1	1	0.06	0.4978
Earache	1	1	0.11	0	0	0.00	1	1	0.06	0.4978
**Various inspections**	0	0	0.00	0	0	0.00	0	0	0.00	1.0000
Elevated heart rate	0	0	0.00	0	0	0.00	0	0	0.00	1.0000
**Immune system disease**	0	0	0.00	0	0	0.00	0	0	0.00	1.0000
Hypersensitivity reaction	0	0	0.00	0	0	0.00	0	0	0.00	1.0000

[1]P value was calculated by Fisher exact test.

(1) Adverse events were coded using MedDRA version 24.1.

(2) Occurrence time (day) = occurrence date of adverse events-vaccination date of the booster dose

(3) Study vaccine-related means that the relationship between adverse events and study vaccine is "definitely related," "most likely related," or " likely related."

(4) This analysis data is up to January 25, 2022.

Table 1-14 Adverse events occurred within 8-30 days during the booster period for the whole population (solicited and unsolicited) (SS)

	Recombinant vaccine group (N=896)			Inactivated vaccine group (N=904)			Total (N=1800)			
**Adverse events**	**Adverse events**	**Cases**	**Incidence (%)**	**Adverse events**	**Cases**	**Incidence (%)**	**Adverse events**	**Cases**	**Incidence (%)**	**P Value[1]**
**TEAE**	70	42	4.69	57	34	3.76	127	76	4.22	0.3499
**Solicited adverse events**	0	0	0.00	0	0	0.00	0	0	0.00	1.0000
**Non-injection site (systemic)**	0	0	0.00	0	0	0.00	0	0	0.00	1.0000
Fever	0	0	0.00	0	0	0.00	0	0	0.00	1.0000
Diarrhoea	0	0	0.00	0	0	0.00	0	0	0.00	1.0000
Dysphagia	0	0	0.00	0	0	0.00	0	0	0.00	1.0000
Vomiting	0	0	0.00	0	0	0.00	0	0	0.00	1.0000
Nausea	0	0	0.00	0	0	0.00	0	0	0.00	1.0000
Muscle pain (non-inoculation site)	0	0	0.00	0	0	0.00	0	0	0.00	1.0000
Arthralgia	0	0	0.00	0	0	0.00	0	0	0.00	1.0000
Headache	0	0	0.00	0	0	0.00	0	0	0.00	1.0000
Cough	0	0	0.00	0	0	0.00	0	0	0.00	1.0000
Dyspnea	0	0	0.00	0	0	0.00	0	0	0.00	1.0000
Fatigue/Asthenia	0	0	0.00	0	0	0.00	0	0	0.00	1.0000
Vertigo	0	0	0.00	0	0	0.00	0	0	0.00	1.0000
**Injection site (local)**	0	0	0.00	0	0	0.00	0	0	0.00	1.0000
Pain	0	0	0.00	0	0	0.00	0	0	0.00	1.0000
Swelling	0	0	0.00	0	0	0.00	0	0	0.00	1.0000
Skin rash	0	0	0.00	0	0	0.00	0	0	0.00	1.0000
Flushing	0	0	0.00	0	0	0.00	0	0	0.00	1.0000
Pruritus	0	0	0.00	0	0	0.00	0	0	0.00	1.0000
**Unsolicited adverse events**	70	42	4.69	57	34	3.76	127	76	4.22	0.3499
**Respiratory, thoracic and mediastinal diseases**	20	14	1.56	25	19	2.10	45	33	1.83	0.4829
Cough	12	12	1.34	14	14	1.55	26	26	1.44	0.8439
Runny nose	4	4	0.45	3	3	0.33	7	7	0.39	0.7249
Oropharyngeal pain	3	3	0.33	3	3	0.33	6	6	0.33	1.0000
Nasal obstruction	0	0	0.00	3	3	0.33	3	3	0.17	0.2496
Difficulty breathing	1	1	0.11	2	2	0.22	3	3	0.17	1.0000
Sneeze	0	0	0.00	0	0	0.00	0	0	0.00	1.0000
Epistaxis	0	0	0.00	0	0	0.00	0	0	0.00	1.0000
Allergic coryza	0	0	0.00	0	0	0.00	0	0	0.00	1.0000
hemoptysis	0	0	0.00	0	0	0.00	0	0	0.00	1.0000

Adverse events	Recombinant vaccine group (N=896)			Inactivated vaccine group (N=904)			Total (N=1800)			P Value[1]
	Adverse events	Cases	Incidence (%)	Adverse events	Cases	Incidence (%)	Adverse events	Cases	Incidence (%)	
**General disorders and administration site conditions**	22	18	2.01	15	14	1.55	37	32	1.78	0.4812
Pain	0	0	0.00	0	0	0.00	0	0	0.00	1.0000
Fever	15	15	1.67	8	8	0.88	23	23	1.28	0.1473
**General disorders and administration site conditions**	6	6	0.67	5	5	0.55	11	11	0.61	0.7728
Pain	0	0	0.00	1	1	0.11	1	1	0.06	1.0000
Feeling of fever	0	0	0.00	0	0	0.00	0	0	0.00	1.0000
Swelling	0	0	0.00	0	0	0.00	0	0	0.00	1.0000
Chills	0	0	0.00	0	0	0.00	0	0	0.00	1.0000
Nodule	0	0	0.00	1	1	0.11	1	1	0.06	1.0000
Influenza-like illness	0	0	0.00	0	0	0.00	0	0	0.00	1.0000
Itching at vaccination site	1	1	0.11	0	0	0.00	1	1	0.06	0.4978
**Various nervous system diseases**	9	9	1.00	8	8	0.88	17	17	0.94	0.8129
Headache	6	6	0.67	6	6	0.66	12	12	0.67	1.0000
Sleepiness	0	0	0.00	0	0	0.00	0	0	0.00	1.0000
Dizziness	2	2	0.22	1	1	0.11	3	3	0.17	0.6231
Hyposensation	1	1	0.11	0	0	0.00	1	1	0.06	0.4978
Dysarthria	0	0	0.00	1	1	0.11	1	1	0.06	1.0000
Myasthenia	0	0	0.00	0	0	0.00	0	0	0.00	1.0000
Loss of taste	0	0	0.00	0	0	0.00	0	0	0.00	1.0000
Loss of olfaction	0	0	0.00	0	0	0.00	0	0	0.00	1.0000
**Musculoskeletal and connective tissue disorders**	14	13	1.45	9	9	1.00	23	22	1.22	0.4001
Myalgia	14	13	1.45	8	8	0.88	22	21	1.17	0.2815
Arthralgia	0	0	0.00	1	1	0.11	1	1	0.06	1.0000
**Infections and infestations**	1	1	0.11	0	0	0.00	1	1	0.06	0.4978
Nasopharyngitis	1	1	0.11	0	0	0.00	1	1	0.06	0.4978
Influenza	0	0	0.00	0	0	0.00	0	0	0.00	1.0000
**Gastrointestinal system diseases**	0	0	0.00	0	0	0.00	0	0	0.00	1.0000
Abdominal pain	0	0	0.00	0	0	0.00	0	0	0.00	1.0000
Epigastric pain	0	0	0.00	0	0	0.00	0	0	0.00	1.0000
Abdominal distension	0	0	0.00	0	0	0.00	0	0	0.00	1.0000
Dry mouth	0	0	0.00	0	0	0.00	0	0	0.00	1.0000
Oral pain	0	0	0.00	0	0	0.00	0	0	0.00	1.0000
Poor digestion	0	0	0.00	0	0	0.00	0	0	0.00	1.0000
Toothache	0	0	0.00	0	0	0.00	0	0	0.00	1.0000

Adverse events	Recombinant vaccine group (N=896)			Inactivated vaccine group (N=904)			Total (N=1800)			P Value[1]
	Adverse events	Cases	Incidence (%)	Adverse events	Cases	Incidence (%)	Adverse events	Cases	Incidence (%)	
**Renal and urinary disorders**	0	0	0.00	0	0	0.00	0	0	0.00	1.0000
Frequency of urination	0	0	0.00	0	0	0.00	0	0	0.00	1.0000
Polyuria	0	0	0.00	0	0	0.00	0	0	0.00	1.0000
Dysuria	0	0	0.00	0	0	0.00	0	0	0.00	1.0000
Hematuria	0	0	0.00	0	0	0.00	0	0	0.00	1.0000
**Psychiatric category**	0	0	0.00	0	0	0.00	0	0	0.00	1.0000
Insomnia	0	0	0.00	0	0	0.00	0	0	0.00	1.0000
Anxiety	0	0	0.00	0	0	0.00	0	0	0.00	1.0000
**Immune system disease**	3	3	0.33	0	0	0.00	3	3	0.17	0.1231
Hypersensitivity reaction	3	3	0.33	0	0	0.00	3	3	0.17	0.1231
**Various inspections**	0	0	0.00	0	0	0.00	0	0	0.00	1.0000
Blood pressure increased	0	0	0.00	0	0	0.00	0	0	0.00	1.0000
Elevated heart rate	0	0	0.00	0	0	0.00	0	0	0.00	1.0000
**Eye disorders**	0	0	0.00	0	0	0.00	0	0	0.00	1.0000
Ocular congestion	0	0	0.00	0	0	0.00	0	0	0.00	1.0000
Eye irritation	0	0	0.00	0	0	0.00	0	0	0.00	1.0000
Eye Pruritus	0	0	0.00	0	0	0.00	0	0	0.00	1.0000
**Ear and labyrinth disorders**	1	1	0.11	0	0	0.00	1	1	0.06	0.4978
Earache	1	1	0.11	0	0	0.00	1	1	0.06	0.4978
**Skin and subcutaneous tissue disorders**	0	0	0.00	0	0	0.00	0	0	0.00	1.0000
Erythema	0	0	0.00	0	0	0.00	0	0	0.00	1.0000
**Reproductive system and breast disorders**	0	0	0.00	0	0	0.00	0	0	0.00	1.0000
Erectile dysfunctiom	0	0	0.00	0	0	0.00	0	0	0.00	1.0000

[1]P value was calculated by Fisher exact test.
(1) Unsolicited adverse events were coded using MedDRA version 24.1. and solicited adverse events are listed according to the event name specified in the protocol.
(2) Occurrence time (day) = occurrence date of adverse events-vaccination date of the booster dose
(3) This analysis data is up to January 25, 2022.

Table 1-15 Adverse events related to the study vaccine occurred within 8-30 days during the booster period for the whole population (listed by solicited and unsolicited events) (SS)

Adverse events	Recombinant vaccine group (N=896)			Inactivated vaccine group (N=904)			Total (N=1800)			P Value[1]
	Adverse events	Cases	Incidence (%)	Adverse events	Cases	Incidence (%)	Adverse events	Cases	Incidence (%)	
**TEAE**	20	9	1.00	34	20	2.21	54	29	1.61	0.0594
**Solicited adverse events**	0	0	0.00	0	0	0.00	0	0	0.00	1.0000
**Non-injection site (systemic)**	0	0	0.00	0	0	0.00	0	0	0.00	1.0000
Fever	0	0	0.00	0	0	0.00	0	0	0.00	1.0000
Diarrhoea	0	0	0.00	0	0	0.00	0	0	0.00	1.0000
Vomiting	0	0	0.00	0	0	0.00	0	0	0.00	1.0000
Nausea	0	0	0.00	0	0	0.00	0	0	0.00	1.0000
Muscle pain (non-inoculation site)	0	0	0.00	0	0	0.00	0	0	0.00	1.0000
Arthralgia	0	0	0.00	0	0	0.00	0	0	0.00	1.0000
Headache	0	0	0.00	0	0	0.00	0	0	0.00	1.0000
Cough	0	0	0.00	0	0	0.00	0	0	0.00	1.0000
Dyspnea	0	0	0.00	0	0	0.00	0	0	0.00	1.0000
Fatigue/Asthenia	0	0	0.00	0	0	0.00	0	0	0.00	1.0000
Vertigo	0	0	0.00	0	0	0.00	0	0	0.00	1.0000
**Injection site (local)**	0	0	0.00	0	0	0.00	0	0	0.00	1.0000
Pain	0	0	0.00	0	0	0.00	0	0	0.00	1.0000
Swelling	0	0	0.00	0	0	0.00	0	0	0.00	1.0000
Skin rash	0	0	0.00	0	0	0.00	0	0	0.00	1.0000
Flushing	0	0	0.00	0	0	0.00	0	0	0.00	1.0000
Pruritus	0	0	0.00	0	0	0.00	0	0	0.00	1.0000
**Unsolicited adverse events**	20	9	1.00	34	20	2.21	54	29	1.61	0.0594
**Respiratory, thoracic and mediastinal diseases**	7	4	0.45	16	11	1.22	23	15	0.83	0.1170
Cough	3	3	0.33	8	8	0.88	11	11	0.61	0.2252
Runny nose	2	2	0.22	2	2	0.22	4	4	0.22	1.0000
Oropharyngeal pain	1	1	0.11	2	2	0.22	3	3	0.17	1.0000
Nasal obstruction	0	0	0.00	2	2	0.22	2	2	0.11	0.4997
Difficulty breathing	1	1	0.11	2	2	0.22	3	3	0.17	1.0000
Sneeze	0	0	0.00	0	0	0.00	0	0	0.00	1.0000
Epistaxis	0	0	0.00	0	0	0.00	0	0	0.00	1.0000
Allergic coryza	0	0	0.00	0	0	0.00	0	0	0.00	1.0000
**General disorders and administration site conditions**	5	4	0.45	9	9	1.00	14	13	0.72	0.2652
Pain	0	0	0.00	0	0	0.00	0	0	0.00	1.0000
Fever	4	4	0.45	5	5	0.55	9	9	0.50	1.0000
Chest pain	0	0	0.00	0	0	0.00	0	0	0.00	1.0000

Adverse events	Recombinant vaccine group (N=896)			Inactivated vaccine group (N=904)			Total (N=1800)			P Value[1]
	Adverse events	Cases	Incidence (%)	Adverse events	Cases	Incidence (%)	Adverse events	Cases	Incidence (%)	
Fatigue	1	1	0.11	4	4	0.44	5	5	0.28	0.3743
Feeling of fever	0	0	0.00	0	0	0.00	0	0	0.00	1.0000
Swelling	0	0	0.00	0	0	0.00	0	0	0.00	1.0000
Influenza-like disease	0	0	0.00	0	0	0.00	0	0	0.00	1.0000
**Nervous system disorders**	1	1	0.11	3	3	0.33	4	4	0.22	0.6246
Headache	0	0	0.00	3	3	0.33	3	3	0.17	0.2496
Sleepiness	0	0	0.00	0	0	0.00	0	0	0.00	1.0000
Dizziness	1	1	0.11	0	0	0.00	1	1	0.06	0.4978
Hypoaesthesia	0	0	0.00	0	0	0.00	0	0	0.00	1.0000
Myasthenia	0	0	0.00	0	0	0.00	0	0	0.00	1.0000
**Musculoskeletal and connective tissue disorders**	6	5	0.56	6	6	0.66	12	11	0.61	1.0000
Myalgia	6	5	0.56	6	6	0.66	12	11	0.61	1.0000
**Infections and infestations**	0	0	0.00	0	0	0.00	0	0	0.00	1.0000
Nasopharyngitis	0	0	0.00	0	0	0.00	0	0	0.00	1.0000
Influenza	0	0	0.00	0	0	0.00	0	0	0.00	1.0000
**Gastrointestinal system diseases**	0	0	0.00	0	0	0.00	0	0	0.00	1.0000
Abdominal pain	0	0	0.00	0	0	0.00	0	0	0.00	1.0000
Epigastric pain	0	0	0.00	0	0	0.00	0	0	0.00	1.0000
Abdominal distension	0	0	0.00	0	0	0.00	0	0	0.00	1.0000
Dry mouth	0	0	0.00	0	0	0.00	0	0	0.00	1.0000
Oral pain	0	0	0.00	0	0	0.00	0	0	0.00	1.0000
Dyspepsia	0	0	0.00	0	0	0.00	0	0	0.00	1.0000
**Psychiatric disorders**	0	0	0.00	0	0	0.00	0	0	0.00	1.0000
Insomnia	0	0	0.00	0	0	0.00	0	0	0.00	1.0000
**Renal and urinary disorders**	0	0	0.00	0	0	0.00	0	0	0.00	1.0000
Polyuria	0	0	0.00	0	0	0.00	0	0	0.00	1.0000
Frequency of urination	0	0	0.00	0	0	0.00	0	0	0.00	1.0000
Dysuria	0	0	0.00	0	0	0.00	0	0	0.00	1.0000
**Eye disorders**	0	0	0.00	0	0	0.00	0	0	0.00	1.0000
Ocular congestion	0	0	0.00	0	0	0.00	0	0	0.00	1.0000
Eye irritation	0	0	0.00	0	0	0.00	0	0	0.00	1.0000
Eye puritus	0	0	0.00	0	0	0.00	0	0	0.00	1.0000
**Ear and labyrinth disorders**	1	1	0.11	0	0	0.00	1	1	0.06	0.4978
Earache	1	1	0.11	0	0	0.00	1	1	0.06	0.4978
**Various inspections**	0	0	0.00	0	0	0.00	0	0	0.00	1.0000

Adverse events	Recombinant vaccine group (N=896) Adverse events	Cases	Incidence (%)	Inactivated vaccine group (N=904) Adverse events	Cases	Incidence (%)	Total (N=1800) Adverse events	Cases	Incidence (%)	P Value[1]
Heart rate increased	0	0	0.00	0	0	0.00	0	0	0.00	1.0000
**Immune system disease**	0	0	0.00	0	0	0.00	0	0	0.00	1.0000
Hypersensitivity reaction	0	0	0.00	0	0	0.00	0	0	0.00	1.0000
**Skin and subcutaneous tissue diseases**	0	0	0.00	0	0	0.00	0	0	0.00	1.0000
Erythema	0	0	0.00	0	0	0.00	0	0	0.00	1.0000

[1]P value was calculated by Fisher exact test.
(1) Unsolicited adverse events were coded using MedDRA version 24.1. and solicited adverse events are listed according to the event name specified in the protocol.
(2) Occurrence time (day) = occurrence date of adverse events-vaccination date of the booster dose
(3) Study vaccine-related means that the relationship between adverse events and study vaccine is "definitely related," "most likely related," or " likely related."
(4) This analysis data is up to January 25, 2022.

1.2.3 Solicited and unsolicited adverse events

There were 146 cases (16.29%) with 215 solicitation adverse events in the test group (i.e., the recombinant vaccine group) and 115 cases (12.72%) with 176 adverse events in the control group (i.e., the inactivated group). The difference between groups was statistically significant (*P*=0.0323). Among them, the incidence of systemic adverse events related to the test group and in the control group was 12.39% and 9.29%, respectively. The difference between groups was statistically significant (*P*=0.0404). The incidence of local adverse events related to the test group was 5.13% and 4.65% in the control group, and there was no significant difference between groups. The incidence of non-solicitation adverse events related to the test group was 5.36% in the test group and 5.31% in the control group, respectively, and there was no significant difference between the groups. See Table 1-16 for details.

Table 1-16 The incidence of adverse events in all subjects during the booster period (Listed in terms of solicitation and non-solicitation)（SS）

Name of Adverse event	Recombinant Vaccine Group (N=896) Adverse events	Cases	Incidence (%)	Inactivated Vaccine Group(N=904) Adverse events	Cases	Incidence (%)	Total (N=1800) Adverse events	Cases	Incidence (%)	P Value[1]
Solicited Adverse Events	220	146	16.29	180	118	13.05	400	264	14.67	0.0536

Name of Adverse event	Recombinant Vaccine Group (N=896)			Inactivated Vaccine Group(N=904)			Total (N=1800)			
	Adverse events	Cases	Incidence (%)	Adverse events	Cases	Incidence (%)	Adverse events	Cases	Incidence (%)	P Value[1]
Releated	215	146	16.29	176	115	12.72	391	261	14.50	0.0323
Systemic adverse events	173	111	12.39	135	87	9.62	308	198	11.00	0.0704
Releated	168	111	12.39	131	84	9.29	299	195	10.83	0.0404
Local adverse events	47	46	5.13	45	42	4.65	92	88	4.89	0.6629
Releated	47	46	5.13	45	42	4.65	92	88	4.89	0.6629
Unsolicited adverse events	133	81	9.04	102	66	7.30	235	147	8.17	0.1967
Releated	74	48	5.36	74	48	5.31	148	96	5.33	1.0000

[1] P value was calculated using Fisher's exact probability method.

(1)Non-solicitation adverse events are coded by MedDRA version 24.1, and solicitation adverse events are listed according to the event names specified in the plan.

(2) This analysis data is up to January 25, 2022.

1.2.3.1 Solicited Adverse Events

Systemic Adverse Events Related to the test vaccines:

After booster vaccination, the most common systemic adverse reaction related to the test vaccines (systemic adverse reaction) was headache, for which there are 52 cases (5.80%) with 54 times occurring in the test group (i.e., the recombinant vaccine group), and 38 cases (4.20%) with 38 times occurred in control group (i.e., the inactivated vaccine group). The other frequently occurred systemic adverse reactions include muscle pain (non-vaccination site) (with an incidence rate of 3.35% in the test group and 2.77% in the control group) fatigue/asthenia (with an incidence rate of 3.01% in the test group and 2.43% in the control group), fever (with an incidence rate of 2.68% in the test group and 2.43% in the control group), and cough (twith an incidence rate of 2.23% in the test group and 1.44% in the control group).

The severity of the above systemic adverse reactions was mainly grade 1 or grade 2. In the recombinant vaccine group, fever ofgrade 3 occurred in 3 cases with 3 times while muscle pain with grade 3 occurred in 1 case with 1 time (non-vaccination site); In the inactivated vaccine group, fever of grade 3 occurred in 2 cases with 2 times while muscle pain with grade 3 occurred in 1 case with 1 time (non-vaccination site). There was no significant difference among all kinds of systemic adverse reactions. See Table 1-17， Table 1-18 for details.

Table 1-17 Systemic adverse events related to test vaccine in all subjects during the booster period (SS)

Name of Adverse event	Recombinant Vaccine group (N=896)			Inactivated vaccine group(N=904)			Total (N=1800)			P Value[1]
	Adverse events	Cases	Incidence (%)	Adverse events	Cases	Incidence (%)	Adverse events	Cases	Incidence (%)	
Systemic Adverse Events Related to test vaccine	168	111	12.39	131	84	9.29	299	195	10.83	0.0404
Fever	24	24	2.68	22	22	2.43	46	46	2.56	0.7671
Diarrhea	2	2	0.22	2	2	0.22	4	4	0.22	1.0000
Constipation	0	0	0.00	0	0	0.00	0	0	0.00	1.0000
Dysphagia	0	0	0.00	0	0	0.00	0	0	0.00	1.0000
Anorexia	0	0	0.00	0	0	0.00	0	0	0.00	1.0000
Vomiting	2	2	0.22	1	1	0.11	3	3	0.17	0.6231
Nausea	5	5	0.56	2	2	0.22	7	7	0.39	0.2861
Muscle pain (non-inoculation site)	30	30	3.35	25	25	2.77	55	55	3.06	0.4961
Arthralgia	2	2	0.22	2	2	0.22	4	4	0.22	1.0000
Headache	54	52	5.80	38	38	4.20	92	90	5.00	0.1304
Cough	20	20	2.23	14	13	1.44	34	33	1.83	0.2233
Dyspnea	0	0	0.00	1	1	0.11	1	1	0.06	1.0000
Non-injection site pruritus (no skin lesions)	0	0	0.00	0	0	0.00	0	0	0.00	1.0000
Abnormal skin and mucosa	0	0	0.00	0	0	0.00	0	0	0.00	1.0000
Acute allergic reaction	0	0	0.00	0	0	0.00	0	0	0.00	1.0000
Fatigue/asthenia	28	27	3.01	22	22	2.43	50	49	2.72	0.4721
Vertigo	1	1	0.11	2	2	0.22	3	3	0.17	1.0000

[1]P value was calculated by Fisher exact test.

(1) Unsolicited adverse events are coded using MedDRA version 24.1, and solicited adverse events are listed according to the event name specified in the protocol.

(2) Vaccine-related means that the relationship between adverse events and vaccine-related is "definitely related," "most likely related," or "likely related."

(3) This analysis data is up to January 25, 2022.

Table 1-18 Distribution of severity of systemic adverse events related to test vaccine in all subjects during the booster period (SS)

Adverse Event Name<br>Severity	Recombinant Vaccine group(N=896)			Inactivated vaccine (N=904)			Total (N=1800)			P Value[1]
	**Adverse events**	**Cases**	**Incidence (%)**	**Adverse events**	**Cases**	**Incidence (%)**	**Adverse events**	**Cases**	**Incidence (%)**	
Systemic Adverse Events Related to Test Vaccine	168	111	12.39	131	84	9.29	299	195	10.83	0.0404
Grade 1	110	89	9.93	88	65	7.19	198	154	8.56	0.0429
Grade 2	54	39	4.35	40	30	3.32	94	69	3.83	0.2708
Grade 3	4	4	0.45	3	2	0.22	7	6	0.33	0.4504
Grade 4	0	0	0.00	0	0	0.00	0	0	0.00	1.0000
Grade 5	0	0	0.00	0	0	0.00	0	0	0.00	1.0000

Adverse Event Name	Recombinant Vaccine group(N=896)			Inactivated vaccine (N=904)			Total (N=1800)			
Severity	**Adverse events**	**Cases**	**Incidence (%)**	**Adverse events**	**Cases**	**Incidence (%)**	**Adverse events**	**Cases**	**Incidence (%)**	**P Value[1]**
Grade 2 and above	58	43	4.80	43	30	3.32	101	73	4.06	0.1210
Grade 3 and above	4	4	0.45	3	2	0.22	7	6	0.33	0.4504
fever	24	24	2.68	22	22	2.43	46	46	2.56	0.7671
Grade 1	18	18	2.01	19	19	2.10	37	37	2.06	1.0000
Grade 2	3	3	0.33	1	1	0.11	4	4	0.22	0.3724
Grade 3	3	3	0.33	2	2	0.22	5	5	0.28	0.6858
Grade 4	0	0	0.00	0	0	0.00	0	0	0.00	1.0000
Grade 5	0	0	0.00	0	0	0.00	0	0	0.00	1.0000
Grade 2 and above	6	6	0.67	3	3	0.33	9	9	0.50	0.3405
Grade 3 and above	3	3	0.33	2	2	0.22	5	5	0.28	0.6858
Diarrhea	2	2	0.22	2	2	0.22	4	4	0.22	1.0000
Grade 1	2	2	0.22	2	2	0.22	4	4	0.22	1.0000
Grade 2	0	0	0.00	0	0	0.00	0	0	0.00	1.0000
Grade 3	0	0	0.00	0	0	0.00	0	0	0.00	1.0000
Grade 4	0	0	0.00	0	0	0.00	0	0	0.00	1.0000
Grade 5	0	0	0.00	0	0	0.00	0	0	0.00	1.0000
Grade 2 and above	0	0	0.00	0	0	0.00	0	0	0.00	1.0000
Grade 3 and above	0	0	0.00	0	0	0.00	0	0	0.00	1.0000
Constipation	0	0	0.00	0	0	0.00	0	0	0.00	1.0000
Grade 1	0	0	0.00	0	0	0.00	0	0	0.00	1.0000
Grade 2	0	0	0.00	0	0	0.00	0	0	0.00	1.0000
Grade 3	0	0	0.00	0	0	0.00	0	0	0.00	1.0000
Grade 4	0	0	0.00	0	0	0.00	0	0	0.00	1.0000
Grade 5	0	0	0.00	0	0	0.00	0	0	0.00	1.0000
Grade 2 and above	0	0	0.00	0	0	0.00	0	0	0.00	1.0000
Grade 3 and above	0	0	0.00	0	0	0.00	0	0	0.00	1.0000
Dysphagia	0	0	0.00	0	0	0.00	0	0	0.00	1.0000
Grade 1	0	0	0.00	0	0	0.00	0	0	0.00	1.0000
Grade 2	0	0	0.00	0	0	0.00	0	0	0.00	1.0000
Grade 3	0	0	0.00	0	0	0.00	0	0	0.00	1.0000
Grade 4	0	0	0.00	0	0	0.00	0	0	0.00	1.0000
Grade 5	0	0	0.00	0	0	0.00	0	0	0.00	1.0000
Grade 2 and above	0	0	0.00	0	0	0.00	0	0	0.00	1.0000
Grade 3 and above	0	0	0.00	0	0	0.00	0	0	0.00	1.0000
Anorexia	0	0	0.00	0	0	0.00	0	0	0.00	1.0000
Grade 1	0	0	0.00	0	0	0.00	0	0	0.00	1.0000
Grade 2	0	0	0.00	0	0	0.00	0	0	0.00	1.0000
Grade 3	0	0	0.00	0	0	0.00	0	0	0.00	1.0000

Adverse Event Name<br><br>Severity	Recombinant Vaccine group(N=896)			Inactivated vaccine (N=904)			Total (N=1800)			P Value[1]
	Adverse events	Cases	Incidence (%)	Adverse events	Cases	Incidence (%)	Adverse events	Cases	Incidence (%)	
Grade 4	0	0	0.00	0	0	0.00	0	0	0.00	1.0000
Grade 5	0	0	0.00	0	0	0.00	0	0	0.00	1.0000
Grade 2 and above	0	0	0.00	0	0	0.00	0	0	0.00	1.0000
Grade 3 and above	0	0	0.00	0	0	0.00	0	0	0.00	1.0000
Vomiting	2	2	0.22	1	1	0.11	3	3	0.17	0.6231
Grade 1	1	1	0.11	1	1	0.11	2	2	0.11	1.0000
Grade 2	1	1	0.11	0	0	0.00	1	1	0.06	0.4978
Grade 3	0	0	0.00	0	0	0.00	0	0	0.00	1.0000
Grade 4	0	0	0.00	0	0	0.00	0	0	0.00	1.0000
Grade 5	0	0	0.00	0	0	0.00	0	0	0.00	1.0000
Grade 2 and above	1	1	0.11	0	0	0.00	1	1	0.06	0.4978
Grade 3 and above	0	0	0.00	0	0	0.00	0	0	0.00	1.0000
Nausea	5	5	0.56	2	2	0.22	7	7	0.39	0.2861
Grade 1	4	4	0.45	2	2	0.22	6	6	0.33	0.4504
Grade 2	1	1	0.11	0	0	0.00	1	1	0.06	0.4978
Grade 3	0	0	0.00	0	0	0.00	0	0	0.00	1.0000
Grade 4	0	0	0.00	0	0	0.00	0	0	0.00	1.0000
Grade 5	0	0	0.00	0	0	0.00	0	0	0.00	1.0000
Grade 2 and above	1	1	0.11	0	0	0.00	1	1	0.06	0.4978
Grade 3 and above	0	0	0.00	0	0	0.00	0	0	0.00	1.0000
Muscle pain (non-inoculation site)	30	30	3.35	25	25	2.77	55	55	3.06	0.4961
Grade 1	19	19	2.12	16	16	1.77	35	35	1.94	0.6128
Grade 2	10	10	1.12	8	8	0.88	18	18	1.00	0.6446
Grade 3	1	1	0.11	1	1	0.11	2	2	0.11	1.0000
Grade 4	0	0	0.00	0	0	0.00	0	0	0.00	1.0000
Grade 5	0	0	0.00	0	0	0.00	0	0	0.00	1.0000
Grade 2 and above	11	11	1.23	9	9	1.00	20	20	1.11	0.6605
Grade 3 and above	1	1	0.11	1	1	0.11	2	2	0.11	1.0000
Arthralgia	2	2	0.22	2	2	0.22	4	4	0.22	1.0000
Grade 1	1	1	0.11	2	2	0.22	3	3	0.17	1.0000
Grade 2	1	1	0.11	0	0	0.00	1	1	0.06	0.4978
Grade 3	0	0	0.00	0	0	0.00	0	0	0.00	1.0000
Grade 4	0	0	0.00	0	0	0.00	0	0	0.00	1.0000
Grade 5	0	0	0.00	0	0	0.00	0	0	0.00	1.0000
Grade 2 and above	1	1	0.11	0	0	0.00	1	1	0.06	0.4978
Grade 3 and above	0	0	0.00	0	0	0.00	0	0	0.00	1.0000
Headache	54	52	5.80	38	38	4.20	92	90	5.00	0.1304

Adverse Event Name	Recombinant Vaccine group(N=896)			Inactivated vaccine (N=904)			Total (N=1800)			
Severity	Adverse events	Cases	Incidence (%)	Adverse events	Cases	Incidence (%)	Adverse events	Cases	Incidence (%)	P Value[1]
Grade 1	31	30	3.35	18	18	1.99	49	48	2.67	0.0801
Grade 2	23	23	2.57	20	20	2.21	43	43	2.39	0.6463
Grade 3	0	0	0.00	0	0	0.00	0	0	0.00	1.0000
Grade 4	0	0	0.00	0	0	0.00	0	0	0.00	1.0000
Grade 5	0	0	0.00	0	0	0.00	0	0	0.00	1.0000
Grade 2 and above	23	23	2.57	20	20	2.21	43	43	2.39	0.6463
Grade 3 and above	0	0	0.00	0	0	0.00	0	0	0.00	1.0000
coughing	20	20	2.23	14	13	1.44	34	33	1.83	0.2233
Grade 1	14	14	1.56	9	9	1.00	23	23	1.28	0.3024
Grade 2	6	6	0.67	5	5	0.55	11	11	0.61	0.7728
Grade 3	0	0	0.00	0	0	0.00	0	0	0.00	1.0000
Grade 4	0	0	0.00	0	0	0.00	0	0	0.00	1.0000
Grade 5	0	0	0.00	0	0	0.00	0	0	0.00	1.0000
Grade 2 and above	6	6	0.67	5	5	0.55	11	11	0.61	0.7728
Grade 3 and above	0	0	0.00	0	0	0.00	0	0	0.00	1.0000
Dyspnea	0	0	0.00	1	1	0.11	1	1	0.06	1.0000
Grade 1	0	0	0.00	1	1	0.11	1	1	0.06	1.0000
Grade 2	0	0	0.00	0	0	0.00	0	0	0.00	1.0000
Grade 3	0	0	0.00	0	0	0.00	0	0	0.00	1.0000
Grade 4	0	0	0.00	0	0	0.00	0	0	0.00	1.0000
Grade 5	0	0	0.00	0	0	0.00	0	0	0.00	1.0000
Grade 2 and above	0	0	0.00	0	0	0.00	0	0	0.00	1.0000
Grade 3 and above	0	0	0.00	0	0	0.00	0	0	0.00	1.0000
Itching at non-inoculation site (no skin damage)	0	0	0.00	0	0	0.00	0	0	0.00	1.0000
Grade 1	0	0	0.00	0	0	0.00	0	0	0.00	1.0000
Grade 2	0	0	0.00	0	0	0.00	0	0	0.00	1.0000
Grade 3	0	0	0.00	0	0	0.00	0	0	0.00	1.0000
Grade 4	0	0	0.00	0	0	0.00	0	0	0.00	1.0000
Grade 5	0	0	0.00	0	0	0.00	0	0	0.00	1.0000
Grade 2 and above	0	0	0.00	0	0	0.00	0	0	0.00	1.0000
Grade 3 and above	0	0	0.00	0	0	0.00	0	0	0.00	1.0000
Abnormal skin and mucosa	0	0	0.00	0	0	0.00	0	0	0.00	1.0000
Grade 1	0	0	0.00	0	0	0.00	0	0	0.00	1.0000
Grade 2	0	0	0.00	0	0	0.00	0	0	0.00	1.0000
Grade 3	0	0	0.00	0	0	0.00	0	0	0.00	1.0000
Grade 4	0	0	0.00	0	0	0.00	0	0	0.00	1.0000
Grade 5	0	0	0.00	0	0	0.00	0	0	0.00	1.0000

Adverse Event Name<br><br>Severity	Recombinant Vaccine group(N=896)			Inactivated vaccine (N=904)			Total (N=1800)			
	**Adverse events**	**Cases**	**Incidence (%)**	**Adverse events**	**Cases**	**Incidence (%)**	**Adverse events**	**Cases**	**Incidence (%)**	**P Value[1]**
Grade 2 and above	0	0	0.00	0	0	0.00	0	0	0.00	1.0000
Grade 3 and above	0	0	0.00	0	0	0.00	0	0	0.00	1.0000
Acute allergic reaction	0	0	0.00	0	0	0.00	0	0	0.00	1.0000
Grade 1	0	0	0.00	0	0	0.00	0	0	0.00	1.0000
Grade 2	0	0	0.00	0	0	0.00	0	0	0.00	1.0000
Grade 3	0	0	0.00	0	0	0.00	0	0	0.00	1.0000
Grade 4	0	0	0.00	0	0	0.00	0	0	0.00	1.0000
Grade 5	0	0	0.00	0	0	0.00	0	0	0.00	1.0000
Grade 2 and above	0	0	0.00	0	0	0.00	0	0	0.00	1.0000
Grade 3 and above	0	0	0.00	0	0	0.00	0	0	0.00	1.0000
Fatigue	28	27	3.01	22	22	2.43	50	49	2.72	0.4721
Grade 1	19	19	2.12	17	17	1.88	36	36	2.00	0.7393
Grade 2	9	9	1.00	5	5	0.55	14	14	0.78	0.2979
Grade 3	0	0	0.00	0	0	0.00	0	0	0.00	1.0000
Grade 4	0	0	0.00	0	0	0.00	0	0	0.00	1.0000
Grade 5	0	0	0.00	0	0	0.00	0	0	0.00	1.0000
Grade 2 and above	9	9	1.00	5	5	0.55	14	14	0.78	0.2979
Grade 3 and above	0	0	0.00	0	0	0.00	0	0	0.00	1.0000
Vertigo	1	1	0.11	2	2	0.22	3	3	0.17	1.0000
Grade 1	1	1	0.11	1	1	0.11	2	2	0.11	1.0000
Grade 2	0	0	0.00	1	1	0.11	1	1	0.06	1.0000
Grade 3	0	0	0.00	0	0	0.00	0	0	0.00	1.0000
Grade 4	0	0	0.00	0	0	0.00	0	0	0.00	1.0000
Grade 5	0	0	0.00	0	0	0.00	0	0	0.00	1.0000
Grade 2 and above	0	0	0.00	1	1	0.11	1	1	0.06	1.0000
Grade 3 and above	0	0	0.00	0	0	0.00	0	0	0.00	1.0000

[1]P value was calculated by Fisher exact test.

(1) Unsolicited adverse events are coded using MedDRA version 24.1, and solicited adverse events are listed according to the event name specified in the protocol.

(2) Vaccine-related means that the relationship between adverse events and vaccine-related is "definitely related," "likely related," or "likely related."

(3) This analysis data is up to January 25, 2022.

Local adverse events related to the test vaccines:

After the whole subjects received the booster vaccinations, the most common local adverse events (local adverse reactions) related to the test vaccines were pain, occurred 42 cases (4.62%) with 42 times in the recombinant vaccine group and 36 cases (3.98%) with 36 times in inactivated vaccine group. There was a statistically different between the

groups. The incidence of other local adverse reactions was less than 1.00% both in the two groups, and there was no statistically significant difference between the groups. The severity of all kinds of local adverse reactions was mainly graded 1. In the recombinant vaccine group, 6 cases with 6 times of grade 2 pain occurred, while in the inactivated vaccine group, 4 cases with 4 times of grade 2 pain and 4 cases with 4 times of grade 2 pruritus occurred. No local adverse reactions with grade 3 occurred in both groups. See Table 1-19 and Table 1-20 for details.

Table 1-19 Local adverse events related to the test vaccines in all subjects during the booster period (SS)

	Recombinant Vaccine Group (N=896)			Inactivated Vaccine Group (N=904)			Total (N=1800)			
Adverse event name	Adverse events	Cases	Incidence (%)	Adverse events	Cases	Incidence (%)	Adverse events	Cases	Incidence (%)	P Value[1]
Local adverse events related to test vaccine	47	46	5.13	45	42	4.65	92	88	4.89	0.6629
Pain	42	42	4.69	36	36	3.98	78	78	4.33	0.4888
Hard knot	0	0	0.00	0	0	0.00	0	0	0.00	1.0000
Swelling	0	0	0.00	1	1	0.11	1	1	0.06	1.0000
Rash	0	0	0.00	2	2	0.22	2	2	0.11	0.4997
Flush	2	2	0.22	0	0	0.00	2	2	0.11	0.2476
Pruritus	3	3	0.33	6	6	0.66	9	9	0.50	0.5068

[1]P value was calculated by Fisher exact test.
(1) Unsolicited adverse events are coded using MedDRA version 24.1, and solicited adverse events are listed according to the event name specified in the protocol.
(2) Vaccine-related means that the relationship between adverse events and vaccine-related is "definitely related," "most likely related," or "likely related."
(3) This analysis data is up to January 25, 2022.

Table 1-20 Distribution of severity of local adverse events related to the test vaccines in all subjects during the booster period (SS)

Name of Adverse Event	Recombinant vaccine group (N=896)			Inactivated vaccine group(N=904)			Total (N=1800)			
Severity	Adverse events	Cases	Incidence (%)	Adverse events	Cases	Incidence (%)	Adverse events	Cases	Incidence (%)	P Value[1]
Local adverse events related to test vaccine	47	46	5.13	45	42	4.65	92	88	4.89	0.6629
Grade 1	41	40	4.46	37	37	4.09	78	77	4.28	0.7277
Grade 2	6	6	0.67	8	8	0.88	14	14	0.78	0.7897
Grade 3	0	0	0.00	0	0	0.00	0	0	0.00	1.0000
Grade 4	0	0	0.00	0	0	0.00	0	0	0.00	1.0000

Name of Adverse Event	Recombinant vaccine group (N=896)			Inactivated vaccine group(N=904)			Total (N=1800)			
Severity	**Adverse events**	**Cases**	**Incidence (%)**	**Adverse events**	**Cases**	**Incidence (%)**	**Adverse events**	**Cases**	**Incidence (%)**	**P Value[1]**
Grade 5	0	0	0.00	0	0	0.00	0	0	0.00	1.0000
Grade 2 and above	6	6	0.67	8	8	0.88	14	14	0.78	0.7897
Grade 3 and above	0	0	0.00	0	0	0.00	0	0	0.00	1.0000
pain	42	42	4.69	36	36	3.98	78	78	4.33	0.4888
Grade 1	36	36	4.02	32	32	3.54	68	68	3.78	0.6225
Grade 2	6	6	0.67	4	4	0.44	10	10	0.56	0.5460
Grade 3	0	0	0.00	0	0	0.00	0	0	0.00	1.0000
Grade 4	0	0	0.00	0	0	0.00	0	0	0.00	1.0000
Grade 5	0	0	0.00	0	0	0.00	0	0	0.00	1.0000
Grade 2 and above	6	6	0.67	4	4	0.44	10	10	0.56	0.5460
Grade 3 and above	0	0	0.00	0	0	0.00	0	0	0.00	1.0000
Induration	0	0	0.00	0	0	0.00	0	0	0.00	1.0000
Grade 1	0	0	0.00	0	0	0.00	0	0	0.00	1.0000
Grade 2	0	0	0.00	0	0	0.00	0	0	0.00	1.0000
Grade 3	0	0	0.00	0	0	0.00	0	0	0.00	1.0000
Grade 4	0	0	0.00	0	0	0.00	0	0	0.00	1.0000
Grade 5	0	0	0.00	0	0	0.00	0	0	0.00	1.0000
Grade 2 and above	0	0	0.00	0	0	0.00	0	0	0.00	1.0000
Grade 3 and above	0	0	0.00	0	0	0.00	0	0	0.00	1.0000
Swelling	0	0	0.00	1	1	0.11	1	1	0.06	1.0000
Grade 1	0	0	0.00	1	1	0.11	1	1	0.06	1.0000
Grade 2	0	0	0.00	0	0	0.00	0	0	0.00	1.0000
Grade 3	0	0	0.00	0	0	0.00	0	0	0.00	1.0000
Grade 4	0	0	0.00	0	0	0.00	0	0	0.00	1.0000
Grade 5	0	0	0.00	0	0	0.00	0	0	0.00	1.0000
Grade 2 and above	0	0	0.00	0	0	0.00	0	0	0.00	1.0000
Grade 3 and above	0	0	0.00	0	0	0.00	0	0	0.00	1.0000
Skin rash	0	0	0.00	2	2	0.22	2	2	0.11	0.4997
Grade 1	0	0	0.00	2	2	0.22	2	2	0.11	0.4997
Grade 2	0	0	0.00	0	0	0.00	0	0	0.00	1.0000
Grade 3	0	0	0.00	0	0	0.00	0	0	0.00	1.0000
Grade 4	0	0	0.00	0	0	0.00	0	0	0.00	1.0000
Grade 5	0	0	0.00	0	0	0.00	0	0	0.00	1.0000
Grade 2 and above	0	0	0.00	0	0	0.00	0	0	0.00	1.0000
Grade 3 and above	0	0	0.00	0	0	0.00	0	0	0.00	1.0000
Flush	2	2	0.22	0	0	0.00	2	2	0.11	0.2476

Name of Adverse Event	Recombinant vaccine group (N=896)			Inactivated vaccine group(N=904)			Total (N=1800)			
Severity	Adverse events	Cases	Incidence (%)	Adverse events	Cases	Incidence (%)	Adverse events	Cases	Incidence (%)	P Value[1]
Grade 1	2	2	0.22	0	0	0.00	2	2	0.11	0.2476
Grade 2	0	0	0.00	0	0	0.00	0	0	0.00	1.0000
Grade 3	0	0	0.00	0	0	0.00	0	0	0.00	1.0000
Grade 4	0	0	0.00	0	0	0.00	0	0	0.00	1.0000
Grade 5	0	0	0.00	0	0	0.00	0	0	0.00	1.0000
Grade 2 and above	0	0	0.00	0	0	0.00	0	0	0.00	1.0000
Grade 3 and above	0	0	0.00	0	0	0.00	0	0	0.00	1.0000
Pruritus	3	3	0.33	6	6	0.66	9	9	0.50	0.5068
Grade 1	3	3	0.33	2	2	0.22	5	5	0.28	0.6858
Grade 2	0	0	0.00	4	4	0.44	4	4	0.22	0.1246
Grade 3	0	0	0.00	0	0	0.00	0	0	0.00	1.0000
Grade 4	0	0	0.00	0	0	0.00	0	0	0.00	1.0000
Grade 5	0	0	0.00	0	0	0.00	0	0	0.00	1.0000
Grade 2 and above	0	0	0.00	4	4	0.44	4	4	0.22	0.1246
Grade 3 and above	0	0	0.00	0	0	0.00	0	0	0.00	1.0000

[1]P value was calculated by Fisher exact test.

(1) Unsolicited adverse events are coded using MedDRA version 24.1, and solicited adverse events are listed according to the event name specified in the protocol.

(2) Vaccine-related means that the relationship between adverse events and vaccine-related is "definitely related," "likely related," or "likely related."

(3) This analysis data is up to January 25, 2022.

1.2.3.2 Unsolicited adverse events

There were 147 cases (8.17%) with 235 unsolicited adverse events in all subjects after booster doses, 81 cases (9.04%) with 133 times, and 66 cases (7.30%) with 102 times in the Recombinant vaccine group and in the inactivated vaccine group, respectively. There was no statistically significant difference between the two groups. Unsolicited adverse events related to testing vaccines occurred in 96 cases (5.33%) with 148 times, of which 48 cases (5.36%) with 74 times were reported in the recombinant vaccine group and 48 cases (5.31%) with 74 times were recorded in the inactivated vaccine group, respectively. There was no statistically significant difference between the groups. The main diseases are the respiratory system, chest, and mediastinum, of which the incidence rate was 2.01% and 2.65% in the recombinant vaccine and inactivated vaccine groups, respectively. The severity of all kinds of non-solicitation adverse events related to testing vaccines is mainly Grade 1 or Grade 2. There was 1 case with 1 time of grade 3 oropharyngeal pain and 1 case with 1 time of grade 3 myalgia in the Recombinant vaccine

group, and 1 case with 1 times of grade 3 chest pain in the inactivated vaccine group. See Table 1-21, Table 1-22, and Table 1-23 for details.

Table 1-21 Unsolicited adverse events in all subjects during the booster period (SS)

Name of Adverse event	Recombinant vaccine group(N=896)			Inactivated vaccine group(N=904)			Total (N=1800)			
	Adverse events	Cases	Incidence (%)	Adverse events	Cases	Incidence (%)	Adverse events	Cases	Incidence (%)	P Value[1]
Unsolicited adverse events	133	81	9.04	102	66	7.30	235	147	8.17	0.1967
**Respiratory, thoracic and mediastinal diseases**	40	28	3.13	44	33	3.65	84	61	3.39	0.6029
Cough	12	12	1.34	14	14	1.55	26	26	1.44	0.8439
Runny nose	10	10	1.12	10	10	1.11	20	20	1.11	1.0000
Oropharyngeal pain	10	9	1.00	7	7	0.77	17	16	0.89	0.6261
Nasal congestion	5	5	0.56	8	8	0.88	13	13	0.72	0.5797
Difficult breathing	1	1	0.11	2	2	0.22	3	3	0.17	1.0000
Sneeze	0	0	0.00	2	2	0.22	2	2	0.11	0.4997
Epistaxis	0	0	0.00	1	1	0.11	1	1	0.06	1.0000
Anaphylactic rhinitis	1	1	0.11	0	0	0.00	1	1	0.06	0.4978
Hemoptysis	1	1	0.11	0	0	0.00	1	1	0.06	0.4978
**Systemic diseases and various reactions at administration sites**	33	28	3.13	22	19	2.10	55	47	2.61	0.1859
Pain	3	3	0.33	1	1	0.11	4	4	0.22	0.3724
Fever	15	15	1.67	8	8	0.88	23	23	1.28	0.1473
Fatigue	6	6	0.67	5	5	0.55	11	11	0.61	0.7728
chest pain	2	2	0.22	5	5	0.55	7	7	0.39	0.4522
exogenous fever	4	4	0.45	0	0	0.00	4	4	0.22	0.0612
Swell	1	1	0.11	1	1	0.11	2	2	0.11	1.0000
Chill	1	1	0.11	0	0	0.00	1	1	0.06	0.4978
Nodule	0	0	0.00	1	1	0.11	1	1	0.06	1.0000
Influenza like illness	0	0	0.00	1	1	0.11	1	1	0.06	1.0000
Pruritus at the vaccination site	1	1	0.11	0	0	0.00	1	1	0.06	0.4978
**Diseases of the nervous system**	16	15	1.67	12	12	1.33	28	27	1.50	0.5671
Headache	6	6	0.67	6	6	0.66	12	12	0.67	1.0000
drowsiness	3	3	0.33	2	2	0.22	5	5	0.28	0.6858
swirl	3	3	0.33	2	2	0.22	5	5	0.28	0.6858
hypaesthesia	2	2	0.22	0	0	0.00	2	2	0.11	0.2476
dysarthria	0	0	0.00	1	1	0.11	1	1	0.06	1.0000
myasthenia	0	0	0.00	1	1	0.11	1	1	0.06	1.0000
ageusia	1	1	0.11	0	0	0.00	1	1	0.06	0.4978
anosmia	1	1	0.11	0	0	0.00	1	1	0.06	0.4978
**Various musculoskeletal and connective tissue diseases**	14	13	1.45	9	9	1.00	23	22	1.22	0.4001
myalgia	14	13	1.45	8	8	0.88	22	21	1.17	0.2815
arthralgia	0	0	0.00	1	1	0.11	1	1	0.06	1.0000

Name of Adverse event	Recombinant vaccine group(N=896)			Inactivated vaccine group(N=904)			Total (N=1800)			
	Adverse events	Cases	Incidence (%)	Adverse events	Cases	Incidence (%)	Adverse events	Cases	Incidence (%)	P Value[1]
**Infectious and infectious diseases**	7	7	0.78	5	4	0.44	12	11	0.61	0.3842
nasopharyngitis	7	7	0.78	3	3	0.33	10	10	0.56	0.2233
influenza	0	0	0.00	2	1	0.11	2	1	0.06	1.0000
Diseases of gastrointestinal system	6	6	0.67	5	4	0.44	11	10	0.56	0.5460
stomachache	0	0	0.00	3	3	0.33	3	3	0.17	0.2496
epigastric pain	2	2	0.22	1	1	0.11	3	3	0.17	0.6231
abdominal distension	1	1	0.11	0	0	0.00	1	1	0.06	0.4978
Xerostomia	1	1	0.11	0	0	0.00	1	1	0.06	0.4978
Oral pain	1	1	0.11	0	0	0.00	1	1	0.06	0.4978
indigestion	1	1	0.11	0	0	0.00	1	1	0.06	0.4978
toothache	0	0	0.00	1	1	0.11	1	1	0.06	1.0000
**Kidney and urinary system diseases**	4	3	0.33	1	1	0.11	5	4	0.22	0.3724
frequent micturition	1	1	0.11	1	1	0.11	2	2	0.11	1.0000
diuresis	1	1	0.11	0	0	0.00	1	1	0.06	0.4978
dysuresia	1	1	0.11	0	0	0.00	1	1	0.06	0.4978
haematuria	1	1	0.11	0	0	0.00	1	1	0.06	0.4978
**psychonosology**	3	3	0.33	1	1	0.11	4	4	0.22	0.3724
insomnia	2	2	0.22	1	1	0.11	3	3	0.17	0.6231
anxiety	1	1	0.11	0	0	0.00	1	1	0.06	0.4978
**immune system disease**	3	3	0.33	1	1	0.11	4	4	0.22	0.3724
hypersensitivity	3	3	0.33	1	1	0.11	4	4	0.22	0.3724
**All kinds of inspection**	1	1	0.11	2	2	0.22	3	3	0.17	1.0000
elevated blood pressure	1	1	0.11	1	1	0.11	2	2	0.11	1.0000
Heart rate increases	0	0	0.00	1	1	0.11	1	1	0.06	1.0000
Ocular diseases	3	3	0.33	0	0	0.00	3	3	0.17	0.1231
wild-eyed	1	1	0.11	0	0	0.00	1	1	0.06	0.4978
Eye irritation	1	1	0.11	0	0	0.00	1	1	0.06	0.4978
Pruritus ophthalmia	1	1	0.11	0	0	0.00	1	1	0.06	0.4978
**Diseases of the ear and labyrinth**	1	1	0.11	0	0	0.00	1	1	0.06	0.4978
earache	1	1	0.11	0	0	0.00	1	1	0.06	0.4978
**Skin and subcutaneous tissue diseases**	1	1	0.11	0	0	0.00	1	1	0.06	0.4978
erythema	1	1	0.11	0	0	0.00	1	1	0.06	0.4978

Name of Adverse event	Recombinant vaccine group(N=896)			Inactivated vaccine group(N=904)			Total (N=1800)			
	Adverse events	Cases	Incidence (%)	Adverse events	Cases	Incidence (%)	Adverse events	Cases	Incidence (%)	P Value[1]
**Diseases of reproductive system and mammary gland**	1	1	0.11	0	0	0.00	1	1	0.06	0.4978
erectile dysfunction	1	1	0.11	0	0	0.00	1	1	0.06	0.4978

[1]P-value was calculated using Fisher's exact probability method.

(1) Non-solicitation adverse events are coded by MedDRA version 24.1, and solicitation adverse events are listed according to the event names specified in the plan.

(2) This analysis data is up to January 25, 2022.

Table 1-22 Unsolicited adverse events related to the testing vaccines in all subjects during the booster period (SS)

Adverse Event	Recombinant vaccine group(N=896)			Inactivated vaccine Group (N=904)			Total (N=1800)			
	Adverse events	Cases	Incidence (%)	Adverse events	Cases	Incidence (%)	Adverse events	Cases	Incidence (%)	P Value[1]
Unsolicited adverse events	74	48	5.36	74	48	5.31	148	96	5.33	1.0000
**Respiratory, thoracic and mediastinal diseases**	26	18	2.01	34	24	2.65	60	42	2.33	0.4356
Cough	3	3	0.33	8	8	0.88	11	11	0.61	0.2252
Runny nose	8	8	0.89	9	9	1.00	17	17	0.94	1.0000
Oropharyngeal pain	8	8	0.89	6	6	0.66	14	14	0.78	0.6043
Nasal congestion	5	5	0.56	6	6	0.66	11	11	0.61	1.0000
Difficult breathing	1	1	0.11	2	2	0.22	3	3	0.17	1.0000
Sneeze	0	0	0.00	2	2	0.22	2	2	0.11	0.4997
Epistaxis	0	0	0.00	1	1	0.11	1	1	0.06	1.0000
Anaphylactic rhinitis	1	1	0.11	0	0	0.00	1	1	0.06	0.4978
**Systemic diseases and various reactions at administration sites**	15	13	1.45	16	15	1.66	31	28	1.56	0.8494
Pain	3	3	0.33	1	1	0.11	4	4	0.22	0.3724
Fever	4	4	0.45	5	5	0.55	9	9	0.50	1.0000
stethalgia	2	2	0.22	4	4	0.44	6	6	0.33	0.6870
fatigue	1	1	0.11	4	4	0.44	5	5	0.28	0.3743
exogenous fever	4	4	0.45	0	0	0.00	4	4	0.22	0.0612
swell	1	1	0.11	1	1	0.11	2	2	0.11	1.0000
influenza like illness	0	0	0.00	1	1	0.11	1	1	0.06	1.0000
**Diseases of the nervous system**	5	5	0.56	7	7	0.77	12	12	0.67	0.7737
headache	0	0	0.00	3	3	0.33	3	3	0.17	0.2496
drowsiness	2	2	0.22	2	2	0.22	4	4	0.22	1.0000

Adverse Event	Recombinant vaccine group(N=896)			Inactivated vaccine Group (N=904)			Total (N=1800)			P Value[1]
	Adverse events	Cases	Incidence (%)	Adverse events	Cases	Incidence (%)	Adverse events	Cases	Incidence (%)	
swirl	2	2	0.22	1	1	0.11	3	3	0.17	0.6231
hypaesthesia	1	1	0.11	0	0	0.00	1	1	0.06	0.4978
myasthenia	0	0	0.00	1	1	0.11	1	1	0.06	1.0000
**Various musculoskeletal and connective tissue diseases**	6	5	0.56	6	6	0.66	12	11	0.61	1.0000
myalgia	6	5	0.56	6	6	0.66	12	11	0.61	1.0000
**Infectious and infectious diseases**	6	6	0.67	5	4	0.44	11	10	0.56	0.5460
nasopharyngitis	6	6	0.67	3	3	0.33	9	9	0.50	0.3405
influenza	0	0	0.00	2	1	0.11	2	1	0.06	1.0000
**Diseases of gastrointestinal system**	6	6	0.67	3	3	0.33	9	9	0.50	0.3405
stomachache	0	0	0.00	3	3	0.33	3	3	0.17	0.2496
epigastric pain	2	2	0.22	0	0	0.00	2	2	0.11	0.2476
abdominal distension	1	1	0.11	0	0	0.00	1	1	0.06	0.4978
Xerostomia	1	1	0.11	0	0	0.00	1	1	0.06	0.4978
Oral pain	1	1	0.11	0	0	0.00	1	1	0.06	0.4978
indigestion	1	1	0.11	0	0	0.00	1	1	0.06	0.4978
**psychonosology**	2	2	0.22	1	1	0.11	3	3	0.17	0.6231
insomnia	2	2	0.22	1	1	0.11	3	3	0.17	0.6231
**Kidney and urinary system diseases**	3	2	0.22	0	0	0.00	3	2	0.11	0.2476
frequent micturition	1	1	0.11	0	0	0.00	1	1	0.06	0.4978
diuresis	1	1	0.11	0	0	0.00	1	1	0.06	0.4978
dysuresia	1	1	0.11	0	0	0.00	1	1	0.06	0.4978
**Ocular diseases**	3	3	0.33	0	0	0.00	3	3	0.17	0.1231
wild-eyed	1	1	0.11	0	0	0.00	1	1	0.06	0.4978
Eye irritation	1	1	0.11	0	0	0.00	1	1	0.06	0.4978
Pruritus ophthalmia	1	1	0.11	0	0	0.00	1	1	0.06	0.4978
**Diseases of the ear and labyrinth**	1	1	0.11	0	0	0.00	1	1	0.06	0.4978
earache	1	1	0.11	0	0	0.00	1	1	0.06	0.4978
**All kinds of inspection**	0	0	0.00	1	1	0.11	1	1	0.06	1.0000
Heart rate increases	0	0	0.00	1	1	0.11	1	1	0.06	1.0000
**disease of immune system**	0	0	0.00	1	1	0.11	1	1	0.06	1.0000
hypersensitivity	0	0	0.00	1	1	0.11	1	1	0.06	1.0000
**Diseases of skin and subcutaneous tissue**	1	1	0.11	0	0	0.00	1	1	0.06	0.4978

Adverse Event	Recombinant vaccine group(N=896) Adverse events	Cases	Incidence (%)	Inactivated vaccine Group (N=904) Adverse events	Cases	Incidence (%)	Total (N=1800) Adverse events	Cases	Incidence (%)	P Value[1]
erythema	1	1	0.11	0	0	0.00	1	1	0.06	0.4978

[1]P-value was calculated using Fisher's exact probability method.

(1) Non-solicitation adverse events are coded by MedDRA version 24.1, and solicitation adverse events are listed according to the event names specified in the plan.

(2) Vaccine-related means that the relationship between adverse events and vaccine-related is "definitely related," "most likely related," or "likely related."

(3) This analysis data is up to January 25, 2022.

Table 1-23 Severity distribution of unsolicited adverse events related to the testing vaccines during the booster period for all subjects (SS)

Name of Adverse Event / Severity	Recombinant vaccine group (N=896) Adverse events	Cases	Incidence (%)	Inactivated vaccine group(N=904) Adverse events	Cases	Incidence (%)	Total (N=1800) Adverse events	Cases	Incidence (%)	P Value[1]
**Unsolicited adverse events**	74	48	5.36	74	48	5.31	148	96	5.33	1.0000
Grade 1	49	40	4.46	42	32	3.54	91	72	4.00	0.3376
Grade 2	23	13	1.45	31	22	2.43	54	35	1.94	0.1714
Grade 3	2	2	0.22	1	1	0.11	3	3	0.17	0.6231
Grade 4	0	0	0.00	0	0	0.00	0	0	0.00	1.0000
Grade 5	0	0	0.00	0	0	0.00	0	0	0.00	1.0000
Grade 2 and above	25	15	1.67	32	22	2.43	57	37	2.06	0.3191
Grade 3 and above	2	2	0.22	1	1	0.11	3	3	0.17	0.6231
Respiratory, thoracic and mediastinal diseases	26	18	2.01	34	24	2.65	60	42	2.33	0.4356
Grade 1	15	12	1.34	17	15	1.66	32	27	1.50	0.6990
Grade 2	10	6	0.67	17	12	1.33	27	18	1.00	0.2356
Grade 3	1	1	0.11	0	0	0.00	1	1	0.06	0.4978
Grade 4	0	0	0.00	0	0	0.00	0	0	0.00	1.0000
Grade 5	0	0	0.00	0	0	0.00	0	0	0.00	1.0000
Grade 2 and above	11	7	0.78	17	12	1.33	28	19	1.06	0.3568
Grade 3 and above	1	1	0.11	0	0	0.00	1	1	0.06	0.4978
Cough	3	3	0.33	8	8	0.88	11	11	0.61	0.2252
Grade 1	2	2	0.22	4	4	0.44	6	6	0.33	0.6870
Grade 2	1	1	0.11	4	4	0.44	5	5	0.28	0.3743
Grade 3	0	0	0.00	0	0	0.00	0	0	0.00	1.0000

Name of Adverse Event	Recombinant vaccine group (N=896)			Inactivated vaccine group(N=904)			Total (N=1800)			
Severity	**Adverse events**	**Cases**	**Incidence (%)**	**Adverse events**	**Cases**	**Incidence (%)**	**Adverse events**	**Cases**	**Incidence (%)**	**P Value[1]**
Grade 4	0	0	0.00	0	0	0.00	0	0	0.00	1.0000
Grade 5	0	0	0.00	0	0	0.00	0	0	0.00	1.0000
Grade 2 and above	1	1	0.11	4	4	0.44	5	5	0.28	0.3743
Grade 3 and above	0	0	0.00	0	0	0.00	0	0	0.00	1.0000
Runny nose	8	8	0.89	9	9	1.00	17	17	0.94	1.0000
Grade 1	4	4	0.45	5	5	0.55	9	9	0.50	1.0000
Grade 2	4	4	0.45	4	4	0.44	8	8	0.44	1.0000
Grade 3	0	0	0.00	0	0	0.00	0	0	0.00	1.0000
Grade 4	0	0	0.00	0	0	0.00	0	0	0.00	1.0000
Grade 5	0	0	0.00	0	0	0.00	0	0	0.00	1.0000
Grade 2 and above	4	4	0.45	4	4	0.44	8	8	0.44	1.0000
Grade 3 and above	0	0	0.00	0	0	0.00	0	0	0.00	1.0000
Oropharyngeal pain	8	8	0.89	6	6	0.66	14	14	0.78	0.6043
Grade 1	4	4	0.45	3	3	0.33	7	7	0.39	0.7249
Grade 2	3	3	0.33	3	3	0.33	6	6	0.33	1.0000
Grade 3	1	1	0.11	0	0	0.00	1	1	0.06	0.4978
Grade 4	0	0	0.00	0	0	0.00	0	0	0.00	1.0000
Grade 5	0	0	0.00	0	0	0.00	0	0	0.00	1.0000
Grade 2 and above	4	4	0.45	3	3	0.33	7	7	0.39	0.7249
Grade 3 and above	1	1	0.11	0	0	0.00	1	1	0.06	0.4978
Nasal obstruction	5	5	0.56	6	6	0.66	11	11	0.61	1.0000
Grade 1	4	4	0.45	2	2	0.22	6	6	0.33	0.4504
Grade 2	1	1	0.11	4	4	0.44	5	5	0.28	0.3743
Grade 3	0	0	0.00	0	0	0.00	0	0	0.00	1.0000
Grade 4	0	0	0.00	0	0	0.00	0	0	0.00	1.0000
Grade 5	0	0	0.00	0	0	0.00	0	0	0.00	1.0000
Grade 2 and above	1	1	0.11	4	4	0.44	5	5	0.28	0.3743
Grade 3 and above	0	0	0.00	0	0	0.00	0	0	0.00	1.0000
dyspnea	1	1	0.11	2	2	0.22	3	3	0.17	1.0000
Grade 1	0	0	0.00	1	1	0.11	1	1	0.06	1.0000
Grade 2	1	1	0.11	1	1	0.11	2	2	0.11	1.0000
Grade 3	0	0	0.00	0	0	0.00	0	0	0.00	1.0000
Grade 4	0	0	0.00	0	0	0.00	0	0	0.00	1.0000
Grade 5	0	0	0.00	0	0	0.00	0	0	0.00	1.0000
Grade 2 and above	1	1	0.11	1	1	0.11	2	2	0.11	1.0000
Grade 3 and above	0	0	0.00	0	0	0.00	0	0	0.00	1.0000

Name of Adverse Event	Recombinant vaccine group **(N=896)**			Inactivated vaccine group**(N=904)**			**Total (N=1800)**			
Severity	**Adverse events**	**Cases**	**Incidence (%)**	**Adverse events**	**Cases**	**Incidence (%)**	**Adverse events**	**Cases**	**Incidence (%)**	**P Value[1]**
Sneeze	0	0	0.00	2	2	0.22	2	2	0.11	0.4997
Grade 1	0	0	0.00	1	1	0.11	1	1	0.06	1.0000
Grade 2	0	0	0.00	1	1	0.11	1	1	0.06	1.0000
Grade 3	0	0	0.00	0	0	0.00	0	0	0.00	1.0000
Grade 4	0	0	0.00	0	0	0.00	0	0	0.00	1.0000
Grade 5	0	0	0.00	0	0	0.00	0	0	0.00	1.0000
Grade 2 and above	0	0	0.00	1	1	0.11	1	1	0.06	1.0000
Grade 3 and above	0	0	0.00	0	0	0.00	0	0	0.00	1.0000
epistaxis	0	0	0.00	1	1	0.11	1	1	0.06	1.0000
Grade 1	0	0	0.00	1	1	0.11	1	1	0.06	1.0000
Grade 2	0	0	0.00	0	0	0.00	0	0	0.00	1.0000
Grade 3	0	0	0.00	0	0	0.00	0	0	0.00	1.0000
Grade 4	0	0	0.00	0	0	0.00	0	0	0.00	1.0000
Grade 5	0	0	0.00	0	0	0.00	0	0	0.00	1.0000
Grade 2 and above	0	0	0.00	0	0	0.00	0	0	0.00	1.0000
Grade 3 and above	0	0	0.00	0	0	0.00	0	0	0.00	1.0000
anaphylactic rhinitis	1	1	0.11	0	0	0.00	1	1	0.06	0.4978
Grade 1	1	1	0.11	0	0	0.00	1	1	0.06	0.4978
Grade 2	0	0	0.00	0	0	0.00	0	0	0.00	1.0000
Grade 3	0	0	0.00	0	0	0.00	0	0	0.00	1.0000
Grade 4	0	0	0.00	0	0	0.00	0	0	0.00	1.0000
Grade 5	0	0	0.00	0	0	0.00	0	0	0.00	1.0000
Grade 2 and above	0	0	0.00	0	0	0.00	0	0	0.00	1.0000
Grade 3 and above	0	0	0.00	0	0	0.00	0	0	0.00	1.0000
Systemic diseases and various reactions at administration sites	15	13	1.45	16	15	1.66	31	28	1.56	0.8494
Grade 1	12	12	1.34	10	9	1.00	22	21	1.17	0.5193
Grade 2	3	3	0.33	5	5	0.55	8	8	0.44	0.7260
Grade 3	0	0	0.00	1	1	0.11	1	1	0.06	1.0000
Grade 4	0	0	0.00	0	0	0.00	0	0	0.00	1.0000
Grade 5	0	0	0.00	0	0	0.00	0	0	0.00	1.0000
Grade 2 and above	3	3	0.33	6	6	0.66	9	9	0.50	0.5068
Grade 3 and above	0	0	0.00	1	1	0.11	1	1	0.06	1.0000
Pain	3	3	0.33	1	1	0.11	4	4	0.22	0.3724
Grade 1	3	3	0.33	1	1	0.11	4	4	0.22	0.3724
Grade 2	0	0	0.00	0	0	0.00	0	0	0.00	1.0000

Name of Adverse Event	Recombinant vaccine group **(N=896)**			Inactivated vaccine group**(N=904)**			**Total (N=1800)**			
Severity	**Adverse events**	**Cases**	**Incidence (%)**	**Adverse events**	**Cases**	**Incidence (%)**	**Adverse events**	**Cases**	**Incidence (%)**	**P Value[1]**
Grade 3	0	0	0.00	0	0	0.00	0	0	0.00	1.0000
Grade 4	0	0	0.00	0	0	0.00	0	0	0.00	1.0000
Grade 5	0	0	0.00	0	0	0.00	0	0	0.00	1.0000
Grade 2 and above	0	0	0.00	0	0	0.00	0	0	0.00	1.0000
Grade 3 and above	0	0	0.00	0	0	0.00	0	0	0.00	1.0000
Fever	4	4	0.45	5	5	0.55	9	9	0.50	1.0000
Grade 1	3	3	0.33	4	4	0.44	7	7	0.39	1.0000
Grade 2	1	1	0.11	1	1	0.11	2	2	0.11	1.0000
Grade 3	0	0	0.00	0	0	0.00	0	0	0.00	1.0000
Grade 4	0	0	0.00	0	0	0.00	0	0	0.00	1.0000
Grade 5	0	0	0.00	0	0	0.00	0	0	0.00	1.0000
Grade 2 and above	1	1	0.11	1	1	0.11	2	2	0.11	1.0000
Grade 3 and above	0	0	0.00	0	0	0.00	0	0	0.00	1.0000
stethalgia	2	2	0.22	4	4	0.44	6	6	0.33	0.6870
Grade 1	1	1	0.11	3	3	0.33	4	4	0.22	0.6246
Grade 2	1	1	0.11	0	0	0.00	1	1	0.06	0.4978
Grade 3	0	0	0.00	1	1	0.11	1	1	0.06	1.0000
Grade 4	0	0	0.00	0	0	0.00	0	0	0.00	1.0000
Grade 5	0	0	0.00	0	0	0.00	0	0	0.00	1.0000
Grade 2 and above	1	1	0.11	1	1	0.11	2	2	0.11	1.0000
Grade 3 and above	0	0	0.00	1	1	0.11	1	1	0.06	1.0000
Fatigue	1	1	0.11	4	4	0.44	5	5	0.28	0.3743
Grade 1	1	1	0.11	0	0	0.00	1	1	0.06	0.4978
Grade 2	0	0	0.00	4	4	0.44	4	4	0.22	0.1246
Grade 3	0	0	0.00	0	0	0.00	0	0	0.00	1.0000
Grade 4	0	0	0.00	0	0	0.00	0	0	0.00	1.0000
Grade 5	0	0	0.00	0	0	0.00	0	0	0.00	1.0000
Grade 2 and above	0	0	0.00	4	4	0.44	4	4	0.22	0.1246
Grade 3 and above	0	0	0.00	0	0	0.00	0	0	0.00	1.0000
exogenous fever	4	4	0.45	0	0	0.00	4	4	0.22	0.0612
Grade 1	3	3	0.33	0	0	0.00	3	3	0.17	0.1231
Grade 2	1	1	0.11	0	0	0.00	1	1	0.06	0.4978
Grade 3	0	0	0.00	0	0	0.00	0	0	0.00	1.0000
Grade 4	0	0	0.00	0	0	0.00	0	0	0.00	1.0000
Grade 5	0	0	0.00	0	0	0.00	0	0	0.00	1.0000
Grade 2 and above	1	1	0.11	0	0	0.00	1	1	0.06	0.4978
Grade 3 and above	0	0	0.00	0	0	0.00	0	0	0.00	1.0000

Name of Adverse Event	Recombinant vaccine group (N=896)			Inactivated vaccine group(N=904)			Total (N=1800)			
Severity	**Adverse events**	**Cases**	**Incidence (%)**	**Adverse events**	**Cases**	**Incidence (%)**	**Adverse events**	**Cases**	**Incidence (%)**	**P Value[1]**
swell	1	1	0.11	1	1	0.11	2	2	0.11	1.0000
Grade 1	1	1	0.11	1	1	0.11	2	2	0.11	1.0000
Grade 2	0	0	0.00	0	0	0.00	0	0	0.00	1.0000
Grade 3	0	0	0.00	0	0	0.00	0	0	0.00	1.0000
Grade 4	0	0	0.00	0	0	0.00	0	0	0.00	1.0000
Grade 5	0	0	0.00	0	0	0.00	0	0	0.00	1.0000
Grade 2 and above	0	0	0.00	0	0	0.00	0	0	0.00	1.0000
Grade 3 and above	0	0	0.00	0	0	0.00	0	0	0.00	1.0000
influenza like illness	0	0	0.00	1	1	0.11	1	1	0.06	1.0000
Grade 1	0	0	0.00	1	1	0.11	1	1	0.06	1.0000
Grade 2	0	0	0.00	0	0	0.00	0	0	0.00	1.0000
Grade 3	0	0	0.00	0	0	0.00	0	0	0.00	1.0000
Grade 4	0	0	0.00	0	0	0.00	0	0	0.00	1.0000
Grade 5	0	0	0.00	0	0	0.00	0	0	0.00	1.0000
Grade 2 and above	0	0	0.00	0	0	0.00	0	0	0.00	1.0000
Grade 3 and above	0	0	0.00	0	0	0.00	0	0	0.00	1.0000
Diseases of the nervous system	5	5	0.56	7	7	0.77	12	12	0.67	0.7737
Grade 1	4	4	0.45	4	4	0.44	8	8	0.44	1.0000
Grade 2	1	1	0.11	3	3	0.33	4	4	0.22	0.6246
Grade 3	0	0	0.00	0	0	0.00	0	0	0.00	1.0000
Grade 4	0	0	0.00	0	0	0.00	0	0	0.00	1.0000
Grade 5	0	0	0.00	0	0	0.00	0	0	0.00	1.0000
Grade 2 and above	1	1	0.11	3	3	0.33	4	4	0.22	0.6246
Grade 3 and above	0	0	0.00	0	0	0.00	0	0	0.00	1.0000
headache	0	0	0.00	3	3	0.33	3	3	0.17	0.2496
Grade 1	0	0	0.00	0	0	0.00	0	0	0.00	1.0000
Grade 2	0	0	0.00	3	3	0.33	3	3	0.17	0.2496
Grade 3	0	0	0.00	0	0	0.00	0	0	0.00	1.0000
Grade 4	0	0	0.00	0	0	0.00	0	0	0.00	1.0000
Grade 5	0	0	0.00	0	0	0.00	0	0	0.00	1.0000
Grade 2 and above	0	0	0.00	3	3	0.33	3	3	0.17	0.2496
Grade 3 and above	0	0	0.00	0	0	0.00	0	0	0.00	1.0000
drowsiness	2	2	0.22	2	2	0.22	4	4	0.22	1.0000
Grade 1	2	2	0.22	2	2	0.22	4	4	0.22	1.0000
Grade 2	0	0	0.00	0	0	0.00	0	0	0.00	1.0000
Grade 3	0	0	0.00	0	0	0.00	0	0	0.00	1.0000
Grade 4	0	0	0.00	0	0	0.00	0	0	0.00	1.0000

Name of Adverse Event	Recombinant vaccine group (N=896)			Inactivated vaccine group(N=904)			Total (N=1800)			
Severity	Adverse events	Cases	Incidence (%)	Adverse events	Cases	Incidence (%)	Adverse events	Cases	Incidence (%)	P Value[1]
Grade 5	0	0	0.00	0	0	0.00	0	0	0.00	1.0000
Grade 2 and above	0	0	0.00	0	0	0.00	0	0	0.00	1.0000
Grade 3 and above	0	0	0.00	0	0	0.00	0	0	0.00	1.0000
swirl	2	2	0.22	1	1	0.11	3	3	0.17	0.6231
Grade 1	1	1	0.11	1	1	0.11	2	2	0.11	1.0000
Grade 2	1	1	0.11	0	0	0.00	1	1	0.06	0.4978
Grade 3	0	0	0.00	0	0	0.00	0	0	0.00	1.0000
Grade 4	0	0	0.00	0	0	0.00	0	0	0.00	1.0000
Grade 5	0	0	0.00	0	0	0.00	0	0	0.00	1.0000
Grade 2 and above	1	1	0.11	0	0	0.00	1	1	0.06	0.4978
Grade 3 and above	0	0	0.00	0	0	0.00	0	0	0.00	1.0000
hypesthesia	1	1	0.11	0	0	0.00	1	1	0.06	0.4978
Grade 1	1	1	0.11	0	0	0.00	1	1	0.06	0.4978
Grade 2	0	0	0.00	0	0	0.00	0	0	0.00	1.0000
Grade 3	0	0	0.00	0	0	0.00	0	0	0.00	1.0000
Grade 4	0	0	0.00	0	0	0.00	0	0	0.00	1.0000
Grade 5	0	0	0.00	0	0	0.00	0	0	0.00	1.0000
Grade 2 and above	0	0	0.00	0	0	0.00	0	0	0.00	1.0000
Grade 3 and above	0	0	0.00	0	0	0.00	0	0	0.00	1.0000
myasthenia	0	0	0.00	1	1	0.11	1	1	0.06	1.0000
Grade 1	0	0	0.00	1	1	0.11	1	1	0.06	1.0000
Grade 2	0	0	0.00	0	0	0.00	0	0	0.00	1.0000
Grade 3	0	0	0.00	0	0	0.00	0	0	0.00	1.0000
Grade 4	0	0	0.00	0	0	0.00	0	0	0.00	1.0000
Grade 5	0	0	0.00	0	0	0.00	0	0	0.00	1.0000
Grade 2 and above	0	0	0.00	0	0	0.00	0	0	0.00	1.0000
Grade 3 and above	0	0	0.00	0	0	0.00	0	0	0.00	1.0000
Various musculoskeletal and connective tissue diseases	6	5	0.56	6	6	0.66	12	11	0.61	1.0000
Grade 1	1	1	0.11	2	2	0.22	3	3	0.17	1.0000
Grade 2	4	3	0.33	4	4	0.44	8	7	0.39	1.0000
Grade 3	1	1	0.11	0	0	0.00	1	1	0.06	0.4978
Grade 4	0	0	0.00	0	0	0.00	0	0	0.00	1.0000
Grade 5	0	0	0.00	0	0	0.00	0	0	0.00	1.0000
Grade 2 and above	5	4	0.45	4	4	0.44	9	8	0.44	1.0000
Grade 3 and above	1	1	0.11	0	0	0.00	1	1	0.06	0.4978

Name of Adverse Event	Recombinant vaccine group (N=896)			Inactivated vaccine group(N=904)			Total (N=1800)			
Severity	**Adverse events**	**Cases**	**Incidence (%)**	**Adverse events**	**Cases**	**Incidence (%)**	**Adverse events**	**Cases**	**Incidence (%)**	**P Value[1]**
myalgia	6	5	0.56	6	6	0.66	12	11	0.61	1.0000
Grade 1	1	1	0.11	2	2	0.22	3	3	0.17	1.0000
Grade 2	4	3	0.33	4	4	0.44	8	7	0.39	1.0000
Grade 3	1	1	0.11	0	0	0.00	1	1	0.06	0.4978
Grade 4	0	0	0.00	0	0	0.00	0	0	0.00	1.0000
Grade 5	0	0	0.00	0	0	0.00	0	0	0.00	1.0000
Grade 2 and above	5	4	0.45	4	4	0.44	9	8	0.44	1.0000
Grade 3 and above	1	1	0.11	0	0	0.00	1	1	0.06	0.4978
Infectious and infectious diseases	6	6	0.67	5	4	0.44	11	10	0.56	0.5460
Grade 1	6	6	0.67	4	4	0.44	10	10	0.56	0.5460
Grade 2	0	0	0.00	1	1	0.11	1	1	0.06	1.0000
Grade 3	0	0	0.00	0	0	0.00	0	0	0.00	1.0000
Grade 4	0	0	0.00	0	0	0.00	0	0	0.00	1.0000
Grade 5	0	0	0.00	0	0	0.00	0	0	0.00	1.0000
Grade 2 and above	0	0	0.00	1	1	0.11	1	1	0.06	1.0000
Grade 3 and above	0	0	0.00	0	0	0.00	0	0	0.00	1.0000
nasopharyngitis	6	6	0.67	3	3	0.33	9	9	0.50	0.3405
Grade 1	6	6	0.67	3	3	0.33	9	9	0.50	0.3405
Grade 2	0	0	0.00	0	0	0.00	0	0	0.00	1.0000
Grade 3	0	0	0.00	0	0	0.00	0	0	0.00	1.0000
Grade 4	0	0	0.00	0	0	0.00	0	0	0.00	1.0000
Grade 5	0	0	0.00	0	0	0.00	0	0	0.00	1.0000
Grade 2 and above	0	0	0.00	0	0	0.00	0	0	0.00	1.0000
Grade 3 and above	0	0	0.00	0	0	0.00	0	0	0.00	1.0000
influenza	0	0	0.00	2	1	0.11	2	1	0.06	1.0000
Grade 1	0	0	0.00	1	1	0.11	1	1	0.06	1.0000
Grade 2	0	0	0.00	1	1	0.11	1	1	0.06	1.0000
Grade 3	0	0	0.00	0	0	0.00	0	0	0.00	1.0000
Grade 4	0	0	0.00	0	0	0.00	0	0	0.00	1.0000
Grade 5	0	0	0.00	0	0	0.00	0	0	0.00	1.0000
Grade 2 and above	0	0	0.00	1	1	0.11	1	1	0.06	1.0000
Grade 3 and above	0	0	0.00	0	0	0.00	0	0	0.00	1.0000
Diseases of gastrointestinal system	6	6	0.67	3	3	0.33	9	9	0.50	0.3405
Grade 1	4	4	0.45	2	2	0.22	6	6	0.33	0.4504
Grade 2	2	2	0.22	1	1	0.11	3	3	0.17	0.6231

Name of Adverse Event	Recombinant vaccine group **(N=896)**			Inactivated vaccine group**(N=904)**			**Total (N=1800)**			
Severity	**Adverse events**	**Cases**	**Incidence (%)**	**Adverse events**	**Cases**	**Incidence (%)**	**Adverse events**	**Cases**	**Incidence (%)**	**P Value[1]**
Grade 3	0	0	0.00	0	0	0.00	0	0	0.00	1.0000
Grade 4	0	0	0.00	0	0	0.00	0	0	0.00	1.0000
Grade 5	0	0	0.00	0	0	0.00	0	0	0.00	1.0000
Grade 2 and above	2	2	0.22	1	1	0.11	3	3	0.17	0.6231
Grade 3 and above	0	0	0.00	0	0	0.00	0	0	0.00	1.0000
stomachache	0	0	0.00	3	3	0.33	3	3	0.17	0.2496
Grade 1	0	0	0.00	2	2	0.22	2	2	0.11	0.4997
Grade 2	0	0	0.00	1	1	0.11	1	1	0.06	1.0000
Grade 3	0	0	0.00	0	0	0.00	0	0	0.00	1.0000
Grade 4	0	0	0.00	0	0	0.00	0	0	0.00	1.0000
Grade 5	0	0	0.00	0	0	0.00	0	0	0.00	1.0000
Grade 2 and above	0	0	0.00	1	1	0.11	1	1	0.06	1.0000
Grade 3 and above	0	0	0.00	0	0	0.00	0	0	0.00	1.0000
epigastric pain	2	2	0.22	0	0	0.00	2	2	0.11	0.2476
Grade 1	0	0	0.00	0	0	0.00	0	0	0.00	1.0000
Grade 2	2	2	0.22	0	0	0.00	2	2	0.11	0.2476
Grade 3	0	0	0.00	0	0	0.00	0	0	0.00	1.0000
Grade 4	0	0	0.00	0	0	0.00	0	0	0.00	1.0000
Grade 5	0	0	0.00	0	0	0.00	0	0	0.00	1.0000
Grade 2 and above	2	2	0.22	0	0	0.00	2	2	0.11	0.2476
Grade 3 and above	0	0	0.00	0	0	0.00	0	0	0.00	1.0000
abdominal distension	1	1	0.11	0	0	0.00	1	1	0.06	0.4978
Grade 1	1	1	0.11	0	0	0.00	1	1	0.06	0.4978
Grade 2	0	0	0.00	0	0	0.00	0	0	0.00	1.0000
Grade 3	0	0	0.00	0	0	0.00	0	0	0.00	1.0000
Grade 4	0	0	0.00	0	0	0.00	0	0	0.00	1.0000
Grade 5	0	0	0.00	0	0	0.00	0	0	0.00	1.0000
Grade 2 and above	0	0	0.00	0	0	0.00	0	0	0.00	1.0000
Grade 3 and above	0	0	0.00	0	0	0.00	0	0	0.00	1.0000
Xerostomia	1	1	0.11	0	0	0.00	1	1	0.06	0.4978
Grade 1	1	1	0.11	0	0	0.00	1	1	0.06	0.4978
Grade 2	0	0	0.00	0	0	0.00	0	0	0.00	1.0000
Grade 3	0	0	0.00	0	0	0.00	0	0	0.00	1.0000
Grade 4	0	0	0.00	0	0	0.00	0	0	0.00	1.0000
Grade 5	0	0	0.00	0	0	0.00	0	0	0.00	1.0000
Grade 2 and above	0	0	0.00	0	0	0.00	0	0	0.00	1.0000

Name of Adverse Event	Recombinant vaccine group **(N=896)**			Inactivated vaccine group**(N=904)**			**Total (N=1800)**			
Severity	**Adverse events**	**Cases**	**Incidence (%)**	**Adverse events**	**Cases**	**Incidence (%)**	**Adverse events**	**Cases**	**Incidence (%)**	**P Value[1]**
Grade 3 and above	0	0	0.00	0	0	0.00	0	0	0.00	1.0000
Oral pain	1	1	0.11	0	0	0.00	1	1	0.06	0.4978
Grade 1	1	1	0.11	0	0	0.00	1	1	0.06	0.4978
Grade 2	0	0	0.00	0	0	0.00	0	0	0.00	1.0000
Grade 3	0	0	0.00	0	0	0.00	0	0	0.00	1.0000
Grade 4	0	0	0.00	0	0	0.00	0	0	0.00	1.0000
Grade 5	0	0	0.00	0	0	0.00	0	0	0.00	1.0000
Grade 2 and above	0	0	0.00	0	0	0.00	0	0	0.00	1.0000
Grade 3 and above	0	0	0.00	0	0	0.00	0	0	0.00	1.0000
indigestion	1	1	0.11	0	0	0.00	1	1	0.06	0.4978
Grade 1	1	1	0.11	0	0	0.00	1	1	0.06	0.4978
Grade 2	0	0	0.00	0	0	0.00	0	0	0.00	1.0000
Grade 3	0	0	0.00	0	0	0.00	0	0	0.00	1.0000
Grade 4	0	0	0.00	0	0	0.00	0	0	0.00	1.0000
Grade 5	0	0	0.00	0	0	0.00	0	0	0.00	1.0000
Grade 2 and above	0	0	0.00	0	0	0.00	0	0	0.00	1.0000
Grade 3 and above	0	0	0.00	0	0	0.00	0	0	0.00	1.0000
ɔsychonosology	2	2	0.22	1	1	0.11	3	3	0.17	0.6231
Grade 1	2	2	0.22	1	1	0.11	3	3	0.17	0.6231
Grade 2	0	0	0.00	0	0	0.00	0	0	0.00	1.0000
Grade 3	0	0	0.00	0	0	0.00	0	0	0.00	1.0000
Grade 4	0	0	0.00	0	0	0.00	0	0	0.00	1.0000
Grade 5	0	0	0.00	0	0	0.00	0	0	0.00	1.0000
Grade 2 and above	0	0	0.00	0	0	0.00	0	0	0.00	1.0000
Grade 3 and above	0	0	0.00	0	0	0.00	0	0	0.00	1.0000
insomnia	2	2	0.22	1	1	0.11	3	3	0.17	0.6231
Grade 1	2	2	0.22	1	1	0.11	3	3	0.17	0.6231
Grade 2	0	0	0.00	0	0	0.00	0	0	0.00	1.0000
Grade 3	0	0	0.00	0	0	0.00	0	0	0.00	1.0000
Grade 4	0	0	0.00	0	0	0.00	0	0	0.00	1.0000
Grade 5	0	0	0.00	0	0	0.00	0	0	0.00	1.0000
Grade 2 and above	0	0	0.00	0	0	0.00	0	0	0.00	1.0000
Grade 3 and above	0	0	0.00	0	0	0.00	0	0	0.00	1.0000
Kidney and urinary system diseases	3	2	0.22	0	0	0.00	3	2	0.11	0.2476
Grade 1	3	2	0.22	0	0	0.00	3	2	0.11	0.2476
Grade 2	0	0	0.00	0	0	0.00	0	0	0.00	1.0000

Name of Adverse Event	Recombinant vaccine group (N=896)			Inactivated vaccine group(N=904)			Total (N=1800)			
Severity	**Adverse events**	**Cases**	**Incidence (%)**	**Adverse events**	**Cases**	**Incidence (%)**	**Adverse events**	**Cases**	**Incidence (%)**	**P Value[1]**
Grade 3	0	0	0.00	0	0	0.00	0	0	0.00	1.0000
Grade 4	0	0	0.00	0	0	0.00	0	0	0.00	1.0000
Grade 5	0	0	0.00	0	0	0.00	0	0	0.00	1.0000
Grade 2 and above	0	0	0.00	0	0	0.00	0	0	0.00	1.0000
Grade 3 and above	0	0	0.00	0	0	0.00	0	0	0.00	1.0000
diuresis	1	1	0.11	0	0	0.00	1	1	0.06	0.4978
Grade 1	1	1	0.11	0	0	0.00	1	1	0.06	0.4978
Grade 2	0	0	0.00	0	0	0.00	0	0	0.00	1.0000
Grade 3	0	0	0.00	0	0	0.00	0	0	0.00	1.0000
Grade 4	0	0	0.00	0	0	0.00	0	0	0.00	1.0000
Grade 5	0	0	0.00	0	0	0.00	0	0	0.00	1.0000
Grade 2 and above	0	0	0.00	0	0	0.00	0	0	0.00	1.0000
Grade 3 and above	0	0	0.00	0	0	0.00	0	0	0.00	1.0000
frequent micturition	1	1	0.11	0	0	0.00	1	1	0.06	0.4978
Grade 1	1	1	0.11	0	0	0.00	1	1	0.06	0.4978
Grade 2	0	0	0.00	0	0	0.00	0	0	0.00	1.0000
Grade 3	0	0	0.00	0	0	0.00	0	0	0.00	1.0000
Grade 4	0	0	0.00	0	0	0.00	0	0	0.00	1.0000
Grade 5	0	0	0.00	0	0	0.00	0	0	0.00	1.0000
Grade 2 and above	0	0	0.00	0	0	0.00	0	0	0.00	1.0000
Grade 3 and above	0	0	0.00	0	0	0.00	0	0	0.00	1.0000
dysuresia	1	1	0.11	0	0	0.00	1	1	0.06	0.4978
Grade 1	1	1	0.11	0	0	0.00	1	1	0.06	0.4978
Grade 2	0	0	0.00	0	0	0.00	0	0	0.00	1.0000
Grade 3	0	0	0.00	0	0	0.00	0	0	0.00	1.0000
Grade 4	0	0	0.00	0	0	0.00	0	0	0.00	1.0000
Grade 5	0	0	0.00	0	0	0.00	0	0	0.00	1.0000
Grade 2 and above	0	0	0.00	0	0	0.00	0	0	0.00	1.0000
Grade 3 and above	0	0	0.00	0	0	0.00	0	0	0.00	1.0000
Ocular diseases	3	3	0.33	0	0	0.00	3	3	0.17	0.1231
Grade 1	1	1	0.11	0	0	0.00	1	1	0.06	0.4978
Grade 2	2	2	0.22	0	0	0.00	2	2	0.11	0.2476
Grade 3	0	0	0.00	0	0	0.00	0	0	0.00	1.0000
Grade 4	0	0	0.00	0	0	0.00	0	0	0.00	1.0000
Grade 5	0	0	0.00	0	0	0.00	0	0	0.00	1.0000
Grade 2 and above	2	2	0.22	0	0	0.00	2	2	0.11	0.2476

Name of Adverse Event	Recombinant vaccine group (N=896)			Inactivated vaccine group(N=904)			Total (N=1800)			
Severity	Adverse events	Cases	Incidence (%)	Adverse events	Cases	Incidence (%)	Adverse events	Cases	Incidence (%)	P Value[1]
Grade 3 and above	0	0	0.00	0	0	0.00	0	0	0.00	1.0000
wild-eyed	1	1	0.11	0	0	0.00	1	1	0.06	0.4978
Grade 1	1	1	0.11	0	0	0.00	1	1	0.06	0.4978
Grade 2	0	0	0.00	0	0	0.00	0	0	0.00	1.0000
Grade 3	0	0	0.00	0	0	0.00	0	0	0.00	1.0000
Grade 4	0	0	0.00	0	0	0.00	0	0	0.00	1.0000
Grade 5	0	0	0.00	0	0	0.00	0	0	0.00	1.0000
Grade 2 and above	0	0	0.00	0	0	0.00	0	0	0.00	1.0000
Grade 3 and above	0	0	0.00	0	0	0.00	0	0	0.00	1.0000
Eye irritation	1	1	0.11	0	0	0.00	1	1	0.06	0.4978
Grade 1	0	0	0.00	0	0	0.00	0	0	0.00	1.0000
Grade 2	1	1	0.11	0	0	0.00	1	1	0.06	0.4978
Grade 3	0	0	0.00	0	0	0.00	0	0	0.00	1.0000
Grade 4	0	0	0.00	0	0	0.00	0	0	0.00	1.0000
Grade 5	0	0	0.00	0	0	0.00	0	0	0.00	1.0000
Grade 2 and above	1	1	0.11	0	0	0.00	1	1	0.06	0.4978
Grade 3 and above	0	0	0.00	0	0	0.00	0	0	0.00	1.0000
Pruritus ophthalmia	1	1	0.11	0	0	0.00	1	1	0.06	0.4978
Grade 1	0	0	0.00	0	0	0.00	0	0	0.00	1.0000
Grade 2	1	1	0.11	0	0	0.00	1	1	0.06	0.4978
Grade 3	0	0	0.00	0	0	0.00	0	0	0.00	1.0000
Grade 4	0	0	0.00	0	0	0.00	0	0	0.00	1.0000
Grade 5	0	0	0.00	0	0	0.00	0	0	0.00	1.0000
Grade 2 and above	1	1	0.11	0	0	0.00	1	1	0.06	0.4978
Grade 3 and above	0	0	0.00	0	0	0.00	0	0	0.00	1.0000
Diseases of the ear and labyrinth	1	1	0.11	0	0	0.00	1	1	0.06	0.4978
Grade 1	0	0	0.00	0	0	0.00	0	0	0.00	1.0000
Grade 2	1	1	0.11	0	0	0.00	1	1	0.06	0.4978
Grade 3	0	0	0.00	0	0	0.00	0	0	0.00	1.0000
Grade 4	0	0	0.00	0	0	0.00	0	0	0.00	1.0000
Grade 5	0	0	0.00	0	0	0.00	0	0	0.00	1.0000
Grade 2 and above	1	1	0.11	0	0	0.00	1	1	0.06	0.4978
Grade 3 and above	0	0	0.00	0	0	0.00	0	0	0.00	1.0000
earache	1	1	0.11	0	0	0.00	1	1	0.06	0.4978
Grade 1	0	0	0.00	0	0	0.00	0	0	0.00	1.0000
Grade 2	1	1	0.11	0	0	0.00	1	1	0.06	0.4978

Name of Adverse Event	Recombinant vaccine group **(N=896)**			Inactivated vaccine group**(N=904)**			**Total (N=1800)**			
Severity	**Adverse events**	**Cases**	**Incidence (%)**	**Adverse events**	**Cases**	**Incidence (%)**	**Adverse events**	**Cases**	**Incidence (%)**	**P Value[1]**
Grade 3	0	0	0.00	0	0	0.00	0	0	0.00	1.0000
Grade 4	0	0	0.00	0	0	0.00	0	0	0.00	1.0000
Grade 5	0	0	0.00	0	0	0.00	0	0	0.00	1.0000
Grade 2 and above	1	1	0.11	0	0	0.00	1	1	0.06	0.4978
Grade 3 and above	0	0	0.00	0	0	0.00	0	0	0.00	1.0000
All kinds of inspection	0	0	0.00	1	1	0.11	1	1	0.06	1.0000
Grade 1	0	0	0.00	1	1	0.11	1	1	0.06	1.0000
Grade 2	0	0	0.00	0	0	0.00	0	0	0.00	1.0000
Grade 3	0	0	0.00	0	0	0.00	0	0	0.00	1.0000
Grade 4	0	0	0.00	0	0	0.00	0	0	0.00	1.0000
Grade 5	0	0	0.00	0	0	0.00	0	0	0.00	1.0000
Grade 2 and above	0	0	0.00	0	0	0.00	0	0	0.00	1.0000
Grade 3 and above	0	0	0.00	0	0	0.00	0	0	0.00	1.0000
Heart rate increases	0	0	0.00	1	1	0.11	1	1	0.06	1.0000
Grade 1	0	0	0.00	1	1	0.11	1	1	0.06	1.0000
Grade 2	0	0	0.00	0	0	0.00	0	0	0.00	1.0000
Grade 3	0	0	0.00	0	0	0.00	0	0	0.00	1.0000
Grade 4	0	0	0.00	0	0	0.00	0	0	0.00	1.0000
Grade 5	0	0	0.00	0	0	0.00	0	0	0.00	1.0000
Grade 2 and above	0	0	0.00	0	0	0.00	0	0	0.00	1.0000
Grade 3 and above	0	0	0.00	0	0	0.00	0	0	0.00	1.0000
disease of immune system	0	0	0.00	1	1	0.11	1	1	0.06	1.0000
Grade 1	0	0	0.00	1	1	0.11	1	1	0.06	1.0000
Grade 2	0	0	0.00	0	0	0.00	0	0	0.00	1.0000
Grade 3	0	0	0.00	0	0	0.00	0	0	0.00	1.0000
Grade 4	0	0	0.00	0	0	0.00	0	0	0.00	1.0000
Grade 5	0	0	0.00	0	0	0.00	0	0	0.00	1.0000
Grade 2 and above	0	0	0.00	0	0	0.00	0	0	0.00	1.0000
Grade 3 and above	0	0	0.00	0	0	0.00	0	0	0.00	1.0000
hypersensitivity	0	0	0.00	1	1	0.11	1	1	0.06	1.0000
Grade 1	0	0	0.00	1	1	0.11	1	1	0.06	1.0000
Grade 2	0	0	0.00	0	0	0.00	0	0	0.00	1.0000
Grade 3	0	0	0.00	0	0	0.00	0	0	0.00	1.0000
Grade 4	0	0	0.00	0	0	0.00	0	0	0.00	1.0000
Grade 5	0	0	0.00	0	0	0.00	0	0	0.00	1.0000

Name of Adverse Event	Recombinant vaccine group **(N=896)**			Inactivated vaccine group**(N=904)**			**Total (N=1800)**			
Severity	**Adverse events**	**Cases**	**Incidence (%)**	**Adverse events**	**Cases**	**Incidence (%)**	**Adverse events**	**Cases**	**Incidence (%)**	**P Value[1]**
Grade 2 and above	0	0	0.00	0	0	0.00	0	0	0.00	1.0000
Grade 3 and above	0	0	0.00	0	0	0.00	0	0	0.00	1.0000
Diseases of skin and subcutaneous tissue	1	1	0.11	0	0	0.00	1	1	0.06	0.4978
Grade 1	1	1	0.11	0	0	0.00	1	1	0.06	0.4978
Grade 2	0	0	0.00	0	0	0.00	0	0	0.00	1.0000
Grade 3	0	0	0.00	0	0	0.00	0	0	0.00	1.0000
Grade 4	0	0	0.00	0	0	0.00	0	0	0.00	1.0000
Grade 5	0	0	0.00	0	0	0.00	0	0	0.00	1.0000
Grade 2 and above	0	0	0.00	0	0	0.00	0	0	0.00	1.0000
Grade 3 and above	0	0	0.00	0	0	0.00	0	0	0.00	1.0000
erythema	1	1	0.11	0	0	0.00	1	1	0.06	0.4978
Grade 1	1	1	0.11	0	0	0.00	1	1	0.06	0.4978
Grade 2	0	0	0.00	0	0	0.00	0	0	0.00	1.0000
Grade 3	0	0	0.00	0	0	0.00	0	0	0.00	1.0000
Grade 4	0	0	0.00	0	0	0.00	0	0	0.00	1.0000
Grade 5	0	0	0.00	0	0	0.00	0	0	0.00	1.0000
Grade 2 and above	0	0	0.00	0	0	0.00	0	0	0.00	1.0000
Grade 3 and above	0	0	0.00	0	0	0.00	0	0	0.00	1.0000

[1]P value was calculated by Fisher exact test.
(1) Unsolicited adverse events are coded using MedDRA version 24.1, and solicited adverse events are listed according to the event name specified in the protocol.
(2) Vaccine-related means that the relationship between adverse events and vaccine-related is "definitely related," "most likely related," or "likely related."
(3) This analysis data is up to January 25, 2022.

1.2.4 Listing of Adverse Events

See appendix 3.1-3.7 for a list of adverse events.

1.3 Deaths, other serious adverse events, and other significant adverse events

1.3.1 List and narrative of death

No deaths occurred in this clinical trial.

1.3.2 List and narrative of serious adverse events other than death

In this clinical trial, there were 5 cases (0.28%) with 9 times of serious adverse event, 2 cases (0.22%) with 2 times in the recombinant vaccine group, and 3 cases (0.33%) with

7 times (0.33%) in the inactivated vaccine group. The difference between the groups was not statistically significant, and none of them was judged by the investigator to be related to the study vaccine. See Appendix 3.6 for serious adverse event list for details.

1.4 Safety Summary

1) The incidence of total adverse events associated with the test vaccines was 17.52% in the recombinant vaccine group and 14.93% in the inactivated vaccine group, with no significant difference between the two groups (*P*=0.1417); In the recombinant and inactivated vaccine groups, the severity of grade 1 was 15.07% and 12.06%, grade 2 was 5.58% and 5.42%, and grade 3 was 0.56% and 0.22%, respectively.

2) Adverse events related to the testing vaccines mainly occurred in 0-7 days. No adverse events related to the vaccine were observed within 30 minutes. Within 0-7 days, the incidence of adverse reactions in the recombinant vaccine group and inactivated vaccine group were 17.08% and 13.61%, respectively; The incidence of adverse reactions in 8-30 days after booster vaccination was 1.00% and 2.21% in the recombinant vaccine and inactivated vaccine groups, respectively.

3) The incidence of solicited adverse events related to the test vaccine was 16.29% and 12.72% in the recombinant vaccine group and the inactivated vaccine group, respectively, and the difference between the groups was statistically significant (*P*=0.0323); The incidence of systemic adverse reactions in the recombinant and inactivated vaccine groups was 12.39% and 9.29%, respectively (*P*=0.0404). Headache (5.80% and 4.20% in recombinant and inactivated vaccine groups, respectively), muscle pain (3.35% in recombinant vaccine group and 2.77% in inactivated vaccine group), fatigue (3.01% in recombinant vaccine group and 2.43% in inactivated vaccine group) and fever (2.68% in recombinant vaccine group and 2.43% in inactivated vaccine group) were the main ones. The severity was mainly graded 1 or grade 2. In the recombinant vaccine group, 3 cases with 3 times of fever with grade 3 severity and 1 case with 1 time of muscle pain with grade 3 severity (non-inoculated site) occurred, while in the inactivated vaccine group, 2 cases with 2 times of fever with grade 3 severity and 1 case with 1 time of muscle pain with grade 3 severity (non-inoculated site) occurred. The incidence of local adverse reactions in the recombinant vaccine group and the inactivated vaccine group were 5.13% and 4.65%, respectively. There was no statistically significant difference between the groups (*P*=0.6629). The most common local adverse reaction was pain (the incidence rate was 4.69% and 3.98% in the recombinant and inactivated vaccine groups, respectively).

Other local adverse reactions were less than 1% in both groups. There were 6 cases with 6 times of pain of severity grade 2 in the recombinant vaccine group, 4 cases with 4 times of pain of severity grade 2 in the inactivated vaccine group, and 4 cases of 4 times of severity grade 2 itching. There were no local adverse reactions of severity grade 3 in the two groups.

4) The incidence of unsolicited adverse events related to the study vaccine was 5.36% and 5.31% in the recombinant vaccine group and inactivated vaccine group, respectively, and there was no statistically significant difference between the groups (P=1.0000). The most frequent adverse events were mainly respiratory, thoracic, and mediastinal diseases (the incidence rate was 2.01% and 2.65% in the recombinant and inactivated vaccine groups, respectively), and the severity was mainly graded 1 or grade 2. In the recombinant vaccine group, 1 case with 1 time of grade 3 oropharyngeal pain and 1 case with 1 time grade 3 myalgia occurred, while in the inactivated vaccine group, 1 case with 1 time of grade 3 chest pain occurred.

5) During the clinical trial, no subjects died, no adverse events of special interest occurred, and no serious adverse event related to the test vaccine occurred.

2. Discussion And Overall Conclusions (30 Days After Vaccination)

This clinical study evaluated the safety and immunogenicity of the test vaccine (recombinant vaccine, NVSI-06-08) in subjects 18 years of age and above by comparison with positive control (inactivated vaccine).

Safety:

A total of 1800 subjects were included in the SS set of this clinical study, including 896 subjects in the experimental group (receiving a booster dose of the recombinant vaccine) and 904 subjects in the control group (receiving a booster dose of the inactivated vaccine). The baseline characteristics were comparable between the experimental and control groups.

The results showed that there was no significant difference in the incidence of vaccine-related adverse events (adverse reactions) between the experimental and control groups. Adverse reactions mainly occur within 0-7 days after vaccination, which were mainly pain at the vaccination site and headache at the non-vaccination site, muscle pain, fatigue, and fever. The severity of the adverse reactions was mainly grade 1 or 2.

Only a small number of adverse reactions of grade 3 were observed, which include fever (3 cases with 3 adverse events), muscular pain (1 case with 1 adverse event), oropharyngeal pain (1 case with 1 adverse event) and myalgia (1 case with 1 adverse event) in the recombinant vaccine group, and fever (2 cases with 2 adverse events), muscular pain (1 case with 1 adverse event) and chest pain (1 case with 1 adverse event) in the inactivated vaccine group.

In summary, there is no significant difference in the overall safety profile between heterologous boosting with the recombinant vaccines and homologous boosting with the inactivated vaccines.

3. Appendix

3.1. Listing of adverse events related to the study vaccine occurring during the booster period (SS)

Table 3-1 List of adverse events related to the study vaccine during the extraction period (SS)

Adverse event description	System Organ Class	Preferred terms	Type of Adverse Event	Date of inoculation	Start Date	End date	Duration Days	Days from inoculation	Whether within 30min	Severity	Treatment situation	Correlation
Subject No.: 0008, Group: Inactivated Vaccine Group, Age (years): 29.7, Sex: Male, Nationality: Afghanistan												
Cough	Respiratory, thoracic and mediastinal disorders	coughing	Unsolicited	October 24, 2021	2021-11-14			21	No	Level 2	Self-medication	May be relevant
Nasal Congestion	Respiratory, thoracic and mediastinal diseases	Nasal congestion	Non-solicitation	2021-10-24	November 14, 2021			21	No	Grade 2	Self-medication	Possibly related
Subject No.: 0013, Group: Recombinant Vaccine Group, Age (years): 26.1, Sex: Male, Nationality: Pakistan												
Pain	Systemic diseases and various reactions at the administration site	Vaccination site pain	Collection//inoculation site (local)	2021-10-24	October 24, 2021	2021-10-26	3	0	No	Grade 1	Untreated	Definitely related
Subject number: 0036, group: recombinant vaccine group, age (years): 30, sex: male, nationality: India												
Fever: 38.1℃	Systemic diseases and various reactions at the administration site	Fever	Collection//Non-inoculation site (whole body)	2021-10-24	October 24, 2021	October 25, 2021	2	0	No	Grade 1	Self-medication	Definitely related
Headache	Nervous system disorders	Headache	Recruited//Non-Vaccination Site (Systemic)	October 24, 2021	2021-10-24	October 25, 2021	2	0	No	Level 1	No treatment	May be relevant
Fatigue, Asthenia	Systemic diseases and various reactions at the administration site	Fatigue	Non-solicitation	2021-10-24	2021-11-07	2021-11-10	4	14	No	Grade 1	Untreated	Probably related

Adverse event description	System Organ Class	Preferred terms	Type of Adverse Event	Date of inoculation	Start Date	End date	Duration Days	Days from inoculation	Whether within 30min	Severity	Treatment situation	Correlation
Fever: 38.5 ℃.-Temporal Artery-Taken 1000mg tab 2x daily for 3 days.	General disorders and administration site conditions	fever	Unsolicited	October 24, 2021	2021-11-07	November 10, 2021	4	14	No	Level 2	Self-medication	Probably related
Myalgia	Various musculoskeletal and connective tissue diseases	Myalgia	Non-solicitation	2021-10-24	2021-11-07	2021-11-10	4	14	No	Grade 2	Self-medication	Probably related
Subject No.: 0038, Group: Inactivated Vaccine Group, Age (years): 36.5, Sex: Male, Nationality: Bangladesh												
Headache	Various nervous system diseases	Headache	Collection//Non-inoculation site (whole body)	2021-10-24	October 24, 2021	2021-10-26	3	0	No	Grade 1	No medication in outpatient department	Possibly related
Subject No.: 0040, Group: Inactivated Vaccine Group, Age (years): 29.5, Sex: Male, Nationality: Pakistan												
Pain	Systemic diseases and various reactions at the administration site	Vaccination site pain	Collection//inoculation site (local)	2021-10-24	October 25, 2021	2021-10-25	1	1	No	Grade 1	Untreated	Definitely related
Subject No.: 0050, Group: Recombinant Vaccine Group, Age (years): 29.5, Sex: Male, Nationality: Bangladesh												
skin energy on stomach (redness)f	Skin and subcutaneous tissue diseases	Erythema	Non-solicitation	2021-10-24	October 24, 2021	2021-10-26	3	0	No	Grade 1	Untreated	Possibly related
Subject No.: 0057, Group: Recombinant Vaccine Group, Age (years): 24.5, Sex: Male, Nationality: Bangladesh												
Muscle pain (non-injection site)	Various musculoskeletal and connective tissue diseases	Myalgia	Collection//Non-inoculation site (whole body)	2021-10-24	October 24, 2021	2021-10-27	4	0	No	Grade 1	Untreated	Possibly related
Myalgia	Musculoskeletal and connective tissue disorders	Myalgia	Unsolicited	October 24, 2021	2021-11-06	November 30, 2021	25	13	No	Level 2	No treatment	Probably related
Subject No.: 0060, Group: Inactivated Vaccine Group, Age (years): 34.2, Sex: Male, Nationality: Bangladesh												

Adverse event description	System Organ Class	Preferred terms	Type of Adverse Event	Date of inoculation	Start Date	End date	Duration Days	Days from inoculation	Whether within 30min	Severity	Treatment situation	Correlation
Nasal Congestion	Respiratory, thoracic and mediastinal disorders	Nasal obstruction	Unsolicited	October 24, 2021	2021-10-25	October 28, 2021	4	1	No	Level 2	Self-medication	Probably related
RUNNY NOSE	Respiratory, thoracic and mediastinal diseases	Runny nose	Non-solicitation	2021-10-24	October 25, 2021	2021-10-26	2	1	No	Grade 1	Untreated	Possibly related
Fever: 38.1 ℃	General disorders and administration site conditions	fever	Recruited//Non-Vaccination Site (Systemic)	October 24, 2021	2021-10-25	October 28, 2021	4	1	No	Level 1	Self-medication	Definitely relevant
Muscle pain (non-injection site)	Various musculoskeletal and connective tissue diseases	Myalgia	Collection//Non-inoculation site (whole body)	2021-10-24	October 25, 2021	2021-10-28	4	1	No	Grade 2	Self-medication	Probably related
Subject No.: 0068, Group: Recombinant Vaccine Group, Age (years): 27.8, Sex: Male, Nationality: Bangladesh												
Fever: 39.0℃	Systemic diseases and various reactions at the administration site	Fever	Collection//Non-inoculation site (whole body)	2021-10-24	October 24, 2021	2021-10-31	8	0	No	Grade 3	Outpatient medication	Possibly related
coughing	Respiratory, thoracic and mediastinal disorders	coughing	Recruited//Non-Vaccination Site (Systemic)	October 24, 2021	2021-10-27			3	No	Level 1	No treatment	Probably related
Muscle pain (non-injection site)	Various musculoskeletal and connective tissue diseases	Myalgia	Collection//Non-inoculation site (whole body)	2021-10-24	October 27, 2021			3	No	Grade 1	Untreated	Probably related
Subject No.: 0069, Group: Inactivated Vaccine Group, Age (years): 31.4, Sex: Male, Nationality: Bangladesh												
Cough	Respiratory, thoracic and mediastinal diseases	Cough	Collection//Non-inoculation site (whole body)	2021-10-24	October 29, 2021	2021-10-31	3	5	No	Grade 1	Untreated	Possibly related
Subject No.: 0076, Group: Inactivated Vaccine Group, Age (years): 25.8, Sex: Male, Nationality: Bangladesh												
energy	immune system disease	Hypersensitivity reaction	Non-solicitation	2021-10-24	2021-10-26	2021-10-31	6	2	No	Grade 1	No medication in outpatient department	Possibly related

Adverse event description	System Organ Class	Preferred terms	Type of Adverse Event	Date of inoculation	Start Date	End date	Duration Days	Days from inoculation	Whether within 30min	Severity	Treatment situation	Correlation
Subject No.: 0087, Group: Recombinant Vaccine Group, Age (years): 35.1, Sex: Male, Nationality: Bangladesh												
Arthralgia	Various musculoskeletal and connective tissue diseases	Arthralgia	Collection//Non-inoculation site (whole body)	2021-10-25	October 29, 2021	2021-11-05	8	4	No	Grade 2	Self-medication	Possibly related
Subject No.: 0094, Group: Inactivated Vaccine Group, Age (years): 25.2, Sex: Male, Nationality: Pakistan												
Nasal Congestion	Respiratory, thoracic and mediastinal diseases	Nasal congestion	Non-solicitation	2021-10-25	October 25, 2021	2021-10-28	4	0	No	Grade 1	Untreated	Possibly related
Fever: 38.0 ℃, Panadol tab	General disorders and administration site conditions	fever	Recruited//Non-Vaccination Site (Systemic)	October 25, 2021	2021-10-25	October 27, 2021	3	0	No	Level 1	Self-medication	Probably related
Cough	Respiratory, thoracic and mediastinal diseases	Cough	Collection//Non-inoculation site (whole body)	2021-10-25	October 25, 2021	2021-10-27	3	0	No	Grade 2	Self-medication	Probably related
Headache	Nervous system disorders	Headache	Recruited//Non-Vaccination Site (Systemic)	October 25, 2021	2021-10-25	October 28, 2021	4	0	No	Level 1	Self-medication	May be relevant
Fatigue/Asthenia	Systemic diseases and various reactions at the administration site	Fatigue	Collection//Non-inoculation site (whole body)	2021-10-25	October 25, 2021	2021-10-28	4	0	No	Grade 1	Untreated	Possibly related
Muscle pain (non-inoculation site)	Musculoskeletal and connective tissue disorders	Myalgia	Recruited//Non-Vaccination Site (Systemic)	October 25, 2021	2021-10-25	October 28, 2021	4	0	No	Level 2	Self-medication	May be relevant
Flu Symptoms	Infection and infectious diseases	Influenza	Non-solicitation	2021-10-25	October 27, 2021	2021-10-27	1	2	No	Grade 2	Self-medication	Possibly related
Flu symbols	Infections and infestations	Influenza	Unsolicited	October 25, 2021	2021-11-01	November 3, 2021	3	7	No	Level 1	No treatment	May be relevant
Cough	Respiratory, thoracic and mediastinal diseases	Cough	Collection//Non-inoculation site (whole body)	2021-10-25	2021-11-01	2021-11-03	3	7	No	Grade 1	Untreated	Possibly related
Subject No.: 0107, Group: Recombinant Vaccine Group, Age (years): 38.9, Sex: Male, Nationality: Pakistan												

Adverse event description	System Organ Class	Preferred terms	Type of Adverse Event	Date of inoculation	Start Date	End date	Duration Days	Days from inoculation	Whether within 30min	Severity	Treatment situation	Correlation
Headache	Various nervous system diseases	Headache	Collection//Non-inoculation site (whole body)	2021-10-25	October 25, 2021	2021-10-25	1	0	No	Grade 1	Untreated	Possibly related
Muscle pain (non-inoculation site)	Musculoskeletal and connective tissue disorders	Myalgia	Recruited//Non-Vaccination Site (Systemic)	October 25, 2021	2021-10-25	2021-10-26	2	0	No	Level 1	No treatment	May be relevant
Subject No.: 0109, Group: Recombinant vaccine group, Age (years): 25.8, Sex: Male, Nationality: Pakistan												
Fever: 37.8 ℃, CHECKED VIA TYMPANIC, TOOK PANADOL 500 MG PER TABLET ORALLY.ONCE, ALREADY RESOLVED.	General disorders and administration site conditions	fever	Recruited//Non-Vaccination Site (Systemic)	October 25, 2021	2021-10-25	October 25, 2021	1	0	No	Level 1	Self-medication	May be relevant
Subject No.: 0128, Group: Inactivated Vaccine Group, Age (years): 20.6, Sex: Male, Nationality: Pakistan												
pain	General disorders and administration site conditions	Pain at vaccination site	Recruitment//injection site (local)	2021-10-26	2021-10-26	2021-10-26	1	0	No	Level 1	No treatment	Definitely relevant
Subject No.: 0136, Group: Recombinant vaccine group, Age (years): 35.5, Sex: Male, Nationality: India												
Fatigue/fatigue	General disorders and administration site conditions	Fatigue	Recruited//Non-Vaccination Site (Systemic)	2021-10-26	2021-10-27	October 30, 2021	4	1	No	Level 2	No treatment	May be relevant
Subject No.: 0185, Group: Inactivated Vaccine Group, Age (years): 35.4, Sex: Male, Nationality: Bangladesh												
Myalgia	Musculoskeletal and connective tissue disorders	Myalgia	Unsolicited	October 27, 2021	2021-11-13	2021-11-21	9	17	No	Level 2	Self-medication	Probably related

Adverse event description	System Organ Class	Preferred terms	Type of Adverse Event	Date of inoculation	Start Date	End date	Duration Days	Days from inoculation	Whether within 30min	Severity	Treatment situation	Correlation
Subject No.: 0188, Group: Recombinant vaccine group, Age (years): 23.8, Sex: Male, Nationality: Pakistan												
Myalgia	Musculoskeletal and connective tissue disorders	Myalgia	Unsolicited	October 27, 2021	2021-11-19	2021-11-21	3	23	No	Level 1	Self-medication	May be relevant
Subject No.: 0190, Group: Inactivated Vaccine Group, Age (years): 34.4, Sex: Male, Nationality: Pakistan												
Muscle pain (non-inoculation site)	Musculoskeletal and connective tissue disorders	Myalgia	Recruited//Non-Vaccination Site (Systemic)	October 27, 2021	2021-10-27	2021-11-03	8	0	No	Level 2	Self-medication	Probably related
Subject No.: 0204, Group: Recombinant vaccine group, Age (years): 33.9, Sex: Male, Nationality: Pakistan												
Fatigue/fatigue	General disorders and administration site conditions	Fatigue	Recruited//Non-Vaccination Site (Systemic)	October 31, 2021	2021-11-01	November 21-02, 2021	2	1	No	Level 1	No treatment	Probably related
Subject No.: 0207, Group: Inactivated Vaccine Group, Age (years): 32.8, Sex: Male, Nationality: Bangladesh												
Fever: 38.8 ℃	General disorders and administration site conditions	fever	Recruited//Non-Vaccination Site (Systemic)	October 31, 2021	2021-11-01	November 4, 2021	4	1	No	Level 2	No treatment	May be relevant
Fatigue/Asthenia	Systemic diseases and various reactions at the administration site	Fatigue	Collection//Non-inoculation site (whole body)	2021-10-31	2021-11-01	2021-11-04	4	1	No	Grade 1	Untreated	Possibly related
Subject No.: 0210, Group: Inactivated Vaccine Group, Age (years): 20.4, Sex: Male, Nationality: Bangladesh												
Fatigue/Asthenia	Systemic diseases and various reactions at the administration site	Fatigue	Collection//Non-inoculation site (whole body)	2021-10-31	October 31, 2021	2021-11-03	4	0	No	Grade 1	Untreated	Probably related
Subject No.: 0223, Group: Recombinant Vaccine Group, Age (years): 32.8, Sex: Male, Nationality: Philippines												
Sore throat	Respiratory, thoracic and mediastinal diseases	Oropharyngeal pain	Non-solicitation	2021-10-31	2021-11-01	2021-11-01	1	1	No	Grade 1	Untreated	Probably related

Adverse event description	System Organ Class	Preferred terms	Type of Adverse Event	Date of inoculation	Start Date	End date	Duration Days	Days from inoculation	Whether within 30min	Severity	Treatment situation	Correlation
Fatigue/fatigue	General disorders and administration site conditions	Fatigue	Recruited//Non-Vaccination Site (Systemic)	October 31, 2021	2021-11-01	2021-11-01	1	1	No	Level 1	No treatment	Probably related
Subject No.: 0272, Group: Recombinant Vaccine Group, Age (Y): 36.3, Sex: Male, Nationality: Bangladesh												
pain	General disorders and administration site conditions	Pain at vaccination site	Recruitment//injection site (local)	November 21-02, 2021	2021-11-02	November 21-02, 2021	1	0	No	Level 1	No treatment	Definitely relevant
Headache	Various nervous system diseases	Headache	Collection//Non-inoculation site (whole body)	2021-11-02	2021-11-07	2021-11-11	5	5	No	Grade 2	Self-medication	Probably related
Subject No.: 0273, Group: Recombinant Vaccine Group, Age (years): 23.7, Sex: Male, Nationality: Bangladesh												
Cold Symptoms	Infection and infectious diseases	Nasopharyngitis	Non-solicitation	2021-11-02	November 4, 2021	2021-11-06	3	2	No	Grade 1	Untreated	Possibly related
coughing	Respiratory, thoracic and mediastinal disorders	coughing	Recruited//Non-Vaccination Site (Systemic)	November 21-02, 2021	2021-11-04	2021-11-06	3	2	No	Level 1	No treatment	May be relevant
Fever: 37.8℃, TAB.PANADOL 2 TAKEN FOR 2 DAYS.	Systemic diseases and various reactions at the administration site	Fever	Non-solicitation	2021-11-02	November 25, 2021	2021-11-28	4	23	No	Grade 1	Self-medication	Probably related
Cough	Respiratory, thoracic and mediastinal disorders	coughing	Unsolicited	November 21-02, 2021	2021-11-28	November 30, 2021	3	26	No	Level 1	No medication in outpatient clinic	Probably related
Subject No.: 0278, Group: Inactivated Vaccine Group, Age (years): 26.9, Sex: Male, Nationality: India												
Cough	Respiratory, thoracic and mediastinal disorders	coughing	Unsolicited	November 21-02, 2021	2021-11-25	November 28, 2021	4	23	No	Level 1	No treatment	May be relevant

Adverse event description	System Organ Class	Preferred terms	Type of Adverse Event	Date of inoculation	Start Date	End date	Duration Days	Days from inoculation	Whether within 30min	Severity	Treatment situation	Correlation
Fever: 38.0℃, resolved	Systemic diseases and various reactions at the administration site	Fever	Non-solicitation	2021-11-02	November 25, 2021	2021-11-26	2	23	No	Grade 1	Self-medication	Possibly related
Subject No.: 0297, Group: Recombinant Vaccine Group, Age (years): 49.1, Sex: Male, Nationality: Ghana												
frequent urination	Diseases of kidney and urinary system	Urinary frequency	Non-solicitation	2021-11-02	2021-11-03	2021-11-08	6	1	No	Grade 1	Untreated	Possibly related
Subject No.: 0301, Group: Recombinant Vaccine Group, Age (years): 39.2, Sex: Male, Nationality: Uganda												
Headache	Various nervous system diseases	Headache	Collection//Non-inoculation site (whole body)	2021-11-02	November 21-02, 2021	2021-11-02	1	0	No	Grade 1	Untreated	Possibly related
pain	General disorders and administration site conditions	Pain at vaccination site	Recruitment//injection site (local)	November 21-02, 2021	2021-11-02	November 4, 2021	3	0	No	Level 1	No treatment	Definitely relevant
Headache	Various nervous system diseases	Headache	Collection//Non-inoculation site (whole body)	2021-11-02	2021-11-06	2021-11-06	1	4	No	Grade 1	Untreated	Possibly related
Subject No.: 0307, Group: Inactivated Vaccine Group, Age (years): 46.1, Sex: Male, Nationality: Bangladesh												
Headache	Various nervous system diseases	Headache	Collection//Non-inoculation site (whole body)	2021-11-02	November 21-02, 2021	2021-11-02	1	0	No	Grade 2	Self-medication	Probably related
Subject No.: 0308, Group: Inactivated Vaccine Group, Age (years): 41.9, Sex: Male, Nationality: Zimbabwe												
Pain	Systemic diseases and various reactions at the administration site	Vaccination site pain	Collection//inoculation site (local)	2021-11-02	November 21-02, 2021	2021-11-03	2	0	No	Grade 1	Untreated	Definitely related
Subject No.: 0309, Group: Recombinant Vaccine Group, Age (years): 47.4, Sex: Male, Nationality: India												
Headache	Various nervous system diseases	Headache	Collection//Non-inoculation site (whole body)	2021-11-02	2021-11-07	2021-11-07	1	5	No	Grade 1	Untreated	Possibly related
Subject No.: 0311, Group: Recombinant Vaccine Group, Age (years): 25.5, Sex: Male, Nationality: Uganda												

Adverse event description	System Organ Class	Preferred terms	Type of Adverse Event	Date of inoculation	Start Date	End date	Duration Days	Days from inoculation	Whether within 30min	Severity	Treatment situation	Correlation
Fever: 38.0℃, Panadol tab	Systemic diseases and various reactions at the administration site	Fever	Collection//Non-inoculation site (whole body)	2021-11-02	2021-11-03	2021-11-06	4	1	No	Grade 1	Self-medication	Probably related
Fatigue/fatigue	General disorders and administration site conditions	Fatigue	Recruited//Non-Vaccination Site (Systemic)	November 21-02, 2021	2021-11-03	2021-11-06	4	1	No	Level 2	Self-medication	Probably related
Muscle pain (non-injection site)	Various musculoskeletal and connective tissue diseases	Myalgia	Collection//Non-inoculation site (whole body)	2021-11-02	2021-11-03	2021-11-06	4	1	No	Grade 2	Self-medication	Probably related
Subject No.: 0314, Group: Recombinant Vaccine Group, Age (years): 38.1, Sex: Male, Nationality: Zimbabwe												
Feeling of hotness	Systemic diseases and various reactions at the administration site	Feeling hot	Non-solicitation	2021-11-02	November 21-02, 2021	2021-11-05	4	0	No	Grade 1	Self-medication	Probably related
Muscle pain (non-inoculation site)	Musculoskeletal and connective tissue disorders	Myalgia	Recruited//Non-Vaccination Site (Systemic)	November 21-02, 2021	2021-11-02	November 5, 2021	4	0	No	Level 1	Self-medication	Probably related
chest pain	Systemic diseases and various reactions at the administration site	Chest pain	Non-solicitation	2021-11-02	2021-11-03	2021-11-06	4	1	No	Grade 2	Self-medication	Possibly related
Runny Nose	Respiratory, thoracic and mediastinal disorders	Runny nose	Unsolicited	November 21-02, 2021	2021-11-03	2021-11-06	4	1	No	Level 2	Self-medication	May be relevant
sore throat	Respiratory, thoracic and mediastinal diseases	Oropharyngeal pain	Non-solicitation	2021-11-02	2021-11-03	2021-11-06	4	1	No	Grade 2	Self-medication	Probably related
coughing	Respiratory, thoracic and mediastinal disorders	coughing	Recruited//Non-Vaccination Site (Systemic)	November 21-02, 2021	2021-11-03	2021-11-06	4	1	No	Level 2	Self-medication	Probably related
Headache	Various nervous system diseases	Headache	Collection//Non-inoculation site (whole body)	2021-11-02	2021-11-03	2021-11-06	4	1	No	Grade 2	Self-medication	Probably related

Adverse event description	System Organ Class	Preferred terms	Type of Adverse Event	Date of inoculation	Start Date	End date	Duration Days	Days from inoculation	Whether within 30min	Severity	Treatment situation	Correlation
Fatigue/fatigue	General disorders and administration site conditions	Fatigue	Recruited//Non-Vaccination Site (Systemic)	November 21-02, 2021	2021-11-03	2021-11-06	4	1	No	Level 1	No treatment	Probably related
Subject No.: 0318, Group: Recombinant vaccine group, Age (years): 45, Sex: Male, Nationality: Nepal												
Cough	Respiratory, thoracic and mediastinal disorders	coughing	Unsolicited	November 21-02, 2021	2021-11-29	December 5, 2021	7	27	No	Level 1	No treatment	May be relevant
Subject No.: 0321, Group: Recombinant vaccine group, Age (years): 35.8, Sex: Male, Nationality: Pakistan												
Headache	Nervous system disorders	Headache	Recruited//Non-Vaccination Site (Systemic)	November 21-02, 2021	2021-11-08	2021-11-08	1	6	No	Level 1	No treatment	Probably related
Muscle pain (non-injection site)	Various musculoskeletal and connective tissue diseases	Myalgia	Collection//Non-inoculation site (whole body)	2021-11-02	2021-11-08	2021-11-08	1	6	No	Grade 1	Untreated	Probably related
Subject No.: 0325, Group: Inactivated Vaccine Group, Age (years): 37.8, Sex: Male, Nationality: Nepal												
Headache	Various nervous system diseases	Headache	Collection//Non-inoculation site (whole body)	2021-11-02	November 21-02, 2021	2021-11-04	3	0	No	Grade 1	Untreated	Probably related
Subject No.: 0326, Group: Inactivated Vaccine Group, Age (years): 58.1, Sex: Male, Nationality: Bangladesh												
Myalgia	Various musculoskeletal and connective tissue diseases	Myalgia	Non-solicitation	2021-11-02	November 27, 2021	December 3, 2021	7	25	No	Grade 2	Untreated	Probably related
Subject No.: 0328, Group: Inactivated Vaccine Group, Age (years): 26.7, Sex: Male, Nationality: Pakistan												
Pain	Systemic diseases and various reactions at the administration site	Vaccination site pain	Collection//inoculation site (local)	2021-11-02	November 21-02, 2021	2021-11-04	3	0	No	Grade 1	Untreated	Definitely related
Subject No.: 0332, Group: Inactivated Vaccine Group, Age (years): 31.4, Sex: Male, Nationality: Zimbabwe												

Adverse event description	System Organ Class	Preferred terms	Type of Adverse Event	Date of inoculation	Start Date	End date	Duration Days	Days from inoculation	Whether within 30min	Severity	Treatment situation	Correlation
Headache	Various nervous system diseases	Headache	Collection//Non-inoculation site (whole body)	2021-11-02	2021-11-03	2021-11-04	2	1	No	Grade 1	No medication in outpatient department	Probably related
Subject No.: 0333, Group: Recombinant Vaccine Group, Age (years): 27.2, Sex: Male, Nationality: Zimbabwe												
Headache	Various nervous system diseases	Headache	Collection//Non-inoculation site (whole body)	2021-11-02	2021-11-06	2021-11-08	3	4	No	Grade 1	No medication in outpatient department	Probably related
Subject No.: 0342, Group: Inactivated Vaccine Group, Age (years): 42.7, Sex: Male, Nationality: Bangladesh												
Fatigue, Asthenia	Systemic diseases and various reactions at the administration site	Fatigue	Non-solicitation	2021-11-03	November 15, 2021			12	No	Grade 2	Untreated	Probably related
Subject No.: 0346, Group: Inactivated Vaccine Group, Age (years): 39, Sex: Male, Nationality: Nepal												
Headache	Various nervous system diseases	Headache	Collection//Non-inoculation site (whole body)	2021-11-03	2021-11-03	2021-11-10	8	0	No	Grade 2	Self-medication	Probably related
Epistaxis	Respiratory, thoracic and mediastinal disorders	Epistaxis	Unsolicited	2021-11-03	2021-11-04	2021-11-07	4	1	No	Level 1	No treatment	Probably related
Subject No.: 0348, Group: Recombinant vaccine group, Age (years): 32, Sex: Male, Nationality: Nepal												
Fatigue/fatigue	General disorders and administration site conditions	Fatigue	Recruited//Non-Vaccination Site (Systemic)	2021-11-03	2021-11-03	November 4, 2021	2	0	No	Level 1	No treatment	Probably related
Subject No.: 0359, Group: Inactivated Vaccine Group, Age (years): 40.5, Sex: Male, Nationality: Nepal												
Cold Symptoms	Infections and infestations	Nasopharyngitis	Unsolicited	2021-11-03	2021-11-04	November 5, 2021	2	1	No	Level 1	No treatment	May be relevant
Cough	Respiratory, thoracic and mediastinal diseases	Cough	Collection//Non-inoculation site (whole body)	2021-11-03	November 4, 2021	2021-11-07	4	1	No	Grade 1	Untreated	Possibly related

Adverse event description	System Organ Class	Preferred terms	Type of Adverse Event	Date of inoculation	Start Date	End date	Duration Days	Days from inoculation	Whether within 30min	Severity	Treatment situation	Correlation
Runny nose	Respiratory, thoracic and mediastinal disorders	Runny nose	Unsolicited	2021-11-03	2021-11-05	November 15, 2021	11	2	No	Level 2	Self-medication	May be relevant
Fever: 38.0℃	Systemic diseases and various reactions at the administration site	Fever	Collection//Non-inoculation site (whole body)	2021-11-03	November 5, 2021	2021-11-07	3	2	No	Grade 1	Self-medication	Possibly related
Subject No.: 0366, Group: Recombinant Vaccine Group, Age (years): 39.2, Sex: Male, Nationality: Bangladesh												
Nasal congestion	Respiratory, thoracic and mediastinal diseases	Nasal congestion	Non-solicitation	2021-11-03	2021-11-03	2021-11-07	5	0	No	Grade 2	Self-medication	Probably related
Fever: 37.8 ℃, thermal thermometer, on panadol tablet	General disorders and administration site conditions	fever	Recruited//Non-Vaccination Site (Systemic)	2021-11-03	2021-11-03	2021-11-07	5	0	No	Level 1	Self-medication	May be relevant
Cough	Respiratory, thoracic and mediastinal diseases	Cough	Collection//Non-inoculation site (whole body)	2021-11-03	2021-11-03	2021-11-07	5	0	No	Grade 2	Self-medication	Probably related
Subject No.: 0368, Group: Recombinant Vaccine Group, Age (years): 34.3, Sex: Male, Nationality: Pakistan												
Abdominal Bloatedness	Gastrointestinal system diseases	Abdominal distension	Non-solicitation	2021-11-03	November 4, 2021	2021-11-08	5	1	No	Grade 1	Untreated	Probably related
Headache	Nervous system disorders	Headache	Recruited//Non-Vaccination Site (Systemic)	2021-11-03	2021-11-04	2021-11-06	3	1	No	Level 2	Self-medication	Probably related
Subject No.: 0380, Group: Recombinant vaccine group, Age (years): 42.9, Sex: Male, Nationality: Uganda												
pain	General disorders and administration site conditions	Pain at vaccination site	Recruitment//injection site (local)	2021-11-03	2021-11-03	2021-11-06	4	0	No	Level 1	No treatment	Definitely relevant
Cough	Respiratory, thoracic and mediastinal diseases	Cough	Collection//Non-inoculation site (whole body)	2021-11-03	November 5, 2021	2021-11-06	2	2	No	Grade 1	Untreated	Probably related

Adverse event description	System Organ Class	Preferred terms	Type of Adverse Event	Date of inoculation	Start Date	End date	Duration Days	Days from inoculation	Whether within 30min	Severity	Treatment situation	Correlation
Subject No.: 0381, Group: Recombinant Vaccine Group, Age (years): 59.8, Sex: Male, Nationality: Pakistan												
Muscle pain (non-injection site)	Various musculoskeletal and connective tissue diseases	Myalgia	Collection//Non-inoculation site (whole body)	2021-11-03	2021-11-03	2021-11-04	2	0	No	Grade 1	Untreated	Probably related
Subject No.: 0382, Group: Inactivated Vaccine Group, Age (years): 36.4, Sex: Male, Nationality: Uganda												
Cough	Respiratory, thoracic and mediastinal diseases	Cough	Non-solicitation	2021-11-03	2021-11-21	2021-11-26	6	18	No	Grade 1	Untreated	Probably related
Myalgia	Musculoskeletal and connective tissue disorders	Myalgia	Unsolicited	2021-11-03	2021-11-21	November 24, 2021	4	18	No	Level 1	No treatment	Probably related
runny nose	Respiratory, thoracic and mediastinal diseases	Runny nose	Non-solicitation	2021-11-03	2021-11-21	2021-11-26	6	18	No	Grade 1	No medication in outpatient department	Probably related
Subject No.: 0387, Group: Recombinant Vaccine Group, Age (years): 34.6, Sex: Male, Nationality: Nepal												
Headache	Various nervous system diseases	Headache	Collection//Non-inoculation site (whole body)	2021-11-03	2021-11-03	2021-11-15	13	0	No	Grade 2	Prescription drug	Probably related
Muscle pain (non-inoculation site)	Musculoskeletal and connective tissue disorders	Myalgia	Recruited//Non-Vaccination Site (Systemic)	2021-11-03	2021-11-03	November 16, 2021	14	0	No	Level 2	Prescription Drug	Probably related
Cold Symptoms	Infection and infectious diseases	Nasopharyngitis	Non-solicitation	2021-11-03	November 4, 2021	2021-11-06	3	1	No	Grade 1	Untreated	Possibly related
Nausea	Gastrointestinal disorders	Nausea	Recruited//Non-Vaccination Site (Systemic)	2021-11-03	2021-11-04	2021-11-06	3	1	No	Level 1	No treatment	May be relevant
Subject No.: 0388, Group: Recombinant vaccine group, Age (years): 35.8, Sex: Male, Nationality: Nepal												
cold	Infections and infestations	Nasopharyngitis	Unsolicited	2021-11-03	2021-11-03	2021-11-07	5	0	No	Level 1	No medication in outpatient clinic	May be relevant

Adverse event description	System Organ Class	Preferred terms	Type of Adverse Event	Date of inoculation	Start Date	End date	Duration Days	Days from inoculation	Whether within 30min	Severity	Treatment situation	Correlation
Cough	Respiratory, thoracic and mediastinal diseases	Cough	Collection//Non-inoculation site (whole body)	2021-11-03	November 4, 2021	2021-11-07	4	1	No	Grade 1	No medication in outpatient department	Possibly related
Subject No.: 0405, Group: Inactivated Vaccine Group, Age (years): 35.5, Sex: Male, Nationality: Nepal												
Pain	Systemic diseases and various reactions at the administration site	Vaccination site pain	Collection//inoculation site (local)	2021-11-03	2021-11-03	2021-11-04	2	0	No	Grade 1	Untreated	Definitely related
Subject No.: 0410, Group: Recombinant Vaccine Group, Age (years): 31.2, Sex: Male, Nationality: Uganda												
Pain	Systemic diseases and various reactions at the administration site	Vaccination site pain	Collection//inoculation site (local)	2021-11-03	2021-11-03	2021-11-06	4	0	No	Grade 1	Untreated	Definitely related
Subject No.: 0413, Group: Recombinant Vaccine Group, Age (years): 38.6, Sex: Male, Nationality: Nepal												
Headache	Various nervous system diseases	Headache	Collection//Non-inoculation site (whole body)	2021-11-03	November 5, 2021	2021-11-05	1	2	No	Grade 2	Self-medication	Probably related
Subject No.: 0418, Group: Inactivated Vaccine Group, Age (years): 31.3, Sex: Male, Nationality: Pakistan												
Sore Throat	Respiratory, thoracic and mediastinal diseases	Oropharyngeal pain	Non-solicitation	2021-11-03	2021-11-03	2021-11-20	18	0	No	Grade 2	Prescription drug	Possibly related
Fever: 37.9 ℃	General disorders and administration site conditions	fever	Recruited//Non-Vaccination Site (Systemic)	2021-11-03	2021-11-03	November 10, 2021	8	0	No	Level 1	Prescription Drug	Probably related
Cough	Respiratory, thoracic and mediastinal diseases	Cough	Collection//Non-inoculation site (whole body)	2021-11-03	2021-11-03	2021-11-17	15	0	No	Grade 2	Prescription drug	Probably related
Headache	Nervous system disorders	Headache	Recruited//Non-Vaccination Site (Systemic)	2021-11-03	2021-11-03	November 17, 2021	15	0	No	Level 2	Prescription Drug	Probably related
Fatigue/Asthenia	Systemic diseases and various reactions at the administration site	Fatigue	Collection//Non-inoculation site (whole body)	2021-11-03	2021-11-03	2021-11-20	18	0	No	Grade 2	Prescription drug	Probably related

Adverse event description	System Organ Class	Preferred terms	Type of Adverse Event	Date of inoculation	Start Date	End date	Duration Days	Days from inoculation	Whether within 30min	Severity	Treatment situation	Correlation
Muscle pain (non-inoculation site)	Musculoskeletal and connective tissue disorders	Myalgia	Recruited//Non-Vaccination Site (Systemic)	2021-11-03	2021-11-03	November 17, 2021	15	0	No	Level 2	Prescription Drug	Probably related
nasal congestion	Respiratory, thoracic and mediastinal diseases	Nasal congestion	Non-solicitation	2021-11-03	November 4, 2021	2021-11-18	15	1	No	Grade 2	Prescription drug	Probably related
Subject No.: 0420, Group: Inactivated Vaccine Group, Age (years): 55.9, Sex: Male, Nationality: Bangladesh												
Headache	Various nervous system diseases	Headache	Collection//Non-inoculation site (whole body)	2021-11-03	2021-11-03	2021-11-03	1	0	No	Grade 2	Self-medication	Probably related
Subject No.: 0428, Group: Inactivated Vaccine Group, Age (years): 30.3, Sex: Male, Nationality: Uganda												
Headache	Various nervous system diseases	Headache	Collection//Non-inoculation site (whole body)	2021-11-03	2021-11-03	2021-11-18	16	0	No	Grade 2	Self-medication	Probably related
Fatigue/fatigue	General disorders and administration site conditions	Fatigue	Recruited//Non-Vaccination Site (Systemic)	2021-11-03	2021-11-03	November 5, 2021	3	0	No	Level 2	Prescription Drug	Probably related
Subject No.: 0430, Group: Recombinant vaccine group, Age (years): 31.7, Sex: Male, Nationality: Uganda												
Headache	Nervous system disorders	Headache	Recruited//Non-Vaccination Site (Systemic)	2021-11-03	2021-11-04	November 4, 2021	1	1	No	Level 1	No treatment	Probably related
Subject No.: 0432, Group: Inactivated Vaccine Group, Age (years): 35.8, Sex: Male, Nationality: Ethiopia												
Skin rash	General disorders and administration site conditions	Rash at vaccination site	Recruitment//injection site (local)	2021-11-03	2021-11-05	2021-11-06	2	2	No	Level 1	No treatment	Definitely relevant
Pruritus	Skin and subcutaneous tissue diseases	Pruritus	Collection//inoculation site (local)	2021-11-03	2021-11-06	2021-11-10	5	3	No	Grade 2	Untreated	Possibly related
Subject No.: 0435, Group: Inactivated Vaccine Group, Age (years): 33.7, Sex: Male, Nationality: Bangladesh												
Headache	Various nervous system diseases	Headache	Collection//Non-inoculation site (whole body)	2021-11-03	2021-11-03	2021-11-06	4	0	No	Grade 2	Self-medication	Probably related
Subject No.: 0438, Group: Recombinant Vaccine Group, Age (years): 46.1, Sex: Male, Nationality: Nepal												

Adverse event description	System Organ Class	Preferred terms	Type of Adverse Event	Date of inoculation	Start Date	End date	Duration Days	Days from inoculation	Whether within 30min	Severity	Treatment situation	Correlation
Pain	Systemic diseases and various reactions at the administration site	Vaccination site pain	Collection//inoculation site (local)	2021-11-03	2021-11-03	2021-11-03	1	0	No	Grade 1	Untreated	Definitely related
Subject No.: 0440, Group: Recombinant Vaccine Group, Age (years): 36.1, Sex: Male, Nationality: Uganda												
Headache	Various nervous system diseases	Headache	Collection//Non-inoculation site (whole body)	2021-11-03	November 4, 2021	2021-11-06	3	1	No	Grade 1	Untreated	Probably related
Subject No.: 0441, Group: Inactivated Vaccine Group, Age (years): 37.1, Sex: Male, Nationality: Nepal												
sleepiness	Various nervous system diseases	Sleepiness	Non-solicitation	2021-11-03	2021-11-03	2021-11-06	4	0	No	Grade 1	Untreated	Probably related
Fatigue/fatigue	General disorders and administration site conditions	Fatigue	Recruited//Non-Vaccination Site (Systemic)	2021-11-03	2021-11-03	2021-11-06	4	0	No	Level 1	No treatment	Probably related
Headache	Various nervous system diseases	Headache	Collection//Non-inoculation site (whole body)	2021-11-03	November 4, 2021	2021-11-06	3	1	No	Grade 1	Untreated	Possibly related
Muscle pain (non-inoculation site)	Musculoskeletal and connective tissue disorders	Myalgia	Recruited//Non-Vaccination Site (Systemic)	2021-11-03	2021-11-04	2021-11-06	3	1	No	Level 1	No treatment	May be relevant
Subject No.: 0443, Group: Inactivated Vaccine Group, Age (years): 52.5, Sex: Male, Nationality: Ghana												
Chest Pain	General disorders and administration site conditions	Chest pain	Unsolicited	2021-11-03	2021-11-04	2021-11-07	4	1	No	Level 1	No treatment	Probably related
Subject No.: 0445, Group: Inactivated Vaccine Group, Age (years): 29.3, Sex: Male, Nationality: Nepal												
pain	General disorders and administration site conditions	Pain at vaccination site	Recruitment//injection site (local)	2021-11-03	2021-11-04	November 5, 2021	2	1	No	Level 1	No treatment	Definitely relevant
Subject No.: 0450, Group: Recombinant vaccine group, Age (years): 39.3, Sex: Male, Nationality: Uganda												
Nausea	Gastrointestinal disorders	Nausea	Recruited//Non-Vaccination Site (Systemic)	2021-11-03	2021-11-03	November 5, 2021	3	0	No	Level 1	No treatment	May be relevant

Adverse event description	System Organ Class	Preferred terms	Type of Adverse Event	Date of inoculation	Start Date	End date	Duration Days	Days from inoculation	Whether within 30min	Severity	Treatment situation	Correlation
Subject No.: 0453, Group: Recombinant vaccine group, Age (years): 36.3, Sex: Male, Nationality: Uganda												
Fever: 37.8 ℃	General disorders and administration site conditions	fever	Recruited//Non-Vaccination Site (Systemic)	2021-11-03	2021-11-03	November 5, 2021	3	0	No	Level 1	No treatment	Definitely relevant
Cough	Respiratory, thoracic and mediastinal diseases	Cough	Collection//Non-inoculation site (whole body)	2021-11-03	2021-11-03	2021-11-06	4	0	No	Grade 1	Untreated	Probably related
Subject No.: 0455, Group: Recombinant Vaccine Group, Age (years): 35.3, Sex: Male, Nationality: Bangladesh												
Muscle pain (non-injection site)	Various musculoskeletal and connective tissue diseases	Myalgia	Collection//Non-inoculation site (whole body)	2021-11-03	November 4, 2021	2021-11-16	13	1	No	Grade 2	Untreated	Probably related
Subject No.: 0456, Group: Recombinant Vaccine Group, Age (years): 25.7, Sex: Male, Nationality: Pakistan												
Headache	Various nervous system diseases	Headache	Collection//Non-inoculation site (whole body)	2021-11-03	2021-11-03	2021-11-04	2	0	No	Grade 2	Self-medication	Probably related
Subject No.: 0460, Group: Recombinant Vaccine Group, Age (years): 35.1, Sex: Male, Nationality: Pakistan												
Headache	Various nervous system diseases	Headache	Collection//Non-inoculation site (whole body)	2021-11-03	2021-11-03	2021-11-06	4	0	No	Grade 2	Self-medication	Probably related
Subject No.: 0461, Group: Recombinant Vaccine Group, Age (years): 40.1, Sex: Female, Nationality: Syria												
Pain	Systemic diseases and various reactions at the administration site	Vaccination site pain	Collection//inoculation site (local)	2021-11-03	2021-11-03	2021-11-04	2	0	No	Grade 2	Self-medication	Definitely related
Subject No.: 0470, Group: Inactivated Vaccine Group, Age (years): 42.3, Sex: Male, Nationality: Nepal												
Pain	Systemic diseases and various reactions at the administration site	Vaccination site pain	Collection//inoculation site (local)	2021-11-03	2021-11-03	2021-11-05	3	0	No	Grade 1	Untreated	Definitely related
Subject No.: 0471, Group: Recombinant Vaccine Group, Age (years): 33.9, Sex: Male, Nationality: Nepal												
reduced sensing in left arm	Various nervous system diseases	Hypoaesthesia	Non-solicitation	2021-11-03	November 4, 2021	2021-11-05	2	1	No	Grade 1	Untreated	Possibly related

Adverse event description	System Organ Class	Preferred terms	Type of Adverse Event	Date of inoculation	Start Date	End date	Duration Days	Days from inoculation	Whether within 30min	Severity	Treatment situation	Correlation
Headache	Nervous system disorders	Headache	Recruited//Non-Vaccination Site (Systemic)	2021-11-03	2021-11-06	2021-11-09	4	3	No	Level 2	Self-medication	May be relevant
Nausea	Gastrointestinal system diseases	Nausea	Collection//Non-inoculation site (whole body)	2021-11-03	2021-11-06	2021-11-08	3	3	No	Grade 1	Untreated	Possibly related
Vomiting	Gastrointestinal disorders	Vomiting	Recruited//Non-Vaccination Site (Systemic)	2021-11-03	2021-11-08	2021-11-08	1	5	No	Level 1	No treatment	May be relevant
chest pain	Systemic diseases and various reactions at the administration site	Chest pain	Non-solicitation	2021-11-03	November 10, 2021	2021-11-10	1	7	No	Grade 1	Untreated	Possibly related
Subject No.: 0474, Group: Recombinant Vaccine Group, Age (years): 31.2, Sex: Male, Nationality: Uganda												
Muscle pain (non-injection site)	Various musculoskeletal and connective tissue diseases	Myalgia	Collection//Non-inoculation site (whole body)	2021-11-03	2021-11-07	2021-11-07	1	4	No	Grade 1	Self-medication	Possibly related
Fatigue/fatigue	General disorders and administration site conditions	Fatigue	Recruited//Non-Vaccination Site (Systemic)	2021-11-03	2021-11-08	2021-11-09	2	5	No	Level 1	Self-medication	May be relevant
Headache	Various nervous system diseases	Headache	Collection//Non-inoculation site (whole body)	2021-11-03	2021-11-09	2021-11-09	1	6	No	Grade 2	Self-medication	Possibly related
Subject No.: 0477, Group: Recombinant Vaccine Group, Age (years): 45.4, Sex: Male, Nationality: Uganda												
Dry Mouth	Gastrointestinal system diseases	Dry mouth	Non-solicitation	2021-11-03	November 5, 2021	2021-11-07	3	2	No	Grade 1	Untreated	Possibly related
Swelling in the Axilla	General disorders and administration site conditions	Swelling	Unsolicited	2021-11-03	2021-11-05	2021-11-07	3	2	No	Level 1	No medication in outpatient clinic	May be relevant
Subject No.: 0479, Group: Inactivated Vaccine Group, Age (years): 44.6, Sex: Male, Nigeria												
coughing	Respiratory, thoracic and mediastinal disorders	coughing	Recruited//Non-Vaccination Site (Systemic)	2021-11-03	2021-11-09	2021-11-09	1	6	No	Level 1	No treatment	Probably related

Adverse event description	System Organ Class	Preferred terms	Type of Adverse Event	Date of inoculation	Start Date	End date	Duration Days	Days from inoculation	Whether within 30min	Severity	Treatment situation	Correlation
Headache	Various nervous system diseases	Headache	Collection//Non-inoculation site (whole body)	2021-11-03	2021-11-09	2021-11-09	1	6	No	Grade 2	Self-medication	Probably related
Subject No.: 0480, Group: Inactivated Vaccine Group, Age (years): 32.4, Sex: Male, Nationality: Pakistan												
Fever: 37.8℃, taken panadol 500mg, 1 tab	Systemic diseases and various reactions at the administration site	Fever	Non-solicitation	2021-11-03	December 2, 2021	December 3, 2021	2	29	No	Grade 1	Self-medication	Possibly related
Runny nose	Respiratory, thoracic and mediastinal disorders	Runny nose	Unsolicited	2021-11-03	December 2, 2021	2021-12-09	8	29	No	Level 1	No treatment	Probably related
Subject No.: 0498, Group: Inactivated Vaccine Group, Age (years): 26.9, Sex: Male, Nationality: Uganda												
pain	General disorders and administration site conditions	Pain at vaccination site	Recruitment//injection site (local)	2021-11-03	2021-11-03	2021-11-06	4	0	No	Level 1	No treatment	Definitely relevant
Subject No.: 0509, Group: Recombinant vaccine group, Age (years): 33.3, Sex: Male, Nationality: Bangladesh												
Fever: 38.0 ℃	General disorders and administration site conditions	fever	Recruited//Non-Vaccination Site (Systemic)	2021-11-03	2021-11-08	2021-11-09	2	5	No	Level 1	Self-medication	Probably related
Subject No.: 0512, Group: Inactivated Vaccine Group, Age (years): 32.7, Sex: Male, Nationality: Nepal												
Nausea	Gastrointestinal disorders	Nausea	Recruited//Non-Vaccination Site (Systemic)	2021-11-03	2021-11-03	November 4, 2021	2	0	No	Level 1	No treatment	Probably related
Fatigue/Asthenia	Systemic diseases and various reactions at the administration site	Fatigue	Collection//Non-inoculation site (whole body)	2021-11-03	2021-11-03	2021-11-06	4	0	No	Grade 1	Untreated	Probably related
Subject No.: 0514, Group: Recombinant Vaccine Group, Age (years): 27.7, Sex: Male, Nationality: Pakistan												
redness on bot eyes	Ocular organ disease	Ocular hyperemia	Non-solicitation	2021-11-03	2021-11-03	2021-11-05	3	0	No	Grade 1	Untreated	Probably related

Adverse event description	System Organ Class	Preferred terms	Type of Adverse Event	Date of inoculation	Start Date	End date	Duration Days	Days from inoculation	Whether within 30min	Severity	Treatment situation	Correlation
Fatigue/fatigue	General disorders and administration site conditions	Fatigue	Recruited//Non-Vaccination Site (Systemic)	2021-11-03	2021-11-04	November 4, 2021	1	1	No	Level 1	No treatment	May be relevant
Subject No.: 0526, Group: Inactivated Vaccine Group, Age (years): 35.8, Sex: Male, Nationality: Uganda												
Cold Symptoms	Infections and infestations	Nasopharyngitis	Unsolicited	2021-11-03	2021-11-05	2021-11-06	2	2	No	Level 1	No medication in outpatient clinic	May be relevant
Subject No.: 0539, Group: Recombinant vaccine group, Age (years): 31.8, Sex: Male, Nationality: Pakistan												
Nasal conjecture	Respiratory, thoracic and mediastinal disorders	Nasal obstruction	Unsolicited	2021-11-03	2021-11-05	2021-11-08	4	2	No	Level 1	No treatment	Probably related
Subject No.: 0540, Group: Inactivated Vaccine Group, Age (years): 39.8, Sex: Male, Nationality: Pakistan												
pain	General disorders and administration site conditions	Pain at vaccination site	Recruitment//injection site (local)	2021-11-03	2021-11-03	November 4, 2021	2	0	No	Level 1	No treatment	Definitely relevant
Subject No.: 0547, Group: Recombinant Vaccine Group, Age (Y): 43.5, Sex: Male, Nationality: Bangladesh												
Body pain	General disorders and administration site conditions	pain	Unsolicited	November 4, 2021	2021-11-07	November 11, 2021	5	3	No	Level 1	No medication in outpatient clinic	May be relevant
Cough	Respiratory, thoracic and mediastinal diseases	Cough	Collection//Non-inoculation site (whole body)	2021-11-04	2021-11-07	2021-11-11	5	3	No	Grade 1	No medication in outpatient department	Possibly related
Subject No.: 0555, Group: Inactivated Vaccine Group, Age (years): 29.7, Sex: Male, Nationality: India												
Cough	Respiratory, thoracic and mediastinal diseases	Cough	Non-solicitation	2021-11-04	2021-11-21	2021-11-26	6	17	No	Grade 2	Prescription drug	Possibly related
Subject No.: 0578, Group: Recombinant Vaccine Group, Age (years): 40.2, Sex: Male, Nationality: Bangladesh												
Fever: 37.8℃, Tympanic	Systemic diseases and various reactions at the administration site	Fever	Collection//Non-inoculation site (whole body)	2021-11-04	November 4, 2021	2021-11-05	2	0	No	Grade 1	Self-medication	Probably related

Adverse event description	System Organ Class	Preferred terms	Type of Adverse Event	Date of inoculation	Start Date	End date	Duration Days	Days from inoculation	Whether within 30min	Severity	Treatment situation	Correlation
Subject No.: 0582, Group: Inactivated Vaccine Group, Age (years): 48.1, Sex: Male, Nationality: Bangladesh												
Fever: 37.8℃, Tympanic	Systemic diseases and various reactions at the administration site	Fever	Collection//Non-inoculation site (whole body)	2021-11-04	2021-11-06	2021-11-07	2	2	No	Grade 1	Untreated	Possibly related
Subject No.: 0583, Group: Recombinant Vaccine Group, Age (years): 32.4, Sex: Male, Nationality: India												
Headache	Various nervous system diseases	Headache	Collection//Non-inoculation site (whole body)	2021-11-04	November 4, 2021	2021-11-07	4	0	No	Grade 2	Untreated	Probably related
Subject No.: 0588, Group: Inactivated Vaccine Group, Age (years): 41.8, Sex: Male, Nationality: Sudan												
Pain	Systemic diseases and various reactions at the administration site	Vaccination site pain	Collection//inoculation site (local)	2021-11-04	November 4, 2021	2021-11-08	5	0	No	Grade 2	Untreated	Definitely related
Swelling	General disorders and administration site conditions	Swelling of vaccination site	Recruitment//injection site (local)	November 4, 2021	2021-11-04	2021-11-08	5	0	No	Level 1	No treatment	Definitely relevant
Fever: 39.0℃	Systemic diseases and various reactions at the administration site	Fever	Collection//Non-inoculation site (whole body)	2021-11-04	November 5, 2021	2021-11-06	2	1	No	Grade 3	Outpatient medication	Probably related
Dyspnea	Respiratory, thoracic and mediastinal disorders	Dyspnea	Recruited//Non-Vaccination Site (Systemic)	November 4, 2021	2021-11-05	November 5, 2021	1	1	No	Level 1	No treatment	Probably related
Cough	Respiratory, thoracic and mediastinal diseases	Cough	Collection//Non-inoculation site (whole body)	2021-11-04	November 5, 2021	2021-11-06	2	1	No	Grade 2	Outpatient medication	Probably related
Headache	Nervous system disorders	Headache	Recruited//Non-Vaccination Site (Systemic)	November 4, 2021	2021-11-05	2021-11-08	4	1	No	Level 2	Outpatient medication	Probably related
Fatigue/Asthenia	Systemic diseases and various reactions at the administration site	Fatigue	Collection//Non-inoculation site (whole body)	2021-11-04	November 5, 2021	2021-11-08	4	1	No	Grade 2	Outpatient medication	Probably related

Adverse event description	System Organ Class	Preferred terms	Type of Adverse Event	Date of inoculation	Start Date	End date	Duration Days	Days from inoculation	Whether within 30min	Severity	Treatment situation	Correlation
Muscle pain (non-inoculation site)	Musculoskeletal and connective tissue disorders	Myalgia	Recruited//Non-Vaccination Site (Systemic)	November 4, 2021	2021-11-05	2021-11-08	4	1	No	Grade 3	Outpatient medication	Probably related
Runny Nose	Respiratory, thoracic and mediastinal diseases	Runny nose	Non-solicitation	2021-11-04	2021-11-06	2021-11-08	3	2	No	Grade 2	Prescription drug	Possibly related
Sneezing	Respiratory, thoracic and mediastinal disorders	Sneeze	Unsolicited	November 4, 2021	2021-11-06	2021-11-08	3	2	No	Level 2	Prescription Drug	May be relevant
Vomiting	Gastrointestinal system diseases	Vomiting	Collection//Non-inoculation site (whole body)	2021-11-04	2021-11-06	2021-11-06	1	2	No	Grade 1	Untreated	Probably related
Subject No.: 0591, Group: Recombinant Vaccine Group, Age (years): 33.4, Sex: Male, Nationality: Philippines												
Epigastric pain	Gastrointestinal system diseases	Abdominal pain upper	Non-solicitation	2021-11-04	November 5, 2021	2021-11-07	3	1	No	Grade 2	Outpatient medication	Probably related
Vomiting	Gastrointestinal disorders	Vomiting	Recruited//Non-Vaccination Site (Systemic)	November 4, 2021	2021-11-05	2021-11-07	3	1	No	Level 2	Outpatient medication	Probably related
Nausea	Gastrointestinal system diseases	Nausea	Collection//Non-inoculation site (whole body)	2021-11-04	November 5, 2021	2021-11-06	2	1	No	Grade 2	Outpatient medication	Possibly related
Subject No.: 0594, Group: Recombinant Vaccine Group, Age (years): 32.4, Sex: Male, Nationality: Bangladesh												
Myalgia	Various musculoskeletal and connective tissue diseases	Myalgia	Non-solicitation	2021-11-04	November 12, 2021			8	No	Grade 2	Prescription drug	Probably related
Cough	Respiratory, thoracic and mediastinal disorders	coughing	Unsolicited	November 4, 2021	2021-11-22	November 26, 2021	5	18	No	Level 2	Prescription Drug	May be relevant
Dizziness	Various nervous system diseases	Dizziness	Non-solicitation	2021-11-04	November 22, 2021	2021-11-26	5	18	No	Grade 2	Prescription drug	Probably related

Adverse event description	System Organ Class	Preferred terms	Type of Adverse Event	Date of inoculation	Start Date	End date	Duration Days	Days from inoculation	Whether within 30min	Severity	Treatment situation	Correlation
Dyspnea	Respiratory, thoracic and mediastinal disorders	Dyspnea	Unsolicited	November 4, 2021	2021-11-22	November 27, 2021	6	18	No	Level 2	Prescription Drug	Probably related
Fever: 38.0℃, on Prescribed medicine	Systemic diseases and various reactions at the administration site	Fever	Non-solicitation	2021-11-04	November 22, 2021	2021-11-26	5	18	No	Grade 1	Prescription drug	Probably related
Myalgia	Musculoskeletal and connective tissue disorders	Myalgia	Unsolicited	November 4, 2021	2021-11-22	November 26, 2021	5	18	No	Level 2	Prescription Drug	Probably related
Runny Nose	Respiratory, thoracic and mediastinal diseases	Runny nose	Non-solicitation	2021-11-04	November 22, 2021	2021-11-26	5	18	No	Grade 2	Prescription drug	Probably related
Sore Throat	Respiratory, thoracic and mediastinal disorders	Oropharyngeal pain	Unsolicited	November 4, 2021	2021-11-22	November 26, 2021	5	18	No	Level 2	Prescription Drug	Probably related
Subject No.: 0598, Group: Recombinant vaccine group, Age (years): 37.2, Sex: Male, Nationality: Bangladesh												
coughing	Respiratory, thoracic and mediastinal disorders	coughing	Recruited//Non-Vaccination Site (Systemic)	November 4, 2021	2021-11-04	2021-11-06	3	0	No	Level 1	No treatment	Probably related
Subject No.: 0600, Group: Inactivated Vaccine Group, Age (years): 28.4, Sex: Male, Nationality: Pakistan												
Headache	Nervous system disorders	Headache	Recruited//Non-Vaccination Site (Systemic)	November 4, 2021	2021-11-04	November 5, 2021	2	0	No	Level 2	Self-medication	Probably related
Subject No.: 1205, Group: Inactivated Vaccine Group, Age (years): 18.8, Sex: Male, Nationality: Egypt												
Mild swelling	General disorders and administration site conditions	Swelling	Unsolicited	October 23, 2021	2021-10-23	October 25, 2021	3	0	No	Level 1	No treatment	May be relevant
pain with mild swelling	Systemic diseases and various reactions at the administration site	Pain	Non-solicitation	2021-10-23	October 23, 2021	2021-10-25	3	0	No	Grade 1	Untreated	Possibly related

Adverse event description	System Organ Class	Preferred terms	Type of Adverse Event	Date of inoculation	Start Date	End date	Duration Days	Days from inoculation	Whether within 30min	Severity	Treatment situation	Correlation
Headache	Nervous system disorders	Headache	Unsolicited	October 23, 2021	2021-11-06	November 25, 2021	20	14	No	Level 2	Self-medication	Probably related
Subject No.: 1213, Group: Inactivated Vaccine Group, Age (years): 42.4, Sex: Male, Nationality: India												
Headache	Nervous system disorders	Headache	Recruited//Non-Vaccination Site (Systemic)	October 24, 2021	2021-10-26	2021-10-26	1	2	No	Level 2	Self-medication	May be relevant
Pruritus	Skin and subcutaneous tissue diseases	Pruritus	Collection//inoculation site (local)	2021-10-24	October 28, 2021	2021-10-31	4	4	No	Grade 2	Self-medication	Possibly related
Subject No.: 1216, Group: Inactivated Vaccine Group, Age (years): 36.9, Sex: Male, Nationality: Pakistan												
Headache	Various nervous system diseases	Headache	Collection//Non-inoculation site (whole body)	2021-10-24	October 24, 2021	2021-10-26	3	0	No	Grade 1	Untreated	Possibly related
Subject number: 1222, group: recombinant vaccine group, age (years): 29.1, sex: male, nationality: Afghanistan												
Runny Nose	Respiratory, thoracic and mediastinal diseases	Runny nose	Non-solicitation	2021-10-24	2021-10-26	2021-10-29	4	2	No	Grade 1	Untreated	Probably related
coughing	Respiratory, thoracic and mediastinal disorders	coughing	Recruited//Non-Vaccination Site (Systemic)	October 24, 2021	2021-10-26	October 29, 2021	4	2	No	Level 1	No treatment	Probably related
Subject No.: 1227, Group: Recombinant vaccine group, Age (years): 21, Sex: Male, Nationality: Pakistan												
Headache	Nervous system disorders	Headache	Recruited//Non-Vaccination Site (Systemic)	October 24, 2021	2021-10-24	October 31, 2021	8	0	No	Level 1	No treatment	May be relevant
Muscle pain (non-injection site)	Various musculoskeletal and connective tissue diseases	Myalgia	Collection//Non-inoculation site (whole body)	2021-10-24	October 24, 2021	2021-11-02	10	0	No	Grade 2	Untreated	Definitely related
Subject No.: 1228, Group: Recombinant Vaccine Group, Age (years): 25.3, Sex: Male, Nationality: Bangladesh												
Flushing	Systemic diseases and various reactions at the administration site	Vaccination site erythema	Collection//inoculation site (local)	2021-10-24	October 24, 2021	2021-10-26	3	0	No	Grade 1	Untreated	Definitely related

Adverse event description	System Organ Class	Preferred terms	Type of Adverse Event	Date of inoculation	Start Date	End date	Duration Days	Days from inoculation	Whether within 30min	Severity	Treatment situation	Correlation
Fever: 37.8 ℃, TEMPORAL	General disorders and administration site conditions	fever	Unsolicited	October 24, 2021	2021-11-17	November 18, 2021	2	24	No	Level 1	Self-medication	May be relevant
Subject No.: 1232, Group: Inactivated Vaccine Group, Age (years): 43.8, Sex: Male, Nationality: Pakistan												
Headache	Nervous system disorders	Headache	Recruited//Non-Vaccination Site (Systemic)	October 24, 2021	2021-10-25	2021-10-26	2	1	No	Level 1	No treatment	May be relevant
Subject No.: 1237, Group: Inactivated Vaccine Group, Age (years): 33.4, Sex: Male, Nationality: India												
Fever: 38.1 ℃, ON 25.10. 21 TEMPERATURE RECORDED WAS 37.8	General disorders and administration site conditions	fever	Recruited//Non-Vaccination Site (Systemic)	October 24, 2021	2021-10-24	October 25, 2021	2	0	No	Level 1	Self-medication	Definitely relevant
Subject No.: 1240, Group: Inactivated Vaccine Group, Age (years): 34.1, Sex: Male, Nationality: Pakistan												
Fever: 37.8 ℃	General disorders and administration site conditions	fever	Recruited//Non-Vaccination Site (Systemic)	October 24, 2021	2021-10-24	October 24, 2021	1	0	No	Level 1	No treatment	Definitely relevant
Subject No.: 1249, Group: Inactivated Vaccine Group, Age (years): 31.6, Sex: Male, Nationality: Bangladesh												
Fever: 37.8 ℃	General disorders and administration site conditions	fever	Recruited//Non-Vaccination Site (Systemic)	October 24, 2021	2021-10-24	2021-10-26	3	0	No	Level 1	Self-medication	Probably related
Subject No.: 1251, Group: Recombinant vaccine group, Age (years): 66.8, Sex: Male, Nationality: Pakistan												
Headache	Nervous system disorders	Headache	Recruited//Non-Vaccination Site (Systemic)	October 24, 2021	2021-10-25	October 27, 2021	3	1	No	Level 1	No treatment	May be relevant
Subject number: 1261, group: recombinant vaccine group, age (years): 46.3, gender: male, nationality: Sudan												
Headache	Nervous system disorders	Headache	Recruited//Non-Vaccination Site (Systemic)	October 24, 2021	2021-10-25	October 25, 2021	1	1	No	Level 2	Self-medication	Probably related
Subject No.: 1273, Group: Recombinant vaccine group, Age (years): 40.1, Sex: Male, Nationality: Pakistan												

Adverse event description	System Organ Class	Preferred terms	Type of Adverse Event	Date of inoculation	Start Date	End date	Duration Days	Days from inoculation	Whether within 30min	Severity	Treatment situation	Correlation
Headache	Nervous system disorders	Headache	Recruited//Non-Vaccination Site (Systemic)	October 24, 2021	2021-10-24	2021-10-26	3	0	No	Level 2	Self-medication	Probably related
Subject No.: 1282, Group: Inactivated Vaccine Group, Age (years): 24.3, Sex: Male, Nationality: India												
Muscle pain (non-inoculation site)	Musculoskeletal and connective tissue disorders	Myalgia	Recruited//Non-Vaccination Site (Systemic)	October 24, 2021	2021-10-27	October 29, 2021	3	3	No	Level 1	No treatment	May be relevant
Subject No.: 1288, Group: Inactivated Vaccine Group, Age (years): 40.4, Sex: Male, Nationality: India												
Fever: 37.8 ℃	General disorders and administration site conditions	fever	Recruited//Non-Vaccination Site (Systemic)	October 24, 2021	2021-10-24	October 25, 2021	2	0	No	Level 1	No medication in outpatient clinic	May be relevant
Cough	Respiratory, thoracic and mediastinal diseases	Cough	Collection//Non-inoculation site (whole body)	2021-10-24	October 24, 2021	2021-10-26	3	0	No	Grade 1	No medication in outpatient department	Possibly related
Headache	Nervous system disorders	Headache	Recruited//Non-Vaccination Site (Systemic)	October 24, 2021	2021-10-24	October 25, 2021	2	0	No	Level 1	No medication in outpatient clinic	May be relevant
Pain	Systemic diseases and various reactions at the administration site	Vaccination site pain	Collection//inoculation site (local)	2021-10-24	October 24, 2021	2021-10-25	2	0	No	Grade 1	No medication in outpatient department	Definitely related
Muscle pain (non-inoculation site)	Musculoskeletal and connective tissue disorders	Myalgia	Recruited//Non-Vaccination Site (Systemic)	October 24, 2021	2021-10-24	October 25, 2021	2	0	No	Level 1	No medication in outpatient clinic	May be relevant
Cough	Respiratory, thoracic and mediastinal diseases	Cough	Non-solicitation	2021-10-24	2021-11-07	2021-11-10	4	14	No	Grade 1	Untreated	Probably related
Fever: 37.8 ℃	General disorders and administration site conditions	fever	Unsolicited	October 24, 2021	2021-11-11	November 13, 2021	3	18	No	Level 1	No treatment	Probably related
Subject No.: 1289, Group: Recombinant vaccine group, Age (years): 33.1, Sex: Male, Nationality: India												

Adverse event description	System Organ Class	Preferred terms	Type of Adverse Event	Date of inoculation	Start Date	End date	Duration Days	Days from inoculation	Whether within 30min	Severity	Treatment situation	Correlation
Headache	Nervous system disorders	Headache	Recruited//Non-Vaccination Site (Systemic)	October 24, 2021	2021-10-24	October 25, 2021	2	0	No	Level 1	No medication in outpatient clinic	May be relevant
Subject No.: 1290, Group: Inactivated Vaccine Group, Age (years): 33.8, Sex: Male, Nationality: Pakistan												
Headache	Nervous system disorders	Headache	Recruited//Non-Vaccination Site (Systemic)	October 24, 2021	2021-10-27	October 31, 2021	5	3	No	Level 2	Outpatient medication	Probably related
Subject No.: 1293, Group: Recombinant vaccine group, Age (years): 35.8, Sex: Male, Nationality: Pakistan												
Fever: 37.9 ℃, Volunteer book Panadol 500mg. He had ever for 1 day.	General disorders and administration site conditions	fever	Recruited//Non-Vaccination Site (Systemic)	October 24, 2021	2021-10-25	2021-10-26	2	1	No	Level 1	Self-medication	May be relevant
Subject No.: 1297, Group: Inactivated Vaccine Group, Age (years): 39.7, Sex: Male, Nationality: Bangladesh												
Headache	Nervous system disorders	Headache	Recruited//Non-Vaccination Site (Systemic)	October 24, 2021	2021-10-24	October 25, 2021	2	0	No	Level 1	No treatment	May be relevant
Subject No.: 1298, Group: Recombinant vaccine group, Age (years): 36.8, Sex: Male, Nationality: Syria												
pain	General disorders and administration site conditions	Pain at vaccination site	Recruitment//injection site (local)	October 24, 2021	2021-10-24	2021-10-26	3	0	No	Level 1	No medication in outpatient clinic	Definitely relevant
Subject No.: 1324, Group: Recombinant vaccine group, Age (years): 28.5, Sex: Male, Nationality: Pakistan												
pain	General disorders and administration site conditions	Pain at vaccination site	Recruitment//injection site (local)	October 25, 2021	2021-10-25	2021-10-26	2	0	No	Level 2	Self-medication	Definitely relevant
Subject No.: 1328, Group: Inactivated Vaccine Group, Age (years): 36.9, Sex: Male, Nationality: India												
Fatigue/fatigue	General disorders and administration site conditions	Fatigue	Recruited//Non-Vaccination Site (Systemic)	October 25, 2021	2021-10-25	October 27, 2021	3	0	No	Level 1	No treatment	May be relevant
Subject No.: 1342, Group: Inactivated Vaccine Group, Age (years): 32.1, Sex: Female, Nationality: Philippines												

Adverse event description	System Organ Class	Preferred terms	Type of Adverse Event	Date of inoculation	Start Date	End date	Duration Days	Days from inoculation	Whether within 30min	Severity	Treatment situation	Correlation
DIZZINESS	Nervous system disorders	Dizziness	Unsolicited	October 25, 2021	2021-10-25	2021-10-26	2	0	No	Level 1	No treatment	Probably related
Fever: 37.8℃, CHECKED VIA TYMPANIC.TOOK PANADOL 500 MG PER TAB ORALLY, 2 TABLETS.	Systemic diseases and various reactions at the administration site	Fever	Collection//Non-inoculation site (whole body)	2021-10-25	October 25, 2021	2021-10-26	2	0	No	Grade 1	Self-medication	Possibly related
Sore troat	Respiratory, thoracic and mediastinal disorders	Oropharyngeal pain	Unsolicited	October 25, 2021	2021-10-26	October 27, 2021	2	1	No	Level 1	No treatment	Probably related
Cough	Respiratory, thoracic and mediastinal diseases	Cough	Collection//Non-inoculation site (whole body)	2021-10-25	2021-10-26	2021-11-01	7	1	No	Grade 2	Self-medication	Probably related
Subject number: 1349, group: recombinant vaccine group, age (years): 25.4, sex: female, nationality: Kazakhstan												
Vertigo	Various nervous system diseases	Dizziness	Collection//Non-inoculation site (whole body)	2021-10-25	October 25, 2021	2021-10-29	5	0	No	Grade 1	No medication in outpatient department	Possibly related
Subject number: 1358, group: recombinant vaccine group, age (years): 44.3, sex: male, nationality: Egypt												
Arthralgia	Various musculoskeletal and connective tissue diseases	Arthralgia	Collection//Non-inoculation site (whole body)	2021-10-25	October 27, 2021	2021-11-01	6	2	No	Grade 1	Untreated	Possibly related
Subject No.: 1360, Group: Inactivated Vaccine Group, Age (years): 34.3, Sex: Male, Nationality: Bangladesh												
Headache	Various nervous system diseases	Headache	Collection//Non-inoculation site (whole body)	2021-10-25	2021-10-26	2021-10-29	4	1	No	Grade 1	Untreated	Possibly related
Muscle pain (non-inoculation site)	Musculoskeletal and connective tissue disorders	Myalgia	Recruited//Non-Vaccination Site (Systemic)	October 25, 2021	2021-10-26	October 29, 2021	4	1	No	Level 1	No treatment	May be relevant

Adverse event description	System Organ Class	Preferred terms	Type of Adverse Event	Date of inoculation	Start Date	End date	Duration Days	Days from inoculation	Whether within 30min	Severity	Treatment situation	Correlation
Subject No.: 1362, Group: Inactivated Vaccine Group, Age (years): 41.5, Sex: Male, Nationality: India												
pain	General disorders and administration site conditions	Pain at vaccination site	Recruitment//injection site (local)	October 25, 2021	2021-10-25	October 29, 2021	5	0	No	Level 1	No treatment	Definitely relevant
Subject number: 1363, group: recombinant vaccine group, age (years): 30.3, gender: male, nationality: India												
COLD symptoms	Infections and infestations	Nasopharyngitis	Unsolicited	October 25, 2021	2021-10-25	October 27, 2021	3	0	No	Level 1	No treatment	May be relevant
Runny Nose	Respiratory, thoracic and mediastinal diseases	Runny nose	Non-solicitation	2021-10-25	2021-10-26	2021-10-29	4	1	No	Grade 1	Untreated	Possibly related
Subject No.: 1380, Group: Inactivated Vaccine Group, Age (years): 34.4, Sex: Male, Nationality: Bangladesh												
Fatigue/Asthenia	Systemic diseases and various reactions at the administration site	Fatigue	Collection//Non-inoculation site (whole body)	2021-10-26	2021-10-26	2021-11-10	16	0	No	Grade 1	Untreated	Probably related
MILD CHEST PAIN	General disorders and administration site conditions	Chest pain	Unsolicited	2021-10-26	2021-10-30	November 10, 2021	12	4	No	Level 1	No treatment	May be relevant
Subject number: 1383, group: recombinant vaccine group, age (years): 27.7, gender: male, nationality: Egypt												
Fatigue/fatigue	General disorders and administration site conditions	Fatigue	Recruited//Non-Vaccination Site (Systemic)	2021-10-26	2021-10-31	November 21-02, 2021	3	5	No	Level 2	Prescription Drug	May be relevant
Muscle pain (non-injection site)	Various musculoskeletal and connective tissue diseases	Myalgia	Collection//Non-inoculation site (whole body)	2021-10-26	October 31, 2021	2021-11-02	3	5	No	Grade 2	Prescription drug	Possibly related
Subject No.: 1404, Group: Inactivated Vaccine Group, Age (years): 33.4, Sex: Male, Nationality: Bangladesh												
Cough	Respiratory, thoracic and mediastinal diseases	Cough	Non-solicitation	2021-10-26	November 15, 2021	2021-11-18	4	20	No	Grade 2	Self-medication	Possibly related

Adverse event description	System Organ Class	Preferred terms	Type of Adverse Event	Date of inoculatio n	Start Date	End date	Dura tion Days	Days from inocula tion	Whethe r within 30min	Seve rity	Treatment situation	Correlatio n
Myalgia	Musculoskeletal and connective tissue disorders	Myalgia	Unsolicited	2021-10-2 6	2021-11-1 5	2021-11-2 1	7	20	No	Leve l 1	No treatment	Probably related
Sore Throat	Respiratory, thoracic and mediastinal diseases	Orophary ngeal pain	Non-solicitation	2021-10-2 6	November 15, 2021	2021-11-2 1	7	20	No	Grad e 1	Untreated	Probably related
Subjective ever	General disorders and administration site conditions	fever	Unsolicited	2021-10-2 6	2021-11-1 5	November 18, 2021	4	20	No	Leve l 2	Self-medication	May be relevant
Dyspnea	Respiratory, thoracic and mediastinal diseases	Difficulty breathing	Non-solicitation	2021-10-2 6	November 17, 2021	2021-11-2 1	5	22	No	Grad e 1	Untreated	Probably related
Subject number: 1423, group: recombinant vaccine group, age (years): 55.5, sex: female, nationality: India												
Pruritus	Skin and subcutaneous tissue diseases	Pruritus	Collection//inoculation site (local)	2021-10-2 6	October 28, 2021	2021-10-2 8	1	2	No	Grad e 1	Untreated	Possibly related
Eye irritation	Eye disorders	Ocular irritation	Unsolicited	2021-10-2 6	2021-11-0 1			6	No	Leve l 2	No treatment	Probably related
Subject No.: 1437, Group: Recombinant vaccine group, Age (years): 49.8, Sex: Male, Nationality: Pakistan												
Fever: 38.5 ℃	General disorders and administration site conditions	fever	Recruited//Non-Vaccinat ion Site (Systemic)	October 27, 2021	2021-10-3 0	October 31, 2021	2	3	No	Leve l 2	Self-medication	May be relevant
Cough	Respiratory, thoracic and mediastinal diseases	Cough	Collection//Non-inocula tion site (whole body)	2021-10-2 7	October 30, 2021	2021-10-3 1	2	3	No	Grad e 1	Untreated	Possibly related
Headache	Nervous system disorders	Headache	Recruited//Non-Vaccinat ion Site (Systemic)	October 27, 2021	2021-10-3 0	October 31, 2021	2	3	No	Leve l 2	Self-medication	May be relevant
Subject No.: 1456, Group: Inactivated Vaccine Group, Age (years): 24.3, Sex: Female, Nationality: India												
Sore throat.	Respiratory, thoracic and mediastinal disorders	Orophary ngeal pain	Unsolicited	October 27, 2021	2021-11-1 9	2021-11-2 1	3	23	No	Leve l 2	No treatment	May be relevant
Subject No.: 1466, Group: Recombinant vaccine group, Age (years): 32.7, Sex: Male, Nationality: Bangladesh												

Adverse event description	System Organ Class	Preferred terms	Type of Adverse Event	Date of inoculation	Start Date	End date	Duration Days	Days from inoculation	Whether within 30min	Severity	Treatment situation	Correlation
pain	General disorders and administration site conditions	Pain at vaccination site	Recruitment//injection site (local)	October 27, 2021	2021-11-02	2021-11-03	2	6	No	Level 1	No treatment	Definitely relevant
Subject No.: 1485, Group: Inactivated Vaccine Group, Age (years): 19.8, Sex: Male, Nationality: Pakistan												
Fever: 37.8 ℃	General disorders and administration site conditions	fever	Recruited//Non-Vaccination Site (Systemic)	October 31, 2021	2021-10-31	November 4, 2021	5	0	No	Level 1	Self-medication	Definitely relevant
Headache	Various nervous system diseases	Headache	Collection//Non-inoculation site (whole body)	2021-10-31	October 31, 2021	2021-11-04	5	0	No	Grade 2	Self-medication	Possibly related
Subject number: 1487, group: recombinant vaccine group, age (years): 32.6, sex: male, nationality: Bangladesh												
Pain	Systemic diseases and various reactions at the administration site	Vaccination site pain	Collection//inoculation site (local)	2021-10-31	October 31, 2021	2021-11-01	2	0	No	Grade 1	Untreated	Definitely related
Muscle pain (non-inoculation site)	Musculoskeletal and connective tissue disorders	Myalgia	Recruited//Non-Vaccination Site (Systemic)	October 31, 2021	2021-10-31	November 21-02, 2021	3	0	No	Level 1	No treatment	May be relevant
Subject number: 1488, group: recombinant vaccine group, age (years): 37.9, gender: male, nationality: Bangladesh												
pain	General disorders and administration site conditions	Pain at vaccination site	Recruitment//injection site (local)	October 31, 2021	2021-11-04	2021-11-06	3	4	No	Level 1	No treatment	Definitely relevant
Subject No.: 1493, Group: Inactivated Vaccine Group, Age (years): 31.8, Sex: Male, Nationality: Pakistan												
Muscle pain (non-inoculation site)	Musculoskeletal and connective tissue disorders	Myalgia	Recruited//Non-Vaccination Site (Systemic)	October 31, 2021	2021-10-31	October 31, 2021	1	0	No	Level 1	Self-medication	May be relevant
Subject No.: 1504, Group: Inactivated Vaccine Group, Age (years): 23.8, Sex: Male, Nationality: Bangladesh												
Nausea	Gastrointestinal disorders	Nausea	Recruited//Non-Vaccination Site (Systemic)	2021-11-01	2021-11-02	November 4, 2021	3	1	No	Level 1	No treatment	May be relevant
Subject number: 1507, group: recombinant vaccine group, age (years): 34.2, gender: male, nationality: India												

Adverse event description	System Organ Class	Preferred terms	Type of Adverse Event	Date of inoculation	Start Date	End date	Duration Days	Days from inoculation	Whether within 30min	Severity	Treatment situation	Correlation
pain	General disorders and administration site conditions	Pain at vaccination site	Recruitment//injection site (local)	2021-11-01	2021-11-02	2021-11-03	2	1	No	Level 1	No treatment	Definitely relevant
Subject number: 1512, group: recombinant vaccine group, age (years): 53.2, gender: male, nationality: Egypt												
pain	General disorders and administration site conditions	Pain at vaccination site	Recruitment//injection site (local)	2021-11-01	2021-11-02	2021-11-03	2	1	No	Level 1	No treatment	Definitely relevant
Subject No.: 1525, Group: Inactivated Vaccine Group, Age (years): 44.5, Sex: Female, Nationality: Syria												
Sleepiness	Nervous system disorders	Sleepiness	Unsolicited	2021-11-01	2021-11-02	November 4, 2021	3	1	No	Level 1	No treatment	Probably related
Headache	Various nervous system diseases	Headache	Collection//Non-inoculation site (whole body)	2021-11-01	November 21-02, 2021	2021-11-04	3	1	No	Grade 2	Self-medication	Probably related
Fatigue/fatigue	General disorders and administration site conditions	Fatigue	Recruited//Non-Vaccination Site (Systemic)	2021-11-01	2021-11-02	November 4, 2021	3	1	No	Level 1	No treatment	May be relevant
Fever: 38.0℃	Systemic diseases and various reactions at the administration site	Fever	Collection//Non-inoculation site (whole body)	2021-11-01	2021-11-03	2021-11-04	2	2	No	Grade 1	Self-medication	Probably related
Subject number: 1529, group: recombinant vaccine group, age (years): 24.8, sex: male, nationality: India												
Pain	Systemic diseases and various reactions at the administration site	Vaccination site pain	Collection//inoculation site (local)	2021-11-01	2021-11-01	2021-11-04	4	0	No	Grade 1	Untreated	Definitely related
Subject No.: 1538, Group: Inactivated Vaccine Group, Age (years): 27.9, Sex: Male, Nationality: Nepal												
Pain	Systemic diseases and various reactions at the administration site	Vaccination site pain	Collection//inoculation site (local)	2021-11-01	November 21-02, 2021	2021-11-02	1	1	No	Grade 1	Untreated	Definitely related
Subject No.: 1539, Group: Inactivated Vaccine Group, Age (years): 24.8, Sex: Male, Nationality: Nepal												

Adverse event description	System Organ Class	Preferred terms	Type of Adverse Event	Date of inoculation	Start Date	End date	Duration Days	Days from inoculation	Whether within 30min	Severity	Treatment situation	Correlation
Pain	Systemic diseases and various reactions at the administration site	Vaccination site pain	Collection//inoculation site (local)	2021-11-01	2021-11-01	2021-11-02	2	0	No	Grade 1	Untreated	Definitely related
Subject No.: 1540, Group: Inactivated Vaccine Group, Age (years): 28.5, Sex: Male, Nationality: Nepal												
runny nose	Respiratory, thoracic and mediastinal diseases	Runny nose	Non-solicitation	2021-11-01	November 21-02, 2021	2021-11-06	5	1	No	Grade 1	Untreated	Possibly related
Cough	Respiratory, thoracic and mediastinal disorders	coughing	Unsolicited	2021-11-01	2021-11-29			28	No	Level 2	Self-medication	May be relevant
Subject No.: 1541, Group: Recombinant vaccine group, Age (years): 32.2, Sex: Male, Nationality: Pakistan												
pain	General disorders and administration site conditions	Pain at vaccination site	Recruitment//injection site (local)	2021-11-01	2021-11-02	2021-11-03	2	1	No	Level 1	No treatment	Definitely relevant
Subject No.: 1542, Group: Inactivated Vaccine Group, Age (years): 37.3, Sex: Male, Nationality: Nepal												
Arthralgia	Musculoskeletal and connective tissue disorders	Arthralgia	Recruited//Non-Vaccination Site (Systemic)	2021-11-01	2021-11-01	2021-11-03	3	0	No	Level 1	No treatment	May be relevant
Muscle pain (non-injection site)	Various musculoskeletal and connective tissue diseases	Myalgia	Collection//Non-inoculation site (whole body)	2021-11-01	2021-11-01	2021-11-03	3	0	No	Grade 1	Untreated	Possibly related
Subject number: 1544, group: recombinant vaccine group, age (years): 31.8, sex: male, nationality: Pakistan												
Dizzy	Various nervous system diseases	Dizziness	Non-solicitation	2021-11-01	2021-11-01	2021-11-05	5	0	No	Grade 1	Untreated	Possibly related
Stomach ache	Gastrointestinal disorders	Epigastric pain	Unsolicited	2021-11-01	2021-11-01	November 4, 2021	4	0	No	Level 2	Prescription Drug	May be relevant
Headache	Various nervous system diseases	Headache	Collection//Non-inoculation site (whole body)	2021-11-01	2021-11-01	2021-11-05	5	0	No	Grade 2	Prescription drug	Possibly related

Adverse event description	System Organ Class	Preferred terms	Type of Adverse Event	Date of inoculation	Start Date	End date	Duration Days	Days from inoculation	Whether within 30min	Severity	Treatment situation	Correlation
Fatigue/fatigue	General disorders and administration site conditions	Fatigue	Recruited//Non-Vaccination Site (Systemic)	2021-11-01	2021-11-01	November 5, 2021	5	0	No	Level 2	Prescription Drug	May be relevant
Subject No.: 1548, Group: Inactivated Vaccine Group, Age (years): 45.9, Sex: Male, Nationality: Uganda												
Headache	Nervous system disorders	Headache	Recruited//Non-Vaccination Site (Systemic)	2021-11-01	2021-11-02	2021-11-06	5	1	No	Level 2	Self-medication	Probably related
sneezing	Respiratory, thoracic and mediastinal diseases	Sneezing	Non-solicitation	2021-11-01	2021-11-03	2021-11-06	4	2	No	Grade 1	Untreated	Possibly related
Fatigue/fatigue	General disorders and administration site conditions	Fatigue	Recruited//Non-Vaccination Site (Systemic)	2021-11-01	2021-11-03	2021-11-06	4	2	No	Level 2	Self-medication	Probably related
Subject number: 1549, group: recombinant vaccine group, age (years): 39.5, gender: male, nationality: Bangladesh												
pain	General disorders and administration site conditions	Pain at vaccination site	Recruitment//injection site (local)	November 21-02, 2021	2021-11-03	2021-11-03	1	1	No	Level 1	Self-medication	Definitely relevant
Subject number: 1553, group: recombinant vaccine group, age (years): 32.6, gender: female, nationality: Philippines												
Muscle pain (non-inoculation site)	Musculoskeletal and connective tissue disorders	Myalgia	Recruited//Non-Vaccination Site (Systemic)	November 21-02, 2021	2021-11-02	November 21-02, 2021	1	0	No	Level 1	Self-medication	May be relevant
Mouth Sore	Gastrointestinal system diseases	Oral pain	Non-solicitation	2021-11-02	2021-11-06	2021-11-10	5	4	No	Grade 1	Untreated	Probably related
Subject No.: 1555, Group: Recombinant Vaccine Group, Age (years): 35.7, Sex: Male, Nationality: Bangladesh												
Myalgia	Various musculoskeletal and connective tissue diseases	Myalgia	Non-solicitation	2021-11-02	November 27, 2021	2021-11-29	3	25	No	Grade 3	Prescription drug	Probably related
Subject No.: 1563, Group: Recombinant Vaccine Group, Age (years): 34.7, Sex: Male, Nationality: Bangladesh												

Adverse event description	System Organ Class	Preferred terms	Type of Adverse Event	Date of inoculation	Start Date	End date	Duration Days	Days from inoculation	Whether within 30min	Severity	Treatment situation	Correlation
Pain	Systemic diseases and various reactions at the administration site	Vaccination site pain	Collection//inoculation site (local)	2021-11-02	November 4, 2021	2021-11-04	1	2	No	Grade 1	Untreated	Definitely related
Subject number: 1568, group: inactivated vaccine group, age (years): 37.3, sex: male, nationality: Eritrea												
Pain	Systemic diseases and various reactions at the administration site	Vaccination site pain	Collection//inoculation site (local)	2021-11-02	November 21-02, 2021	2021-11-02	1	0	No	Grade 1	Untreated	Definitely related
Subject No.: 1573, Group: Recombinant Vaccine Group, Age (years): 33.2, Sex: Male, Nationality: Bangladesh												
Pain	Systemic diseases and various reactions at the administration site	Vaccination site pain	Collection//inoculation site (local)	2021-11-02	November 21-02, 2021	2021-11-08	7	0	No	Grade 1	Untreated	Definitely related
Stomach burning sensation	Gastrointestinal disorders	Dyspepsia	Unsolicited	November 21-02, 2021	2021-11-03	2021-11-08	6	1	No	Level 1	No treatment	May be relevant
Fever: 38.1℃, temporal, today temperature is 37.1	Systemic diseases and various reactions at the administration site	Fever	Collection//Non-inoculation site (whole body)	2021-11-02	2021-11-03	2021-11-05	3	1	No	Grade 1	Self-medication	Probably related
Headache	Nervous system disorders	Headache	Recruited//Non-Vaccination Site (Systemic)	November 21-02, 2021	2021-11-03	November 5, 2021	3	1	No	Level 2	Self-medication	May be relevant
Fatigue/Asthenia	Systemic diseases and various reactions at the administration site	Fatigue	Collection//Non-inoculation site (whole body)	2021-11-02	2021-11-03	2021-11-08	6	1	No	Grade 2	Self-medication	Possibly related
Muscle pain (non-inoculation site)	Musculoskeletal and connective tissue disorders	Myalgia	Recruited//Non-Vaccination Site (Systemic)	November 21-02, 2021	2021-11-03	2021-11-08	6	1	No	Level 2	Self-medication	Probably related

Adverse event description	System Organ Class	Preferred terms	Type of Adverse Event	Date of inoculation	Start Date	End date	Duration Days	Days from inoculation	Whether within 30min	Severity	Treatment situation	Correlation
Sore Throat	Respiratory, thoracic and mediastinal diseases	Oropharyngeal pain	Non-solicitation	2021-11-02	2021-11-06	2021-11-08	3	4	No	Grade 2	Self-medication	Possibly related
Subject number: 1575, group: recombinant vaccine group, age (years): 29.4, sex: male, nationality: Nepal												
Fatigue/Asthenia	Systemic diseases and various reactions at the administration site	Fatigue	Collection//Non-inoculation site (whole body)	2021-11-02	2021-11-03	2021-11-03	1	1	No	Grade 1	Untreated	Probably related
Subject number: 1586, group: recombinant vaccine group, age (years): 40, sex: male, nationality: Bangladesh												
feels sleepy ways	Various nervous system diseases	Sleepiness	Non-solicitation	2021-11-02	November 21-02, 2021	2021-11-12	11	0	No	Grade 1	Untreated	Possibly related
Headache	Nervous system disorders	Headache	Recruited//Non-Vaccination Site (Systemic)	November 21-02, 2021	2021-11-02	November 19, 2021	18	0	No	Level 1	No treatment	Probably related
Subject No.: 1594, Group: Recombinant vaccine group, Age (years): 35.4, Sex: Male, Nationality: Zimbabwe												
Fatigue/fatigue	General disorders and administration site conditions	Fatigue	Recruited//Non-Vaccination Site (Systemic)	November 21-02, 2021	2021-11-03	2021-11-03	1	1	No	Level 2	Self-medication	Probably related
clopged nose	Respiratory, thoracic and mediastinal diseases	Nasal congestion	Non-solicitation	2021-11-02	November 5, 2021	2021-11-08	4	3	No	Grade 1	Untreated	Probably related
Feeling of hotness and runny nose	General disorders and administration site conditions	Feeling of fever	Unsolicited	November 21-02, 2021	2021-11-05	2021-11-07	3	3	No	Level 2	Self-medication	May be relevant
Feeling of hotness and runny nose	Respiratory, thoracic and mediastinal diseases	Runny nose	Non-solicitation	2021-11-02	November 5, 2021	2021-11-07	3	3	No	Grade 1	Self-medication	Possibly related

Adverse event description	System Organ Class	Preferred terms	Type of Adverse Event	Date of inoculation	Start Date	End date	Duration Days	Days from inoculation	Whether within 30min	Severity	Treatment situation	Correlation
coughing	Respiratory, thoracic and mediastinal disorders	coughing	Recruited//Non-Vaccination Site (Systemic)	November 21-02, 2021	2021-11-05	2021-11-08	4	3	No	Level 2	Self-medication	Probably related
Headache	Various nervous system diseases	Headache	Collection//Non-inoculation site (whole body)	2021-11-02	November 5, 2021	2021-11-08	4	3	No	Grade 2	Self-medication	Probably related
Fatigue/fatigue	General disorders and administration site conditions	Fatigue	Recruited//Non-Vaccination Site (Systemic)	November 21-02, 2021	2021-11-05	2021-11-08	4	3	No	Level 1	Self-medication	Probably related
Muscle pain (non-injection site)	Various musculoskeletal and connective tissue diseases	Myalgia	Collection//Non-inoculation site (whole body)	2021-11-02	2021-11-07	2021-11-07	1	5	No	Grade 1	Self-medication	Possibly related
Subject number: 1597, group: recombinant vaccine group, age (years): 53.3, sex: male, nationality: Pakistan												
runny nose	Respiratory, thoracic and mediastinal diseases	Runny nose	Non-solicitation	2021-11-02	2021-11-03	2021-11-03	1	1	No	Grade 1	Untreated	Probably related
Subject No.: 1598, Group: Recombinant Vaccine Group, Age (years): 50.8, Sex: Male, Nationality: Bangladesh												
Sleepiness	Various nervous system diseases	Sleepiness	Non-solicitation	2021-11-02	November 21-02, 2021	2021-11-05	4	0	No	Grade 1	Untreated	Probably related
pain	General disorders and administration site conditions	Pain at vaccination site	Recruitment//injection site (local)	November 21-02, 2021	2021-11-02	November 4, 2021	3	0	No	Level 2	Self-medication	Definitely relevant
Subject No.: 1607, Group: Inactivated Vaccine Group, Age (years): 31.8, Sex: Male, Nationality: Bangladesh												
Fatigue/fatigue	General disorders and administration site conditions	Fatigue	Recruited//Non-Vaccination Site (Systemic)	2021-11-03	2021-11-03	November 4, 2021	2	0	No	Level 1	No treatment	May be relevant
Subject No.: 1620, Group: Inactivated Vaccine Group, Age (years): 38.5, Sex: Male, Nationality: Bangladesh												
Sleeplessness	Psychiatric disorders	Insomnia	Unsolicited	2021-11-03	2021-11-03	2021-11-07	5	0	No	Level 1	No treatment	Probably related

Adverse event description	System Organ Class	Preferred terms	Type of Adverse Event	Date of inoculation	Start Date	End date	Duration Days	Days from inoculation	Whether within 30min	Severity	Treatment situation	Correlation
Subject No.: 1623, Group: Inactivated Vaccine Group, Age (years): 33.8, Sex: Male, Nationality: Pakistan												
cold	Infections and infestations	Nasopharyngitis	Unsolicited	2021-11-03	2021-11-04	2021-11-07	4	1	No	Level 1	No medication in outpatient clinic	May be relevant
Cough	Respiratory, thoracic and mediastinal diseases	Cough	Collection//Non-inoculation site (whole body)	2021-11-03	November 4, 2021	2021-11-07	4	1	No	Grade 1	No medication in outpatient department	Possibly related
Subject No.: 1624, Group: Recombinant Vaccine Group, Age (years): 43.7, Sex: Male, Nationality: Bangladesh												
sleeplessness	Psychiatric category	Insomnia	Non-solicitation	2021-11-03	2021-11-03	2021-11-06	4	0	No	Grade 1	Untreated	Probably related
Headache	Nervous system disorders	Headache	Recruited//Non-Vaccination Site (Systemic)	2021-11-03	2021-11-03	2021-11-06	4	0	No	Level 1	No treatment	Probably related
Subject number: 1626, group: recombinant vaccine group, age (years): 40.8, gender: male, nationality: Bangladesh												
pain	General disorders and administration site conditions	Pain at vaccination site	Recruitment//injection site (local)	2021-11-03	2021-11-03	November 5, 2021	3	0	No	Level 1	No treatment	Definitely relevant
Subject No.: 1627, Group: Inactivated Vaccine Group, Age (years): 42, Sex: Male, Nationality: Pakistan												
Fatigue/fatigue	General disorders and administration site conditions	Fatigue	Recruited//Non-Vaccination Site (Systemic)	2021-11-03	2021-11-03	November 4, 2021	2	0	No	Level 1	No treatment	Probably related
Subject number: 1630, group: recombinant vaccine group, age (years): 44.6, gender: male, nationality: Bangladesh												
Feeling of hotness	General disorders and administration site conditions	Feeling of fever	Unsolicited	2021-11-03	2021-11-06	2021-11-07	2	3	No	Level 1	No treatment	May be relevant
Cough	Respiratory, thoracic and mediastinal diseases	Cough	Collection//Non-inoculation site (whole body)	2021-11-03	2021-11-06	2021-11-07	2	3	No	Grade 1	Untreated	Possibly related
Subject No.: 1632, Group: Recombinant Vaccine Group, Age (years): 30.7, Sex: Male, Nationality: Egypt												

Adverse event description	System Organ Class	Preferred terms	Type of Adverse Event	Date of inoculation	Start Date	End date	Duration Days	Days from inoculation	Whether within 30min	Severity	Treatment situation	Correlation
Pain	Systemic diseases and various reactions at the administration site	Vaccination site pain	Collection//inoculation site (local)	2021-11-03	2021-11-03	2021-11-03	1	0	No	Grade 1	Untreated	Definitely related
Subject No.: 1635, Group: Inactivated Vaccine Group, Age (years): 31.2, Sex: Male, Nationality: Pakistan												
Headache	Various nervous system diseases	Headache	Collection//Non-inoculation site (whole body)	2021-11-03	2021-11-03	2021-11-04	2	0	No	Grade 1	Untreated	Probably related
Subject number: 1639, group: recombinant vaccine group, age (years): 34, sex: male, nationality: Pakistan												
Nasal Congestion	Respiratory, thoracic and mediastinal diseases	Nasal congestion	Non-solicitation	2021-11-03	2021-11-03	2021-11-08	6	0	No	Grade 1	Self-medication	Probably related
Sore throat	Respiratory, thoracic and mediastinal disorders	Oropharyngeal pain	Unsolicited	2021-11-03	2021-11-03	November 4, 2021	2	0	No	Level 1	No treatment	Probably related
Fever: 38.0℃, Panadol tab Taken	Systemic diseases and various reactions at the administration site	Fever	Collection//Non-inoculation site (whole body)	2021-11-03	2021-11-03	2021-11-04	2	0	No	Grade 1	Self-medication	Probably related
Subject No.: 1641, Group: Recombinant Vaccine Group, Age (years): 41.1, Sex: Male, Nationality: Egypt												
Allergic Rhinitis	Respiratory, thoracic and mediastinal diseases	allergic coryza	Non-solicitation	2021-11-03	November 5, 2021	2021-11-07	3	2	No	Grade 1	Untreated	Possibly related
Sore throat	Respiratory, thoracic and mediastinal disorders	Oropharyngeal pain	Unsolicited	2021-11-03	2021-11-05	2021-11-07	3	2	No	Level 1	No treatment	May be relevant
Muscle pain (non-injection site)	Various musculoskeletal and connective tissue diseases	Myalgia	Collection//Non-inoculation site (whole body)	2021-11-03	2021-11-07	2021-11-26	20	4	No	Grade 3	Prescription drug	Possibly related
Ear pain	Ear and labyrinth disorders	Earache	Unsolicited	2021-11-03	2021-11-12	November 26, 2021	15	9	No	Level 2	Prescription Drug	Probably related

Adverse event description	System Organ Class	Preferred terms	Type of Adverse Event	Date of inoculation	Start Date	End date	Duration Days	Days from inoculation	Whether within 30min	Severity	Treatment situation	Correlation
Runny Nose	Respiratory, thoracic and mediastinal diseases	Runny nose	Non-solicitation	2021-11-03	November 12, 2021	2021-11-26	15	9	No	Grade 2	Prescription drug	Probably related
Subject No.: 1649, Group: Inactivated Vaccine Group, Age (years): 27.1, Sex: Male, Nationality: Afghanistan												
Headache	Various nervous system diseases	Headache	Collection//Non-inoculation site (whole body)	2021-11-03	2021-11-08	2021-11-10	3	5	No	Grade 1	Untreated	Possibly related
Subject No.: 1655, Group: Inactivated Vaccine Group, Age (years): 23.7, Sex: Male, Nationality: Bangladesh												
Pain	Systemic diseases and various reactions at the administration site	Vaccination site pain	Collection//inoculation site (local)	2021-11-03	2021-11-03			0	No	Grade 1	Untreated	Definitely related
Subject No.: 1663, Group: Recombinant Vaccine Group, Age (years): 44.8, Sex: Male, Nationality: Bangladesh												
Nasal congestion	Respiratory, thoracic and mediastinal diseases	Nasal congestion	Non-solicitation	2021-11-03	2021-11-03	2021-11-05	3	0	No	Grade 1	Self-medication	Probably related
Fever: 37.8 ℃	General disorders and administration site conditions	fever	Recruited//Non-Vaccination Site (Systemic)	2021-11-03	2021-11-03	November 5, 2021	3	0	No	Level 1	Self-medication	May be relevant
Headache	Various nervous system diseases	Headache	Collection//Non-inoculation site (whole body)	2021-11-03	2021-11-03	2021-11-05	3	0	No	Grade 1	Self-medication	Possibly related
Subject No.: 1665, Group: Recombinant Vaccine Group, Age (years): 28.9, Sex: Male, Nationality: Afghanistan												
Fever: 37.9℃	Systemic diseases and various reactions at the administration site	Fever	Collection//Non-inoculation site (whole body)	2021-11-03	November 4, 2021	2021-11-04	1	1	No	Grade 1	Untreated	Definitely related
Headache	Nervous system disorders	Headache	Recruited//Non-Vaccination Site (Systemic)	2021-11-03	2021-11-04	November 5, 2021	2	1	No	Level 1	No treatment	May be relevant
Subject No.: 1666, Group: Recombinant Vaccine Group, Age (years): 36.6, Sex: Male, Nationality: Afghanistan												
Fatigue/fatigue	General disorders and administration site conditions	Fatigue	Recruited//Non-Vaccination Site (Systemic)	2021-11-03	2021-11-03	November 5, 2021	3	0	No	Level 1	No treatment	Probably related

Adverse event description	System Organ Class	Preferred terms	Type of Adverse Event	Date of inoculation	Start Date	End date	Duration Days	Days from inoculation	Whether within 30min	Severity	Treatment situation	Correlation
Subject No.: 1668, Group: Inactivated Vaccine Group, Age (years): 34.7, Sex: Male, Nationality: Philippines												
Pruritus	General disorders and administration site conditions	Itching at vaccination site	Recruitment//injection site (local)	2021-11-03	2021-11-04	November 4, 2021	1	1	No	Level 1	No treatment	Definitely relevant
Subject No.: 1669, Group: Inactivated Vaccine Group, Age (years): 25.1, Sex: Male, Nationality: Pakistan												
Abdominal Pain	Gastrointestinal disorders	Abdominal pain	Unsolicited	2021-11-03	2021-11-03	2021-11-06	4	0	No	Level 1	No treatment	Probably related
Headache	Various nervous system diseases	Headache	Collection//Non-inoculation site (whole body)	2021-11-03	2021-11-03	2021-11-06	4	0	No	Grade 2	Self-medication	Probably related
Subject No.: 1679, Group: Inactivated Vaccine Group, Age (years): 34.8, Sex: Male, Nationality: India												
Fever: 38.0℃	Systemic diseases and various reactions at the administration site	Fever	Collection//Non-inoculation site (whole body)	2021-11-03	2021-11-03	2021-11-05	3	0	No	Grade 1	Self-medication	Probably related
Subject No.: 1681, Group: Inactivated Vaccine Group, Age (years): 33.4, Sex: Male, Nationality: Bangladesh												
Fever: 38.0℃	Systemic diseases and various reactions at the administration site	Fever	Collection//Non-inoculation site (whole body)	2021-11-03	2021-11-03	2021-11-05	3	0	No	Grade 1	Self-medication	Probably related
Fatigue/fatigue	General disorders and administration site conditions	Fatigue	Recruited//Non-Vaccination Site (Systemic)	2021-11-03	2021-11-03	November 5, 2021	3	0	No	Level 1	No treatment	Probably related
Subject No.: 1692, Group: Inactivated Vaccine Group, Age (years): 31.5, Sex: Male, Nationality: India												
pain	General disorders and administration site conditions	Pain at vaccination site	Recruitment//injection site (local)	November 4, 2021	2021-11-04	2021-11-06	3	0	No	Level 1	No treatment	Definitely relevant
Cough	Respiratory, thoracic and mediastinal diseases	Cough	Non-solicitation	2021-11-04	November 22, 2021	2021-11-24	3	18	No	Grade 1	Untreated	Probably related
Subject No.: 1693, Group: Recombinant Vaccine Group, Age (years): 34.6, Sex: Male, Nationality: India												

Adverse event description	System Organ Class	Preferred terms	Type of Adverse Event	Date of inoculation	Start Date	End date	Duration Days	Days from inoculation	Whether within 30min	Severity	Treatment situation	Correlation
Runny Nose	Respiratory, thoracic and mediastinal diseases	Runny nose	Non-solicitation	2021-11-04	2021-11-09	2021-11-13	5	5	No	Grade 2	Prescription drug	Possibly related
Fever: 38.5 ℃,-taking panadol 1000mg 3x daily for 2 days. Resolved-Temporal Artery	General disorders and administration site conditions	fever	Recruited//Non-Vaccination Site (Systemic)	November 4, 2021	2021-11-09	November 10, 2021	2	5	No	Level 2	Outpatient medication	Probably related
Cough	Respiratory, thoracic and mediastinal diseases	Cough	Collection//Non-inoculation site (whole body)	2021-11-04	2021-11-09	2021-11-12	4	5	No	Grade 2	Outpatient medication	Probably related
Headache	Nervous system disorders	Headache	Recruited//Non-Vaccination Site (Systemic)	November 4, 2021	2021-11-09	November 13, 2021	5	5	No	Level 2	Outpatient medication	Probably related
Fatigue/Asthenia	Systemic diseases and various reactions at the administration site	Fatigue	Collection//Non-inoculation site (whole body)	2021-11-04	2021-11-09	2021-11-13	5	5	No	Grade 2	Prescription drug	Possibly related
Subject No.: 1701, Group: Inactivated Vaccine Group, Age (years): 38.7, Sex: Male, Nationality: Pakistan												
Fever: 37.8℃	Systemic diseases and various reactions at the administration site	Fever	Collection//Non-inoculation site (whole body)	2021-11-04	November 4, 2021	2021-11-05	2	0	No	Grade 1	Self-medication	Possibly related
Subject No.: 1703, Group: Recombinant Vaccine Group, Age (years): 34.1, Sex: Male, Nationality: Egypt												
Muscle pain (non-injection site)	Various musculoskeletal and connective tissue diseases	Myalgia	Collection//Non-inoculation site (whole body)	2021-11-04	November 4, 2021	2021-11-11	8	0	No	Grade 2	Self-medication	Probably related
Feeling of hotness	General disorders and administration site conditions	Feeling of fever	Unsolicited	November 4, 2021	2021-11-05	2021-11-08	4	1	No	Level 1	Self-medication	May be relevant

Adverse event description	System Organ Class	Preferred terms	Type of Adverse Event	Date of inoculatio n	Start Date	End date	Dura tion Days	Days from inocula tion	Whethe r within 30min	Seve rity	Treatment situation	Correlatio n
Cough	Respiratory, thoracic and mediastinal diseases	Cough	Collection//Non-inocula tion site (whole body)	2021-11-0 4	November 5, 2021	2021-11-2 0	16	1	No	Grad e 1	Untreated	Probably related
Subject No.: 1713, Group: Inactivated Vaccine Group, Age (years): 39.7, Sex: Male, Nationality: Pakistan												
Muscle pain (non-injection site)	Various musculoskeletal and connective tissue diseases	Myalgia	Collection//Non-inocula tion site (whole body)	2021-11-0 4	November 4, 2021	2021-11-0 5	2	0	No	Grad e 1	Untreated	Possibly related
Myalgia	Musculoskeletal and connective tissue disorders	Myalgia	Unsolicited	November 4, 2021	2021-11-1 5	November 24, 2021	10	11	No	Leve l 2	Self-medication	Probably related
Subject No.: 1715, Group: Inactivated Vaccine Group, Age (years): 41.4, Sex: Male, Nationality: Pakistan												
Fatigue/fatigue	General disorders and administration site conditions	Fatigue	Recruited//Non-Vaccinat ion Site (Systemic)	November 4, 2021	2021-11-0 4	November 11, 2021	8	0	No	Leve l 2	Untreated	Probably related
Subject No.: 1721, Group: Inactivated Vaccine Group, Age (years): 49.2, Sex: Male, Nationality: Sri Lanka												
pain	General disorders and administration site conditions	Pain at vaccinatio n site	Recruitment//injection site (local)	November 4, 2021	November 4, 2021	November 5, 2021	2	0	No	Leve l 1	Untreated	Definitely relevant
Subject No.: 1727, Group: Recombinant vaccine group, Age (years): 40.5, Sex: Male, Nationality: Pakistan												
Coughing	Respiratory, thoracic and mediastinal disorders	coughing	Recruited//Non-Vaccinat ion Site (Systemic)	November 4, 2021	November 4, 2021	November 6, 2021	3	0	No	Grad e 1	Untreated	Probably related
Fatigue/Astheni a	Systemic diseases and various reactions at the administration site	Fatigue	Collection//Non-inocula tion site (whole body)	November 4, 2021	November 4, 2021	November 7, 2021	4	0	No	Grad e 2	Self-medication	Probably related
Subject No.: 1730, Group: Recombinant Vaccine Group, Age (years): 32.4, Sex: Male, Nationality: Pakistan												
Muscle pain (non-injection site)	Various musculoskeletal and connective tissue diseases	Myalgia	Collection//Non-inocula tion site (whole body)	November 4, 2021	November 4, 2021	November 6, 2021	3	0	No	Grad e 1	Untreated	Probably related

Adverse event description	System Organ Class	Preferred terms	Type of Adverse Event	Date of inoculation	Start Date	End date	Duration Days	Days from inoculation	Whether within 30min	Severity	Treatment situation	Correlation
Subject No.: 1747, Group: Recombinant Vaccine Group, Age (years): 40.8, Sex: Male, Nationality: Ghana												
Pain	Systemic diseases and various reactions at the administration site	Vaccination site pain	Collection//inoculation site (local)	November 4, 2021	November 4, 2021	November 6, 2021	3	0	No	Grade 1	Untreated	Definitely related
Subject No.: 1754, Group: Recombinant Vaccine Group, Age (years): 47.6, Sex: Male, Nationality: Bangladesh												
Headache	Various nervous system diseases	Headache	Collection//Non-inoculation site (whole body)	November 4, 2021	November 5, 2021	November 7, 2021	3	1	No	Grade 1	Untreated	Probably related
Subject No.: 1759, Group: Recombinant Vaccine Group, Age (years): 25.9, Sex: Male, Nationality: India												
Fatigue/Asthenia	Systemic diseases and various reactions at the administration site	Fatigue	Collection//Non-inoculation site (whole body)	November 4, 2021	November 4, 2021	November 6, 2021	3	0	No	Grade 1	Untreated	Probably related
Subject No.: 1762, Group: Inactivated Vaccine Group, Age (years): 28.3, Sex: Female, Nationality: Uganda												
Headache	Various nervous system diseases	Headache	Collection//Non-inoculation site (whole body)	November 4, 2021	November 4, 2021	November 8, 2021	5	0	No	Grade 2	Self-medication	Possibly related
Vertigo	Nervous system disorders	Dizziness	Recruited//Non-Vaccination Site (Systemic)	November 4, 2021	November 4, 2021	November 8, 2021	5	0	No	Level 2	Self-medication	May be relevant
Subject number: 1774, group: recombinant vaccine group, age (years): 33.2, gender: male, Nationality: Nepal												
Pain	General disorders and administration site conditions	Pain at vaccination site	Recruitment//injection site (local)	November 4, 2021	November 4, 2021	November 6, 2021	3	0	No	Grade 1	Untreated	Definitely relevant
Subject No.: 1778, Group: Inactivated Vaccine Group, Age (years): 36.7, Sex: Male, Nationality: Pakistan												
Pain	General disorders and administration site conditions	Pain at vaccination site	Recruitment//injection site (local)	November 4, 2021	November 4, 2021	November 6, 2021	3	0	No	Grade 1	Untreated	Definitely relevant
Subject No.: 1783, Group: Recombinant vaccine group, Age (years): 32, Sex: Male, Nationality: India												

Adverse event description	System Organ Class	Preferred terms	Type of Adverse Event	Date of inoculation	Start Date	End date	Duration Days	Days from inoculation	Whether within 30min	Severity	Treatment situation	Correlation
Fever: 38.2 ℃	General disorders and administration site conditions	fever	Recruited//Non-Vaccination Site (Systemic)	November 4, 2021	November 6, 2021	November 9, 2021	4	2	No	Grade 1	Self-medication	Probably related
Cough	Respiratory, thoracic and mediastinal diseases	Cough	Collection//Non-inoculation site (whole body)	November 4, 2021	November 6, 2021	November 9, 2021	4	2	No	Grade 2	Self-medication	Probably related
Subject No.: 1789, Group: Inactivated Vaccine Group, Age (years): 34.1, Sex: Male, Nationality: Pakistan												
Pain	Systemic diseases and various reactions at the administration site	Vaccination site pain	Collection//inoculation site (local)	November 4, 2021	November 4, 2021	November 5, 2021	2	0	No	Grade 1	Untreated	Definitely related
Subject No.: 1790, Group: Inactivated Vaccine Group, Age (years): 29.4, Sex: Male, Nationality: Ghana												
Headache	Various nervous system diseases	Headache	Non-solicitation	November 4, 2021	November 22, 2021	November 23, 2021	2	18	No	Grade 2	Self-medication	Probably related
Subject No.: 1802, Group: Recombinant Vaccine Group, Age (years): 41.8, Sex: Male, Nationality: Pakistan												
Headache	Various nervous system diseases	Headache	Collection//Non-inoculation site (whole body)	November 4, 2021	November 4, 2021	November 6, 2021	3	0	No	Grade 1	Untreated	Probably related
Subject No.: 1803, Group: Recombinant Vaccine Group, Age (years): 30.9, Sex: Male, Nationality: Bangladesh												
Pain	Systemic diseases and various reactions at the administration site	Vaccination site pain	Collection//inoculation site (local)	November 4, 2021	November 10, 2021	November 10, 2021	1	6	No	Grade 1	Untreated	Definitely related
Subject No.: 1805, Group: Inactivated Vaccine Group, Age (years): 38.1, Sex: Male, Nationality: Pakistan												
Pain	Systemic diseases and various reactions at the administration site	Vaccination site pain	Collection//inoculation site (local)	November 4, 2021	November 4, 2021	November 6, 2021	3	0	No	Grade 1	Untreated	Definitely related
Muscle pain (non-inoculation site)	Musculoskeletal and connective tissue disorders	Myalgia	Recruited//Non-Vaccination Site (Systemic)	November 4, 2021	November 4, 2021	November 6, 2021	3	0	No	Grade 1	Untreated	Probably related
Subject No.: 1806, Group: Recombinant vaccine group, Age (years): 37.8, Sex: Male, Nationality: Bangladesh												

Adverse event description	System Organ Class	Preferred terms	Type of Adverse Event	Date of inoculation	Start Date	End date	Duration Days	Days from inoculation	Whether within 30min	Severity	Treatment situation	Correlation
pain	General disorders and administration site conditions	Pain at vaccination site	Recruitment//injection site (local)	November 4, 2021	November 4, 2021	November 6, 2021	3	0	No	Grade 1	Self-medication	Definitely relevant
Subject No.: 1811, Group: Recombinant vaccine group, Age (years): 27.2, Sex: Male, Nationality: India												
pain	General disorders and administration site conditions	Pain at vaccination site	Recruitment//injection site (local)	November 4, 2021	November 4, 2021			0	No	Grade 1	Untreated	Definitely relevant
Subject No.: 2401, Group: Inactivated Vaccine Group, Age (years): 33.3, Sex: Male, Nationality: UAE												
Nasal Congestion	Respiratory, thoracic and mediastinal disorders	Nasal obstruction	Unsolicited	October 23, 2021	October 27, 2021	October 29, 2021	3	4	No	Grade 1	Untreated	May be relevant
Subject number: 2402, group: recombinant vaccine group, age (years): 29.8, gender: female, nationality: UAE												
pain	General disorders and administration site conditions	Pain at vaccination site	Recruitment//injection site (local)	October 23, 2021	October 23, 2021	October 24, 2021	2	0	No	Grade 1	Untreated	Definitely relevant
Headache	Various nervous system diseases	Headache	Collection//Non-inoculation site (whole body)	October 23, 2021	October 24, 2021	October 26, 2021	3	1	No	Grade 1	Untreated	Possibly related
Subject No.: 2410, Group: Recombinant Vaccine Group, Age (years): 35.6, Sex: Male, Nationality: UAE												
Headache	Various nervous system diseases	Headache	Collection//Non-inoculation site (whole body)	October 24, 2021	October 24, 2021	October 24, 2021	1	0	No	Grade 1	Untreated	Possibly related
Subject number: 2426, Group: Recombinant vaccine group, Age (years): 31, Sex: Male, Nationality: Pakistan												

Adverse event description	System Organ Class	Preferred terms	Type of Adverse Event	Date of inoculation	Start Date	End date	Duration Days	Days from inoculation	Whether within 30min	Severity	Treatment situation	Correlation
Fever: 38.5 ℃ .went to clinic for check up prescribed medication was given don't remember the name of medication	Systemic diseases and various reactions at the administration site	Fever	Collection//Non-inoculation site (whole body)	October 24, 2021	October 27, 2021	October 30, 2021	4	3	No	Grade 2	Prescription drug	Probably related
Headache	Nervous system disorders	Headache	Recruited//Non-Vaccination Site (Systemic)	October 24, 2021	October 18, 2021	October 28, 2021	1	4	No	Grade 1	No medication in outpatient clinic	May be relevant
body pain	Systemic diseases and various reactions at the administration site	Pain	Non-solicitation	October 24, 2021	October 29, 2021	October 31, 2021	3	5	No	Grade 1	No medication in outpatient department	Possibly related
Subject number: 2427, group: recombinant vaccine group, age (years): 39, sex: female, nationality: Morocco												
Dysuria	Diseases of kidney and urinary system	Dysuria	Non-solicitation	October 24, 2021	October 24, 2021	October 31, 2021	8	0	No	Grade 1	Untreated	Possibly related
Polyuria	Renal and urinary disorders	Polyuria	Unsolicited	October 24, 2021	October 24, 2021	October 29, 2021	6	0	No	Grade 1	Untreated	May be relevant
Fever: 37.8℃	Systemic diseases and various reactions at the administration site	Fever	Collection//Non-inoculation site (whole body)	October 24, 2021	October 24, 2021	October 25, 2021	2	0	No	Grade 1	Untreated	Possibly related
Fatigue/fatigue	General disorders and administration site conditions	Fatigue	Recruited//Non-Vaccination Site (Systemic)	October 24, 2021	October 24, 2021	October 26, 2021	3	0	No	Grade 1	Untreated	May be relevant
Pain	Systemic diseases and various reactions at the administration site	Vaccination site pain	Collection//inoculation site (local)	October 24, 2021	October 24, 2021	October 29, 2021	6	0	No	Grade 1	Untreated	Possibly related

Adverse event description	System Organ Class	Preferred terms	Type of Adverse Event	Date of inoculation	Start Date	End date	Duration Days	Days from inoculation	Whether within 30min	Severity	Treatment situation	Correlation
Pruritus	General disorders and administration site conditions	Itching at vaccination site	Recruitment//injection site (local)	October 24, 2021	October 24, 2021	October 24, 2021	1	0	No	Grade 1	Untreated	May be relevant
Diarrhoea	Gastrointestinal system diseases	Diarrhoea	Collection//Non-inoculation site (whole body)	October 24, 2021	October 24, 2021	October 26, 2021	3	0	No	Grade 1	Untreated	Possibly related
Subject No.: 2431, Group: Recombinant Vaccine Group, Age (years): 50.4, Sex: Male, Nationality: India												
Muscle pain (non-injection site)	Various musculoskeletal and connective tissue diseases	Myalgia	Collection//Non-inoculation site (whole body)	October 25, 2021	October 25, 2021	October 27, 2021	3	0	No	Grade 1	Untreated	Probably related
Subject No.: 2434, Group: Recombinant Vaccine Group, Age (years): 35.3, Sex: Male, Nationality: Bangladesh												
Eye Itchiness	Ocular organ disease	Eye pruritus	Non-solicitation	October 25, 2021	October 25, 2021	November 18, 2021	25	0	No	Grade 2	Untreated	Probably related
Pruritus	Skin and subcutaneous tissue disorders	Pruritus	Recruitment//injection site (local)	October 25, 2021	October 27, 2021	October 29, 2021	3	2	No	Grade 1	Untreated	May be relevant
Subject number: 2436, group: recombinant vaccine group, age (years): 42.5, gender: female, nationality: Egypt												
Pain	General disorders and administration site conditions	Pain at vaccination site	Recruitment//injection site (local)	October 25, 2021	October 25, 2021	October 28, 2021	4	0	No	Grade 1	Untreated	Definitely relevant
Subject No.: 2437, Group: Inactivated Vaccine Group, Age (years): 49.5, Sex: Male, Nationality: India												
Pain	General disorders and administration site conditions	Pain at vaccination site	Recruitment//injection site (local)	October 25, 2021	October 25, 2021	October 25, 2021	1	0	No	Grade 1	Untreated	Definitely relevant
Subject No.: 2442, Group: Inactivated Vaccine Group, Age (years): 42.4, Sex: Male, Nationality: Bangladesh												
Fatigue/fatigue	General disorders and administration site conditions	Fatigue	Recruited//Non-Vaccination Site (Systemic)	October 25, 2021	October 25, 2021	November 1, 2021	8	0	No	Grade 1	Untreated	May be relevant

Adverse event description	System Organ Class	Preferred terms	Type of Adverse Event	Date of inoculation	Start Date	End date	Duration Days	Days from inoculation	Whether within 30min	Severity	Treatment situation	Correlation
Pruritus	Systemic diseases and various reactions at the administration site	Vaccination site pruritus	Collection//inoculation site (local)	October 25, 2021	October 25, 2021	November 1, 2021	8	0	No	Grade 1	Untreated	Possibly related
Runny nose	Respiratory, thoracic and mediastinal disorders	Runny nose	Unsolicited	October 25, 2021	October 26, 2021	October 27, 2021	2	1	No	Grade 1	Untreated	May be relevant
Subject number: 2444, group: recombinant vaccine group, age (years): 33.8, gender: male, nationality: India												
Headache	Nervous system disorders	Headache	Recruited//Non-Vaccination Site (Systemic)	October 25, 2021	October 29, 2021	October 31, 2021	3	4	No	Level 2	Self-medication	May be relevant
Subject number: 2451, group: recombinant vaccine group, age (years): 43.9, gender: male, nationality: Egypt												
Headache	Nervous system disorders	Headache	Recruited//Non-Vaccination Site (Systemic)	October 26, 2021	October 26, 2021	October 27, 2021	2	0	No	Grade 1	Untreated	Probably related
Subject number: 2454, group: recombinant vaccine group, age (years): 32.4, gender: female, nationality: UAE												
Headache	Nervous system disorders	Headache	Recruited//Non-Vaccination Site (Systemic)	October 27, 2021	October 27, 2021	October 30, 2021	4	0	No	Level 2	Self-medication	May be relevant
Subject No.: 2460, Group: Inactivated Vaccine Group, Age (years): 33, Sex: Male, Nationality: Bangladesh												
pain	General disorders and administration site conditions	Pain at vaccination site	Recruitment//injection site (local)	October 31, 2021	October 31, 2021	November 1, 2021	2	0	No	Grade 1	Self-medication	Definitely relevant
Subject number: 2466, group: recombinant vaccine group, age (years): 58.5, gender: male, nationality: India												
pain	General disorders and administration site conditions	Pain at vaccination site	Recruitment//injection site (local)	November 1, 2021	November 2, 2021	November 3, 2021	2	1	No	Grade 1	Untreated	Definitely relevant
Subject No.: 2471, Group: Inactivated Vaccine Group, Age (years): 30.3, Sex: Male, Nationality: Bangladesh												
pain	General disorders and administration site conditions	Pain at vaccination site	Recruitment//injection site (local)	November 1, 2021	November 6, 2021	November 8, 2021	3	5	No	Level 2	Self-medication	Definitely relevant

Adverse event description	System Organ Class	Preferred terms	Type of Adverse Event	Date of inoculation	Start Date	End date	Duration Days	Days from inoculation	Whether within 30min	Severity	Treatment situation	Correlation
Subject number: 2476, group: recombinant vaccine group, age (years): 59, gender: male, nationality: India												
Headache	Nervous system disorders	Headache	Recruited//Non-Vaccination Site (Systemic)	November 1, 2021	November 3, 2021			2	No	Grade 1	Untreated	Probably related
Headache	Various nervous system diseases	Headache	Collection//Non-inoculation site (whole body)	November 1, 2021	November 6, 2021	November 8, 2021	3	5	No	Grade 2	Self-medication	Possibly related
Subject No.: 2489, Group: Inactivated Vaccine Group, Age (years): 36.1, Sex: Male, Nationality: UAE												
Pain	Systemic diseases and various reactions at the administration site	Vaccination site pain	Collection//inoculation site (local)	November 2, 2021	November 2, 2021	November 3, 2021	2	0	No	Grade 1	Untreated	Definitely related
Headache	Nervous system disorders	Headache	Recruited//Non-Vaccination Site (Systemic)	November 2, 2021	November 3, 2021	November 4, 2021	2	1	No	Level 2	Self-medication	Probably related
Dyspnea	Respiratory, thoracic and mediastinal diseases	Difficulty breathing	Non-solicitation	November 2, 2021	November 22, 2021	November 27, 2021	6	20	No	Grade 2	Prescription drug	Possibly related
Fatigue, Asthenia	General disorders and administration site conditions	Fatigue	Unsolicited	November 2, 2021	2021-11-22	November 27, 2021	6	20	No	Level 2	Prescription Drug	May be relevant
Headache	Various nervous system diseases	Headache	Non-solicitation	November 2, 2021	November 22, 2021	November 27, 2021	6	20	No	Grade 2	Prescription drug	Possibly related
Myalgia	Musculoskeletal and connective tissue disorders	Myalgia	Unsolicited	November 2, 2021	November 22, 2021	November 27, 2021	6	20	No	Level 2	Prescription Drug	May be relevant
nasal congestion	Respiratory, thoracic and mediastinal diseases	Nasal congestion	Non-solicitation	November 2, 2021	November 22, 2021	November 27, 2021	6	20	No	Grade 2	Prescription drug	Possibly related
Subject No.: 2498, Group: Inactivated Vaccine Group, Age (years): 26.8, Sex: Male, Nationality: Sudan												
Muscle pain (non-injection site)	Various musculoskeletal and connective tissue diseases	Myalgia	Collection//Non-inoculation site (whole body)	November 2, 2021	November 3, 2021	November 25, 2021	23	1	No	Grade 1	Untreated	Probably related

Adverse event description	System Organ Class	Preferred terms	Type of Adverse Event	Date of inoculation	Start Date	End date	Duration Days	Days from inoculation	Whether within 30min	Severity	Treatment situation	Correlation
Subject No.: 2507, Group: Recombinant Vaccine Group, Age (years): 38.2, Sex: Male, Nationality: Nigeria												
Pain	Systemic diseases and various reactions at the administration site	Vaccination site pain	Collection//inoculation site (local)	November 2, 2021	November 3, 2021	November 3, 2021	1	1	No	Grade 1	Untreated	Definitely related
Subject No.: 2509, Group: Inactivated Vaccine Group, Age (years): 35.2, Sex: Male, Nationality: Nepal												
Muscle pain (non-injection site)	Various musculoskeletal and connective tissue diseases	Myalgia	Collection//Non-inoculation site (whole body)	November 2, 2021	November 3, 2021	November 3, 2021	1	1	No	Grade 2	Self-medication	Probably related
Subject No.: 2524, Group: Inactivated Vaccine Group, Age (years): 37.8, Sex: Male, Nationality: Bangladesh												
increased heart rate	Various inspections	Heart rate increased	Non-solicitation	November 3, 2021	November 3, 2021	November 4, 2021	2	0	No	Grade 1	Self-medication	Probably related
Subject No.: 2549, Group: Inactivated Vaccine Group, Age (years): 33.8, Sex: Male, Nationality: Bangladesh												
Pain	Systemic diseases and various reactions at the administration site	Vaccination site pain	Collection//inoculation site (local)	November 3, 2021	November 3, 2021	2021-11-24	22	0	No	Grade 2	Self-medication	Definitely related
Subject No.: 2553, Group: Inactivated Vaccine Group, Age (years): 41.6, Sex: Male, Nationality: Sri Lanka												
Fatigue/Asthenia	Systemic diseases and various reactions at the administration site	Fatigue	Collection//Non-inoculation site (whole body)	November 3, 2021	November 3, 2021	November 5, 2021	3	0	No	Grade 1	Untreated	Probably related
Subject No.: 2563, Group: Recombinant Vaccine Group, Age (years): 37.8, Sex: Male, Nationality: Pakistan												
Headache	Various nervous system diseases	Headache	Collection//Non-inoculation site (whole body)	November 3, 2021	November 3, 2021	November 4, 2021	2	0	No	Grade 2	Self-medication	Probably related
Fatigue/fatigue	General disorders and administration site conditions	Fatigue	Recruited//Non-Vaccination Site (Systemic)	November 3, 2021	November 3, 2021	November 5, 2021	3	0	No	Grade 1	Untreated	Probably related
Subject number: 2587, group: recombinant vaccine group, age (years): 58.3, gender: male, nationality: Bangladesh												

Adverse event description	System Organ Class	Preferred terms	Type of Adverse Event	Date of inoculation	Start Date	End date	Duration Days	Days from inoculation	Whether within 30min	Severity	Treatment situation	Correlation
Fatigue/fatigue	General disorders and administration site conditions	Fatigue	Recruited//Non-Vaccination Site (Systemic)	November 4, 2021	November 7, 2021	November 9, 2021	3	3	No	Grade 1	Untreated	Probably related
Subject number: 2594, group: recombinant vaccine group, age (years): 36.5, gender: male, nationality: Uganda												
Headache	Nervous system disorders	Headache	Recruited//Non-Vaccination Site (Systemic)	November 4, 2021	November 4, 2021	November 5, 2021	2	0	No	Grade 1	Untreated	Probably related
Subject No.: 2601, Group: Recombinant vaccine group, Age (years): 45.8, Sex: Male, Nationality: Ghana												
Headache	Nervous system disorders	Headache	Recruited//Non-Vaccination Site (Systemic)	November 4, 2021	November 4, 2021	November 8, 2021	5	0	No	Grade 1	Untreated	Probably related
Muscle pain (non-injection site)	Various musculoskeletal and connective tissue diseases	Myalgia	Collection//Non-inoculation site (whole body)	November 4, 2021	November 4, 2021	November 8, 2021	5	0	No	Grade 1	Untreated	Probably related
Subject No.: 2604, Group: Inactivated Vaccine Group, Age (years): 45.6, Sex: Male, Nationality: India												
Diarrhoea	Gastrointestinal system diseases	Diarrhoea	Collection//Non-inoculation site (whole body)	November 4, 2021	November 5, 2021	November 7, 2021	3	1	No	Grade 1	Untreated	Probably related
Subject number: 2605, group: recombinant vaccine group, age (years): 26.8, sex: male, nationality: Pakistan												
Muscle pain (non-injection site)	Various musculoskeletal and connective tissue diseases	Myalgia	Collection//Non-inoculation site (whole body)	November 4, 2021	November 4, 2021	November 15, 2021	12	0	No	Grade 2	Self-medication	Probably related
Subject No.: 2609, Group: Inactivated Vaccine Group, Age (years): 43.6, Sex: Male, Nationality: Uganda												
Pain	Systemic diseases and various reactions at the administration site	Vaccination site pain	Collection//inoculation site (local)	November 4, 2021	November 4, 2021	November 5, 2021	2	0	No	Grade 1	Untreated	Definitely related
Fatigue, Asthenia	General disorders and administration site conditions	Fatigue	Unsolicited	November 4, 2021	November 16, 2021	November 22, 2021	7	12	No	Level 2	Untreated	Probably related
Subject No.: 2618, Group: Inactivated Vaccine Group, Age (years): 45.9, Sex: Male, Nationality: Sri Lanka												

Adverse event description	System Organ Class	Preferred terms	Type of Adverse Event	Date of inoculation	Start Date	End date	Duration Days	Days from inoculation	Whether within 30min	Severity	Treatment situation	Correlation
Pain	General disorders and administration site conditions	Pain at vaccination site	Recruitment//injection site (local)	November 4, 2021	November 4, 2021	November 6, 2021	3	0	No	Grade 1	Untreated	Definitely relevant
Fever: 38.0℃, Tympanic thermometer	Systemic diseases and various reactions at the administration site	Fever	Non-solicitation	November 4, 2021	December 2, 2021	December 3, 2021	2	28	No	Grade 1	Untreated	Possibly related
Subject No.: 2625, Group: Recombinant Vaccine Group, Age (years): 46.2, Sex: Male, Nationality: Bangladesh												
Fever: 40.0℃	Systemic diseases and various reactions at the administration site	Fever	Collection//Non-inoculation site (whole body)	November 4, 2021	November 4, 2021	November 10, 2021	7	0	No	Grade 3	Self-medication	Probably related
Muscle pain (non-inoculation site)	Musculoskeletal and connective tissue disorders	Myalgia	Recruited//Non-Vaccination Site (Systemic)	November 4, 2021	November 4, 2021	November 7, 2021	4	0	No	Grade 1	Untreated	Probably related
Fatigue/Asthenia	Systemic diseases and various reactions at the administration site	Fatigue	Collection//Non-inoculation site (whole body)	November 4, 2021	November 5, 2021	November 5, 2021	1	1	No	Grade 1	Untreated	Probably related
Subject No.: 2629, Group: Recombinant Vaccine Group, Age (years): 45.8, Sex: Male, Nationality: Uganda												
Fatigue/Asthenia	Systemic diseases and various reactions at the administration site	Fatigue	Collection//Non-inoculation site (whole body)	November 4, 2021	November 4, 2021	November 6, 2021	3	0	No	Grade 1	Untreated	Probably related
Subject No.: 2641, Group: Inactivated Vaccine Group, Age (years): 31.7, Sex: Male, Nationality: Pakistan												
Muscle pain (non-injection site)	Various musculoskeletal and connective tissue diseases	Myalgia	Collection//Non-inoculation site (whole body)	November 4, 2021	November 5, 2021	November 8, 2021	4	1	No	Grade 1	Untreated	Probably related
Subject No.: 2644, Group: Recombinant Vaccine Group, Age (years): 31.8, Sex: Male, Nationality: Pakistan												
Pain	Systemic diseases and various reactions at the administration site	Vaccination site pain	Collection//inoculation site (local)	November 4, 2021	November 4, 2021	November 5, 2021	2	0	No	Grade 1	Untreated	Definitely related

Adverse event description	System Organ Class	Preferred terms	Type of Adverse Event	Date of inoculation	Start Date	End date	Duration Days	Days from inoculation	Whether within 30min	Severity	Treatment situation	Correlation
Subject No.: 2649, Group: Inactivated Vaccine Group, Age (years): 33.3, Sex: Male, Nationality: Pakistan												
Pain	Systemic diseases and various reactions at the administration site	Vaccination site pain	Collection//inoculation site (local)	November 4, 2021	November 4, 2021	November 5, 2021	2	0	No	Grade 1	Untreated	Definitely related
Subject No.: 2662, Group: Recombinant Vaccine Group, Age (years): 42.2, Sex: Male, Nationality: Ethiopia												
Headache	Various nervous system diseases	Headache	Collection//Non-inoculation site (whole body)	November 4, 2021	November 4, 2021	November 4, 2021	1	0	No	Grade 1	Untreated	Probably related
Subject No.: 2665, Group: Recombinant Vaccine Group, Age (years): 43.8, Sex: Male, Nationality: Afghanistan												
Muscle pain (non-injection site)	Various musculoskeletal and connective tissue diseases	Myalgia	Collection//Non-inoculation site (whole body)	November 4, 2021	November 6, 2021	November 6, 2021	1	2	No	Grade 1	Untreated	Probably related
Subject No.: 2666, Group: Inactivated Vaccine Group, Age (years): 28.5, Sex: Male, Nationality: Pakistan												
Headache	Various nervous system diseases	Headache	Collection//Non-inoculation site (whole body)	November 4, 2021	November 4, 2021	November 4, 2021	1	0	No	Grade 1	Untreated	Probably related
Subject No.: 2668, Group: Recombinant Vaccine Group, Age (years): 34.2, Sex: Male, Nationality: Uganda												
Fatigue/Asthenia	Systemic diseases and various reactions at the administration site	Fatigue	Collection//Non-inoculation site (whole body)	November 4, 2021	November 5, 2021	November 5, 2021	1	1	No	Grade 1	Untreated	Possibly related
Subject No.: 2670, Group: Inactivated Vaccine Group, Age (years): 53.5, Sex: Male, Nationality: Sri Lanka												
Headache	Various nervous system diseases	Headache	Collection//Non-inoculation site (whole body)	November 4, 2021	November 4, 2021	November 4, 2021	1	0	No	Grade 2	Self-medication	Probably related
Muscle pain (non-inoculation site)	Musculoskeletal and connective tissue disorders	Myalgia	Recruited//Non-Vaccination Site (Systemic)	November 4, 2021	November 5, 2021	November 11, 2021	7	1	No	Level 2	Untreated	Probably related
Subject No.: 2672, Group: Recombinant vaccine group, Age (years): 40.4, Sex: Male, Nationality: Pakistan												

Adverse event description	System Organ Class	Preferred terms	Type of Adverse Event	Date of inoculation	Start Date	End date	Duration Days	Days from inoculation	Whether within 30min	Severity	Treatment situation	Correlation
Pain	General disorders and administration site conditions	Pain at vaccination site	Recruitment//injection site (local)	November 4, 2021	November 4, 2021	November 6, 2021	3	0	No	Level 2	Self-medication	Definitely relevant
Subject No.: 2678, Group: Inactivated Vaccine Group, Age (years): 43.8, Sex: Male, Nationality: Pakistan												
Muscle pain (non-inoculation site)	Musculoskeletal and connective tissue disorders	Myalgia	Recruited//Non-Vaccination Site (Systemic)	November 4, 2021	November 5, 2021	November 7, 2021	3	1	No	Grade 1	Untreated	Probably related
Headache	Various nervous system diseases	Headache	Collection//Non-inoculation site (whole body)	November 4, 2021	November 6, 2021	November 11, 2021	6	2	No	Grade 2	Untreated	Probably related
Subject No.: 2681, Group: Recombinant Vaccine Group, Age (years): 36.3, Sex: Male, Nationality: Ghana												
Muscle pain (non-injection site)	Various musculoskeletal and connective tissue diseases	Myalgia	Collection//Non-inoculation site (whole body)	November 4, 2021	November 4, 2021	November 7, 2021	4	0	No	Grade 1	Untreated	Probably related
Subject No.: 2705, Group: Recombinant Vaccine Group, Age (years): 33.5, Sex: Male, Nationality: Pakistan												
Muscle pain (non-injection site)	Various musculoskeletal and connective tissue diseases	Myalgia	Collection//Non-inoculation site (whole body)	November 5, 2021	November 5, 2021	November 6, 2021	2	0	No	Grade 2	Self-medication	Probably related
Subject No.: 2710, Group: Recombinant Vaccine Group, Age (years): 25.8, Sex: Male, Nationality: Pakistan												
Cold Symptoms	Infection and infectious diseases	Nasopharyngitis	Non-solicitation	November 5, 2021	November 6, 2021	November 9, 2021	4	1	No	Grade 1	Untreated	Possibly related
coughing	Respiratory, thoracic and mediastinal disorders	coughing	Recruited//Non-Vaccination Site (Systemic)	November 5, 2021	November 6, 2021	November 9, 2021	4	1	No	Grade 1	Untreated	May be relevant
Subject Number: 2721, Group: Inactivated Vaccine Group, Age (years): 31.8, Sex: Male, Nationality: Uganda												
Abdominal pain	Gastrointestinal disorders	Abdominal pain	Unsolicited	November 5, 2021	November 8, 2021	November 14, 2021	7	3	No	Level 2	Untreated	Probably related

Adverse event description	System Organ Class	Preferred terms	Type of Adverse Event	Date of inoculation	Start Date	End date	Duration Days	Days from inoculation	Whether within 30min	Severity	Treatment situation	Correlation
Pruritus	Skin and subcutaneous tissue diseases	Pruritus	Collection//inoculation site (local)	November 5, 2021	November 8, 2021	December 21, 2021	44	3	No	Grade 2	No medication in outpatient department	Probably related
Subject number: 2726, group: recombinant vaccine group, age (years): 26.4, sex: male, nationality: Uganda												
Fever: 37.8℃	Systemic diseases and various reactions at the administration site	Fever	Collection//Non-inoculation site (whole body)	November 5, 2021	November 7, 2021	November 8, 2021	2	2	No	Grade 1	Prescription drug	Possibly related
Headache	Nervous system disorders	Headache	Recruited//Non-Vaccination Site (Systemic)	November 5, 2021	November 7, 2021	November 8, 2021	2	2	No	Level 2	Prescription Drug	May be relevant
Fatigue/Asthenia	Systemic diseases and various reactions at the administration site	Fatigue	Collection//Non-inoculation site (whole body)	November 5, 2021	November 9, 2021	2021-11-12	4	4	No	Grade 1	Untreated	Probably related
Diarrhea	Gastrointestinal disorders	Diarrhea	Recruited//Non-Vaccination Site (Systemic)	November 5, 2021	November 10, 2021	November 10, 2021	1	5	No	Grade 1	Untreated	Probably related
Subject Number: 2728, Group: Inactivated Vaccine Group, Age (years): 37.3, Sex: Male, Nationality: Uganda												
Headache	Nervous system disorders	Headache	Recruited//Non-Vaccination Site (Systemic)	November 5, 2021	November 5, 2021	November 6, 2021	2	0	No	Level 2	Self-medication	Probably related
Subject Number: 2735, Group: Inactivated Vaccine Group, Age (Y): 31.1, Sex: Male, Nationality: India												
coughing	Respiratory, thoracic and mediastinal disorders	coughing	Recruited//Non-Vaccination Site (Systemic)	November 5, 2021	November 7, 2021	November 11, 2021	5	2	No	Grade 1	Untreated	Probably related
Headache	Various nervous system diseases	Headache	Collection//Non-inoculation site (whole body)	November 5, 2021	November 7, 2021	November 9, 2021	3	2	No	Grade 1	Untreated	Probably related
Subject number: 2737, group: recombinant vaccine group, age (years): 38.2, sex: male, nationality: Sri Lanka												
Insomnia	Psychiatric category	Insomnia	Non-solicitation	November 5, 2021	November 5, 2021	November 15, 2021	11	0	No	Grade 1	Untreated	Probably related
Subject No.: 2772, Group: Recombinant Vaccine Group, Age (years): 29.8, Sex: Male, Nationality: Pakistan												

Adverse event description	System Organ Class	Preferred terms	Type of Adverse Event	Date of inoculation	Start Date	End date	Duration Days	Days from inoculation	Whether within 30min	Severity	Treatment situation	Correlation
Pain	Systemic diseases and various reactions at the administration site	Vaccination site pain	Collection//inoculation site (local)	November 5, 2021	November 5, 2021	November 6, 2021	2	0	No	Grade 2	Self-medication	Definitely related
Subject No.: 2788, Group: Inactivated Vaccine Group, Age (years): 59.7, Sex: Male, Nationality: Bangladesh												
Fever: 37.8℃, tympani	Systemic diseases and various reactions at the administration site	Fever	Collection//Non-inoculation site (whole body)	November 6, 2021	November 7, 2021	November 8, 2021	2	1	No	Grade 1	Self-medication	Possibly related
Subject No.: 2790, Group: Inactivated Vaccine Group, Age (years): 47.7, Sex: Male, Nationality: Bangladesh												
Pain	Systemic diseases and various reactions at the administration site	Vaccination site pain	Collection//inoculation site (local)	November 6, 2021	November 6, 2021	November 7, 2021	2	0	No	Grade 1	No medication in outpatient department	Possibly related
Vertigo	Nervous system disorders	Dizziness	Recruited//Non-Vaccination Site (Systemic)	November 6, 2021	November 6, 2021	November 8, 2021	3	0	No	Grade 1	No medication in outpatient clinic	May be relevant
Fatigue/Asthenia	Systemic diseases and various reactions at the administration site	Fatigue	Collection//Non-inoculation site (whole body)	November 6, 2021	November 7, 2021	November 14, 2021	8	1	No	Grade 1	No medication in outpatient department	Possibly related
Subject No.: 2804, Group: Inactivated Vaccine Group, Age (years): 33.3, Sex: Male, Nationality: Bangladesh												
Arthralgia	Various musculoskeletal and connective tissue diseases	Arthralgia	Collection//Non-inoculation site (whole body)	November 6, 2021	November 6, 2021	November 12, 2021	7	0	No	Grade 1	Untreated	Possibly related
Fatigue/fatigue	General disorders and administration site conditions	Fatigue	Recruited//Non-Vaccination Site (Systemic)	November 6, 2021	November 6, 2021	November 12, 2021	7	0	No	Grade 1	Untreated	May be relevant
Muscle pain (non-injection site)	Various musculoskeletal and connective tissue diseases	Myalgia	Collection//Non-inoculation site (whole body)	November 6, 2021	November 6, 2021	November 12, 2021	7	0	No	Grade 1	Untreated	Probably related
Weakness on both hands	Nervous system disorders	Myasthenia	Unsolicited	November 6, 2021	November 8, 2021	November 12, 2021	5	2	No	Grade 1	Untreated	May be relevant

Adverse event description	System Organ Class	Preferred terms	Type of Adverse Event	Date of inoculation	Start Date	End date	Duration Days	Days from inoculation	Whether within 30min	Severity	Treatment situation	Correlation
Subject No.: 2805, Group: Recombinant vaccine group, Age (years): 41.8, Sex: Male, Nationality: India												
Sore throat	Respiratory, thoracic and mediastinal disorders	Oropharyngeal pain	Unsolicited	November 6, 2021	November 7, 2021	November 18, 2021	12	1	No	Grade 3	Hospitalization	Probably related
Fever: 39.1℃, tympanic	Systemic diseases and various reactions at the administration site	Fever	Collection//Non-inoculation site (whole body)	November 6, 2021	November 7, 2021	November 15, 2021	9	1	No	Grade 3	hospitalization	Probably related
Subject No.: 2807, Group: Inactivated Vaccine Group, Age (years): 40.3, Sex: Male, Nationality: Syria												
Cough	Respiratory, thoracic and mediastinal diseases	Cough	Collection//Non-inoculation site (whole body)	November 6, 2021	November 10, 2021	November 14, 2021	5	4	No	Grade 1	Untreated	Probably related
Subject No.: 2816, Group: Recombinant Vaccine Group, Age (years): 48.2, Sex: Male, Nationality: Bangladesh												
Muscle pain (non-injection site)	Various musculoskeletal and connective tissue diseases	Myalgia	Collection//Non-inoculation site (whole body)	November 6, 2021	November 6, 2021	November 6, 2021	1	0	No	Grade 1	Untreated	Possibly related
Subject No.: 2819, Group: Recombinant Vaccine Group, Age (years): 50.5, Sex: Male, Nationality: India												
Pain	Systemic diseases and various reactions at the administration site	Vaccination site pain	Collection//inoculation site (local)	November 6, 2021	November 6, 2021	November 8, 2021	3	0	No	Grade 1	Untreated	Definitely related
Subject No.: 2824, Group: Inactivated Vaccine Group, Age (years): 30.7, Sex: Male, Nationality: Pakistan												
Muscle pain (non-injection site)	Various musculoskeletal and connective tissue diseases	Myalgia	Collection//Non-inoculation site (whole body)	November 6, 2021	November 9, 2021	November 10, 2021	2	3	No	Grade 1	Untreated	Probably related
Subject No.: 2831, Group: Recombinant Vaccine Group, Age (years): 48.5, Sex: Female, Nationality: Philippines												
Flushing	Systemic diseases and various reactions at the administration site	Vaccination site erythema	Collection//inoculation site (local)	November 6, 2021	November 10, 2021	November 14, 2021	5	4	No	Grade 1	Untreated	Definitely related
Subject No.: 2835, Group: Inactivated Vaccine Group, Age (years): 42.9, Sex: Male, Nationality: Bangladesh												

Adverse event description	System Organ Class	Preferred terms	Type of Adverse Event	Date of inoculation	Start Date	End date	Duration Days	Days from inoculation	Whether within 30min	Severity	Treatment situation	Correlation
Chest pain	Systemic diseases and various reactions at the administration site	Chest pain	Non-solicitation	November 6, 2021	November 8, 2021	November 10, 2021	3	2	No	Grade 1	No medication in outpatient department	Possibly related
Muscle pain (non-inoculation site)	Musculoskeletal and connective tissue disorders	Myalgia	Recruited//Non-Vaccination Site (Systemic)	November 6, 2021	November 8, 2021	November 22, 2021	15	2	No	Grade 1	Untreated	Probably related
Subject number: 2842, group: recombinant vaccine group, age (years): 27, gender: male, nationality: Nepal												
Headache	Nervous system disorders	Headache	Recruited//Non-Vaccination Site (Systemic)	November 6, 2021	November 7, 2021	November 8, 2021	2	1	No	Grade 1	Untreated	May be relevant
Subject No.: 2848, Group: Inactivated Vaccine Group, Age (years): 46, Sex: Male, Nationality: Philippines												
Fatigue, Asthenia	General disorders and administration site conditions	Fatigue	Unsolicited	November 6, 2021	November 24, 2021			18	No	Level 2	Prescription Drug	Probably related
Subject No.: 2849, Group: Inactivated Vaccine Group, Age (years): 35.8, Sex: Male, Nationality: Bangladesh												
Fatigue/fatigue	General disorders and administration site conditions	Fatigue	Recruited//Non-Vaccination Site (Systemic)	November 6, 2021	November 8, 2021	November 10, 2021	3	2	No	Grade 1	Untreated	Probably related
Subject No.: 2852, Group: Inactivated Vaccine Group, Age (Y): 50.9, Sex: Female, Nationality: Bangladesh												
Runny nose	Respiratory, thoracic and mediastinal disorders	Runny nose	Unsolicited	November 6, 2021	November 7, 2021	November 11, 2021	5	1	No	Level 2	Self-medication	Probably related
Sore Throat	Respiratory, thoracic and mediastinal diseases	Oropharyngeal pain	Non-solicitation	November 6, 2021	November 7, 2021	November 11, 2021	5	1	No	Grade 2	Self-medication	Probably related
Muscle pain (non-inoculation site)	Musculoskeletal and connective tissue disorders	Myalgia	Recruited//Non-Vaccination Site (Systemic)	November 6, 2021	November 7, 2021	November 11, 2021	5	1	No	Level 2	Self-medication	Probably related
Subject No.: 2854, Group: Inactivated Vaccine Group, Age (years): 49.9, Sex: Male, Nationality: Pakistan												

Adverse event description	System Organ Class	Preferred terms	Type of Adverse Event	Date of inoculation	Start Date	End date	Duration Days	Days from inoculation	Whether within 30min	Severity	Treatment situation	Correlation
Chest pain	General disorders and administration site conditions	Chest pain	Unsolicited	November 6, 2021	November 7, 2021	November 25, 2021	19	1	No	Grade 3	Hospitalization	May be relevant
Runny nose	Respiratory, thoracic and mediastinal diseases	Runny nose	Non-solicitation	November 6, 2021	November 7, 2021	November 10, 2021	4	1	No	Grade 2	Prescription drug	Possibly related
Fever: 39.0 °C	General disorders and administration site conditions	fever	Recruited//Non-Vaccination Site (Systemic)	November 6, 2021	November 7, 2021	November 25, 2021	19	1	No	Grade 3	Hospitalization	May be relevant
Cough	Respiratory, thoracic and mediastinal diseases	Cough	Collection//Non-inoculation site (whole body)	November 6, 2021	November 7, 2021	November 25, 2021	19	1	No	Grade 2	hospitalization	Possibly related
Subject No.: 2867, Group: Recombinant Vaccine Group, Age (years): 41.8, Sex: Male, Nationality: Bangladesh												
Fever: 38.2℃	Systemic diseases and various reactions at the administration site	Fever	Collection//Non-inoculation site (whole body)	November 7, 2021	November 7, 2021	November 7, 2021	1	0	No	Grade 1	Self-medication	Possibly related
Subject No.: 2868, Group: Recombinant Vaccine Group, Age (years): 46.8, Sex: Male, Nationality: Pakistan												
Pain	Systemic diseases and various reactions at the administration site	Vaccination site pain	Collection//inoculation site (local)	November 7, 2021	November 7, 2021	November 9, 2021	3	0	No	Grade 1	Untreated	Definitely related
Subject No.: 2875, Group: Inactivated Vaccine Group, Age (years): 46.8, Sex: Female, Nationality: Philippines												
Headache	Various nervous system diseases	Headache	Collection//Non-inoculation site (whole body)	November 7, 2021	November 7, 2021	November 8, 2021	2	0	No	Grade 1	Untreated	Probably related
Pain	General disorders and administration site conditions	Pain at vaccination site	Recruitment//injection site (local)	November 7, 2021	November 7, 2021	November 8, 2021	2	0	No	Grade 1	Untreated	Definitely relevant
Subject No.: 2879, Group: Inactivated Vaccine Group, Age (years): 49.9, Sex: Male, Nationality: Bangladesh												
Headache	Nervous system disorders	Headache	Recruited//Non-Vaccination Site (Systemic)	November 7, 2021	November 7, 2021	November 8, 2021	2	0	No	Grade 1	Untreated	May be relevant

Adverse event description	System Organ Class	Preferred terms	Type of Adverse Event	Date of inoculation	Start Date	End date	Duration Days	Days from inoculation	Whether within 30min	Severity	Treatment situation	Correlation
Muscle pain (non-injection site)	Various musculoskeletal and connective tissue diseases	Myalgia	Collection//Non-inoculation site (whole body)	November 7, 2021	November 7, 2021	November 7, 2021	1	0	No	Grade 1	Untreated	Possibly related
Abdominal pain	Gastrointestinal disorders	Abdominal pain	Unsolicited	November 7, 2021	November 8, 2021	November 9, 2021	2	1	No	Grade 1	Untreated	May be relevant
Fatigue/Asthenia	Systemic diseases and various reactions at the administration site	Fatigue	Collection//Non-inoculation site (whole body)	November 7, 2021	November 10, 2021	November 12, 2021	3	3	No	Grade 1	Untreated	Possibly related
Diarrhea	Gastrointestinal disorders	Diarrhea	Recruited//Non-Vaccination Site (Systemic)	November 7, 2021	November 10, 2021	November 12, 2021	3	3	No	Grade 1	Untreated	May be relevant
Subject No.: 2883, Group: Recombinant vaccine group, Age (years): 26.9, Sex: Male, Nationality: India												
Muscle pain (non-inoculation site)	Musculoskeletal and connective tissue disorders	Myalgia	Recruited//Non-Vaccination Site (Systemic)	November 7, 2021	November 9, 2021	November 9, 2021	1	2	No	Grade 1	Untreated	May be relevant
Subject No.: 2886, Group: Inactivated Vaccine Group, Age (years): 27.8, Sex: Male, Nationality: Bangladesh												
Fever: 38.3 ℃	General disorders and administration site conditions	Fever	Recruited//Non-Vaccination Site (Systemic)	November 7, 2021	November 8, 2021	November 9, 2021	2	1	No	Grade 1	Self-medication	Probably related
Pain	Systemic diseases and various reactions at the administration site	Vaccination site pain	Collection//inoculation site (local)	November 7, 2021	November 8, 2021	November 10, 2021	3	1	No	Grade 2	Self-medication	Definitely related
Muscle pain (non-inoculation site)	Musculoskeletal and connective tissue disorders	Myalgia	Recruited//Non-Vaccination Site (Systemic)	November 7, 2021	November 8, 2021	November 11, 2021	4	1	No	Level 2	Self-medication	Probably related
Subject No.: 2891, Group: Inactivated Vaccine Group, Age (years): 19.8, Sex: Male, Nationality: Syria												
pain	General disorders and administration site conditions	Pain at vaccination site	Recruitment//injection site (local)	November 7, 2021	November 7, 2021	November 8, 2021	2	0	No	Grade 1	Untreated	Definitely relevant

Adverse event description	System Organ Class	Preferred terms	Type of Adverse Event	Date of inoculation	Start Date	End date	Duration Days	Days from inoculation	Whether within 30min	Severity	Treatment situation	Correlation
Subject No.: 2895, Group: Recombinant vaccine group, Age (years): 29.7, Sex: Male, Nationality: Pakistan												
Headache	Nervous system disorders	Headache	Recruited//Non-Vaccination Site (Systemic)	November 7, 2021	November 7, 2021	November 8, 2021	2	0	No	Grade 1	Untreated	Probably related
Subject No.: 2902, Group: Inactivated Vaccine Group, Age (years): 36.9, Sex: Male, Nationality: Pakistan												
Sore throat	Respiratory, thoracic and mediastinal disorders	Oropharyngeal pain	Unsolicited	November 7, 2021	November 8, 2021	November 9, 2021	2	1	No	Grade 1	Untreated	May be relevant
Cough	Respiratory, thoracic and mediastinal diseases	Cough	Collection//Non-inoculation site (whole body)	November 7, 2021	November 8, 2021	November 9, 2021	2	1	No	Grade 1	Untreated	Possibly related
Subject number: 2910, group: recombinant vaccine group, age (years): 38, sex: male, nationality: India												
Headache	Various nervous system diseases	Headache	Collection//Non-inoculation site (whole body)	November 7, 2021	November 8, 2021	November 8, 2021	1	1	No	Grade 1	Untreated	Probably related
Fatigue/fatigue	General disorders and administration site conditions	Fatigue	Recruited//Non-Vaccination Site (Systemic)	November 7, 2021	November 8, 2021	November 9, 2021	2	1	No	Grade 1	Untreated	Probably related
Subject No.: 2911, Group: Inactivated Vaccine Group, Age (years): 26.4, Sex: Male, Nationality: India												
Pain	General disorders and administration site conditions	Pain at vaccination site	Recruitment//injection site (local)	November 7, 2021	November 8, 2021	November 10, 2021	3	1	No	Grade 1	Untreated	Definitely relevant
Subject number: 2914, group: recombinant vaccine group, age (years): 45.2, gender: male, nationality: Bangladesh												
Headache	Nervous system disorders	Headache	Recruited//Non-Vaccination Site (Systemic)	November 7, 2021	November 9, 2021	November 14, 2021	6	2	No	Grade 1	Untreated	Probably related
Subject number: 2928, group: recombinant vaccine group, age (years): 35.5, gender: male, nationality: Bangladesh												
Fatigue/fatigue	General disorders and administration site conditions	Fatigue	Recruited//Non-Vaccination Site (Systemic)	November 7, 2021	November 7, 2021	November 8, 2021	2	0	No	Grade 1	Untreated	May be relevant

Adverse event description	System Organ Class	Preferred terms	Type of Adverse Event	Date of inoculation	Start Date	End date	Duration Days	Days from inoculation	Whether within 30min	Severity	Treatment situation	Correlation
Pain	Systemic diseases and various reactions at the administration site	Vaccination site pain	Collection//inoculation site (local)	November 7, 2021	November 7, 2021	November 9, 2021	3	0	No	Grade 1	No medication in outpatient department	Definitely related
Cold Symptoms	Infections and infestations	Nasopharyngitis	Unsolicited	November 7, 2021	November 8, 2021	November 10, 2021	3	1	No	Grade 1	Untreated	May be relevant
Cough	Respiratory, thoracic and mediastinal diseases	Cough	Collection//Non-inoculation site (whole body)	November 7, 2021	November 9, 2021	November 10, 2021	2	2	No	Grade 1	Untreated	Possibly related
Subject No.: 2937, Group: Recombinant Vaccine Group, Age (years): 44.6, Sex: Male, Nationality: Bangladesh												
Pain	Systemic diseases and various reactions at the administration site	Vaccination site pain	Collection//inoculation site (local)	November 7, 2021	November 7, 2021	November 10, 2021	4	0	No	Grade 1	No medication in outpatient department	Definitely related
Muscle pain (non-inoculation site)	Musculoskeletal and connective tissue disorders	Myalgia	Recruited//Non-Vaccination Site (Systemic)	November 7, 2021	November 7, 2021	November 11, 2021	5	0	No	Grade 1	No medication in outpatient clinic	Probably related
pricking pain	Systemic diseases and various reactions at the administration site	Pain	Non-solicitation	November 7, 2021	November 12, 2021	November 13, 2021	2	5	No	Grade 1	No medication in outpatient department	Possibly related
Subject No.: 2941, Group: Recombinant Vaccine Group, Age (years): 37.7, Sex: Male, Nationality: Bangladesh												
Headache	Various nervous system diseases	Headache	Collection//Non-inoculation site (whole body)	November 7, 2021	November 10, 2021	November 13, 2021	4	3	No	Grade 1	No medication in outpatient department	Probably related
Subject No.: 2943, Group: Recombinant Vaccine Group, Age (years): 28.9, Sex: Male, Nationality: Pakistan												
Fever: 38.1℃	Systemic diseases and various reactions at the administration site	Fever	Collection//Non-inoculation site (whole body)	November 7, 2021	November 7, 2021	November 12, 2021	6	0	No	Grade 1	Self-medication	Probably related
Nausea	Gastrointestinal disorders	Nausea	Recruited//Non-Vaccination Site (Systemic)	November 7, 2021	November 7, 2021	November 12, 2021	6	0	No	Grade 1	Untreated	May be relevant

Adverse event description	System Organ Class	Preferred terms	Type of Adverse Event	Date of inoculation	Start Date	End date	Duration Days	Days from inoculation	Whether within 30min	Severity	Treatment situation	Correlation
Fatigue/Asthenia	Systemic diseases and various reactions at the administration site	Fatigue	Collection//Non-inoculation site (whole body)	November 7, 2021	November 7, 2021	November 22, 2021	16	0	No	Grade 2	Self-medication	Possibly related
Muscle pain (non-inoculation site)	Musculoskeletal and connective tissue disorders	Myalgia	Recruited//Non-Vaccination Site (Systemic)	November 7, 2021	November 7, 2021	November 12, 2021	6	0	No	Level 2	Self-medication	Probably related
Subject No.: 2944, Group: Inactivated Vaccine Group, Age (years): 28, Sex: Male, Nationality: Cameroon												
Headache	Nervous system disorders	Headache	Recruited//Non-Vaccination Site (Systemic)	November 7, 2021	November 7, 2021	November 9, 2021	3	0	No	Grade 1	Untreated	Probably related
Pain	Systemic diseases and various reactions at the administration site	Vaccination site pain	Collection//inoculation site (local)	November 7, 2021	November 7, 2021	November 9, 2021	3	0	No	Grade 1	Untreated	Definitely related
Subject No.: 2947, Group: Recombinant Vaccine Group, Age (years): 38.3, Sex: Male, Nationality: Bangladesh												
Pain	Systemic diseases and various reactions at the administration site	Vaccination site pain	Collection//inoculation site (local)	November 7, 2021	November 7, 2021	November 8, 2021	2	0	No	Grade 1	Untreated	Definitely related
Coughing	Respiratory, thoracic and mediastinal disorders	Coughing	Recruited//Non-Vaccination Site (Systemic)	November 7, 2021	November 10, 2021	November 15, 2021	6	3	No	Level 2	Untreated	Probably related
Subject number: 2952, group: recombinant vaccine group, age (years): 36.1, gender: male, nationality: Bangladesh												
Pain	General disorders and administration site conditions	Pain at vaccination site	Recruitment//injection site (local)	November 7, 2021	November 7, 2021	November 9, 2021	3	0	No	Grade 1	Untreated	Definitely relevant
Subject No.: 2956, Group: Inactivated Vaccine Group, Age (years): 23.3, Sex: Male, Nationality: Uganda												
Flu like symbols	General disorders and administration site conditions	Influenza-like disease	Unsolicited	November 8, 2021	November 8, 2021	November 8, 2021	1	0	No	Grade 1	Untreated	May be relevant

Adverse event description	System Organ Class	Preferred terms	Type of Adverse Event	Date of inoculation	Start Date	End date	Duration Days	Days from inoculation	Whether within 30min	Severity	Treatment situation	Correlation
Fever: 38.0℃	Systemic diseases and various reactions at the administration site	Fever	Collection//Non-inoculation site (whole body)	November 8, 2021	November 8, 2021	November 8, 2021	1	0	No	Grade 1	Self-medication	Definitely related
Headache	Nervous system disorders	Headache	Recruited//Non-Vaccination Site (Systemic)	November 8, 2021	November 8, 2021	November 8, 2021	1	0	No	Grade 1	Self-medication	May be relevant
Pain	Systemic diseases and various reactions at the administration site	Vaccination site pain	Collection//inoculation site (local)	November 8, 2021	November 8, 2021	November 9, 2021	2	0	No	Grade 1	Untreated	Definitely related
Subject No.: 2958, Group: Inactivated Vaccine Group, Age (years): 24.5, Sex: Male, Nationality: Bangladesh												
Pruritus	Skin and subcutaneous tissue diseases	Pruritus	Collection//inoculation site (local)	November 8, 2021	November 9, 2021	November 16, 2021	8	1	No	Grade 2	Untreated	Possibly related
Skin rash	Skin and subcutaneous tissue disorders	Skin rash	Recruitment//injection site (local)	November 8, 2021	November 10, 2021	November 17, 2021	8	2	No	Grade 1	Untreated	May be relevant
Subject No.: 2966, Group: Inactivated Vaccine Group, Age (years): 47.9, Sex: Male, Nationality: Pakistan												
Fever: 38.0 ℃	General disorders and administration site conditions	fever	Recruited//Non-Vaccination Site (Systemic)	November 8, 2021	November 8, 2021	November 10, 2021	3	0	No	Grade 1	Self-medication	Definitely relevant
Subject No.: 2969, Group: Recombinant vaccine group, Age (years): 36.9, Sex: Male, Nationality: Pakistan												
Pain	General disorders and administration site conditions	Pain at vaccination site	Recruitment//injection site (local)	November 8, 2021	November 8, 2021	November 10, 2021	3	0	No	Level 2	Self-medication	Definitely relevant
Sorethroat	Respiratory, thoracic and mediastinal diseases	Oropharyngeal pain	Non-solicitation	November 8, 2021	November 9, 2021	November 11, 2021	3	1	No	Grade 1	No medication in outpatient department	Possibly related
Fever: 37.8 ℃	General disorders and administration site conditions	fever	Recruited//Non-Vaccination Site (Systemic)	November 8, 2021	November 9, 2021	November 11, 2021	3	1	No	Grade 1	Self-medication	May be relevant

Adverse event description	System Organ Class	Preferred terms	Type of Adverse Event	Date of inoculation	Start Date	End date	Duration Days	Days from inoculation	Whether within 30min	Severity	Treatment situation	Correlation
Headache	Various nervous system diseases	Headache	Collection//Non-inoculation site (whole body)	November 8, 2021	November 9, 2021	November 11, 2021	3	1	No	Grade 2	Self-medication	Possibly related
Subject No.: 2994, Group: Inactivated Vaccine Group, Age (years): 39.7, Sex: Male, Nationality: India												
Muscle pain (non-injection site)	Various musculoskeletal and connective tissue diseases	Myalgia	Collection//Non-inoculation site (whole body)	November 8, 2021	November 8, 2021	November 8, 2021	1	0	No	Grade 1	Untreated	Probably related

3.2. Listing of adverse events not related to the study vaccine occurring during the booster period (SS)

Table 3-2 List of unrelated adverse events (SS) during immunization period

Adverse event description	System Organ Class	Preferred terms	Type of Adverse Event	Date of inoculation	Start Date	End date	Duration Days	Days from inoculation	Whether within 30min	Severity	Treatment situation	Correlation
Subject No.: 0052, Group: Inactivated Vaccine Group, Age (Y): 26, Sex: Male, Nationality: India												
Nasal conjecture	Respiratory, thoracic and mediastinal disorders	Nasal obstruction	Unsolicited	October 24, 2021	October 27, 2021	October 29, 2021	3	3	No	Grade 1	Untreated	May be irrelevant
Subject No.: 0130, Group: Recombinant vaccine group, Age (years): 35.8, Sex: Male, Nationality: Pakistan												
Fever: 37.8 ℃, HE SAID HE HAS FEVER BUT HE DIDNT CHECK	General disorders and administration site conditions	Fever	Unsolicited	October 26, 2021	November 17, 2021	November 20, 2021	4	22	No	Grade 1	Self-medication	Definitely irrelevant
Subject No.: 0242, Group: Recombinant Vaccine Group, Age (Y): 39.7, Sex: Male, Nationality: Bangladesh												

Adverse event description	System Organ Class	Preferred terms	Type of Adverse Event	Date of inoculation	Start Date	End date	Duration Days	Days from inoculation	Whether within 30min	Severity	Treatment situation	Correlation
Myalgia	Musculoskeletal and connective tissue disorders	Myalgia	Unsolicited	November 1, 2021	November 16, 2021	November 27, 2021	12	15	No	Grade 1	Untreated	May be irrelevant
Subject No.: 0301, Group: Recombinant vaccine group, Age (years): 39.2, Sex: Male, Nationality: Uganda												
Erectile dysfuction	Reproductive system and breast disorders	erectile dysfunctiom	Unsolicited	November 21-02, 2021	November 5, 2021	November 5, 2021	1	3	No	Grade 1	Untreated	May be irrelevant
Subject No.: 0329, Group: Recombinant vaccine group, Age (years): 56.1, Sex: Male, Nationality: Bangladesh												
Acute Allergic Reaction	immune system disease	Hypersensitivity reaction	Unsolicited	November 21-02, 2021	November 17, 2021	November 25, 2021	9	15	No	Grade 1	Untreated	May be irrelevant
Subject No.: 0376, Group: Inactivated Vaccine Group, Age (years): 37.8, Sex: Male, Nationality: Bangladesh												
Cough	Respiratory, thoracic and mediastinal disorders	Coughing	Unsolicited	November 3, 2021	November 22, 2021	December 1, 2021	10	19	No	Level 2	Prescription Drug	Definitely irrelevant
Subject No.: 0380, Group: Recombinant vaccine group, Age (years): 42.9, Sex: Male, Nationality: Uganda												
Headache	Nervous system disorders	Headache	Unsolicited	November 3, 2021	November 18, 2021	November 21, 2021	4	15	No	Grade 1	Untreated	May be irrelevant
Subject No.: 0409, Group: Recombinant vaccine group, Age (years): 46.5, Sex: Male, Nationality: Pakistan												
Fever: 38.9 ℃. Went to Oasis hospital and was given medicine/injections as stated.Resolved	General disorders and administration site conditions	fever	Unsolicited	November 3, 2021	December 2, 2021	December 3, 2021	2	29	No	Level 2	Outpatient medication	May be irrelevant
Subject No.: 0457, Group: Inactivated Vaccine Group, Age (years): 35.9, Sex: Male, Nationality: Uganda												

Adverse event description	System Organ Class	Preferred terms	Type of Adverse Event	Date of inoculation	Start Date	End date	Duration Days	Days from inoculation	Whether within 30min	Severity	Treatment situation	Correlation
Cough	Respiratory, thoracic and mediastinal disorders	coughing	Unsolicited	November 3, 2021	December 1, 2021	December 4, 2021	4	28	No	Level 2	Prescription Drug	May be irrelevant
Subject No.: 0474, Group: Recombinant vaccine group, Age (years): 31.2, Sex: Male, Nationality: Uganda												
Headache	Nervous system disorders	Headache	Unsolicited	November 3, 2021	December 2, 2021	December 3, 2021	2	29	No	Level 2	Self-medication	May be irrelevant
Subject No.: 0542, Group: Recombinant vaccine group, Age (years): 35.4, Sex: Male, Nationality: Bangladesh												
Feverish-Verbalized feeling having fever on 03/12/2021 night only, COVID PCR done with negative result	General disorders and administration site conditions	Fever	Unsolicited	November 4, 2021	December 3, 2021	December 3, 2021	1	29	No	Grade 1	Untreated	May be irrelevant
Subject No.: 0547, Group: Recombinant Vaccine Group, Age (Y): 43.5, Sex: Male, Nationality: Bangladesh												
Cough	Respiratory, thoracic and mediastinal disorders	coughing	Unsolicited	November 4, 2021	November 22, 2021			18	No	Level 2	Prescription Drug	May be irrelevant
Myalgia	Various musculoskeletal and connective tissue diseases	Myalgia	Non-solicitation	November 4, 2021	November 22, 2021			18	No	Grade 2	Prescription drug	Unlikely
Subject No.: 0555, Group: Inactivated Vaccine Group, Age (years): 29.7, Sex: Male, Nationality: India												
Fever: 37.8℃, Tympanic	Systemic diseases and various reactions at the administration site	Fever	Non-solicitation	November 4, 2021	November 21, 2021	November 23, 2021	3	17	No	Grade 1	Self-medication	Unlikely
Subject No.: 0557, Group: Recombinant Vaccine Group, Age (years): 41.2, Sex: Male, Nationality: Bangladesh												

Adverse event description	System Organ Class	Preferred terms	Type of Adverse Event	Date of inoculation	Start Date	End date	Duration Days	Days from inoculation	Whether within 30min	Severity	Treatment situation	Correlation
Myalgia	Various musculoskeletal and connective tissue diseases	Myalgia	Non-solicitation	November 4, 2021	November 22, 2021			18	No	Grade 2	Self-medication	Unlikely
Sore throat	Respiratory, thoracic and mediastinal disorders	Oropharyngeal pain	Unsolicited	November 4, 2021	November 22, 2021			18	No	Level 2	Self-medication	May be irrelevant
Subject No.: 0588, Group: Inactivated Vaccine Group, Age (years): 41.8, Sex: Male, Nationality: Sudan												
Nausea	Gastrointestinal disorders	Nausea	Recruited//Non-Vaccination Site (Systemic)	November 4, 2021	November 6, 2021	November 8, 2021	3	2	No	Level 2	Prescription Drug	Definitely irrelevant
Subject No.: 0591, Group: Recombinant vaccine group, Age (years): 33.4, Sex: Male, Nationality: Philippines												
Myalgia	Musculoskeletal and connective tissue disorders	Myalgia	Unsolicited	November 4, 2021	November 22, 2021	November 25, 2021	4	18	No	Grade 3	Prescription Drug	Definitely irrelevant
Subject No.: 1205, Group: Inactivated Vaccine Group, Age (years): 18.8, Sex: Male, Nationality: Egypt												
Subjective ever	General disorders and administration site conditions	fever	Unsolicited	October 23, 2021	November 14, 2021	November 18, 2021	5	22	No	Grade 1	Untreated	May be irrelevant
Subject No.: 1218, Group: Inactivated Vaccine Group, Age (years): 31.8, Sex: Female, Nationality: Uganda												
Dysphagia	Gastrointestinal disorders	Dysphagia	Recruited//Non-Vaccination Site (Systemic)	October 24, 2021	October 24, 2021	October 24, 2021	1	0	Yes	Grade 1	Outpatient medication	Definitely irrelevant
Subject No.: 1232, Group: Inactivated Vaccine Group, Age (years): 43.8, Sex: Male, Nationality: Pakistan												
ELEVATED BLOOD PRESSURE	Investigations	Elevated blood pressure	Unsolicited	October 24, 2021	October 25, 2021	October 26, 2021	2	1	No	Grade 1	Untreated	May be irrelevant
Subject No.: 1293, Group: Recombinant vaccine group, Age (years): 35.8, Sex: Male, Nationality: Pakistan												
Fever: 37.8 ℃	General disorders and administration site conditions	fever	Unsolicited	October 24, 2021	November 22, 2021	November 23, 2021	2	29	No	Grade 1	Self-medication	May be irrelevant

Adverse event description	System Organ Class	Preferred terms	Type of Adverse Event	Date of inoculation	Start Date	End date	Duration Days	Days from inoculation	Whether within 30min	Severity	Treatment situation	Correlation
Subject No.: 1301, Group: Recombinant vaccine group, Age (years): 50.9, Sex: Female, Nationality: Philippines												
Anxiety	Psychiatric disorders	Anxiety	Unsolicited	October 25, 2021	October 25, 2021	October 28, 2021	4	0	No	Level 2	Untreated	May be irrelevant
Subject number: 1361, group: recombinant vaccine group, age (years): 37.6, gender: male, nationality: Bangladesh												
Dizziness	Nervous system disorders	Dizziness	Unsolicited	October 25, 2021	November 21, 2021	November 30, 2021	10	27	No	Grade 1	Untreated	Definitely irrelevant
Subject No.: 1519, Group: Recombinant Vaccine Group, Age (Y): 25.2, Sex: Male, Nationality: Bangladesh												
Cough	Respiratory, thoracic and mediastinal disorders	coughing	Unsolicited	November 1, 2021	November 20, 2021	November 24, 2021	5	19	No	Grade 1	Untreated	May be irrelevant
Fever: 37.8℃	Systemic diseases and various reactions at the administration site	Fever	Non-solicitation	November 1, 2021	November 21, 2021	November 24, 2021	4	20	No	Grade 1	Self-medication	Unlikely
Subject number: 1520, group: recombinant vaccine group, age (years): 38.8, sex: male, nationality: Bangladesh												
Myalgia	Various musculoskeletal and connective tissue diseases	Myalgia	Non-solicitation	November 1, 2021	November 19, 2021			18	No	Grade 2	Self-medication	Unlikely
Subject number: 1544, group: recombinant vaccine group, age (years): 31.8, sex: male, nationality: Pakistan												
Elevated Blood pressure	Various inspections	Blood pressure increased	Non-solicitation	November 1, 2021	November 1, 2021	November 5, 2021	5	0	No	Grade 1	Untreated	Unlikely
Subject number: 1568, group: inactivated vaccine group, age (years): 37.3, sex: male, nationality: Eritrea												
Cough	Respiratory, thoracic and mediastinal diseases	Cough	Non-solicitation	November 2, 2021	November 21, 2021	November 25, 2021	5	19	No	Grade 1	Untreated	Unlikely
Headache	Nervous system disorders	Headache	Unsolicited	November 21-02, 2021	November 21, 2021	November 25, 2021	5	19	No	Level 2	Self-medication	May be irrelevant

Adverse event description	System Organ Class	Preferred terms	Type of Adverse Event	Date of inoculation	Start Date	End date	Duration Days	Days from inoculation	Whether within 30min	Severity	Treatment situation	Correlation
Subject No.: 1577, Group: Inactivated Vaccine Group, Age (years): 43.8, Sex: Female, Nationality: Philippines												
Nodule at the back of left ear	General disorders and administration site conditions	Nodule	Unsolicited	November 21-02, 2021	November 18, 2021	November 19, 2021	2	16	No	Grade 1	Untreated	May be irrelevant
Subject No.: 1591, Group: Inactivated Vaccine Group, Age (years): 47.7, Sex: Male, Nationality: Bangladesh												
Headache	Nervous system disorders	Headache	Unsolicited	November 21-02, 2021	November 25, 2021	December 10, 2021	16	23	No	Level 2	Untreated	May be irrelevant
Subject No.: 1614, Group: Recombinant vaccine group, Age (years): 20.8, Sex: Male, Nationality: India												
Cough	Respiratory, thoracic and mediastinal disorders	coughing	Unsolicited	November 3, 2021	December 1, 2021			28	No	Level 2	Self-medication	May be irrelevant
RUNNY NOSE	Respiratory, thoracic and mediastinal diseases	Runny nose	Non-solicitation	November 3, 2021	December 1, 2021			28	No	Grade 2	Self-medication	Unlikely
Subject No.: 1618, Group: Recombinant Vaccine Group, Age (years): 34.5, Sex: Male, Nationality: Bangladesh												
Cough	Respiratory, thoracic and mediastinal diseases	Cough	Non-solicitation	November 3, 2021	December 1, 2021			28	No	Grade 1	No medication in outpatient department	Unlikely
Subject No.: 1641, Group: Recombinant Vaccine Group, Age (years): 41.1, Sex: Male, Nationality: Egypt												
Cough	Respiratory, thoracic and mediastinal diseases	Cough	Non-solicitation	November 3, 2021	November 12, 2021	November 26, 2021	15	9	No	Grade 3	Prescription drug	Unlikely
Fatigue, Asthenia	General disorders and administration site conditions	Fatigue	Unsolicited	November 3, 2021	November 12, 2021	November 26, 2021	15	9	No	Grade 3	Prescription Drug	May be irrelevant
sore throat	Respiratory, thoracic and mediastinal diseases	Oropharyngeal pain	Non-solicitation	November 3, 2021	November 12, 2021	November 26, 2021	15	9	No	Grade 3	Prescription drug	Unlikely
Subject No.: 1646, Group: Inactivated Vaccine Group, Age (years): 51.6, Sex: Male, Nationality: Bangladesh												

Adverse event description	System Organ Class	Preferred terms	Type of Adverse Event	Date of inoculation	Start Date	End date	Duration Days	Days from inoculation	Whether within 30min	Severity	Treatment situation	Correlation
Dizzines	Various nervous system diseases	Dizziness	Non-solicitation	November 3, 2021	December 2, 2021	December 2, 2021	1	29	No	Grade 1	No medication in outpatient department	Unlikely
Subject number: 1667, group: recombinant vaccine group, age (years): 24.8, sex: male, nationality: Pakistan												
Headache	Various nervous system diseases	Headache	Non-solicitation	November 3, 2021	December 2, 2021	December 5, 2021	4	29	No	Grade 1	Untreated	Unlikely
Subject number: 1680, group: recombinant vaccine group, age (years): 32.6, sex: male, nationality: Bangladesh												
Fatigue, Asthenia	Systemic diseases and various reactions at the administration site	Fatigue	Non-solicitation	November 3, 2021	December 2, 2021	December 5, 2021	4	29	No	Grade 1	Untreated	Unlikely
Headache	Nervous system disorders	Headache	Unsolicited	November 3, 2021	December 2, 2021	December 5, 2021	4	29	No	Level 2	Self-medication	May be irrelevant
subjective fever	Systemic diseases and various reactions at the administration site	Fever	Non-solicitation	November 3, 2021	December 2, 2021	December 5, 2021	4	29	No	Grade 1	Untreated	Unlikely
Subject No.: 1704, Group: Inactivated Vaccine Group, Age (years): 28.8, Sex: Male, Nationality: Egypt												
Fatigue, Asthenia	Systemic diseases and various reactions at the administration site	Fatigue	Non-solicitation	November 4, 2021	December 3, 2021			29	No	Grade 1	Self-medication	Unlikely
Headache	Nervous system disorders	Headache	Unsolicited	November 4, 2021	December 3, 2021			29	No	Level 2	Self-medication	May be irrelevant
nasal congestion	Respiratory, thoracic and mediastinal diseases	Nasal congestion	Non-solicitation	November 4, 2021	December 3, 2021			29	No	Grade 1	Self-medication	Unlikely
Subject No.: 1706, Group: Recombinant Vaccine Group, Age (years): 46.1, Sex: Male, Nationality: India												
FEELS SLEEPY	Various nervous system diseases	Sleepiness	Non-solicitation	November 4, 2021	November 4, 2021	November 10, 2021	7	0	No	Grade 1	Untreated	Unlikely
Subject No.: 1774, Group: Recombinant Vaccine Group, Age (years): 33.2, Sex: Male, Nationality: Nepal												

Adverse event description	System Organ Class	Preferred terms	Type of Adverse Event	Date of inoculation	Start Date	End date	Duration Days	Days from inoculation	Whether within 30min	Severity	Treatment situation	Correlation
Fever: 38.0℃	Systemic diseases and various reactions at the administration site	Fever	Non-solicitation	November 4, 2021	December 2, 2021	December 4, 2021	3	28	No	Grade 1	Self-medication	Unlikely
Runny Nose	Respiratory, thoracic and mediastinal disorders	Runny nose	Unsolicited	November 4, 2021	December 2, 2021	December 8, 2021	7	28	No	Level 2	Self-medication	May be irrelevant
Subject No.: 1784, Group: Recombinant vaccine group, Age (years): 38.6, Sex: Male, Nationality: Pakistan												
Fever: 38.0 ℃, On and off ever for period of 1 month. Visited NMC 3 times. Paracetamol 1000mg was prescribed 3x a day.Dompy 10mg daily	General disorders and administration site conditions	fever	Unsolicited	November 4, 2021	December 3, 2021	January 3, 2022	32	29	No	Grade 1	Prescription Drug	May be irrelevant
Fatigue, Asthenia	Systemic diseases and various reactions at the administration site	Fatigue	Non-solicitation	November 4, 2021	December 4, 2021	December 6, 2021	3	30	No	Grade 1	Untreated	Unlikely
Headache	Nervous system disorders	Headache	Unsolicited	November 4, 2021	December 4, 2021	December 6, 2021	3	30	No	Grade 1	Untreated	May be irrelevant
Subject No.: 1790, Group: Inactivated Vaccine Group, Age (years): 29.4, Sex: Male, Nationality: Ghana												
Myalgia	Musculoskeletal and connective tissue disorders	Myalgia	Unsolicited	November 4, 2021	November 27, 2021			23	No	Grade 1	Self-medication	Definitely irrelevant
Subject No.: 1803, Group: Recombinant vaccine group, Age (years): 30.9, Sex: Male, Nationality: Bangladesh												

Adverse event description	System Organ Class	Preferred terms	Type of Adverse Event	Date of inoculation	Start Date	End date	Duration Days	Days from inoculation	Whether within 30min	Severity	Treatment situation	Correlation
Cough	Respiratory, thoracic and mediastinal disorders	coughing	Unsolicited	November 4, 2021	December 3, 2021	December 6, 2021	4	29	No	Grade 1	Untreated	May be irrelevant
Fatigue, Asthenia	Systemic diseases and various reactions at the administration site	Fatigue	Non-solicitation	November 4, 2021	December 3, 2021	December 6, 2021	4	29	No	Grade 1	Untreated	Unlikely
Subject No.: 1806, Group: Recombinant Vaccine Group, Age (years): 37.8, Sex: Male, Nationality: Bangladesh												
Intermittent numbers in the left leg	Various nervous system diseases	Hypoaesthesia	Non-solicitation	November 4, 2021	November 18, 2021	December 1, 2021	14	14	No	Grade 2	Untreated	Unlikely
Subject No.: 1810, Group: Inactivated Vaccine Group, Age (years): 28.8, Sex: Male, Nationality: Pakistan												
Cough	Respiratory, thoracic and mediastinal diseases	Cough	Non-solicitation	November 4, 2021	November 30, 2021	December 7, 2021	8	26	No	Grade 1	Untreated	Not related
Subject No.: 2445, Group: Inactivated Vaccine Group, Age (years): 56.8, Sex: Male, Nationality: Jordan												
Tooth ache	Gastrointestinal system diseases	Toothache	Non-solicitation	October 26, 2021	November 2, 2021	November 3, 2021	2	7	No	Grade 2	Prescription drug	Unlikely
Subject No.: 2510, Group: Inactivated Vaccine Group, Age (years): 54.2, Sex: Male, Nationality: Bangladesh												
Difficulty breathing	Respiratory, thoracic and mediastinal diseases	Difficulty breathing	Collection//Non-inoculation site (whole body)	November 2, 2021	November 3, 2021	November 20, 2021	18	1	No	Grade 2	Untreated	Unlikely
Subject No.: 2511, Group: Recombinant Vaccine Group, Age (years): 35, Sex: Male, Nationality: Ghana												
COLDS	Infection and infectious diseases	Nasopharyngitis	Non-solicitation	November 2, 2021	November 25, 2021			23	No	Grade 1	Untreated	Unlikely
Subject No.: 2515, Group: Inactivated Vaccine Group, Age (years): 56.5, Sex: Male, Nationality: Bangladesh												
pain on left leg (knee pain)	Various musculoskeletal and connective tissue diseases	Arthralgia	Non-solicitation	November 2, 2021	November 23, 2021			21	No	Grade 2	Self-medication	Unlikely
Subject No.: 2572, Group: Recombinant Vaccine Group, Age (years): 38.7, Sex: Male, Nationality: Bangladesh												

Adverse event description	System Organ Class	Preferred terms	Type of Adverse Event	Date of inoculation	Start Date	End date	Duration Days	Days from inoculation	Whether within 30min	Severity	Treatment situation	Correlation
Fever: 38.0℃, TYMPANIC	Systemic diseases and various reactions at the administration site	Fever	Non-solicitation	November 4, 2021	November 30, 2021	December 2, 2021	3	26	No	Grade 1	Self-medication	Unlikely
Subject No.: 2576, Group: Recombinant Vaccine Group, Age (years): 39.1, Sex: Male, Nationality: Bangladesh												
Myalgia	Various musculoskeletal and connective tissue diseases	Myalgia	Non-solicitation	November 4, 2021	November 12, 2021	November 24, 2021	13	8	No	Grade 1	Untreated	Unlikely
Subject No.: 2594, Group: Recombinant Vaccine Group, Age (years): 36.5, Sex: Male, Nationality: Uganda												
Cough	Respiratory, thoracic and mediastinal diseases	Cough	Non-solicitation	November 4, 2021	December 2, 2021	December 5, 2021	4	28	No	Grade 2	Untreated	Unlikely
Headache	Nervous system disorders	Headache	Unsolicited	November 4, 2021	December 2, 2021	December 5, 2021	4	28	No	Level 2	Untreated	May be irrelevant
Subject number: 2619, group: recombinant vaccine group, age (years): 31.8, gender: male, nationality: Bangladesh												
Acute Allergic Reaction	immune system disease	Hypersensitivity reaction	Unsolicited	November 4, 2021	November 18, 2021	November 25, 2021	8	14	No	Grade 1	Untreated	May be irrelevant
Pruritus	Systemic diseases and various reactions at the administration site	Vaccination site pruritus	Non-solicitation	November 4, 2021	November 22, 2021	November 25, 2021	4	18	No	Grade 2	Untreated	Unlikely
Subject number: 2726, group: recombinant vaccine group, age (years): 26.4, sex: male, nationality: Uganda												
Acute Allergic Reaction	immune system disease	Hypersensitivity reaction	Non-solicitation	November 5, 2021	November 25, 2021	November 27, 2021	3	20	No	Grade 1	Untreated	Unlikely
Subject No.: 2743, Group: Inactivated Vaccine Group, Age (years): 26.8, Sex: Male, Nationality: Pakistan												
Runny nose	Respiratory, thoracic and mediastinal diseases	Runny nose	Non-solicitation	November 5, 2021	November 22, 2021	November 24, 2021	3	17	No	Grade 1	Untreated	Unlikely
Subject No.: 2763, Group: Inactivated Vaccine Group, Age (years): 25.7, Sex: Male, Nationality: Pakistan												

Adverse event description	System Organ Class	Preferred terms	Type of Adverse Event	Date of inoculation	Start Date	End date	Duration Days	Days from inoculation	Whether within 30min	Severity	Treatment situation	Correlation
Dysphagia	Gastrointestinal system diseases	Dysphagia	Collection//Non-inoculation site (whole body)	November 5, 2021	November 5, 2021	November 5, 2021	1	0	Yes	Grade 1	Outpatient medication	Not related
Subject No.: 2784, Group: Inactivated Vaccine Group, Age (years): 24.7, Sex: Male, Nationality: Pakistan												
Myalgia	Various musculoskeletal and connective tissue diseases	Myalgia	Non-solicitation	November 6, 2021	November 30, 2021	December 7, 2021	8	24	No	Grade 2	Untreated	Unlikely
Subject No.: 2805, Group: Recombinant Vaccine Group, Age (years): 41.8, Sex: Male, Nationality: India												
Cough	Respiratory, thoracic and mediastinal diseases	Cough	Collection//Non-inoculation site (whole body)	November 6, 2021	November 7, 2021	November 18, 2021	12	1	No	Grade 4	hospitalization	Unlikely
Headache	Nervous system disorders	Headache	Recruited//Non-Vaccination Site (Systemic)	November 6, 2021	November 7, 2021	November 18, 2021	12	1	No	Grade 3	Hospitalization	May be irrelevant
Fatigue/Asthenia	Systemic diseases and various reactions at the administration site	Fatigue	Collection//Non-inoculation site (whole body)	November 6, 2021	November 7, 2021	November 18, 2021	12	1	No	Grade 3	hospitalization	Unlikely
Muscle pain (non-inoculation site)	Musculoskeletal and connective tissue disorders	Myalgia	Recruited//Non-Vaccination Site (Systemic)	November 6, 2021	November 7, 2021	November 18, 2021	12	1	No	Grade 3	Hospitalization	May be irrelevant
Nausea	Gastrointestinal system diseases	Nausea	Collection//Non-inoculation site (whole body)	November 6, 2021	November 9, 2021	November 15, 2021	7	3	No	Grade 3	hospitalization	Unlikely
Chills	General disorders and administration site conditions	Chills	Unsolicited	November 6, 2021	November 10, 2021	November 18, 2021	9	4	No	Grade 3	Hospitalization	May be irrelevant
Hematuria	Diseases of kidney and urinary system	Hematuria	Non-solicitation	November 6, 2021	November 10, 2021	November 18, 2021	9	4	No	Grade 3	hospitalization	Unlikely
Hemoptysis	Respiratory, thoracic and mediastinal disorders	Hemoptysis	Unsolicited	November 6, 2021	November 10, 2021	November 18, 2021	9	4	No	Grade 3	Hospitalization	May be irrelevant

Adverse event description	System Organ Class	Preferred terms	Type of Adverse Event	Date of inoculation	Start Date	End date	Duration Days	Days from inoculation	Whether within 30min	Severity	Treatment situation	Correlation
Loss of Smell	Various nervous system diseases	Loss of smell	Non-solicitation	November 6, 2021	November 10, 2021	November 14, 2021	5	4	No	Grade 3	hospitalization	Unlikely
Loss of Taste	Nervous system disorders	Loss of taste	Unsolicited	November 6, 2021	November 10, 2021	November 14, 2021	5	4	No	Grade 3	Hospitalization	May be irrelevant
Subject number: 2806, group: recombinant vaccine group, age (years): 42.9, gender: male, nationality: Bangladesh												
Fatigue, Asthenia	General disorders and administration site conditions	Fatigue	Unsolicited	November 6, 2021	November 27, 2021	November 29, 2021	3	21	No	Level 2	Prescription Drug	May be irrelevant
Fever: 38.0℃, RESOLVED	Systemic diseases and various reactions at the administration site	Fever	Non-solicitation	November 6, 2021	November 27, 2021	November 29, 2021	3	21	No	Grade 1	Prescription drug	Unlikely
Subject No.: 2811, Group: Inactivated Vaccine Group, Age (years): 49.9, Sex: Male, Nationality: Pakistan												
Slurred Speech	Various nervous system diseases	Dysarthria	Non-solicitation	November 6, 2021	December 5, 2021			29	No	Grade 2	Untreated	Unlikely
Subject No.: 2816, Group: Recombinant Vaccine Group, Age (years): 48.2, Sex: Male, Nationality: Bangladesh												
Cough	Respiratory, thoracic and mediastinal diseases	Cough	Non-solicitation	November 6, 2021	November 24, 2021	November 28, 2021	5	18	No	Grade 1	Untreated	Unlikely
Fever: 38.0 ℃, VIA TEMPORALRESOLVED	General disorders and administration site conditions	fever	Unsolicited	November 6, 2021	2021-11-24	November 25, 2021	2	18	No	Grade 1	Untreated	May be irrelevant
Subject number: 2844, group: recombinant vaccine group, age (years): 36.2, gender: male, nationality: Bangladesh												
Myalgia	Musculoskeletal and connective tissue disorders	Myalgia	Unsolicited	November 6, 2021	November 25, 2021	December 25, 2021	31	19	No	Grade 2	Prescription Drug	May be irrelevant
Subject No.: 2848, Group: Inactivated Vaccine Group, Age (years): 46, Sex: Male, Nationality: Philippines												

Adverse event description	System Organ Class	Preferred terms	Type of Adverse Event	Date of inoculation	Start Date	End date	Duration Days	Days from inoculation	Whether within 30min	Severity	Treatment situation	Correlation
Cough	Respiratory, thoracic and mediastinal disorders	coughing	Unsolicited	November 6, 2021	November 24, 2021			18	No	Grade 2	Prescription Drug	May be irrelevant
Fever: 37.8℃, tympanic	Systemic diseases and various reactions at the administration site	Fever	Non-solicitation	November 6, 2021	November 24, 2021			18	No	Grade 1	Prescription drug	Unlikely
Sore throat	Respiratory, thoracic and mediastinal disorders	Oropharyngeal pain	Unsolicited	November 6, 2021	November 24, 2021			18	No	Grade 2	Prescription Drug	May be irrelevant
Subject No.: 2879, Group: Inactivated Vaccine Group, Age (years): 49.9, Sex: Male, Nationality: Bangladesh												
Stomach pain	Gastrointestinal disorders	Epigastric pain	Unsolicited	November 7, 2021	November 8, 2021	November 10, 2021	3	1	No	Grade 2	Untreated	May be irrelevant
Subject number: 2928, group: recombinant vaccine group, age (years): 35.5, gender: male, nationality: Bangladesh												
Cough	Respiratory, thoracic and mediastinal disorders	coughing	Unsolicited	November 7, 2021	November 25, 2021			18	No	Grade 2	Self-medication	May be irrelevant
Subject No.: 2943, Group: Recombinant vaccine group, Age (years): 28.9, Sex: Male, Nationality: Pakistan												
Myalgia	Musculoskeletal and connective tissue disorders	Myalgia	Unsolicited	November 7, 2021	December 7, 2021	December 10, 2021	4	30	No	Grade 2	Outpatient medication	May be irrelevant
Subject No.: 2944, Group: Inactivated Vaccine Group, Age (years): 28, Sex: Male, Nationality: Cameroon												
Cough	Respiratory, thoracic and mediastinal disorders	coughing	Unsolicited	November 7, 2021	December 5, 2021	December 7, 2021	3	28	No	Grade 1	Untreated	May be irrelevant
Subject Number: 2980, Group: Inactivated Vaccine Group, Age (Y): 35.9, Sex: Male, Nationality: Bangladesh												

Adverse event description	System Organ Class	Preferred terms	Type of Adverse Event	Date of inoculation	Start Date	End date	Duration Days	Days from inoculation	Whether within 30min	Severity	Treatment situation	Correlation
Increased Urination spcially Nocturia (3-4 times) at night	Renal and urinary disorders	Frequency of urination	Unsolicited	November 8, 2021	November 11, 2021	November 16, 2021	6	3	No	Grade 2	Untreated	May be irrelevant
Subject No.: 2990, Group: Inactivated Vaccine Group, Age (years): 29.9, Sex: Male, Nationality: Bangladesh												
Chest pain	General disorders and administration site conditions	Chest pain	Unsolicited	November 8, 2021	November 18, 2021	November 22, 2021	5	10	No	Grade 3	Hospitalization	May be irrelevant

3.3. Listing of adverse events of grade 3 or higher severity occurring during the boost period (SS)

Table 3-3 List of adverse events with severity grade 3 and above (SS) during the exemption period

Description of Adverse Events	Systematic organ classification	Preferred Term	Types of adverse events	Date of inoculation	Start date	End Date	Duration days	Days from vaccination	Is it within 30min	Severity	Treatment status	Correlation
Subject No.: 0068, Group: Recombinant Vaccine Group, Age (years): 27.8, Sex: Male, Nationality: Bangladesh												
Fever: 39.0℃	Systemic diseases and various reactions at the administration site	Fever	Collection//Non-inoculation site (whole body)	October 24, 2021	October 24, 2021	October 31, 2021	8	0	No	Grade 3	Outpatient medication	Possibly related
Subject No.: 0588, Group: Inactivated Vaccine Group, Age (years): 41.8, Sex: Male, Nationality: Sudan												
Fever: 39.0℃	Systemic diseases and various reactions at the administration site	Fever	Collection//Non-inoculation site (whole body)	November 4, 2021	November 5, 2021	November 6, 2021	2	1	No	Grade 3	Outpatient medication	Probably related

Description of Adverse Events	Systematic organ classification	Preferred Term	Types of adverse events	Date of inoculation	Start date	End Date	Duration days	Days from vaccination	Is it within 30min	Severity	Treatment status	Correlation
Muscle pain (non-inoculation site)	Musculoskeletal and connective tissue disorders	Myalgia	Recruited//Non-Vaccination Site (Systemic)	November 4, 2021	November 5, 2021	November 8, 2021	4	1	No	Grade 3	Outpatient medication	Probably related
Subject No.: 0591, Group: Recombinant vaccine group, Age (years): 33.4, Sex: Male, Nationality: Philippines												
Myalgia	Musculoskeletal and connective tissue disorders	Myalgia	Unsolicited	November 4, 2021	November 22, 2021	November 25, 2021	4	18	No	Grade 3	Prescription Drug	Definitely irrelevant
Subject number: 1555, group: recombinant vaccine group, age (years): 35.7, gender: male, nationality: Bangladesh												
Myalgia	Musculoskeletal and connective tissue disorders	Myalgia	Unsolicited	November 2, 2021	November 27, 2021	November 29, 2021	3	25	No	Grade 3	Prescription Drug	Probably related
Subject number: 1641, group: recombinant vaccine group, age (years): 41.1, gender: male, nationality: Egypt												
Muscle pain (non-inoculation site)	Musculoskeletal and connective tissue disorders	Myalgia	Recruited//Non-Vaccination Site (Systemic)	November 3, 2021	November 7, 2021	November 26, 2021	20	4	No	Grade 3	Prescription Drug	May be relevant
Cough	Respiratory, thoracic and mediastinal diseases	Cough	Non-solicitation	November 3, 2021	November 12, 2021	November 26, 2021	15	9	No	Grade 3	Prescription drug	Unlikely
Fatigue, Asthenia	General disorders and administration site conditions	Fatigue	Unsolicited	November 3, 2021	November 12, 2021	November 26, 2021	15	9	No	Grade 3	Prescription Drug	May be irrelevant
sore throat	Respiratory, thoracic and mediastinal diseases	Oropharyngeal pain	Non-solicitation	November 3, 2021	November 12, 2021	November 26, 2021	15	9	No	Grade 3	Prescription drug	Unlikely
Subject No.: 2625, Group: Recombinant Vaccine Group, Age (years): 46.2, Sex: Male, Nationality: Bangladesh												
Fever: 40.0℃	Systemic diseases and various reactions at the administration site	Fever	Collection//Non-inoculation site (whole body)	November 4, 2021	November 4, 2021	November 10, 2021	7	0	No	Grade 3	Self-medication	Probably related
Subject No.: 2805, Group: Recombinant Vaccine Group, Age (years): 41.8, Sex: Male, Nationality: India												

Description of Adverse Events	Systematic organ classification	Preferred Term	Types of adverse events	Date of inoculation	Start date	End Date	Duration days	Days from vaccination	Is it within 30min	Severity	Treatment status	Correlation
Sore throat	Respiratory, thoracic and mediastinal diseases	Oropharyngeal pain	Non-solicitation	November 6, 2021	November 7, 2021	November 18, 2021	12	1	No	Grade 3	hospitalization	Probably related
Fever: 39.1℃, tympanic	General disorders and administration site conditions	Fever	Recruited//Non-Vaccination Site (Systemic)	November 6, 2021	November 7, 2021	November 15, 2021	9	1	No	Grade 3	Hospitalization	Probably related
Cough	Respiratory, thoracic and mediastinal diseases	Cough	Collection//Non-inoculation site (whole body)	November 6, 2021	November 7, 2021	November 18, 2021	12	1	No	Grade 4	hospitalization	Unlikely
Headache	Nervous system disorders	Headache	Recruited//Non-Vaccination Site (Systemic)	November 6, 2021	November 7, 2021	November 18, 2021	12	1	No	Grade 3	Hospitalization	May be irrelevant
Fatigue/Asthenia	Systemic diseases and various reactions at the administration site	Fatigue	Collection//Non-inoculation site (whole body)	November 6, 2021	November 7, 2021	November 18, 2021	12	1	No	Grade 3	hospitalization	Unlikely
Muscle pain (non-inoculation site)	Musculoskeletal and connective tissue disorders	Myalgia	Recruited//Non-Vaccination Site (Systemic)	November 6, 2021	November 7, 2021	November 18, 2021	12	1	No	Grade 3	Hospitalization	May be irrelevant
Nausea	Gastrointestinal system diseases	Nausea	Collection//Non-inoculation site (whole body)	November 6, 2021	November 9, 2021	November 15, 2021	7	3	No	Grade 3	hospitalization	Unlikely
Chills	General disorders and administration site conditions	Chills	Unsolicited	November 6, 2021	November 10, 2021	November 18, 2021	9	4	No	Grade 3	Hospitalization	May be irrelevant
Hematuria	Diseases of kidney and urinary system	Hematuria	Non-solicitation	November 6, 2021	November 10, 2021	November 18, 2021	9	4	No	Grade 3	hospitalization	Unlikely
Hemoptysis	Respiratory, thoracic and mediastinal disorders	Hemoptysis	Unsolicited	November 6, 2021	November 10, 2021	November 18, 2021	9	4	No	Grade 3	Hospitalization	May be irrelevant
Loss of Smell	Various nervous system diseases	Loss of smell	Non-solicitation	November 6, 2021	November 10, 2021	November 14, 2021	5	4	No	Grade 3	hospitalization	Unlikely

Description of Adverse Events	Systematic organ classification	Preferred Term	Types of adverse events	Date of inoculation	Start date	End Date	Duration days	Days from vaccination	Is it within 30min	Severity	Treatment status	Correlation
Loss of Taste	Nervous system disorders	Loss of taste	Unsolicited	November 6, 2021	November 10, 2021	November 14, 2021	5	4	No	Grade 3	Hospitalization	May be irrelevant
Subject No.: 2854, Group: Inactivated Vaccine Group, Age (years): 49.9, Sex: Male, Nationality: Pakistan												
Chest pain	General disorders and administration site conditions	Chest pain	Unsolicited	November 6, 2021	November 7, 2021	November 25, 2021	19	1	No	Grade 3	Hospitalization	May be relevant
Fever: 39.0℃	Systemic diseases and various reactions at the administration site	Fever	Collection//Non-inoculation site (whole body)	November 6, 2021	November 7, 2021	November 25, 2021	19	1	No	Grade 3	hospitalization	Possibly related
Subject No.: 2990, Group: Inactivated Vaccine Group, Age (years): 29.9, Sex: Male, Nationality: Bangladesh												
chest pain	Systemic diseases and various reactions at the administration site	Chest pain	Non-solicitation	November 8, 2021	November 18, 2021	November 22, 2021	5	10	No	Grade 3	hospitalization	Unlikely

3.4. Listing of adverse events after 30 days of exemption (SS)

Table 3-4 Adverse events list after 30 days of exemption (SS)

Subject number	Group	Age (years)	Sex	Nationality	Description of Adverse Events	Systematic organ classification	Preferred Term	Types of adverse events	Date of inoculation	Start date	End Date	Duration days	Days from vaccination	Is it within 30min	Severity	Treatment status	Correlation
No relevant data																	

3.5. Listing of adverse events of special interest (SS)

Table 3-5 List of adverse events of special concern (SS)

Subject No.	Group	Age (years)	Gender	Nationality	Adverse event description	System Organ Class	Preferred terms	Date of inoculation	Start date	End Date	Duration days	Days from vaccination	Outcome situation	Correlation
No relevant data														

3.6. List of serious adverse events (SS)

Table 3-6 List of serious adverse events (SS)

Description of Adverse Events	Systematic organ classification	Preferred Term	Date of inoculation	Start Date	End date	Duration Days	Days from inoculation	SAE Classification	Outcome situation	Correlation
Subject No.: 0501, Group: Inactivated Vaccine Group, Age (years): 41.7, Sex: Male, Nationality: Bangladesh										
Lumbar transverse process fracture, Back pain	Respiratory, thoracic and mediastinal diseases	Cough	November 3, 2021	December 8, 2021	December 11, 2021	4	35	Hospitalization	Symptoms disappeared	Not related
Lumbar Transverse Process Fracture, Back Pain	Musculoskeletal and connective tissue disorders	Back pain	November 3, 2021	December 8, 2021	December 11, 2021	4	35	Hospitalization	Symptoms dispared	Definitely irrelevant
Lumbar transverse process fracture, Back pain	All kinds of injuries, poisoning and complications of operation	Lumbar spine fracture	November 3, 2021	December 8, 2021	December 11, 2021	4	35	Hospitalization	Symptoms disappeared	Not related
Subject No.: 2572, Group: Recombinant Vaccine Group, Age (years): 38.7, Sex: Male, Nationality: Bangladesh										
Acute appendicitis	Infection and infectious diseases	Appendicitis	November 4, 2021	December 11, 2021	December 12, 2021	2	37	Hospitalization	Symptoms disappeared	Not related
Subject No.: 2805, Group: Recombinant Vaccine Group, Age (years): 41.8, Sex: Male, Nationality: India										
Sepsis due to pneumonia	Infection and infectious diseases	Pneumococcal sepsis	November 6, 2021	November 13, 2021	November 18, 2021	6	7	Hospitalization	Symptoms disappeared	Not related

Description of Adverse Events	Systematic organ classification	Preferred Term	Date of inoculation	Start Date	End date	Duration Days	Days from inoculation	SAE Classification	Outcome situation	Correlation
Subject No.: 2854, Group: Inactivated Vaccine Group, Age (years): 49.9, Sex: Male, Nationality: Pakistan										
Pneumonia, unspecified organism.	Infection and infectious diseases	Pneumonia infectious	November 6, 2021	November 20, 2021	November 24, 2021	5	14	Hospitalization	Symptoms disappeared	Not related
Pneumonia, unspecified organization.	Respiratory, thoracic and mediastinal disorders	Pleural effusion	November 6, 2021	November 20, 2021	November 24, 2021	5	14	Hospitalization	Symptoms dispared	Definitely irrelevant
Subject No.: 2990, Group: Inactivated Vaccine Group, Age (years): 29.9, Sex: Male, Nationality: Bangladesh										
Acute MI	Cardiac disorders	Acute myocardial infarction	November 8, 2021	November 18, 2021	November 22, 2021	5	10	Hospitalization	Symptoms dispared	Definitely irrelevant
Acute MI	Gastrointestinal system diseases	gastroesophageal reflux disease	November 8, 2021	November 18, 2021	November 22, 2021	5	10	Hospitalization	Symptoms disappeared	Not related

3.7. Listing of adverse events (based on all enrolled subjects) before immunization

Table 3-7 Listing of adverse events before exemption (based on all enrolled subjects)

Adverse event description	System Organ Class	Preferred terms	Date of inoculation	Start date	End Date	Duration days	Severity	Treatment status	Correlation
Subject No.: 0545, Group: Inactivated Vaccine Group, Age (years): 34, Sex: Male, Nationality: Bangladesh									
Stomach Pain	Gastrointestinal system diseases	Abdominal pain upper	November 4, 2021	November 2, 2021	November 5, 2021	4	Grade 2	Prescription Drug	May be irrelevant

Supplementary Notes

Supplementary Note 3. Trial protocol.

A Randomized, Controlled Phase I and Sequential Study to Evaluate the Safety and Immunogenicity following Immunization of GEN2-Recombinant COVID-19 vaccine (CHO cells) and Inactivated COVID-19 Vaccine (Vero cells) in Population Aged 18 Years and Above

Protocol Title:	A Randomized, Controlled Phase I and Sequential Study to Evaluate the Safety and Immunogenicity following Immunization of GEN2-Recombinant COVID-19 vaccine (CHO cells) and Inactivated COVID-19 Vaccine (Vero cells) in Population Aged 18 Years and Above
Product Name:	GEN2-Recombinant COVID-19 Vaccine (CHO cells)
Specification:	20ug/dose/0.5 ml
Protocol Number:	CNBG-REC-2021003
Version Date:	Sep 3, 2021
Version Number:	1.0

Sponsor (Seal): China National Biotec Group Co., Ltd (CNBG)
Sinopharm CNBG National Vaccine & Serum Institute
Beijing Insitute of Biological Products Co., Ltd
Lanzhou Institute of Biological Products Co., Ltd

Responsible Institute (seal):

Statistical Institute (Seal):

TABLE OF CONTENTS

TABLE OF CONTENTS 2

STATEMENT OF COMPLIANCE 5

INVESTIGATOR'S COMPLIANCE DECLARATION 6

List of abbreviations 7

Synopsis 9

1. Introduction 18

2. Background and Principle 18

2.1 Background of disease 18

2.2. Pathogenic background 19

2.3. Vaccine background 20

3. Study Objectives 23

3.1 Phase I Study 23

3.2 Sequential Study 24

4. Study Design 24

4.1. Trial Description 24

4.2. Procedures and Methodology 24

4.3. Study Endpoints 28

4.4 Trial Hypothesis 29

4.5 Safety Observation Indicators and Grading Criteria 30

4.6 Randomization and Blinding 35

5. Screening and Withdrawal of Subjects 36

5.1 Subject Population 36

6. Product Introduction 38

6.1 Description and Characteristics of the Investigational Products 38

6.2 Vaccine Packaging and Labeling 39

6.3 Vaccine Storage and Transportation 40

6.4 Administration and Immunization Schedule 41

7. Study Workflow and Visiting Method 42

7.1 Schedule of Visits 42

7.2 Study Procedure 42

7.3 Safety Observation and Follow-up 45

7.4 Laboratory Testing 46

7.5 COVID-19 Case Monitoring 51
7.6 Concomitant Medication 54
7.7 Criteria for Suspension or Early Termination 55
7.8 Protocol Violation and Deviation 55
7.9 Study Duration 56
7.10 Site Closure 56
8. Safety Definition, Reporting and Monitoring 56
8.1 General Precautions 56
8.2 Risk Prevention Measures Related to COVID-19 57
8.3 Handling and Reporting of Serious Adverse Events 58
8.4. Outcome of Serious Adverse Events 60
9. Completion of Clinical Trial 60
10. Statistical Considerations 60
10.1 Sample Size Considerations 60
10.2 Analysis Set 61
10.3 Statistical Method 61
10.4 Subgroup Analysis 63
10.5 Interim Analysis 63
10.6 Multiplicity 63
10.7 Handling of Missing Data 63
11. Ethical and Regulatory Matters 63
11.1. Ethics Committees and Regulatory Authorities 63
11.2. Responsibilities of the Sponsor 64
11.3. Responsibilities of the Investigator 64
11.4. Subject Information and Consent 65
11.5. Compensation to Subjects 66
11.6. Subject Confidentiality 66
11.7. Amendment to Subject Related Information 67
11.8. Direct Access to Source Documentation 67
12. Study Management 68
12.1. Case Report Form (eCRF) Handling 68
12.2. Source Data and Subject Files 69

12.3. Investigator Site File and Archiving 70
12.4. Monitoring, Quality Assurance and Inspection by Authorities 70
12.5. Changes to the Study Protocol 71
12.6. Study Report and Publication Policy 71
13. References 73

STATEMENT OF COMPLIANCE

This study will be performed in compliance with Good Clinical Practice (GCP), the Declaration of Helsinki (with amendments) and local legal and regulatory requirements.

Signature Page

The signature below constitutes the approval of this protocol and the attachments and provides the necessary assurances that this trial will be conducted according to all stipulations of the protocol, including all statements regarding confidentiality, and according to Good Clinical Practice (GCP), Declaration of Helsinki (with amendments) and the laws and regulations of the countries in which the study takes place.

<table>
<tr><td>Protocol name</td><td colspan="2">A Randomized, Controlled Phase I and Sequential Study to Evaluate the Safety and Immunogenicity following Immunization of GEN2-Recombinant COVID-19 vaccine (CHO cells) and Inactivated COVID-19 Vaccine (Vero cells) in Population Aged 18 Years and Above</td></tr>
<tr><td>Protocol number</td><td colspan="2">CNBG-REC-2021003</td></tr>
<tr><td>Version date</td><td colspan="2">Sep 3, 2021</td></tr>
<tr><td>Version number</td><td colspan="2">Version 1.0</td></tr>
<tr><td>Sponsor</td><td colspan="2">China National Biotec Group Co. Ltd. (CNBG)
Sinopharm CNBG National Vaccine & Serum Institute
Beijing Insitute of Biological Products Co., Ltd
Lanzhou Institute of Biological Products Co., Ltd</td></tr>
<tr><td>Sponsor Project leader</td><td>Name: Yang Yunkai
Company: China National Biotec Group Co., Ltd. (CNBG)
Address: No.B 2 Shuangqiao Road, Chaoyang District, Beijing, 100024 P.R. of China
Zip Code: 100024
Tel: 13601126881
E-mail: yangyunkai@sinopharm.com</td><td>Signature:

Date:</td></tr>
</table>

INVESTIGATOR'S COMPLIANCE DECLARATION

- I have read this protocol and agree to conduct the study as outlined herein, and as implemented by any future protocol amendment/update, according to the terms of the clinical trial contract, and in accordance with supplementary study: conduct procedures and/or guidance or documents of this study, complying with the obligations and requirements of clinical investigators and all other requirements listed in relevant national and international regulations including ICH GCP guidelines.
- I assume responsibility for the compliance of the site personnel reporting to me or assisting me with the study.
- I confirm that I am aware of my obligations towards relevant regulatory authorities, as it concerns my participation in this study as a researcher.
- I agree to disclose and provide information to the Sponsor on any potential conflict of interest I may have participating in this study as investigator.
- I declare that I will co-operate with the Sponsor personnel and/or representatives, and vendors managing or supporting the study, including CRO, timely and adequately to ensure timely study conduct and compliance with study documents and relevant regulations.
- I am fully familiar with correct method of using vaccine described in the protocol, and other information provided by sponsor, including but not limited to the following contents: current investigator's brochure (IB) or equivalent documents and relavant supplements.
- I am familiar with and will abide by the GCP, the Guiding Principles for Quality Management of Vaccine Clinical Trials (Tentative) and all existing regulatory requirements.

**Protocol Name**	A Randomized, Controlled Phase I and Sequential Study to Evaluate the Safety and Immunogenicity following Immunization of GEN2-Recombinant COVID-19 vaccine (CHO cells) and Inactivated COVID-19 Vaccine (Vero cells) in Population Aged 18 Years and Above
**Protocol Number**	CNBG-REC-2021003
**Version date**	Sep 3, 2021
**Version Number**	Version 1.0

Name of Investigator (please print) ______________________

Investigator Signature: ________________________________

Date: ____

CONFIDENTIAL

LIST OF ABBREVIATIONS

ACE2	Angiotensin converting enzyme 2
ADE	Antibody Dependence Enhancement
AE	Adverse Event
BP	Blood Pressure
CI	Confidence Interval
COA	Certificate of Analysis
CoV	Coronavirus
COVID-19	Coronavirus disease of 2019
DNA	Deoxyribonucleic acid
DSMB	Data and Safety Monitoring Board
ECRF	Electronic Case Report Form
EDC	Electronic Data Capture System
EAC	Endpoint Assessment Committee
FAS	Full Analysis Set
GCP	Good Clinical Practice
GMP	Good Manufacturing Practice
GMT	Geometric Mean Titer
GMI	Geometric mean increase
IB	Investigator's Brochure
ICF	Informed Content Form
IEC	Independent Ethics Committee
MERS	Middle East Response Syndrome
NMPA	National Medical Products Administration
MFAS	Modified Full Analysis Set
PPS	Per-Protocol Set
PCR	Polymerase Chain Reaction
RBD	Receptor Binding Domain
RNA	Ribonucleic acid
SAE	Serious Adverse Events
SARS	Severe Acute Respiratory Syndrome
SOP	Standard Operating Procedure
SS	Safety Set

CONFIDENTIAL

SUSAR	Suspected and Unexpected Serious Adverse Reactions
VED	Vaccine Enhanced Disease
WHO	World Health Organization

CONFIDENTIAL

Synopsis

**Study Title**	A Randomized, Controlled Phase I and Sequential Study to Evaluate the Safety and Immunogenicity following Immunization of GEN2-Recombinant COVID-19 vaccine (CHO cells) and Inactivated COVID-19 Vaccine (Vero cells) in Population Aged 18 Years and Above
**Products Characteristic**	This recombinant protein was expressed by recombinant Chinese hamster ovary (CHO) cells and designed based on the receptor-binding domain (RBD) of SARS-CoV-2 Spike (S) protein. Engineered cells were cultured, harvested and purified by a series of processes, and then added with aluminum hydroxide adjuvant.
**Indication**	This product is used for population aged 18 years and above. Vaccination with this vaccine can stimulate the body to produce immune response to SARS-CoV-2, which is used to prevent COVID-19.
**Study Design**	This is a randomized, double blinded and positive controlled design. Among the subjects who have been vaccinated with two doses of Inactivated COVID-19 vaccine (Vero cell), based on a step-wise approach, the subjects will receive one dose of recombinant COVID-19 vaccine sequentially at different shedules of 4-6 months, 7-9 months and >9 months after two doses of vaccination, and the subjects vaccinated at different schedules will be randomly assigned to different sequential immunization groups. At the same time, each sequential immunization group will be matched with a control group with the inactivated COVID-19 vaccine (vero cells) as the booster dose.<br>**Sample Size**: Total sample size is 1,848.<br>*Phase I clinical study (Phase I Study)*: The sample size for Phase I Study is 48 subjects, which will be assigned to age groups of 18-59 years and ≥60 years to be administered with recombinant vaccine at target dose, with 24 subjects in each group.<br>*Sequential clinical study (Sequential Study)*: The sample size for Sequential Study is 1800 subjects, which will be randomly assigned to recombinant vaccine sequential group and inactivated vaccine sequential group in a 1: 1 ratio, with 300 subjects in each sequential group.<br>**Immunization Schedule:**<br>*Phase I Study*: 18-59-year-old group and ≥60-year-old group will be vaccinated with 3 doses of recombinant COVID-19 vaccine (CHO cells) at target dose according to D0, D30 (+7), D60 (+7) immunization schedule, a safety assessment will be performed on the 8th day

	after the first dose in 18-59-year-old group, and only if the preliminary safety assessment results meet the expectation which is no SAEs occurred within 7 days after vaccination, could initiate the enrollment of ≥60-year-old group. Subsequent vaccinations can be carried out at the study site based on the work to be conducted as appropriate.<br>*Sequential Study*: If the preliminary safety assessment results of each age group in Phase I Study meet the expectation which is no SAEs occurred within 7 days after vaccination, the vaccination in the same age group in the Sequential Study can be initiated. The subjects who have been vaccinated with two doses of inactivated COVID-19 vaccine according to the immunization schedule of D0 and D21 (+7) days, and the interval between the two doses will be no longer than 8 weeks according to *Technical Guidelines for Inoculation of SARS-COV-2 Vaccine (First Edition)* issued by National Health Commission in China, will receive sequential vaccination of one dose of recombinant COVID-19 vaccine (CHO cells) or inactivated COVID-19 Vaccine (Vero cells) at different schedules of 4-6 months, 7-9 months and >9 months.<br>**Route of Administration**: lateral deltoid muscle of upper arm, intramuscular injection<br>**Safety Observation:**<br>The subjects will be observed at the study site for 30 minutes after each dose of vaccination, and the local and systemic reactions of the subjects within 0-7 days will be actively followed up and recorded on the diary card. The safety follow-up will be performed once per week within 8-30 days after vaccination to collect AEs and document them in the follow-up system.<br>Serious adverse events (SAEs) will be collected from the first dose of vaccination and up to 6 months following the full course of immunization.<br>**Immunogenicity Observation:**<br>*Phase I Study*: Blood samples of all the subjects will be collected before the vaccination of the 1st dose and 3rd dose, 15 days, 30 days, 6 months and 12 months after 3rd dose for testing of neutralizing antibodies and anti-protein specific IgG antibodies.<br>*Sequential Study*: Blood samples of all the subjects will be collected before the vaccination of sequential dose and 15 days, 30 days, 3 months, 6 months, 9 months and 12 months after sequential vaccination for testing of neutralizing antibodies and anti-protein specific IgG antibodies.<br>Collect 12.5ml blood samples of all the subjects and separate serum. Neutralizing

antibody will be determined by microdose cytopathogenic efficiency (CPE) assay and specific IgG antibody will be tested by enzyme-linked immunosorbent assay (ELISA).

Time points of blood sample collection are described in Table 1.

Table 1-1 Sample Size and Procedures of Phase I Study

Age Group	Dosage	Immunization Schedule	Sample Size	Safety	Blood Collections for Immunogenicity Testing
18~59 years	20ug/0.5mL	D0,30,60	24	1.Collect AEs and SAEs within 30min(onsite observation)、0-7 days（diary cards） and 8-30 days （safety visits）;<br>2. collect SAEs up to 6 months after full course of immunization	①Before 1st dose;<br>②Before 3rd dose;<br>③15 days after 3rd dose;<br>④30 days after 3rd dose;<br>⑤6 months after 3rd dose<br>⑥12 months after 3rd dose.
≥60 years	20ug/0.5mL		24		
Total			48		

Table 1-2 Sample Size and Procedures of Sequential Study

Sequential Vaccination Time of Recombinant Vaccine	Booster Immunization	Sequential Vaccine Group	Sample Size	Safety	Blood Collection Time Point
4-6 months after 2 doses	91-180 days (+14)	Recombinant Vaccine	300	Collect AEs within 30min(onsite observation), 0-7 days（diary cards） and 8-30 days （safety visits）after immunization with sequential booster dose, and SAEs up to 6 months following immunization with the booster dose.	①Before sequential dose;<br>②15 days after sequential dose;<br>③30 days after sequential dose;<br>④3 months after sequential dose;<br>⑤6 months after sequential dose;<br>⑥9 months after sequential dose;<br>⑦12 months after sequential dose.
		Inactivated Vaccine	300		
7-9 months after 2 doses	181-270 days (+14)	Recombinant Vaccine	300		
		Inactivated Vaccine	300		
> 9 months after 2 doses	≥ 271 days	Recombinant Vaccine	300		
		Inactivated vaccine	300		
**Total sample size**			**1800**		

Note: Blood collection window at 15 days, 30 days is +10 days, blood collection window at 3M, 6M, 9M, 12M is +30 days.

**Study Objective**	**Phase I Study/Sequential combined clinical trial**<br>***Phase I Study***<br>**Primary Objectives:**<br>To evaluate the safety of recombination GEN2-COVID-19 vaccine (CHO cells) 30 days after 1 dose of vaccination in populations aged 18 years and above<br>**Secondary Objectives:**<br>To evaluate the immunogenicty of recombination COVID-19 vaccine (CHO cells) 15 days after 3 doses of vaccination in populations aged 18 years and above<br>To evaluate the immunogenicty of recombination COVID-19 vaccine (CHO cells) 30 days after 3 doses of vaccination in populations aged 18 years and above<br>To evaluate the immune persistence of recombination COVID-19 vaccine (CHO cells) after 3 doses of vaccination in populations aged 18 years and above<br>**Exploratory Objective:**<br>To evaluate the vaccine efficacy of recombinant COVID-19 vaccine (CHO cells) against COVID-19, especially severe cases, and deaths 15 days after full course immunization.<br>To evaluate the vaccine efficacy of recombinant COVID-19 vaccine (CHO cells) against different variants 15 days after full course immunization.<br><br>***Sequential Study***<br>**Primary Objectives:**<br>To compare the immunogenicity between recombinant COVID-19 vaccine (CHO cells) and inactivated COVID-19 vaccine (Vero cells) 15 days after sequential vaccination of one dose in populations aged 18 years and above.<br>To compare the immunogenicity between recombinant COVID-19 vaccine (CHO cells) and inactivated COVID-19 vaccine (Vero cells) 30 days after sequential vaccination of one dose in populations aged 18 years and above.<br>**Secondary Objectives:**<br>To evaluate the safety of recombinant COVID-19 vaccine (CHO cells) and inactivated COVID-19 vaccine (Vero cells) 0-30 days after sequential vaccination of one dose in populations aged 18 years and above.<br>To evaluate the optimal immunization schedule of sequential vaccination of recombinant COVID-19 vaccine (CHO cells) in populations aged 18 years and above.

	To evaluate the immune persistence of sequential vaccination of recombinant COVID-19 vaccine (CHO cells) and inactivated COVID-19 vaccine (Vero cells) in populations aged 18 years and above.<br>**Exploratory Objective:**<br>To evaluate the vaccine efficacy of recombinant COVID-19 vaccine (CHO cells) against COVID-19, especially severe cases, and deaths 15 days after sequential vaccination at different schedules.<br>To evaluate the vaccine efficacy of recombinant COVID-19 vaccine (CHO cells) against different variants 15 days after sequential vaccination at different schedules.
**Investigational Vaccine**	Investigational vaccine: Recombinant COVID-19 Vaccine (CHO cells)<br>Manufacturer: Sinopharm CNBG National Vaccine & Serum Institute<br>Lanzhou Institute of Biological Products Co., Ltd<br>Specifications: 0.5 ml/vial. The dose for human use is 0.5 mL per time, which contains 20ug of viral protein antigen.<br>Storage conditions: 2-8 ℃<br>Batch number: 20210823 Expiry date: August 26, 2023<br><br>Control vaccine: Inactivated SARS-CoV-2 Vaccine (Vero cell)<br>Manufacturer: Beijing Institute of Biological Products Co., Ltd.<br>Specifications: 0.5 ml/vial. The dose for human use is 0.5 mL per time, which contains 6.5U of antigen.<br>Storage conditions: 2-8 ℃<br>Batch number: 2021071931 Expiry date: July 22, 2023
**Inclusion Criteria**	- Age range: populations aged 18 years and above.<br>- Judged by the investigator that the health condition is well after inquiry, medical history, and physical examination.<br>- Hasn't been vaccinated with any COVID-19 vaccines before (*Phase I Study*)<br>- Vaccinated with 2 doses of Sinopharm-CNBG inactivated COVID-19 vaccine *(Sequential Study*).<br>- Female subjects who are not nursing or pregnant at the time of enrollment (negative urine pregnancy test) and have no family plans within the first 6 months after enrollment.

CONFIDENTIAL

	Effective contraceptive measures have been taken within 2 weeks before inclusion.<br>- During the whole follow-up period of the study, be able and willing to complete the whole prescribed study plan;<br>- With self ability to understand the study procedures, the informed consent & voluntarily sign an informed consent form and is able to comply with the requirements of the clinical study protocol.
**Excluion Criteria**	- Confirmed cases, suspected cases or asymptomatic cases of COVID-19;<br>- With a history of SARS and MERS infection (self-report, on-site inquiry);<br>- Has been vaccinated with any other vaccine other than one or more doses of Sinopharm-CNBG inactivated COVID-19 vaccine;<br>- Axillary temperature ≥ 37.3 ℃ (forehead temperature ≥ 37.8 ℃);<br>- Previous severe allergic reactions to vaccination (such as acute allergic reactions, urticaria, dyspnea, angioneurotic edema or abdominal pain)<br>- Allergy to known components of recombinant COVID-19 vaccine (e.g., aluminum and histidine);<br>- Severe respiratory diseases, severe liver and kidney diseases, hypertension (systolic blood pressure ≥ 150 mmHg, diastolic blood pressure ≥ 90 mmHg), diabetic complications, malignant tumors, various acute diseases or acute attacks of chronic diseases;<br>- Has been diagnosed with congenital or acquired immunodeficiency, HIV infection, lymphoma, leukemia or other autoimmune diseases;<br>- Has a history of convulsions, epilepsy, encephalopathy, long-term alcohol and drug abuse, history of thyroidectomy, history of infectious diseases, mental illness or family history (direct);<br>- With congenital malformation or developmental disorder, genetic defect, severe malnutrition, etc.;<br>- Has a history of coagulation dysfunction (e.g., coagulation factors deficiency and coagulation diseases);<br>- Asplenia or splenectomy, functional asplenia caused by any situation;<br>- Receving anti-TB (tuberculosis) therapy;<br>- Received immunoenhancement or inhibitor therapy within 3 months (continuous oral

	or IV administration for more than 14 days);<br>- Received other vaccines within 14 days before vaccination;<br>- Received blood products within 3 months before vaccination;<br>- Received other investigational drugs within 6 months before vaccination;<br>- Plans to move before the end of the study or leave the local area for a long time during the scheduled study visits;<br>- Other circumstances judged by investigators that are not suitable for this clinical trial**.**
**Subject's early withdrawal**	**Early withdrawal means that the subject fails to complete the vaccination and blood collection procedures in accordance with clinical study protocol, and investigator decides whether to continue the follow-up related research according to the situation. When any of the following conditions occur, the subject will withdraw from the clinical trial in advance.**<br>- The subject requests to withdraw from the clinical trial;<br>- Intolerable adverse events, whether related to the investigational drug or not;<br>- The health status of the subjects does not allow them to continue to participate in this trial;<br>- The subjects are vaccinated with other clinical investigational vaccines during the study period;<br>- Any other reason that the investigator considers.
**Criteria for suspension and early termination**	**In case of any of the following circumstances, the trial shall be suspended, and the investigator, the sponsor and the Ethics Committee shall jointly hold a meeting to decide whether to terminate the clinical trial early:**<br>- The number of subjects with adverse reactions of severity Grade 3 or above exceeds 15% of the subjects vaccinated in this subgroup;<br>- One case of Grade 4 adverse reaction related to vaccination or SUSAR has occured.<br>**In case of any of the following circumstances, the clinical trial shall be terminated early:**<br>- Sponsor has found that the vaccine has potential safety hazards or the trial has quality related problems, and requiring the complete termination of the study.<br>- The Ethics Committee requests the termination of the trial because of the violation of ethics in the trial.

	– Request by the regulatory authority to terminate the trial.
**Trial Endpoint**	**Phase I Study**<br>**Safety endpoint:**<br>The incidence and serverity of any adverse reactions/events within 30 minutes after vaccination;<br>The incidence and serverity of solicited adverse reactions/events within 0-7 days after vaccination;<br>The incidence and severity of unsolicited adverse reactions/events within 8-30 days after vaccination;<br>The incidence of SAE and AESI observed after vaccination and up to 6 months after full course of immunization.<br>**Immunogenicity endpoint:**<br>**Primary endpoint:**<br>GMT and 4-fold rise of anti-SARS-CoV-2 neutralizing antibody 15 days after 3rd dose;<br>GMT and 4-fold rise of anti- SARS-CoV-2 neutralizing antibody 30 days after 3rd dose.<br>**Secondary endpoint:**<br>GMT of anti-SARS-CoV-2 IgG antibody, 4-fold rise, GMI, and proportions of neutralizing antibody titered ⩾ 1: 16, ⩾ 1: 32 and ⩾ 1: 64 before vaccination and 30 days after 3rd dose.<br>**Immune persistence endpoint:**<br>Anti-SARS-CoV-2 neutralizing antibody and GMT of IgG antibody 6 months and 12 months after full course of immunization, and the proportions of neutralizing antibody titered ⩾ 1: 16, ⩾ 1: 32 and ⩾ 1: 64.<br>**Exploratory endpoint:**<br>To evaluate the vaccine efficacy of recombinant COVID-19 vaccine (CHO cells) against COVID-19, especially severe cases, and deaths 15 days after full course immunization in populations aged 18 years and above.<br>To evaluate the vaccine efficacy of recombinant COVID-19 vaccine (CHO cells) against different variants 15 days after full course immunization in populations aged 18 years and above.

	**Sequential Study**<br>**Safety endpoint:**<br>The incidence and serverity of any adverse reactions/events within 30 minutes after vaccination;<br>The incidence and serverity of solicited adverse reactions/events within 0-7 days after vaccination;<br>The incidence and severity of unsolicited adverse reactions/events within 8-30 days after vaccination;<br>The incidence of SAE and AESI observed after vaccination and up to 6 months after full course of immunization.<br>**Immunogenicity endpoint:**<br>**Primary endpoint:**<br>GMT and the 4-fold rise of anti-SARS-CoV-2 neutralizing antibody 15 days after sequential vaccination of one booster dose;<br>GMT and 4-fold rise of anti-SARS-CoV-2 neutralizing antibody 30 days after sequential vaccination of one booster dose.<br>**Secondary endpoint:**<br>GMT of anti-SARS-CoV-2 IgG antibody, 4-fold rise, GMI, and proportions of neutralizing antibody titered ⩾ 1: 16, ⩾ 1: 32 and ⩾ 1: 64 before vaccination and 30 days after vaccination of the sequential booster dose.<br>**Immune persistence endpoint:**<br>Anti-SARS-CoV-2 neutralizing antibody and GMT of IgG antibody 3 months, 6 months, 9 months and 12 months after full course of immunization, and the proportions of neutralizing antibody titered ⩾ 1: 16, ⩾ 1: 32 and ⩾ 1: 64.<br>**Exploratory endpoint:**<br>To evaluate the vaccine efficacy of recombinant COVID-19 vaccine (CHO cells) against COVID-19, especially severe cases, and deaths 15 days after sequential vaccination at different schedules in populations aged 18 years and above.<br>To evaluate the vaccine efficacy of recombinant COVID-19 vaccine (CHO cells) against different variants 15 days after sequential vaccination at different schedules in populations aged 18 years and above.

A Randomized, Controlled Phase I and Sequential Study to evaluate the Safety and Immunogenicity following Immunization of Recombinant COVID-19 vaccine (CHO cells) and Inactivated COVID-19 Vaccine (Vero cells) in Population Aged 18 Years and Above

1. INTRODUCTION

The recombinant COVID-19 vaccine (CHO cell) developed by Sinopharm CNBG National Vaccine & Serum Institute (NVSI), Beijing Institute of Biological Products Co., Ltd. (BIBP) and Lanzhou Institute of Biological Products Co., Ltd. (LIBP) affiliated to China National Biotec Group Co., Ltd (CNBG) is used to prevent the diseases caused by SARS-CoV-2. After being reviewed by National Medical Products Administration (NMPA) in accordance with *Drug Administration Law of the People's Republic of China*, *Vaccine Administration Law of the People's Republic of China* and *Provisions for Drug Registration*, it has been approved for Phase I/II clinical study in China, and the data obtained from the Phase I/II clinical trial in population aged 18 years and above completed showed good safety profile.

In this project, a randomized, blinded, positive control method will be used to evaluate the safety and immunogenicity of sequential vaccination of recombinant COVID-19 vaccine (CHO cells) in healthy population aged 18 years and above. This study was approved by the Ministry of Health and Prevention of UAE.

2. BACKGROUND AND PRINCIPLE

2.1 Background of disease

According to WHO data, as of July 23, 2021, there were 194,595,271 confirmed cases and 4,165,733 deaths due to COVID-19, with a mortality rate of 2.1%.[26] The infection sources of COVID-19 are mainly SARS-CoV-2 infected patients and asymptomatic infected persons, but it is found that patients in incubation period are also contagious, and they are highly contagious within 5 days after onset. SARS-CoV-2 is mainly transmitted through respiratory droplets and close contact, which may be transmitted by aerosol. Contact with virus contaminated articles can also cause infection. Because SARS-CoV-2 can be isolated from patients' feces and urine,

CONFIDENTIAL

environmental pollution caused by feces and urine can also cause aerosol or contact transmission. People are generally susceptible, and they can get infection in all ages. People can get certain immunity after infection or vaccination with COVID-19 vaccine, but the duration is not clear. Based on the current epidemiological investigation, the incubation period of virus is generally 1 ~ 14 days, mostly 3 ~ 7 days, and the incubation period of very few cases can reach 24 days.

The main clinical symtoms of COVID-19 are fever, dry cough and fatigue, and the first symptoms of some patients are loss of smell and taste. A few patients are accompanied by nasal congestion, runny nose, sore throat, muscle pain and diarrhea. According to clinical symptoms, it can be divided into four categories: mild, moderate, severe and critical. Severe patients often have dyspnea and/or hypoxemia one week after onset, and severe patients can rapidly progress to acute respiratory distress syndrome, septic shock, metabolic acidosis, coagulation dysfunction and multiple organ failure. Very few patients may also have central nervous system involvement and acrovascular necrosis. It is worth noting that severe and critical patients may have moderate to low fever or even no obvious fever during the course of disease. Mild patients may show low fever, slight fatigue, smell and taste disorders, etc., without pneumonia. A few patients have no obvious clinical symptoms after being infected with SARS-CoV-2. According to the current cases, most patients have a good prognosis, while a few patients are in critical condition. The prognosis of the elderly and those with chronic basic diseases is poor. The clinical process of pregnant women with COVID-19 is similar to that of patients of the same age. Children's cases have relatively mild symptoms.

2.2. Pathogenic background

SARS-CoV-2 belongs to β coronavirus. Under cryoelectron microscope, it is approximately spherical particles with variable diameter, mainly around 100nm. SARS-CovV-2 contains dense virus matrix, surrounded by lipid bilayer, and its outer surface protrudes. It is an enveloped virus, which is composed of non-segmental positive-sense single-stranded RNA and protein. It belongs to Orthocoronavirinae and is widely distributed in humans and other mammals.

The genome of SARS-CoV-2 strain is 29.9 kb long and encodes 9860 amino acids. SARS-CoV-2 has four main structural proteins, known as spike protein (S), membrane protein (M), envelope protein (E) and nucleocapsid protein (N), as well as several helper proteins. S, M and E proteins constitute the viral envelope and are the main surface antigens of virus-induced immune response. S protein is a transmembrane glycoprotein with a molecular weight of about 150kDa, which is divided into two subunits, namely S1 and S2, by Furin protease of host cells. S1 contains one RBD, which is responsible for determining the cell targeting between host cells and virus

CONFIDENTIAL

receptor binding domains; As a membrane fusion subunit, S2 can mediate virus to fuse with host cells. M protein plays an important role in determining the shape of viral envelope. It can bind with other structural proteins, and binding with N protein helps to stabilize nucleocapsid and promote the assembly of virus. E protein plays an important role in the process of virus production and maturation. N protein is located in the core of virus and combines with viral RNA to form nucleocapsid. N protein has a highly conserved sequence, and it participates in virus replication and infection of host cells.

The binding of spike protein (S protein) with angiotensin converting enzyme 2 (ACE2) protein is essential for SARS-CoV-2 infecting human cells. After fusion, transmembrane protein serine 2 (TMPRSS2), which exists on the surface of host cells, scavenges ACE2 and activates S protein. Activation of the S protein causes a conformational change and allows the virus to enter the host cell. The affinity of S protein of SARS-CoV-2 with ACE2 is 10 to 20 times that of SARS virus, which may be the reason of strong transmission of COVID-19. ACE2 expressed in respiratory epithelial cells and type II alveolar cells of lung is the receptor of SARS-CoV-2 entering host cells. Besides lung, ACE2 is also distributed in intestinal epithelial cells of ileum and colon, vascular smooth muscle cells, proximal tubular epithelial cells of kidney, urinary tract epithelial cells of bladder and testis. Therefore, COVID-19 patients will not only have respiratory diseases, but also have heart, kidney, and digestive system diseases. ACE2 expression in vascular intima can damage endothelial cells and lead to the risk of thrombotic events. In addition, the immune system releases a large number of cytokines to viral infection sites or secondary infection, which can induce cytokine storm and septicemia symptoms.

In addition, coronavirus is sensitive to ultraviolet rays and heat. For 30 minutes at 56 ℃, ether, 75% ethanol, chlorine-containing disinfectant, peracetic acid and chloroform can effectively inactivate the virus, but chlorhexidine cannot effectively inactivate the virus.

2.3. Vaccine background

As of 26 March 2021, according to the statistics from World Health Organization (WHO), there are 267 COVID-19 vaccine projects in active research and development around the world, with diversified technical routes, mainly including inactivated vaccines, recombinant protein vaccines, viral vector vaccines and nucleic acid vaccines. There are 83 candidate vaccines in clinical stage, including 27 recombinant protein vaccines, among which NVX-CoV2373 developed by Novavax is in Phase III stage, recombinant protein vaccine developed by Zhifei Bio has been authorized for emergency use in China, and others are in Phase I/II study. And four inactivated vaccines, four recombinant viral vector vaccines and four nucleic acid vaccines are in

CONFIDENTIAL

Phase III clinical trial.

2.3.1 Inactivated vaccine

Inactivated vaccine is made from virus that was grown in cell culture and lost its pathogenicity by physical or chemical methods, but only retains its antigenicity. As a traditional vaccine preparation method, the process is mature. At present, there are 22 inactivated vaccines under research around the world, of which 11 are in clinical stage [26]. The inactivated vaccines of Sinopharm CNBG Wuhan Institute of Biological Products and Beijing Institute of Biological Products have carried out Phase III clinical study in UAE, and have been approved for conditional marketing in 8 countries including UAE and China, and authorized emergency use in 86 countries. The published clinical results showed good safety and efficacy. The seroconversion rate of antibodies in two-dose immunization schedule is 100%, and the vaccine efficacy is 79%. Phase III clinical results of inactivated vaccine CoronaVac of Sinovac Research & Development Co., Ltd. in Brazil, Turkey and Indonesia showed that the vaccine efficacy is higher than 50% efficacy threshold set by the World Health Organization, and it has been approved for conditional marketing in China and authorized for emergency use in many countries and regions.

2.3.2 Recombinant Protein Vaccine

Compared with other types of vaccines, recombinant protein vaccines are one of the key directions of research and development in novel vaccines due to its significant advantages in safety and low cost. At present, 96 COVID-19 recombinant protein vaccine candidates are under development, and 27 of them are at clinical evaluation stage[26].

The recombinant nanoparticle vaccine NVX-COV2373 developed by NOVAVAX consists of full-length SARS-CoV-2 spike glycoprotein trimer and MATRIX-M^{TM} adjuvant. The latest published phase III clinical interim results from UK showed that among 15,000 volunteers aged 18-84 years (27% volunteers are over 65 years old), the overall efficacy was 89.3% (56 cases in the placebo group and 6 cases in the vaccine group), and the efficacy against B.1.1.7 variant was 85.6%. According to the clinical data from Phase IIB in South Africa, among the 4,400 volunteers (6% were HIV positive), the efficacy of NVX-COV2373 in HIV negative population was 60% (29 cases in placebo group and 15 cases in vaccine group), and the efficacy of 49.4% if HIV positive population was included. Among COVID-19 infections, 90% cases were 501Y.V2 variants[32].

In the research pipeline of China, Zifei’s recombinant protein vaccine (CHO cell) contains of SARS-COV-2 S protein receptor binding domain (RBD) unique dimer as antigen, adjuvanted by traditional adjuvant, and has been authorized for emergence use in China. The published clinical phase II data showed that among 900 volunteers, the seroconversion rate of neutralizing antibody

CONFIDENTIAL

was 97% 14 days after full course immunization and the titer of neutralizing antibody (GMT) was 102.5 (CPE test method), which was twice as high as the antibody in the convalescent serum. In terms of safety, no serious adverse events related to vaccines were observed[33]. In addition, Clover Biopharmaceuticals' recombinant protein vaccine is also in Phase II/III clinical trial.

2.3.3 Recombinant Viral Vector Vaccines

Viral vector vaccine uses replicative or non-replicative viruses as vectors, recombines specific antigen gene fragments into the vector genome, and delivers them into host cells, and induces expression and induces corresponding immune response. Currently, there are 42 recombinant viral vector vaccines in clinical stage, of which 13 are in the process of Phase III clinical trial[26].

AZD1222 (CHADOX1 NCOV-19), jointly developed by Oxford University and AstraZeneca, uses replication-deficient chimpanzee adenovirus CHADOX1 as vector to expresses SARS-CoV-2 S protein, and has been authorized for emergency use in UK, Brazil and other countries and regions. Recently, the European Union approved the conditional marketing of the vaccine for immunization in population aged 18 years and above, and the efficacy was evaluated as 59.5%. Previously, the Phase III clinical results published by AstraZeneca from the UK and Brazil trials showed that the average vaccine efficacy was 70% (vaccine efficacy of 90% with 1.5 doses, N=2741; vaccine efficacy of 62% with full doses, N=11636)[35].

AD5-NCOV, jointly developed by Academy of Military Sciences and CanSino, uses replication-defective human adenovirus type 5 as vector to expresses SARS-CoV-2 S protein, and has been approved for conditional marketing in China. The published phase II clinical data showed that 28 days after single dose inoculation, the titers of RBD specific antibody in high dose group (1×10^{11}) and low dose group (5×10^{10}) were 656.5 and 571.0, respectively. The titer of neutralizing antibody was 19.5 and 18.3 respectively, and the seroconversion rate of neutralizing antibody was 59% and 47% respectively[36].

2.3.4 NUCLEIC ACID VACCINE

Nucleic acid vaccines introduce DNA or mRNA encoding specific antigens into human cells, express corresponding antigen proteins, and induce immune response. Currently, there are 60 nucleic acid vaccines in active research and development, and 21 vaccines in clinical stage, including 11 mRNA vaccines and 10 DNA vaccines.

mRNA-1273 developed by MODERNA, uses liposome nanoparticles (LNP) delivery system, and encodes SARS-CoV-2 S-2P antigen (composed of SARS-CoV-2 glycoprotein and complete S1-S2 cleavage site). mRNA-1273 has been granted emergency use authorization by FDA in mid-

CONFIDENTIAL

December 2020. The results of a Phase III clinical trial showed that among more than 30,000 subjects who received vaccine or placebo randomly, the vaccine efficacy was 94.1% (185 cases in the placebo group versus 11 cases in the vaccine group), and there was zero case of severe COVID-19 in the vaccine group[37].

mRNA vaccines BNT162B1 and BNT162B2 jointly developed by BIONTECH, Pfizer and Fosun Pharma uses LNP delivery system and encode SARS-CoV-2 S protein RBD trimer and full-length S protein respectively. BNT162B2 has been authorized for emergency use in the United States, the European Union and other regions, and is the first COVID-19 vaccine authorized by WHO for emergency use listing. The previously published Phase III clinical trial data showed that among 44,000 subjects, vaccine efficacy after two doses of vaccine was 95% (162 cases in the placebo group and 8 cases in the vaccine group)[38].

3. STUDY OBJECTIVE

To evaluate the safety and immunogenicity of sequential vaccination of recombinant COVID-19 vaccine (CHO cells) developed by Sinopharm CNBG National Vaccine & Serum Institute in healthy population aged 18 years and above.

3.1 Phase I Study

Primary Objectives:

To evaluate the safety of recombination COVID-19 vaccine (CHO cells) 30 days after each dose of vaccination in populations aged 18 years and above

Secondary Objectives:

To evaluate the immunogenicty of recombination COVID-19 vaccine (CHO cells) 15 days after 3 doses of vaccination in populations aged 18 years and above

To evaluate the immunogenicty of recombination COVID-19 vaccine (CHO cells) 30 days after 3 doses of vaccination in populations aged 18 years and above

To evaluate the immune persistence of recombination COVID-19 vaccine (CHO cells) after 3 doses of vaccination in populations aged aged 18 years and above

Exploratory Objectives:

To evaluate the vaccine efficacy of recombinant COVID-19 vaccine (CHO cells) against COVID-19, especially severe cases, and deaths 15 days after full course immunization in populations aged 18 years and above.

To evaluate the vaccine efficacy of recombinant COVID-19 vaccine (CHO cells) against different variants 15 days after full course immunization in populations aged 18 years and above.

CONFIDENTIAL

3.2 Sequential Study

Primary Objectives:

To compare the immunogenicity between recombinant COVID-19 vaccine (CHO cells) and inactivated COVID-19 vaccine (Vero cells) 15 days after sequential vaccination of one dose in populations aged 18 years and above.

To compare the immunogenicity between recombinant COVID-19 vaccine (CHO cells) and inactivated COVID-19 vaccine (Vero cells) 30 days after sequential vaccination of one dose in populations aged 18 years and above.

Secondary Objectives:

To evaluate the safety of recombinant COVID-19 vaccine (CHO cells) and inactivated COVID-19 vaccine (Vero cells) 0-30 days after sequential vaccination of one dose in populations aged 18 years and above.

To evaluate the optimal immunization schedule of sequential vaccination of recombinant COVID-19 vaccine (CHO cells) in populations aged 18 years and above.

To evaluate the immune persistence of sequential vaccination of recombinant COVID-19 vaccine (CHO cells) and inactivated COVID-19 vaccine (Vero cells) in populations aged 18 years and above.

Exploratory Objectives:

To evaluate the vaccine efficacy of recombinant COVID-19 vaccine (CHO cells) against COVID-19, especially severe cases, and deaths 15 days after sequential vaccination at different schedules in populations aged 18 years and above.

To evaluate the vaccine efficacy of recombinant COVID-19 vaccine (CHO cells) against different variants 15 days after sequential vaccination at different schedules in populations aged 18 years and above.

CONFIDENTIAL

4. STUDY DESIGN

4.1. Trial Description

This clinical trial is a randomized, double blinded and positive controlled design.

4.2. Procedures and Methodology

Sample size:

The total sample size is 1,848.

Phase I clinical study (*Phase I Study*): The sample size for Phase I Study is 48 subjects,

which will be assigned to age groups of 18-59 years and ≥60 years to be administered with recombinant vaccine at target dose, with 24 subjects in each group.

Sequential clinical study (*Sequential Study*): The sample size for Sequential Study is 1800 subjects, which will be randomly assigned to recombinant vaccine sequential group and inactivated vaccine sequential group in a 1: 1 ratio, with 300 subjects in each sequential group.

Immunization Schedule:

Phase I Study: 18-59-year-old group and ≥60-year-old group will be vaccinated with 3 doses of recombinant COVID-19 vaccine (CHO cells) at target dose according to D0, D30 (+7), D60 (+7) immunization schedule, a safety assessment will be performed on the 8th day after the first dose in 18-59-year-old group, and only if the preliminary safety assessment results meet the expectation which is no SAEs occurred within 7 days after vaccination, could initiate the enrollment of ≥60-year-old group. Subsequent vaccinations can be carried out at the study site based on the work to be conducted as appropriate.

Sequential Study: If the preliminary safety assessment results of each age group in Phase I Study meet the expectation which is no SAEs occurred within 7 days after vaccination, the vaccination in the same age group in the Sequential Study can be initiated. The subjects who have been vaccinated with two doses of inactivated COVID-19 vaccine according to the immunization schedule of D0 and D21 (+7) days, and the interval between the two doses will be no longer than 8 weeks according to Technical Guidelines for Inoculation of SARS-COV-2 Vaccine (First Edition) issued by National Health Commission in China, will receive sequential vaccination of one dose of recombinant COVID-19 vaccine (CHO cells) or inactivated COVID-19 Vaccine (Vero cells) at different schedules of 4-6 months, 7-9 months and >9 months.

Route of Administration: lateral deltoid muscle of upper arm, intramuscular injection

Safety Observation:

The subjects will be observed at the study site for 30 minutes after each dose of vaccination, and the local and systemic reactions of the subjects within 0-7 days will be actively followed up and recorded on the diary card. Perform safety follow-up once per week within 8-30 days after vaccination to collect AEs and document in the follow-up system.

Serious adverse events (SAEs) will be collected from the first dose of vaccination and up to 6 months following the full course of immunization.

Immunogenicity Observation:

Phase I Study: Blood samples of all the subjects will be collected before the vaccination of

CONFIDENTIAL

the 1st dose, before 3rd dose and 15 days, 30 days, 6 months and 12 months after 3rd dose for testing of neutralizing antibodies and anti-protein specific IgG antibodies.

Sequential Study: Blood samples of all the subjects will be collected before the vaccination of sequential dose and 15 days, 30 days, 3 months, 6 months, 9 months and 12 months after sequential vaccination for testing of neutralizing antibodies and anti-protein specific IgG antibodies.

Collect 12.5 ml blood samples of all the subjects and separate serum. Nentralizing antibody will be determined by microdose cytopathogenic efficiency (CPE) assay and specific IgG antibody will be tested by enzyme-linked immunosorbent assay (ELISA).

Time points of blood sample collection is described in Table 1.

Table 1-1 Sample Size and Procedures of Phase I Study

**Age Group**	**Dosage**	**Immunization Schedule**	**Sample Size**	**Safety**	**Blood Collections for Immunogenicity Testing**
18~59 years	20ug/0.5mL	D0,30,60	24	1.Collect AEs and SAEs within 30min (onsite observation)，0-7 days（diary cards） and 8-30 days （safety visits） after each dose;<br>2. collect SAEs up to 6 months after full course of immunization	①Before 1st dose;<br>②Before 3rd dose;<br>③15 days after 3rd dose;<br>④30 days after 3rd dose;<br>⑤6 months after 3rd dose<br>⑥12 months after 3rd dose.
≥60 years	20ug/0.5mL		24		
**Total**			48		

Table 1-2 Sample Size and Procedures of Sequential Study

**Sequential Vaccination Time of Recombinant Vaccine**	**Booster Immunization**	**Sequential Vaccine Group**	**Sample Size**	**Safety**	**Blood Collection Time Point**
4-6 months after 2 doses	91-180 days (+14)	Recombinant Vaccine	300	Collect AEs within 30min(onsite observation), 0-7 days（diary cards） and 8-30 days （safety visits） after immunization with sequential	①Before booster dose;<br>②15 days after sequential dose;<br>③30 days after sequential dose;<br>④3 months after sequential dose;
		Inactivated Vaccine	300		
7-9 months after 2 doses	181-270 days (+14)	Recombinant Vaccine	300		

		Inactivated Vaccine	300	booster dose, and SAEs up to 6 months following immunization with the booster dose.	⑤6 months after sequential dose<br>⑥9 months after sequential dose;<br>⑦12 months after sequential dose.
> 9 months after 2 doses	⩾ 271 days	Recombinant Vaccine	300		
		Inactivated vaccine	300		
Total sample size			**1800**		

Note: Blood collection window at 15 days, 30 days is +10 days, blood collection window at 3M, 6M, 9M, 12M is +30 days.

CONFIDENTIAL

4.3. Study Endpoint

4.3.1 PHASE I STUDY

Safety endpoint:

The incidence and serverity of any adverse reactions/events within 30 minutes after vaccination;

The incidence and serverity of solicited adverse reactions/events within 0-7 days after vaccination;

The incidence and severity of unsolicited adverse reactions/events within 8-30 days after vaccination;

The incidence of SAE and AESI observed after vaccination and up to 6 months after full course of immunization.

Immunogenicity endpoint:

Primary endpoint:

GMT and 4-fold rise of anti-SARS-CoV-2 neutralizing antibody 15 days after 3rd dose;

GMT and 4-fold rise of anti- SARS-CoV-2 neutralizing antibody 30 days after 3rd dose.

Secondary endpoint:

GMT of anti-SARS-CoV-2 IgG antibody, 4-fold rise, GMI, and proportions of neutralizing antibody titered ⩾ 1: 16, ⩾ 1: 32 and ⩾ 1: 64 before vaccination and 30 days after 3rd dose.

Immune persistence endpoint:

Anti-SARS-CoV-2 neutralizing antibody and GMT of IgG antibody 6 months and 12 months after full course of immunization, and the proportions of neutralizing antibody titered ⩾ 1: 16, ⩾ 1: 32 and ⩾ 1: 64.

Exploratory endpoints:

The vaccine efficay of recombinant COVID-19 vaccine (CHO cells) against COVID-19, severe cases and deaths 15 days after full course immunization in populations aged 18 years and above.

To evaluate the vaccine efficacy of recombinant COVID-19 vaccine (CHO cells) against different variants 15 days after full course immunization in populations aged 18 years and above.

4.3.2 SEQUENTIAL STUDY

Safety endpoint:

The incidence and serverity of any adverse reactions/events within 30 minutes after

CONFIDENTIAL

vaccination;

The incidence and serverity of solicited adverse reactions/events within 0-7 days after vaccination;

The incidence and severity of unsolicited adverse reactions/events within 8-30 days after vaccination;

The incidence of SAE and AESI observed after vaccination and up to 6 months after full course of immunization.

Immunogenicity endpoint:

Primary endpoint:

GMT and 4-fold rise of anti-SARS-CoV-2 neutralizing antibody 15 days after sequential vaccination of one booster dose;

GMT and 4-fold rise of anti-SARS-CoV-2 neutralizing antibody 30 days after sequential vaccination of one booster dose.

Secondary endpoint:

GMT of anti-SARS-CoV-2 IgG antibody, 4-fold rise, GMI, and proportions of neutralizing antibody titered ⩾ 1: 16, ⩾ 1: 32 and ⩾ 1: 64 before vaccination and 30 days after vaccination of the sequential booster dose.

Immune persistence endpoint:

Anti-SARS-CoV-2 neutralizing antibody and GMT of IgG antibody 3 months, 6 months, 9 months and 12 months after full course of immunization, and the proportions of neutralizing antibody titered ⩾ 1: 16, ⩾ 1: 32 and ⩾ 1: 64.

Exploratory endpoints:

The vaccine efficay of recombinant COVID-19 vaccine (CHO cells) against COVID-19, severe cases and deaths 15 days after sequential vaccination in population aged 18 years and above.

To evaluate the vaccine efficacy of recombinant COVID-19 vaccine (CHO cells) against different variants 15 days after sequential vaccination at different schedules in population aged 18 years and above.

4.4 Trial Hypothesis

① The 4-fold rise of neutralizing antibody of recombinant COVID-19 vaccine (CHO cells) after sequential vaccination with one dose of vaccine is not inferior to that of inactivated COVID-19 vaccine (Vero cells), and the threshold of non-inferiority is -10%. If the lower bound of 95% CI is >-10%, the hypothesis of non-inferiority in 4-fold rise between vaccine and control groups

CONFIDENTIAL

will be concluded.

② After sequential vaccination of one dose of recombinant COVID-19 vaccine (CHO cells), GMT is not inferior to COVID-19 inactivated vaccine (Vero cells), and the non-inferior threshold is 2/3 (GMT ratio; the difference on log10 scale is -0.176). If the lower bound of GMT ratio 95% CI is > 2/3, the hypothesis of non-inferiority in GMT between vaccine and control groups will be concluded.

4.5 Safety Observation Indicators and Grading Criteria

4.5.1 Safety Observation Indicators

(1) Adverse events collected in 0-7 days

Local adverse events: pain, induration, swelling, rash, redness and pruritis.

Systemic adverse events: fever, diarrhoea, constipation, dysphagia, anorexia, vomiting, nausea, myalgia(systemic), arthralgia, headache, cough, dyspnoea, systemic pruritis (no skin damage), abnormal skin mucosa, acute allergic reactions, fatigue, and dizziness

Other adverse events: any medical events other than the above mentioned during clinical studies, such as acute diseases, accidental injuries, etc.

(2) Adverse events collected in 8-30 days

Any medical events, such as acute diseases and accidental injuries occurred during 8~30 days in clinical studies.

(3) Pregnancy events

Collect all pregnancy events and outcomes between vaccination and end of the study (6 months after full course of vaccination). Follow-up of the new-borns should last at least 30 days after birth.

4.5.2 Adverse Event Grading Criteria

Local adverse events, systemic adverse events, vital signs, and laboratory testing parameters after vaccination were determined based on the *Guiding Principles of Adverse Events Grading Criteria for Clinical Trials of Prophylactic Vaccines* [6].

Table 2 Local Adverse Events Grading

Symptoms/signs	Grade 1	Grade 2	Grade 3	Grade 4
Pain	Do not or slightly affect physical activities	Affect physical activities	Affect daily life	Loss of basic self-care ability, or hospitalization

CONFIDENTIAL

Induration *, swelling * * #	2.5 ~ < 5 cm in diameter or 6.25 ~ < 25 cm^2 in area not or slightly affecting daily life	5 ~ <10cm in diameter or 25 ~ < 100cm^2 in area or affecting daily life	Diameter ≥10 cm or area ≥100 cm^2 or ulceration or secondary infection or phlebitis or sterile abscess or wound drainage or seriously affect daily life	Abscess, exfoliative dermatitis, dermal or deep tissue necrosis
Rash *, flush * * #	2.5 ~ < 5 cm in diameter or 6.25 ~ < 25 cm^2 in area not or slightly affecting daily life	5 ~ < 10 cm in diameter or 25 ~ < 100 cm^2 in area or affecting daily life	Diameter ≥10 cm or area ≥100 cm^2 or ulceration or secondary infection or phlebitis or sterile abscess or wound drainage or serious affect daily life	Abscess, exfoliative dermatitis, dermal or deep tissue necrosis
Pruritus	Itching at the vaccination site was relieved by itself or within 48 hours after treatment	Itching at the vaccination site did not relieve within 48 hours after treatment	Affect daily life	NA

Note: * In addition to measuring the diameter directly for grading evaluation, the progress and change of measurement results should also be recorded.

** Maximum measured diameter or area shall be used.

The evaluation and grading of induration and swelling, rash and redness should be based on the functional grading and actual measurement, with higher grading be selected.

When judging severity, it can be judged as a next grade adverse event only when it meets the grading criteria of the previous one.

Table 3 Systemic Adverse Events Grading

Signs	Grade 1	Grade 2	Grade 3	Grade 4
Fever * (axillary temperature ℃)	37.3 ~ < 38.0	38.0 ~ < 38.5	38.5 ~ < 39.5	≥ 39.5 for more than 3 days
Diarrhea	Mild or transient, 3 ~ 4 times/day, abnormal fecal characteristics, or	Moderate or persistent, 5 ~ 7 times/day, abnormal	> 7 times/day, abnormal fecal characteristics,	Hypotensive shock, requiring hospitalization

CONFIDENTIAL

	mild diarrhea lasting less than 1 week	fecal characteristics, or diarrhea > 1 week	hemorrhagic diarrhea, orthostatic hypotension, and electrolyte imbalance, requiring intravenous infusion > 2L	
Constipation *	Need fecal softener and dietary adjustment	Need laxative drugs	Stubborn constipation requires manual dredging or enema	Toxic megacolon or intestinal obstruction
Dysphagia	Mild discomfort when swallowing	Restricted diet	Diet and conversation are very limited; can't eat solid food	Can't eat liquid food; Need intravenous nutrition
Anorexia	Loss of appetite, but no reduction in food intake	Loss of appetite, food intake decreased, but body weight did not decrease significantly	Loss of appetite and obvious weight loss	Need measures to intervene (e.g., gastric tube feeding, parenteral nutrition)
Vomiting	1 ~ 2 times/24 hours without affecting activities	3 ~ 5 times/24 hours or limited activity	> 6 times within 24 hours or intravenous rehydration is required	hospitalization or other nutrition due to hypotensive shock
Nausea	Transient (< 24 hours) or intermittent and normal food intake	Persistent nausea leads to reduced food intake (24 ~ 48 hours)	Persistent nausea leads to almost no food intake (> 48 hours) or need for intravenous rehydration	Life Threatening
Myalgia (local)	not affect daily activities	Slightly affect daily activities	Severe myalgia, seriously affects daily activities	Emergency or hospitalization
Arthralgia	Mild pain without hindering function	Moderate pain, need analgesics and/or pain interferes with function, but does not affect daily activities	Severe pain, need for painkillers and/or pain affects daily activities	Disabling pain
Headache	not affect daily activities and not need treatment	Transient, slightly affect daily activities and may require	Seriously affect daily activities and need treatment or intervention	Intractable, requiring emergency or

CONFIDENTIAL

		treatment or intervention		hospitalization
Coughing	Transient, without treatment	Persistent cough, effective treatment	Paroxysmal cough, treatment uncontrollable	Emergency or hospitalization
Dyspnea	Dyspnea during exercise	Dyspnea in normal activity	Difficulty breathing at rest	Difficulty breathing, needing oxygen therapy, hospitalization or assisted breathing
Pruritus at non-vaccination site (No skin damage)	Slight itching, not or slightly affects daily life	affects daily life	Itching makes it impossible for daily life	NA
Abnormal Skin Mucosa	Erythema/pruritis/color	Diffuse rash/maculopapular rash/dryness/desquamation	Blister/exudation/desquamation/ulcer	dermatitis involves mucosa, or erythema multiforme, or is suspected of Stevens-Johnson syndrome
Acute Allergic Reaction * *	Local urticaria (blister) without treatment	Local urticaria, requiring treatment or mild angioedema, requiring no treatment	Extensive urticaria or angioedema requiring treatment or mild bronchospasm	Anaphylactic shock or life-threatening bronchospasm or laryngeal edema
Fatigue	Not affect daily activities	Affect daily activities	Seriously affect daily activities and cannot work	Emergency or hospitalization

Note: * Axillary temperature is usually used in China,tympanic temperature/temporal artery temperature = axillary temperature +0.5 ℃. When persistent high fever occurs, the cause of high fever should be determined as soon as possible;

* For constipation, attention should be paid to the changes before and after vaccination;

When judging severity, it can be judged as a next grade adverse event only when it meets the grading criteria of the previous one.

CONFIDENTIAL

For clinical abnormalities not covered in the above table, the severity grading evaluation of adverse events should be carried out according to the following criteria.

Grade 1	Grade 2	Grade 3	Grade 4	Grade 5
Mild: Short-term (< 48h) or slight discomfort, which does not affect activities and does not need treatment	Moderate: Mild or moderate activity restriction, which may require medical treatment, no or only mild treatment	Severe: obvious activity Restricted, need to see a doctor and receive treatment, and may need to be hospitalized	Critical: It may be life-threatening, severely limited in activities, and needs monitoring and treatment	Death

4.5.3 Relationship between Adverse Events and Investigational Vaccines

Impossible: Adverse events occur due to other factors, and there is sufficient evidence to prove that adverse reactions/events are caused by other reasons and unrelated to d vaccination.

Unlikely: The occurrence of adverse events may be caused by other factors, such as the clinical status of the subject, other treatments or accompanying medication, which are inconsistent with the known adverse reactions of vaccination.

Possible: Adverse events are consistent with known investigational vaccine information, have a reasonable temporal sequence with vaccination, and/or have occurred for vaccination. There is also a causal relationship with the investigational vaccine, but it may also be related to other factors.

Likely: Adverse events are consistent with the known investigational vaccine information and have causal relationship with the investigational vaccine, and cannot be explained by other factors, such as the clinical status of the subject, other treatments or concomitant medication.

Definite: Adverse events are consistent with the known investigational vaccine information and have a causal relationship with the investigational vaccine, and this relationship cannot be explained by other factors, such as the clinical status of the subject, other treatments or accompanying drugs. In addition, adverse events occurred repeatedly when the subjects used the investigational vaccine again.

4.5.4 Adverse Event of Special Interest

According to the CIOMS VII definition, AESI refers to a class of scientific and medical events (including serious or non-serious) related to investigational vaccines or projects, which require investigators to continuously monitor and quickly communicate with the sponsor. These events need further study to understand their characteristics; Depending on the nature of the event,

CONFIDENTIAL

the sponsor also needs to communicate quickly with other relevant parties (such as regulatory authorities).

AESI related to this vaccine include: **nervous system diseases** (generalized convulsions, Guillain-Barre syndrome, acute disseminated encephalomyelitis, aseptic meningitis, general convulsions, peripheral facial paralysis, loss of smell and taste), immune system diseases (vaccine-related respiratory diseases increase, anaphylactic shock, multisystem inflammatory syndrome in children), **respiratory diseases** (acute respiratory distress syndrome), **cardiac system diseases** (acute cardiovascular injury (including myocarditis, pericarditis, arrhythmia, heart failure, myocardial infarction), blood system damage (thrombocytopenia, coagulation dysfunction (including coagulation diseases, thrombosis, thromboembolism, internal/external bleeding, stroke)), **kidney system diseases** (acute kidney injury), liver system diseases (acute liver injury) and **skin system diseases** (chilblain-like lesions, single organ skin vasculitis, erythema multiforme, alopecia). The follow-up period of AESI is 6 months after the full course of immunization.

4.6 Randomization and Blinding

4.6.1 Randomization

Stratified block randomization method will be employed in the study to perform stratifications according to sequential vaccination time (4-6 months, 7-9 months, and >9 months after 2 doses). Interactive Web Response System (IWRS) will be used in subject randomization and vaccine allocation.

The randomization statistician will produce subject randomization list and vaccine randomization list by SAS 9.4 (ot later version), which will be imported into IWRS by system engineer. After one subject is sreened successfully, the investigator at each center will log onto IWRS to get randomization number of the subject. Before vaccination, the investigator will receive the vaccine number from IWRS and inoculate vaccines accordingly. If the vaccine is damaged, the investigator could acquire a new vaccine number from IWRS and inoculate the vaccine with new vaccine number.

4.6.2 Blinding

Before the start of the study, the staffs from sponsor who will not participate in the clinical trial will blind the vaccines to be used in the study together with the unblinded randmization statistician. The vaccine label will be sticked on the specified position of each vaccine according to vaccine randomization list. The randomization statistician will supervise and guide the process of blinding. After blinding is completed, the vaccine randomization list will be sealed by the unblind randomization statistician. The whole blinding process will be recorded and written in a

CONFIDENTIAL

document named blinding records, which is one of the important documents of this trial. The blinding personnel cannot participate in other related works of the trial, and they are not allowed to tell any information to any personnel who participats in the trial.

The subject randomization list and vaccine randomization list will be sealed, including randomization number, vaccine number and the corresponding group assignment, together with the randmozation seed number. They will be well sealed and saved until the database is locked.

4.6.3 Emergency Blind Code Breaking

In an emergency, when the investigator thinks that the group assignment information of one subject is beneficial for the handling of adverse events, it will be broken through the Emergency Blind Code Breaking Module in IWRS. The investigators need to discuss with the Sponsor and both shall reach agreement before performing emergency blind code breaking.

5. SCREENING AND WITHDRAWAL OF SUBJECTS

The subjects must fulfill all eligibility criteria to be involved in this study. No exemptions from any in-/exclusion criteria will be allowed. If any deviation from eligibility is retrospectively detected for an already randomized subject, the investigator and sponsor must determine immediately the safety to treat this subject further within the study.

5.1 Subject Population

5.1.1 Inclusion Criteria

- Age range: populations aged 18 years and above;
- Judged by the investigator that the health condition is well after inquiry and physical examination;
- Hasn't been vaccinated with any COVID-19 vaccines before (*Phase I Study*)
- Vaccinated with 2 doses of Sinopharm CNBG's inactivated COVID-19 vaccine (*Sequential Study*);
- Female subjects who are not nursing or pregnant at the time of enrolment (negative urine pregnancy test) and have no family planning within the first 6 months after enrollment. Effective contraceptive measures have been taken within 2 weeks before inclusion.
- During the whole follow-up period of the study, be able and willing to complete the whole prescribed study plan;
- With self ability to understand the study procedures, the informed consent & voluntarily sign an informed consent form and is able to comply with the requirements of the clinical study protocol.

5.1.2 Exclusion Criteria

CONFIDENTIAL

- Confirmed cases, suspected cases or asymptomatic cases of COVID-19;
- With a history of SARS and MERS infection (self-report, on-site inquiry);
- Has vaccinated with any other vaccines rather than one or more doses of Sinopharm CNBG inactivated COVID-19 vaccine;
- Axillary temperature ≥ 37.3 ℃ (forehead temperature ≥ 37.8 ℃);
- Previous severe allergic reactions to vaccination (such as acute allergic reactions, urticaria, dyspnea, angioneurotic edema or abdominal pain)
- Allergy to known components of recombinant COVID-19 vaccine (e.g., aluminum and histidine);
- Severe respiratory diseases, severe liver and kidney diseases, hypertension (systolic blood pressure ≥ 150 mmHg, diastolic blood pressure ≥ 90 mmHg), diabetic complications, malignant tumors, various acute diseases or acute attacks of chronic diseases;
- Has been diagnosed with congenital or acquired immunodeficiency, HIV infection, lymphoma, leukemia or other autoimmune diseases;
- Has a history of convulsions, epilepsy, encephalopathy, long-term alcohol and drug abuse, history of thyroidectomy, history of infectious diseases, mental illness or family history (direct);
- With Congenital malformation or developmental disorder, genetic defect, severe malnutrition, etc.;
- Has a history of coagulation dysfunction (e.g. coagulation factors deficiency and coagulation diseases);
- Asplenia or splenectomy, functional asplenia caused by any situation;
- Receving anti-TB (tuberculosis) therapy;
- Received immunoenhancement or inhibitor therapy within 3 months (continuous oral or IV administration for more than 14 days);
- Received other vaccines within 14 days before vaccination;
- Received blood products within 3 months before vaccination;
- Received other investigational drugs within 6 months before vaccination;
- Plans to move before the end of the study or leave the local area for a long time during the scheduled study visits;
- Other circumstances judged by investigators that are not suitable for this clinical trial.

5.1.3 Criteria for Early Withdrawal from the Trial

Early withdrawal means that the subjects fail to complete vaccination and blood collection

CONFIDENTIAL

according to the protocol, and the investigators decide whether to continue the follow-up research according to the situation. In any of the following situations, the subject will withdraw from the clinical trial in advance.

- Subjects asked to withdraw from clinical trials;
- Intolerable adverse events, whether related to the investigational drug or not
- The health status of the subjects does not allow them to continue to participate in this trial.
- Subjects are vaccinated with other investigational vaccines during the study period.
- Any reasons the investigator deems necessary.

5.1.4 Criteria for Clinical Trial Suspension and Termination

In case of any of the following circumstances, the clinical trial shall be suspended or terminated, and the investigator, the sponsor and the ethics committee shall jointly decide whether to terminate the clinical trial in advance:

- After vaccination, the number of subjects with adverse reactions with severity of Grade 3 exceeds 15% of the number of subjects vaccinated in this subgroup.
- One case of Grade 4 adverse reaction or SUSAR was confirmed to be related to vaccination.

In case of any of the following circumstances, the clinical trial shall be terminated:

- The Sponsor found that the vaccine has potential safety hazard, or the study has quality problems and requires the complete termination of the trial.
- The Ethics Committee requests termination of the trial because of the ethical violation in the study.
- The administrative authority requests termination of the trial.

6. PRODUCT INTRODUCTION

6.1 Description and Characteristics of the Investigational Products

Recombinant COVID-19 vaccine (CHO cells) is a recombinant protein expressed by recombinant Chinese hamster ovary (CHO) cells and designed based on the receptor binding domain (RBD) of S protein of SARS-CoV-2. Engineered cells were cultured, harvested and purified by a series of processes, and then added with aluminum hydroxide adjuvant.

This product is a sterile formulation, packed in vial with a specification of 0.5 ml/dose/vial. After shaking, it is an opalescent suspension, which can be layered due to precipitation and easily shaken away without lumps.

The main active ingredient of this product is the recombinant protein formed by the receptor

CONFIDENTIAL

binding region of SARS-CoV-2 spike protein. Excipients include sodium chloride, aluminum hydroxide adjuvant, histidine, etc. Each dose contains 20μg of recombinant protein, 4.38 mg of sodium chloride, 0.39 mg of histidine, 0.30 mg of aluminum and water for injection. Used to prevent diseases caused by SARS-CoV-2.

6.1.1 Investigational Vaccine

Investigational Vaccine: Recombinant COVID-19 Vaccine (CHO cell)

Manufacturers: Sinopharm CNBG National Vaccine and Serum Institute (NVSI),
Lanzhou Institute of Biological Products Co., Ltd. (LIBP)

Specifications: 0.5 ml/vial per human use, which contains 20ug of viral protein antigen.

Storage Condition: 2-8 ℃.

Vaccine Batch Number: 20210823

Expiry Date: August 26, 2023

Control vaccine: Inactivated COVID-19 Vaccine (Vero cell)

Manufacturer: Beijing Institute of Biological Products Co., Ltd. (BIBP)

Specifications: 0.5 ml/vial per human use, which contains 6.5U of antigen.

Storage conditions: 2-8 ℃

Vaccine Batch Number: 2021071931.

Expiry Date: July 22, 2023

6.1.2 Production Process

The recombinant COVID-19 vaccine (CHO cells) developed in this project takes CHO cells as the express system and SARS-CoV-2 spike protein as the target, applying genetic engineering technology to construct and screen stable cell lines with recombinant expression protein. After culture, harvesting and a series of purification processes and adding aluminum hydroxide adjuvant, the vaccine is finished after filling.

6.2 Vaccine Packaging and Labeling

All investigational vaccines are packaged in doses in boxes with the same appearance and only marked by the vaccine number. Each study ID will be matched with a unique vaccine number, the format of the vaccine number is AE+4-digit Arabic number. See the following label for detailed information.

CONFIDENTIAL

6.2.1 Vaccine Labeling

(1) Each dose of vaccine is packaged separately and the vaccine number is sticked onto.

(2) After the vaccine is put into use, the initials of the subject and the vaccination date shall be filled on the outer package.

Clinical Trial Labeling Illustration

Outer Packaging Box Label

**Recombinant COIVD-19 Vaccine (CHO cell)**
**For Clinical Use Only**
**Vaccine Number：AExxxx**
**Initials： Vaccination Date： YY MM DD**
**Storage at 2-8℃, 0.5mL/dose**
**Batch Number：**
**Expiry date:**

Vaccine Label:

**Recombinant COVID-19 Vaccine(CHO cell)**
**（For Clinical Use Only）**
Vaccine Number：AExxxx
Batch Number：XXXX
Expiry Date：XXXXXX

CONFIDENTIAL

6.3 Vaccine Storage and Transportation

Vaccines should be stored and transported at 2 ~ 8 ℃ away from light to prevent freezing. The temperature during vaccine transportation and storage shall be dynamically monitored and recorded. If the storage and transportation temperature conditions exceed the specified range, the on-site investigators should immediately contact the personnel of the responsible clinical institutions and the sponsors to decide whether the vaccine can be used.

The management of receiving, keeping, formulation, recovery, returning/destroying of the investigational vaccine shall conform to the requirements of relevant laws and regulations.

6.3.1 Vaccine Transportation

The whole process of vaccine management should meet the cold chain requirements, and there should be vaccine transportation and storage conditions that meet the requirements of the protocol. During the vaccine transportation, there should be a transportation sheet and temperature monitoring. Upon arrival, the packaging condition and unpacking temperature should be recorded. After the recipient receives the vaccines, the transportation sheet should be signed, faxed, or copied to the sender. Both parties should properly keep the transportation sheet.

6.3.2 Vaccine Storage and Distribution

The investigational vaccines should be kept in a separate area, locked in dedicated cabinet and managed by dedicated personnel. Vaccine recipient must verify and record the batch number, expiration period and delivery status of vaccines, establish work forms for vaccine handover, registration, use and recovery, fill in them as required, and keep them in the work records.

The trial vaccine shall not be used in non-clinical trial population.

6.3.3 Vaccine Records

Vaccine handover records: The sponsor will provide investigational vaccines, control vaccines (placebo) and vaccine handover sheet, and investigator will verify the name, batch number and quantity of the vaccine upon receiving.

Vaccine registration and use records: the investigator shall establish vaccine registration and use records and distribute investigational vaccine and control vaccines according to the number of people in the observation groups.

Vaccine recycle record: The abandoned, expired and the remaining vaccines in this trial are returned to sponsor. Sponsor receives vaccines and verifies the batch number and quantity of vaccines, fills in the vaccine handover form, and makes relevant records, which are signed by vaccine manager and sponsor representatives

6.4 Administration and Immunization Schedule

Route of Administration: lateral deltoid muscle of either upper arm, intramuscular injection.

Vaccination Procedure: Among the population who had completed two doses of inactivated COVID-19 vaccines according to the 0 and 21 days (+7 days) immunization schedule, and the interval between the two doses will be no longer than 8 weeks according to *Technical Guidelines for Inoculation of SARS-COV-2 Vaccine (First Edition)* issued by National Health Commission in China[39]one dose of recombinant COVID-19 vaccine (CHO cells) or inactivated COVID-19 vaccines (Vero cells) will be inoculated at different time points of 4~6 months, 7~9 months, and >9 months for sequential immunization

CONFIDENTIAL

7. STUDY WORKFLOW AND VISITING METHOD

7.1 Schedule of Visits

Table 4 lists the assessment items and procedures according to the phase of the clinical studies and visit.

7.2 Study Procedure

7.2.1 Screening Period, Baseline Period, and Vaccination Procedure (D0) (Phase I+Sequential)

The first visit was conducted according to the following procedure and evaluation processes.

✓ **Recruitment**

The investigators will jointly publicize this clinical trial recruitment notice to qualified volunteers/guardians, examined, and approved by the Ethics Committee, with local medical staff, and recruit and register candidate subjects based on the principle of voluntary participation. During the study, the recruitment should be adjusted in real time progress, to ensure that the gender and age of the participants are relatively balanced

✓ **Informed Consent**

Informed consent refers to the voluntary participation of subjects in clinical studies. Prior to the commencement of any test-related procedures (any genetics-related sample collection, testing, etc.), a written informed consent must be obtained from the subject.

After arriving at the research site, the subjects first give informed consent. The investigators inform the subjects of the informed consent form of this clinical trial orally and in writing, including study purpose, procedure, duration, risks and benefits, etc. Under the condition of voluntary participation, the subjects and research doctors signed an informed consent form together. The informed consent form shall be made in duplicate, and the guardian or principal of the volunteer shall keep a copy, and the original shall be kept at the study site.

Collect contact information (address, e-mail, landline, and mobile phone numbers, etc., if applicable) of subjects and their families.

✓ **Physical Examination, Inclusion/Exclusion Criteria**

Volunteers who signed the informed consent were given height, weight, body temperature, blood pressure (aged 18 and above), cardiopulmonary auscultation, skin examination. Within 10 minutes before the temperature test, the subjects are forbidden to eat or drink anything hot or cold, or smoke.

According to the "inclusion criteria" and "exclusion criteria", the investigators inquire,

CONFIDENTIAL

understand, and check the vaccination records of the subjects, that is, the past medical history, and judged whether the subjects should be enrolled in this clinical trial according to the inquiry results.

Women of childbearing age (from menarche to menopause) need to collect about 2ml urine samples for urine pregnancy test and the result should be negative before randomization.

✓ **Study Number Assignment**

Eligible subjects are assigned unique study numbers in sequence. Once the study number is assigned, it cannot be reassigned to other subjects.

✓ **Sample Collection before Vaccination**

About 12.5 ml of blood samples are collected on an empty stomach for SARS-CoV-2 antibody detection after recruitment.

Nasopharyngeal swabs are collected for PCR detection to establish antibody baseline and PCR baseline for booster inoculation. After sample collection, the subjects can enter the following process.

✓ **Vaccination**

Subjects can be vaccinated after sample collection. Before vaccination, it is necessary to check the information of subjects and the investigational vaccines, obtain vaccines with corresponding numbers and open the outer package. After the vaccine label and outer package label are checked, fill in the initials of the vaccinee's name and vaccination date on the label of the outer package.

Vaccination site is at the lateral deltoid muscle of upper arm, in principle, the non-habitual arm of the subject.

During vaccination, the investigational vaccine and control vaccine should be kept at 2 ~ 8 ℃ (the temperature should be monitored and recorded every 1 hour), and it should take no more than 30 minutes from the time the vaccine is taken out of the heat preservation container to the completion of vaccination.

During vaccination, in case of abnormal color, damage, insufficient loading or precipitation, it is necessary to stop vaccination immediately, report to the sponsor, auditor and on-site person in charge, discard the original vaccine according to the procedure after confirming the damage of the vaccine, and obtain a new vaccine number in the system by the distribution principle of the central randomized system after approval.

Record the injection site (right arm or left arm) and vaccination information.

✓ **On-site Medical Observation**

Observe the immediate adverse reactions 30 minutes after vaccination. Doctors explain on-

CONFIDENTIAL

site adverse reaction judgment, measurement methods, recording methods, precautions, reporting methods, etc. Diary cards, rulers and thermometers are distributed and train subjects how to use thermometers correctly, observe adverse events and fill in diary cards within 0-7 days after vaccination, and inform them of the time to make an appointment to return the diary cards (return the completed diary cards on 30th day-V6 after vaccination).

Ask the subjects to try to take temperature measurements at the same time every day within 7 days after vaccination.

Before leaving the study site, the vaccination site and body temperature of the subjects should be checked, and the observed adverse events should be evaluated and recorded in the electronic system.

Inform the subjects that they should take the initiative to provide any adverse reactions and accompanying medication. If there is any reaction after vaccination, it is necessary to inform the study center in time. PI or qualified SI will evaluate them, provide the subjects with correct response plan, and ask them to return to the clinic for immediate evaluation if necessary

7.2.2 Procedures Performed at the Subsequent Vaccination Visit (Phase I)

- ✓ Study investigator will review the diary cards with subjects and assess and record all solicited, reported reactions, AEs, SAEs (as applicable) and concomitant medications in the electronic system.
- ✓ Vital signs (if applicable, including body temperature and pulse, etc.), will be obtained prior to the vaccination. A targeted physical examination should be performed by a study physician if assessed as needed based on medical interview concerning period between Visit 1 and Visit 5. Counsel women of childbearing potential on contraception and avoidance of pregnancy. A urine pregnancy test will be performed within 24 hours prior to the vaccination on all female subjects of childbearing potential. Results must be negative and known prior to the vaccination;
- ✓ Examine the vaccination site for previous dose.
- ✓ Eligibility criteria will be reviewed with subjects prior to the subsequent study vaccination to assure continued eligibility.
- ✓ About 12.5 ml of blood samples are collected before 3rd dose (Phase I) on an empty stomach for SARS-CoV-2 antibody detection after recruitment.
- ✓ Nasopharyngeal swabs are collected for PCR detection to establish antibody baseline and PCR baseline for booster inoculation. After sample collection, the subjects can enter the following process.
- ✓ Subjects will receive the subsequent dose of study vaccine via IM injection in the deltoid muscle of the non-dominant arm. The site of injection (right or left arm) and time of

CONFIDENTIAL

administration will be recorded in the electronic system. Subjects will be observed in the clinic for at least 30 minutes after the vaccination. The vaccination site will be examined, and observed AEs will be assessed and recorded in the electronic system prior to discharge from the study site.

- ✓ Subjects will be provided with new subject diaries to record daily temperature, solicited injection site and systemic reactions, any unsolicited AEs, and concomitant medications. Subjects will be encouraged to take their temperature on the vaccination day and then at the same time as much as possible each day for 7 days after the vaccination day.
- ✓ Subjects will be instructed on how to correctly complete the subject dairy within 0-7 days after vaccination, prior to discharge from the study site.
- ✓ The subjects will be instructed to return the completed subject diary, covering period between Visit 4 and Visit 6.

7.3 Safety Observation and Follow-up（Phase I and Sequential Study）

7.3.1 Safety Observation

(1) Observe the site for 30 minutes after vaccination to collect local and systemic adverse events.

(2) Telephone follow-up once after vaccination, within 1-3 days and 4-7 days respectively, the subjects were followed-up by telephone and guided to observe the adverse events correctly and fill in the diary card.

(3) On the 30th day after vaccination, the investigator reviews the filling of the diary card during this period and re-collect the diary card.

(4) On the 8th -30th day after vaccination, the investigator uses telephone follow-up once a week, combined with active report of the subjects to monitor the safety.

(5) When it is known that the subject has adverse reactions/events of Grade 3 or above, a face-to-face visit should take place within 24 hours.

(6) SAEs and AESIs are observed from the first dose to 6 months after the full course of vaccination by means of monthly telephone follow-up and self-reports from the subjects.

(7) If the subject develops any COVID-19 like symptom, he/she should contact with the doctors on the ICF/diary card immediately.

7.3.2 Safety Information Record

After vaccination, all subjects should complete the diary card. Investigators will ask subjects about adverse events during on-site visits and telephone follow-up after vaccination (except for scheduled/unscheduled visits to the research center). If the subject has persistent local or systemic

CONFIDENTIAL

reaction within 0-30 days after vaccination, it should be continuously recorded in diary cards/follow-up system and reexamined until the symptoms are relieved.

7.4 Laboratory Testing

Collect laboratory samples (including blood samples) after physical examination, and complete sample collection before vaccination.

The sampling time and arrangement of laboratory testing samples during the study period are shown in the testing flow chart, and the samples are tested and analyzed by the central and/or local laboratories.

Pregnancy Sample Collection

All female subjects of childbearing age need to collect urine/blood samples for pregnancy test, and the test results will be used for screening.

Nucleic Acid Sample Collection

All subjects need to collect nasopharyngeal swabs for RT-PCR detection at the same time of recruitment, and the test results will be used for subsequent analysis.

Blood Sample Collection before Immunization (D0)

After recruited, all subjects collect about 12.5 ml venous blood before vaccination, separate the serum within 24 hours, package into multiple test tubes (each tube is not less than 0.5 ml), and store at-20 ℃ or below for antibody detection.

Blood Sample Collection after Immunization

Phase I Study: About 12.5 ml of venous blood will be collected from all subjects before 3rd dose, 15 days, 30 days, 6M, 12M after 3rd dose, and the serum was separated within 24 hours and packed into 2 tubes (each tube is not less than 0.5 ml), which were stored at -20℃ and below for immunogenicity neutralizing antibody detection.

Sequential Study: About 12.5 ml of venous blood will be collected from all subjects at 15 days, 30 days, 3 months, 6 months, 9 months and 12 months after immunization, and the serum was separated within 24 hours and packed into 2 tubes (each tube is not less than 0.5 ml), which were stored at -20 ℃ and below for immunogenicity neutralizing antibody detection.

Blood Sample Collection of Cases (Suspected or Confirmed)

After the subjects develop typical symptoms of SARS-CoV-2 infection, about 4 ml of venous blood is collected for antibody detection, and the serum is separated and packed into 2 tubes (each tube is not less than 0.5 ml) within 1-3 days. In addition, about 10ml venous blood is collected,

CONFIDENTIAL

and cytokines, cellular immunity and other detection items are carried out according to local medical conditions, laboratory clinical level. Serum is stored at -20℃ and below (antibody detection is carried out for confirmed and suspected cases).

Blood Sample Numbering Rule

Phase I Study

Before 1st dose: study number – pre0

Before 3rd dose: study number – pre1

15 days after 3rd dose (D15+10 days) study number – pre2

30 days after 3rd dose (D30 + 10 days) study number -pre3

6M after 3rd dose (6M + 30 days) study number -pre4

12M after 3rd dose (12M + 30 days) study number -pre5

Sequential Study

Before the sequential dose: study number – 0

15 days after vaccination (D15+10 days) study number – 1

30 days after vaccination (D30 + 10 days) study number -2

3 months after vaccination (D90 + 30 days) study number-3

6 months after vaccination (D180 + 30 days) study number -4

9 months after vaccination (D270 + 30 days) study number -5

12 months after vaccination (D360 + 30 days) study number-6

Suspected and confirmed cases are coded in P1, P2, P3…according to the order of blood collection.

CONFIDENTIAL

Table 4-1 Schedule of Events (Phase I Study)

Visit number	V1	V2	V3		V4	V5	V6	V7		V8	V9
Date (Safety followup method)	D0 👓	D3 ☎	D7 ☎	Weekly Call ☎ D8-D30	D30 👓	D60 👓	15d after V5 👓 D75	30d after V5 👓 D90	Monthly Call ☎ V7-V8 (D90-D240)	6M after V5 👓 D240	12M after V 👓 D420
Visit (window +/-days) The subsequent visit is scheduled accordingly	0D	-2D	-3D		+ 7D	+ 7D	+ 10D	+ 10D		+ 30D	+ 30D
**SCREENING/BASELINE**											
Study informed consent [1]	✓										
Inclusion/Exclusion criteria evaluation	✓										
Demographics	✓										
Medication history	✓										
Contraception [2]	✓				✓	✓					
IWRS Registration	✓				✓	✓					
**INTERVENTION**											
Vaccination	✓				✓	✓					
**SAFETY ASSESSMENT**											
Vital signs (blood pressure, body temperature, heart rate)	✓				✓	✓					
Height, Weight	✓				✓	✓					
Physical Examination	✓				✓	✓					
Observation at site after vaccination [3]	✓				✓	✓					
Telephone follow-up after vaccination		✓	✓		Once per week within V2-V6				Once per month within V7-V8		
Concomitant Medication	✓	✓	✓		✓	✓	✓	✓			
Adverse events	✓	✓	✓		✓	✓	✓	✓			
SAE	✓	✓	✓		✓	✓	✓	✓		✓	
Diary Card [4]	✓				✓	✓	✓				
**LABORATORY TESTING**											
COVID-19 PCR[5]	✓										
Neutralizing Antibody Test	✓					✓	✓	✓		✓	✓
Specific IgG Antibody Test (anti-N, anti-S protein)	✓					✓	✓	✓		✓	✓

Table 4-2 Schedule of Events (Sequential Study)

Visit number	V1	V2	V3	V4	V5	V6	V7	V8	V10	V11	V12	V13	V14
Date (Safety Follow-up Method)	D0 👓	D3 ☎	D7 ☎	D15 👓	D21 ☎	D30 👓	2M after V1 ☎	3M after V1 👓	4M after V1 ☎	5M after V1 ☎	6M after V1 👓	9M after V1 👓	12M after V1 👓
Visit (window +/-days) The subsequent visit is postponed accordingly	0d	-2d	-3d	+ 10d	+7d	+ 10d	+10d	+ 30d	+10d	+10d	+ 30d	+ 30d	+ 30d
**SCREENING/BASELINE**													
Study informed consent [1]	✓												
Inclusion/Exclusion criteria evaluation	✓												
Demographics	✓												
Medication history	✓												
Contraception [2]	✓												
IWRS Registration	✓												
**INTERVENTION**													
Vaccination	✓												
**SAFETY ASSESSMENT**													
Vital signs (blood pressure, body temperature, heart rate)	✓												
Height, Weight	✓												
Physical Examination	✓												
Observation at site after vaccination [3]	✓												
Telephone follow-up after vaccination	✓	✓	✓		✓		✓	✓	✓	✓	✓		
Concomitant Medication	✓	✓	✓	✓	✓	✓	✓	✓	✓	✓	✓		
Adverse events	✓	✓	✓	✓	✓	✓	✓	✓	✓	✓	✓		
SAE	✓	✓	✓	✓	✓	✓	✓	✓	✓	✓	✓		
Diary Card [4]	✓			✓									
**LABORATORY TESTING**													
COVID-19 PCR[5]							✓						
Neutralizing Antibody Test	✓			✓		✓		✓			✓	✓	✓
Specific IgG Antibody Test (anti-N, anti-S protein)	✓			✓		✓		✓			✓	✓	✓

CONFIDENTIAL

Notes:

1. Consent process completed, and form signed before any study-related procedures are conducted.
2. Urine/blood pregnancy test must be completed within 24 hours prior to vaccination for women of childbearing potential, and only negative test results are eligible for enrollment.
3. All subjects should be under observation for at least 30 minutes after vaccination.
4. Paper diary cards will be used for safety information records.
5. PCR testing could be performed any time during the study when the subject developed any suspected symptoms.

CONFIDENTIAL

7.5 COVID-19 Case Monitoring

This clinical trial adopts a monitoring modality with passive monitoring as the main approach and active monitoring as a supplement. After the subjects receives the vaccination, the monitoring of COVID-19 cases will be initiated until the end of the study, and relevant information will be collected. Fig.1 illustrates the overall workflow for case monitoring.

A. Passive Monitoring

Passive monitoring has two situations where subjects spontaneously report and when the subjects develop any symptoms, they actively go to a hospital to seek for medical care.

1) After the subjects develop symptoms, they actively report to the investigator through the hotline or email;

2) When the subjects visit a hospital after developing any symptoms, the subjects are monitored through hospital system;

- When the subject has fever and/or respiratory symptoms that meet the definition of a suspected case, the subject should inform the investigator and go to the designated place for PCR testing;
- If the subject visits a hospital for treatment, the investigator should arrange to collect nasopharyngeal swabs for PCR testing every 2-3 days; determine the clinical classification based on the severity and duration of symptoms, and isolate the mild and moderate cases; arrange hospitalization for severe and critical cases;
- For non-hospitalized patients, the subjects will be followed up by remote teleconsultation and re-tested by PCR testing every 3 days to monitor and document the progress of the illness, medication status, symptom severity and other related information. If the subject's condition deteriorates, he/she needs to be transferred to the hospital for timely treatment;
- For hospitalized patients, continue to pay attention to the development of the disease, and conduct necessary testing and examination such as blood routine, blood chemistry, imaging examinations as well as other medical treatment. Closely monitor and collect evidence related to the subject's diagnosis and treatment, and record the progress of the disease, medication, and the severity of symptoms until the outcome;
- Collect the PCR result, a confirmed case is determined based on the positive result;

CONFIDENTIAL

- A convalescent phase is defined as 3-4 weeks after the first positive PCR result. During the convalescent period, the subject's nasopharyngeal swab is collected for PCR re-test and neutralizing antibody test; if the PCR test result is still positive, repeated PCR testing needs to be performed once a week until the PCR result turns negative.

B. Active monitoring

1）Weekly teleconsultation to collect information；

2）Regularly monitor and collect information；

The subsequent procedures for suspected cases identified through the above two monitoring process approaches is the same as passive monitoring procedures.

Endpoint assessment of the cases

Establish an independent case Endpoint Assessment or Adjudication Committee (EAC) composed of 3 or more local medical experts to make a final diagnosis for each case. When a case meets the etiological indicators, the diagnosis and treatment progress document, case investigation document, serology, cytokines and other lab reports will be submitted to EAC, the case can be determined as a confirmed case of COVID-19 (clinical endpoint) after a blinded review of the EAC experts.

CONFIDENTIAL

Fig.1 Case Monitoring Flowchart

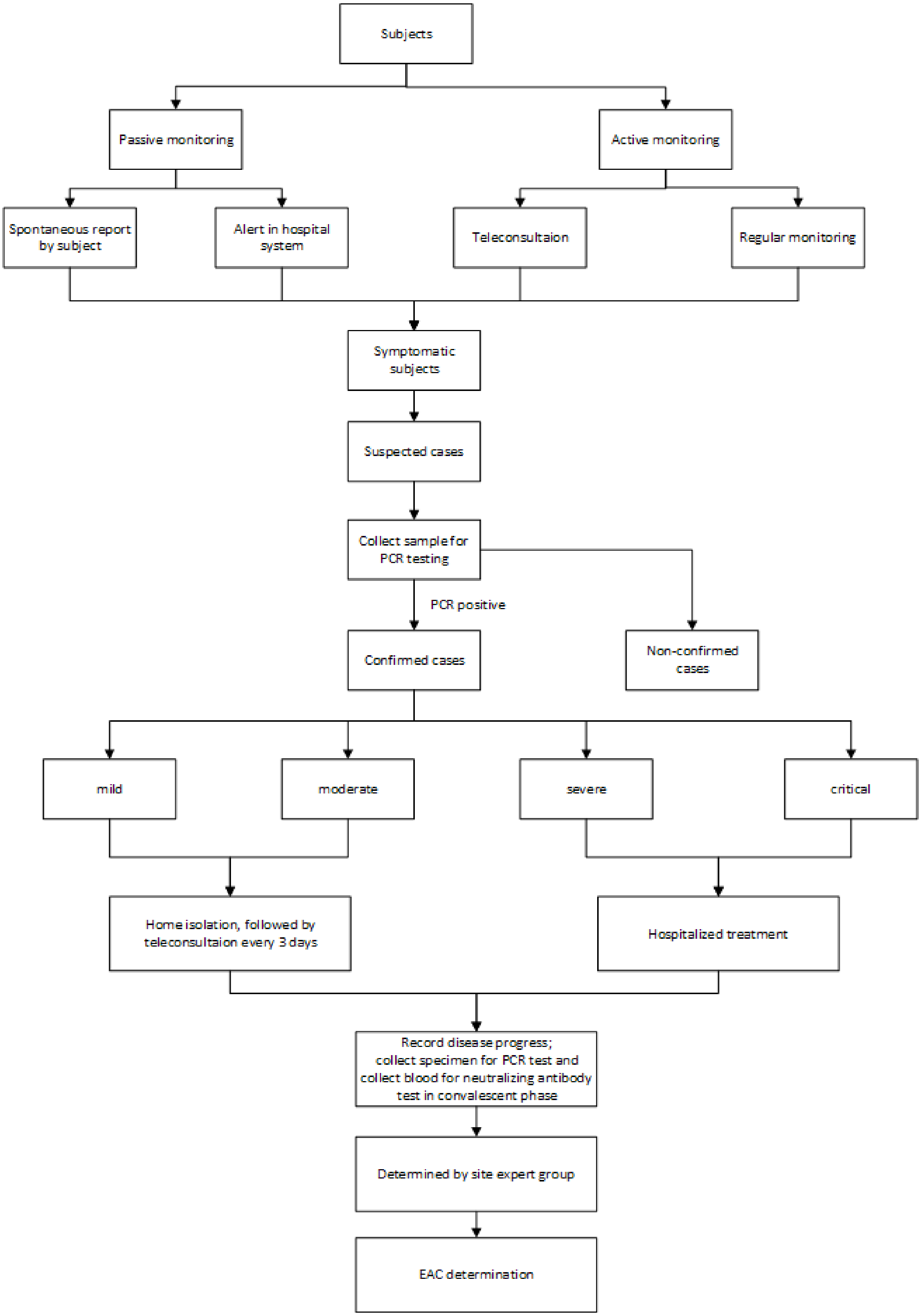

Table 5 Testing for PCR positive cases in acute and convalescent phase

	Clinical Classification	Acute Phase	Convalescent Phase
PCR-positive cases	Non-hospitalized-Asymptomatic / Mild	SARS-CoV-2 PCR testing	SARS-CoV-2 PCR testing
		IgM, IgG, Neutralizing antibody	IgM, IgG, Neutralizing antibody
		Pathogenic Differential diagnosis (if applicable)	
	Hospitalization-Moderate/Severe / Critical	SARS-CoV-2 PCR testing	SARS-CoV-2 PCR testing
		IgM, IgG, Neutralizing antibody	IgM, IgG, Neutralizing antibody
		CBC with differential (WBC, lymphocytes…)	
		Blood chemistry (liver enzymes, eGFR, LDH, CRP)	
		Chest CT scan/CXR	
		Pathogenic Differential diagnosis (if applicable)	
		Cytokines : IL-2, IL-6, IL-4, TNF-α, IFN-γ and other cytokines (if applicable)	

CONFIDENTIAL

7.6 Concomitant Medication

Within 0-30 days, actively follow up and observe the local and systemic adverse events of the subjects and record them on the vaccination diary card/follow-up system. At the same time, it is necessary to record the drug used of the subjects in the vaccination diary card.

Medication Allowed: During the clinical trial, if the subject has adverse events, necessary medication and medical treatment should be allowed.

Vaccines Allowed: Other vaccines are allowed during the trial, which should be carried out 14 days after the trial vaccination. Emergency vaccination, such as rabies vaccine and tetanus vaccine, is not restricted.

Medication Record: To understand the influence of drug use during the trial on vaccine safety and immunogenicity, and to collect adverse events that may be related to vaccination without omission, investigators should instruct the guardians of the subjects to record the medical treatment and medication in diary cards as much as possible and assist in collecting the hospitalization medication records of the subjects during the observation period. When SAE occurs, copies of corresponding medical records and medication records

should be collected and kept. The following drugs need to be transcribed into the electronic case report form (eCRF).

(1) Hormone/steroid drugs and other immunosuppressants.

(2) Antiallergic Drugs

(3) Antipyretics/analgesics/NSAIDs

(4) Prophylactic Vaccines

(5) Therapeutical Medical Products

(6) Antibiotics

(7) Antiviral Drugs

(8) Chinese Patent Drugs

(9) Others

7.7 Criteria for Suspension or Early Termination

In case of any of the following circumstances, the trial shall be suspended, and the investigator, the sponsor and the Ethics Committee shall jointly hold a meeting to decide whether to terminate the clinical trial early:

- One case of Grade 4 adverse reaction related to vaccination or SUSAR in any group, including death or life-threatening SAE;
- The number of subjects with adverse reactions of severity Grade 3 or above exceeds 15% of the subjects vaccinated in this subgroup;
- DSMB evaluation of clinical research has high potential safety risks.

CONFIDENTIAL

In case of any of the following circumstances, the clinical trial shall be terminated:

- Sponsor has found that the vaccine has potential safety hazards or the trial has quality related problems, and requiring the complete termination of the study.
- The Ethics Committee requests the termination of the trial because of the violation of ethics in the trial.
- Request by the administrative authority to terminate the trial

7.8 Protocol Violation and Deviation

7.8.1 Protocol Violations

The list of protocol violations is as follows (including but not limited to):

- Fail of obtain subject’s informed consent;
- Subjects who did not meet the inclusion criteria or met the exclusion criteria were enrolled in the study;
- Subjects received wrong research interventions (such as vaccination errors);

- Fail to report serious adverse events (SAE) within specified time;
- Received other investigational vaccines during the study period.

7.8.2 Protocol Deviations

The list of protocol deviations is as follows (including but not limited to):

- Not receive the investigational vaccine during the window period;
- Not collect blood during the window period;
- The interval between vaccination and other vaccines is not long enough, except for emergency vaccination such as rabies.

7.9 Study Duration

Early Subject Recruitment: 1 month;

Sequential Vaccination + immune persistence observation: about 18 months

Sample Testing: about 1 month

Complete the Summary Report: about 1 month

7.10 Site Closure

All subjects completed long-term safety observation for 6 months after vaccination and blood collection for 12 months after immunization. All original data are collated, entered, and verified and submitted to the statistician. After clarifying the data after blind audit by the statistician, the study site is closed.

CONFIDENTIAL

8. SAFETY DEFINITION, REPORTING AND MONITORING

8.1 General Precautions

Before the start of the trial, the sponsor shall strictly examine the trial site in accordance with GCP requirements, focusing on whether the environmental facilities of the trial site meet the requirements of "Vaccination Management Standards" and "Guiding Principles for Quality Management of Vaccine Clinical Trials". Ensure the first aid facilities and first aid equipment in the first aid room are complete and effective, and the first aid doctors have corresponding qualifications and capabilities. Emergency related personnel (emergency doctors, emergency nurses, ambulance drivers, etc.) are trained to be qualified and familiar with the transfer routes and procedures of the agreed hospital. They are on standby at the trial site during vaccination. The trial site shall be equipped with ambulances. The ambulance shall be parked in a fixed position to keep the vehicle in good condition and in an emergency and shall be under the command and transfer of the emergency response team at any time. During the vaccination period, the agreement hospital will make daily preparations for medical personnel, instruments and equipment, first aid drugs and first aid

sites to ensure that the subjects can receive timely treatment. The trial site shall formulate an emergency plan, stipulate personnel responsibilities, contact numbers, rescue routes and other measures to ensure timely handling of unexpected adverse events, and ensure effective contact between subjects and investigators so that any adverse events can be reported and handled quickly.

8.2 Risk Prevention Measures Related to COVID-19

(1) Trial site consideration

Investigators during recruitment should confirm whether there are new COVID-19 cases or suspected cases in the same village/community by investigating or inquiring volunteers. Whether fever, dry cough, fatigue, nasal obstruction, runny nose, sore throat, myalgia, diarrhea, shortness of breath and dyspnea occurred recently;

(2) Strengthen the management and personal protection of subjects during the study period

Disinfection of each functional area of clinical research should be strictly done in the test site according to regulations, and windows should be opened and ventilated regularly. Strictly implement independent areas and special passages, and the recipients and their accompanying personnel wait in relative divisions to avoid contact with people other than research doctors and nurses; All functional areas and public places should be equipped with hand disinfectants and temperature measuring devices, and recipients and their accompanying personnel entering the clinical research site should be equipped with masks to disinfect their hands and measure their body temperature.

CONFIDENTIAL

The trial site shall strictly disinfect each functional area of clinical research according to regulations, and regularly open windows for air-ventilation. Strict implementation of independent areas and special passages, recipients and their accompanying personnel waiting in relative zones, to avoid contact with research doctors, nurses and other personnel other than research; All functional areas and public places should be equipped with hand disinfectants and temperature measuring devices. Recipients entering the clinical research site and their accompanying personnel are required to be equipped with masks to disinfect their hands and measure their body temperature.

During the first dose of inoculation, the on-site investigators shall remind the subjects to strengthen their own protection. If COVID-19 pandemic occurs locally, the subjects shall be provided with necessary protective materials such as masks, alcohol, etc. in time, and close attention to the health status of the subjects shall be drawn, especially the symptoms related to COVID-19.

(3) Detection of COVID-19 RT-PCR

Subjects need to be tested for COVID-19 RT-PCR before first dose vaccination. PCR tests should be performed when subject visits the site for vaccination or blood collection, and regularly afterwards at the sites with permitted conditions. Investigators need to regularly follow up and track the subjects to identify whether they have developed fever, dry cough, fatigue and other symptoms recently. If the above symptoms exist, the subjects will be collected samples for etiological detection of SARS-CoV-2 according to the prevention and protection policy for COVID-19 and based on the symptoms of the subjects, or are advised to go to the hospital for blood testing and radiographic examination. If there are no symptoms such as fever, dry cough and fatigue, the subject should be home isolated according to the requirements of COVID-19 prevention and control protocol. If the subject is diagnosed as confirmed COVID-19 case, the positive PCR report at the time of diagnosis should be collected.

8.3 Handling and Reporting of Serious Adverse Events

Serious adverse events refer to adverse medical events such as disability or loss of function, the need for hospitalization or prolonged hospitalization of subjects, and congenital abnormalities or birth defects.

CONFIDENTIAL

Monitoring and reporting of adverse events in vaccine clinical trials are jointly completed by subjects, serious adverse event investigators, trial sites and responsible institutions at different observation time points with different stages.

The sponsor is the main body responsible for monitoring, evaluating and SAE reporting safety information of vaccine clinical trials. The person shall be designed as the administrator of clinical trial safety information monitoring and SAE reporting, and work with investigators to establish SOPs for clinical trial safety information monitoring and SAE reporting, know well the latest status of the safety information of the whole clinical trial, and timely report updates to all clinical trial institutions/ investigators and regulatory authorities.

If it is difficult to make a judgment on the correlation between SAE and vaccine or there is doubt about the judgment, when it is necessary to make a new judgment, the expert meeting shall make a judgment after argumentation.

(1) On-site Treatment Measures

Emergency plans for SAE treatment in clinical trials shall be established at the trial

site, and all relevant personnel shall be trained. If the subjects show serious adverse events, the investigators shall immediately take appropriate measures for the subjects and record them. The investigators must take relevant approaches to know in time any clinically significant diseases/events related to vaccination. According to relevant national regulations, the subjects should be received appropriate treatment in time in the designated hospitals.

Investigators at the trial site should follow up serious adverse events until the symptoms disappear or stabilize. The progress and outcome of all symptoms will be recorded in detail, and all drug treatments and medical treatments will be recorded at each follow-up. Investigators should truthfully record serious adverse events, which shall be evaluated and discussed in the final report after the test is completed or terminated.

During the whole observation process, if the subjects suffer from physical injuries caused by serious adverse reactions related to vaccination, which is confirmed by the expert investigation team, the local insurance company will give corresponding compensation.

(2) Reporting Procedures for Serious Adverse Events

- Reporting procedures of investigators

CONFIDENTIAL

Any serious adverse event, whether or not related to the investigational vaccine, the investigator must submit the initial report of the "Serious Adverse Event Report Form" to the drug administration department, the sponsor and the ethics committee by fax, e-mail or EDC system or personal delivery within 24 hours after learning about it.

Subsequently, the follow-up report of the "Serious Adverse Event Report Form" shall be submitted regularly until the end of the event. All information is reported in the "Serious Adverse Event Report Form" in the form of written reports, including description of adverse reactions/events, onset time and type, duration, intensity, causal relationship with vaccination, results, treatment methods (symptomatic treatment) and other relevant clinical and laboratory data.

When receiving the report of serious adverse events/reactions, the investigator shall, together with the sponsor, comprehensively consider the duration, scope, intensity, outcome and the willingness of the subject to decide whether the subject should continue to participate in the study or withdraw from the study in advance.

- Reporting procedure of sponsors

During the clinical trial of drugs, sponsors need to quickly report unexpected and

serious adverse reactions (SUSAR) that are definitely related to or suspicious of the tested drugs in the form of case-by-case safety reports according to the Standards and Procedures for Rapid Reporting of Safety Data during Clinical Trial of Drugs. If the researcher and the sponsor cannot reach an agreement on the judgment of the causal relationship between adverse events and drugs, either party's judgment cannot exclude those related to the test drugs, and the sponsor should also make a quick report.

For SUSAR that is fatal or life-threatening, the sponsor should report it as soon as possible after the first knowledge, but not more than 7 days, and report it within the following 8 days to improve the follow-up information (Note: the day when the sponsor first learned it is the 0th day). For SUSAR that is not fatal or life-threatening, the sponsor should report it as soon as possible after the first knowledge, but not more than 15 days. For other potential serious safety risk information, the sponsor should also report to the national drug evaluation agency as soon as possible, and at the same time make medical and scientific judgment on each situation. After the first report, the sponsor shall continue to track the serious adverse reactions and submit relevant new information or changes to the previous report in a timely manner in the form of a follow-up report. The reporting time limit shall be within 15 days from the date of obtaining the new information.

CONFIDENTIAL

8.4. Outcome of Serious Adverse Events

The outcomes of serious adverse events include: (1) Symptoms resolved (with sequelae); (2) Symptoms resolved (no sequelae); (3) Symptoms persist; (4) require hospitalization; (5) require medical intervention to prevent serious outcomes; and (6) death.

9. COMPLETION OF CLINICAL TRIAL

After obtaining the immunogenicity test results 30days after the full course of immunization, and completing the safety follow-up for no less than 1 month, and ensuring the authenticity, integrity and accuracy of clinical trial data, the database is locked and unblinded to perform safety, immunogenicity and vaccine efficacy evaluation, and blood collections for 12 months immune persistence are completed, the study site will be closed.

10. STATISTICAL CONSIDERATIONS

10.1 Sample Size Considerations

Sequential Study:

Assume 4-fold rise rate after sequential vaccination achieve 85%, 208 subjects in each arm will be required to have 80% power to conclude non-inferiority with margin of -10%

and one-sided significance level of 2.5% using Miettinen & Nurminen method.

Equal GMT after sequential immunization is assumed, and standard deviation of GMT after log10 transformation is considered to be 0.7. 250 subjects in each arm will be required to have 80% power to conclude non-inferiority with margin of 2/3 and one-sided significance level of 2.5%.

Considering about 15%~20% drop-out rate, 600 subjects in three sequential cohorts (4-6 months, 7-9 months and >9 months after 2 doses), and 1800 subjects (900 subjects in test and control groups respectively) in total are planned to enroll.

10.2 Analysis Set

Full Analysis Set (FAS): It includes all randomized participants who follow ITT principle, receive 1 dose of vaccine, and have valid immunogenicity data before the first dose.

Per-Protocol Set (PPS): It includs all participants who do not violate the inclusion criteria/exclusion criteria, receive 1 dose of vaccine, and have both valid pre- and post-vaccination immunogenicity data. PPS will be defined for immunogencity evaluations 15 and 30 days after sequential vaccination, respectively.

CONFIDENTIAL

Immunogenicity Persistence Set (IPS): It include the participants who are collected with blood samples at each time point for immunogencity persistence evaluation and with valid antibody data. Define the IPS for immunogenicity for 3, 6, 9, 12 months after sequential immunization respectively.

Safety Set (SS): It includes all participants who receive one dose of vaccination.

In immunogencity analysis, the participant who takes the vaccine erroneously will be included as randomized, and the safety data will be analyzed as treated.

10.3 Statistical Method

10.3.1 General Considerations

Summary statistics including the number of patients, mean, standard deviation, median, minimum and maximum, will be presented for all continuous variables. For categorical variables, per category, the absolute counts (n) and percentages (%) of patients with data, and if appropriate, the number of patients with missing data, will be presented.

All statistical analysis will be performed by using SAS 9.4 or later version.

10.3.2 Subject Disposition

The number of subjects who are enrolled, completed and discontinued will be summarized in each group. The reason for discontinuation will be analyzed as well.

The demographic characteristics will be summarized and described by treatment group.

10.3.3 Immunogenicity Analysis

ANCOVA model will be employed to analyze post-vacciantion GMT. In the model, log-trandformed GMT after vacciantion will be included as dependent variable, pre-vaccination GMT after log-transformation is the covariate and group assignment and sequential vaccination time (4-6 months, 7-9 months and >9 months after 2 doses) are the fixed effect. The adjusted LSMean in each arm and difference between the two groups together with 95% confidence interval (CI) will be estimated from the model. After inverse log-transformation, the adjusted GMT after vaccination and GMT ratio together with 95% CI between treatment and control groups will be derived. If the lower bound of 95%CI is larger than 0.67, the non-inferiority result will be concluded.

The 4-fold rise, and the proportions of antibody tittered ≥1:16、≥1:32 and ≥1:64 after vaccination in both vaccine and placebo groups will be estimated. The corresponding 95% CIs will be derived from Clopper-Pearson method. The difference between the two groups and their 95%CIs will be estimated by CMH method including the stratification factor (sequential vaccination time). If the lower bound of 95%CI in 4-fold rise rate is larger than -10%, the non-inferiority will be concluded.

CONFIDENTIAL

The descriptive statistics, including geometric mean and corresponding 95% CI, are employed to summarize GMT and GMI after sequential dose. The t-tests after log transformation will be compared between the two groups.

The reverse cumulative distribution plot is to depict the pre- and post-vaccination antibody data in the vaccine and placebo groups.

10.3.4 Safety Evaluation

Adverse events and serious adverse events will be coded using the Medical Dictionary for Regulatory Activities (MedDRA), and summarized by system organ class (SOC) and preferred term (PT). AEs will be summarized by solicied and unsolicited AEs as well. The treatment emergent adverse events (TEAEs) will be analyzed here. AEs occurring before vaccination will be listed.

All TEAEs, vaccine-related TEAEs, vaccine-unrelated TEAEs, Grade≥3 TEAEs and vaccine-related Grade≥3 TEAEs will be summarized by group. The frequency, subjects and percentage in each group will be reported. Fisher exact test will be used to compare the difference between the groups. The strat time and severity of TEAEs will be summaried.

The vaccine-related TEAEs and vaccine-unrelated TEAEs will be listed.

All TESAE, vaccine-related TESAEs and vaccine-unrelated TESAEs will be summarized by group. The frequency, subjects and percentage in each group will be reported. Fisher exact test will be used to compare the difference between the groups. All TESAEs will be listed.

10.4 Subgroup Analysis

The immunogencity analysis will be conducted in each sequential vaccination cohort (4-6 months, 7-9 months and >9 months after two doses).

10.5 Interim Analysis

No interim analysis will be planned in this study.

10.6 Multiplicity

Only when both non-inferiority conclusion in 4-fold rise and GMT after vaccaition between treatment and control groups are achieved, the non-inferiority between the two groups will be concluded finally. Therefore, no multiplicity adjustment is required here.

10.7 Handling of Missing Data

In FAS immunogenicity analysis, LOCF (Last Observation Carried Forward) method will be used to impute the missing values in antibody data after vaccination. And other immunogencity endpoints will be derived from the imputed data. The missing data in safety and immunogencity persietence evaluation will be not imputed.

CONFIDENTIAL

11. ETHICAL AND REGULATORY MATTERS

This clinical trial will be conducted by the Sponsor, the Investigator, and delegated Investigator staff and Sub-investigator, in accordance with consensus ethics principles derived from international ethics guidelines, including the Declaration of Helsinki, and the ICH guidelines for good clinical practice (GCP), all applicable laws, rules and regulations.

11.1. Ethics Committees and Regulatory Authorities

The ICH-GCP (E6 (R2)) guidelines require that approval must be obtained from an Independent Ethics Committee (IEC) prior to participation of human subjects in research studies. Prior to the study onset, the protocol, informed consent, advertisements to be used for subject recruitment, and any other written information regarding this study to be provided to the subject or the subject's legal guardian must be approved by the IEC. Documentation of the relevant national IEC approval and of the IEC compliance with ICH Guideline E6 will be maintained by the site and will be available for review by the Sponsor or its designee or by the authorized members of regulatory agencies.

The respective Ethics Committees approvals should be signed by the IEC Chairman or designee and must identify the IEC name and address, the clinical protocol by title and/or protocol number and the date approval and/or favorable opinion was granted. Also, a list of the EC members who attended the meeting when the Protocol/ Protocol amendment was discussed, including names and qualifications, needs to be provided by the EC to the investigator or the sponsor/ his representative.

If any alterations, other than changes of administrative nature only, are made to the study protocol, a formal protocol amendment will be issued and submitted to relevant IEC for approval. The amendment will not be implemented until IEC approval, except in cases where immediate implementation is necessary to eliminate or prevent imminent hazard to the subjects.

In the same way, approval from regulatory authorities (RA) should be granted before beginning the study. The investigator or the sponsor representative must provide to the regulatory authorities the name and address of the EC along with a statement from the EC that it is organized according to GCP and the applicable laws and regulations. Amendments will be submitted to RA too for approval.

CONFIDENTIAL

11.2. Responsibilities of the Sponsor

The Sponsor of this clinical trial is responsible to regulatory authorities for taking all reasonable steps to ensure the proper conduct of the clinical trial as regards ethics, clinical trial protocol compliance, and integrity and validity of the data recorded on the e-CRFs. Thus, the main duty of the monitoring team is to help the Investigator and the Sponsor maintain a high level of ethical, scientific, technical and regulatory quality in all aspects of the clinical trial.

At regular intervals during the clinical trial, the site will be contacted, through monitoring visits, letters or telephone calls, by a representative of the monitoring team to review study progress, Investigator and subject compliance with clinical trial protocol requirements and any emergent problems. These monitoring visits will include but not be limited to review of the following aspects: subject informed consent, subject recruitment and follow-up, SAE documentation and reporting, AE documentation, IP allocation, subject compliance with the instructions, IP accountability, concomitant therapy use, and quality of data.

11.3. Responsibilities of the Investigator

The Investigator is responsible for ensuring that the clinical study is performed in

accordance with the protocol, the ethical principles that have their origin in the Declaration of Helsinki (version as of October 2013) as well as with the ICH Note for Guidance on Good Clinical Practice (ICH, Topic E6, R2, 2016), relevant site SOPs and applicable regulatory requirements. These documents state that the informed consent of subjects is an essential precondition for participation in the clinical study.

The Investigator is required to ensure compliance with all procedures required by the clinical trial protocol and with all study procedures provided by the Sponsor (including security rules). The Investigator agrees to provide reliable data and all information requested by the clinical trial protocol (with the help of the e-CRF, Discrepancy Resolution Form [DRF] or other appropriate instrument) in an accurate and legible manner according to the instructions provided and to ensure direct access to source documents by Sponsor representatives.

If any circuit includes transfer of data particular attention should be paid to the confidentiality of the subject's data to be transferred.

The Investigator may appoint such other individuals as he/she may deem appropriate as Sub-investigators to assist in the conduct of the clinical trial in accordance with the clinical trial protocol. All Sub-investigators shall be appointed and listed in a timely manner. The Sub-investigators will be supervised by and work under the responsibility of the Investigator. The Investigator will provide them with a copy of the clinical trial protocol and all necessary information.

CONFIDENTIAL

In 1998, the US Food and Drug Administration (FDA) introduced a regulation (21 CFR, Part 54) entitled “Financial Disclosure by Clinical Investigators.” For studies conducted in any country that could result in a product submission to the FDA for marketing approval and which contribute significantly to the demonstration of efficacy and safety of the drug (named “covered studies” by the FDA), the Investigator and all sub-Investigators are obliged to disclose their financial interest which they or their spouses and dependent children may have in the Sponsor. This information is required during the study and until 12 months after its completion.

11.4. Subject Information and Consent

A prerequisite for a subject participating in the study is his/her or their legal representative or parent’s/guardian’s written informed consent. Adequate information must therefore be given to the subject by the Investigator/ designated personnel before informed

consent is obtained. One or more Informed Consent Forms in the local language and prepared in accordance with the Note for Guidance on Good Clinical Practice (ICH, Topic E6 (R2)) will be provided by the Sponsor for the purpose of obtaining informed consent. In addition to this written information, the Investigator or his designate will inform the subject verbally. In doing so, the wording used will be chosen so that the information can be fully and readily understood by laypersons. The Informed Consent Form will be revised whenever important new information becomes available that may be relevant to the consent of subjects.

The subject must be informed that his/her personal trial-related data will be used by CNBG and its subsidiaries (sponsor) in accordance with the local data protection law. The level of disclosure must also be explained to the subject.

The subject must be informed that his/ her medical records may be examined by authorized monitors or Clinical Quality Assurance auditors appointed by CNBG and its subsidiaries (sponsor), by appropriate IRB / IEC members, and by inspectors from regulatory authorities.

CONFIDENTIAL

The Informed Consent Form must also be signed and personally dated by the subject and legal representative or parents/guardian and by the Investigator/person designated by the Investigator to conduct the informed consent discussion. Provision of consent will be confirmed in the eCRF by the Investigator. The signed and dated declaration of informed consent will remain at the Investigator's site and must be safely archived by the Investigator so that the forms can be retrieved at any time for monitoring, auditing and inspection purposes. A copy of the signed and dated information and consent should be provided to the subject prior to participation.

11.5. Compensation to Subjects

Appropriate insurance coverage is provided by China National Biotec Group (CNBG) in line with legal requirements and GCP guidance. Details can be asked at the investigator’s site (certificates and conditions in the Investigator Site File).

Insurance coverage will be provided by a local insurance provider.

11.6. Subject Confidentiality

The investigator(s) will respect and protect the confidentiality of the subject in all possible ways. Subject identification, other than subject number, initials and date of birth, will not appear in any Case Report Form (eCRF) pages or other documents given to the Sponsor. Only the investigator and the persons authorized to verify the quality and integrity

of the study will have access to subject records where the subject can be identified.

Ensure that the personal secrets of the subjects will not be disclosed under the conditions of testing, biological sample collection, reporting and publication, etc. Only the subject code, blood sample number, blood collection time and test index are recorded in the blood sample. It is strictly limited to the core testing personnel to obtain electronic or written copies.

11.7. Amendment to Subject Related Information

Should a Protocol amendment become necessary, the subject information and consent form may need to be revised to reflect the changes to the Protocol. It is the responsibility of the investigator to ensure that an amended consent form is reviewed and has received approval/ favorable opinion from the IRB / IEC and CA and/or the regulatory authority has provided approval / has been notified (depending on local laws and regulations), and that it is signed by all subjects subsequently entered in the trial and those currently in the trial, if affected by the amendment.

CONFIDENTIAL

11.8. Direct Access to Source Documentation

Source data are all the information in original records and certified copies of original records of clinical findings, observations, or other activities in the study, which are necessary for the reconstruction and evaluation of the study. Source data are contained in source documents (originals or certified copies).

Source Documents are original documents, data, and records (e.g., clinical and office charts, laboratory notes, memoranda, subjects' diaries, pharmacy dispensing records, recorded data from automated instruments, copies or transcriptions certified after verification as being accurate and complete, subject files, and pharmacy records or prescriptions, laboratory reports or, computer printouts from the laboratory websites, vaccine handover sheet and delivery notes). All of them are expected to be reviewed signed, dated and assessed by the investigator. The source documents must contain study participation information.

It is the Investigator’s obligation to collect and present all relevant medical data in the subject’s medical file. Sponsor name and trial number, subject identification (name, date of birth, address, etc.), information that the informed consent was obtained prior any screening procedures, visit dates, subject number, vaccine information, efficacy data, safety data, concomitant medications and date and reason of completion of the study.

Source records should be preserved for the maximum period of time required by local requirements.

All information recorded on the eCRFs for this study must be consistent with the subject's source documentation.

Besides the monitor of the Contract Research Organization (CRO), regulatory authorities, members of ethics committees and the Sponsor's clinical quality assurance group or any other Sponsor's representative, may carry out source data checks and/or on-site audits or inspections. Direct access to source data will be required for these audits and inspections; they will be carried out giving due consideration to data protection and medical confidentiality. The investigator will assure the CRO and the sponsor of the necessary support at all times.

12. STUDY MANAGEMENT

12.1. Case Report Form (eCRF) Handling

The data recorded during the course of this study will be documented in the form of eCRF, and must be forwarded to the sponsor or appointed designee. Then they should be processed, evaluated and stored in anonymous form in accordance with data protection regulations.

The investigator must ensure that the eCRFs and any other associated documents contain no mention of any subject names and other privacy information. The eCRFs must be completely filled in. They are regulatory documents and must be suitable for submission to authorities.

All data in the eCRFs must be derived from source documentations. Clinical data will be captured via local medical system, the data will be transmitted to CTMS and generate eCRF. The investigator site staff will enter and edit the data via a secure network, with secure access features (username, password and secure identification or username and password – an electronic password system). A complete electronic audit trail will be maintained. The investigator will approve the data using an electronic signature (Ref: 21 CFR Part 11), and this approval is used to confirm the accuracy of the data recorded. The electronic CRFs (eCRFs) will be used, the investigator's data will be accessible from the investigator's site throughout the trial. Relevant medical history prior to enrolment will be documented at the baseline visit. Thereafter during the trial, narrative statements relative to the subject's progress during the trial will be maintained. The electronic CRFs must be kept current to reflect subject status at each phase during the course of the trial. The subjects

must not be identified on the electronic CRF by name. Appropriate coded identification (i.e. Subject Number) must be used. The investigator must make a separate confidential record of these details (subject identification code list) to permit identification of all subjects enrolled in a clinical trial in case follow-up is required. While a trial is ongoing and until the access to the database has been terminated, there will be no Documentation of Changes (DOCs).

12.2. Source Data and Subject Files

Source documents provide evidence for the existence of the subject and substantiate the integrity of the data collected. Source documents are filed at the investigator's site.

The investigator has to keep a written or electronic subject file for every subject participating in the clinical study. In this subject file, the available demographic and medical information of a subject has to be documented, in particular the following: name, date of birth, sex, height, weight, statement of entry into the study, study identification, randomization number, the date of informed consent, all study visit dates, predefined performed examinations and clinical findings, observed AEs, and reason for withdrawal from the study, if applicable.

CONFIDENTIAL

It should be possible to verify the inclusion and exclusion criteria defined in the protocol from the available data in this file.

It must be possible to identify each subject by using this subject file. Additionally, any other documents with source data, especially original printouts of data that were generated by technical equipment have to be filed. This includes but not limited to ECG tracings, X-ray films, CT and MRI scans, laboratory value listings and QoL questionnaires, etc. All these documents have to bear at least the subject identifier and the printing date printed by the recording device to indicate to which subject and to which study procedure the document belongs. The medical evaluation of such records should be documented as necessary and signed/dated by the investigator.

Computerized subject files will be printed whenever source data verification is performed by the monitor. Printouts must be signed and dated by the investigator, countersigned by the monitor and kept in a safe place.

Data entered in the eCRFs that are transcribed from source documents must be consistent with the source documents or the discrepancies must be explained. The investigator may need to request previous medical records or transfer records, depending on the case; also, current medical records must be available.

Data on the subject dairies are considered source data and have to be stored along with

the subject file. The data of the dairies will be forwarded to the sponsor or a CRO appointed by the sponsor for data entry into the data base.

12.3. Investigator Site File and Archiving

The Investigator will be provided with an Investigator Site File at the start of the study. This file contains all relevant documents necessary for the conduct of the study. This file must be safely archived after termination of the study. It is the responsibility of the Investigator to ensure that the files are stored for at least 15 years beyond the end of the clinical study. All original subject files must be stored for the longest possible time permitted by the regulations at the hospital, research institute, or practice in question. If archiving can no longer be maintained at the site, the Investigator should notify the Sponsor.

12.4. Monitoring, Quality Assurance and Inspection by Authorities

This study is to be conducted in accordance with the ICH Note for Guidance on Good Clinical Practice (ICH, Topic E6 (R2), 2016). The appointed clinical monitor will arrange regular visits to the study center(s) to check progress with the study and to check eCRF completion.

During monitoring visits, the monitors will:

CONFIDENTIAL

- Help resolve any problems;
- Examine eCRF for omission of data, compliance and possible AEs;
- Discuss inconsistencies in the study data;
- Ensure that all study materials are correctly stored and dispensed;
- Check adherence to the obligations of the investigator;
- Review consent forms, in particular the date of consent and signature;
- Perform source data verification as described below.

In line with International Conference on Harmonization (ICH)-Good Clinical Practice (GCP) guidelines, monitoring will include verification of data entered in the eCRF against original subject records. This verification will be performed by direct access to the original subject records, and the Sponsor guarantees that subject confidentiality will be respected at all times. Participation in this study will be taken as agreement to permit direct source data verification.

The investigator / institution will permit trial-related monitoring, audits, IRB / IEC review and regulatory inspection, providing direct access to all related source data / documents. eCRFs and all source documents, including progress notes and copies of laboratory and medical test results must be available at all times for review by the sponsor's clinical trial monitor or a CRO appointed by the sponsor, auditor and inspection by health

authorities (e.g. FDA). The Clinical Research Associate (CRA) / on site monitor and auditor may review all CRFs/eCRFs, and written informed consents.

12.5. Changes to the Study Protocol

Changes to, or formal clarifications of, the study protocol must be documented in writing.

Major changes to the protocol will be described in a "Protocol Amendment". It will be submitted to the relevant Ethics Committee(s)/Institutional Review Board(s) and to authorities, where required. Approval/favorable opinion from the relevant Ethics Committee(s)/Institutional Review Board(s) will be required prior to implementation of the amendment.

Any amendment affecting the subject requires the subject's informed consent prior to implementation.

Changes of administrative or technical nature will be recorded in a document entitled "Administrative Change to Study Protocol". It will be sent for information to the relevant Ethics Committee(s)/ Institutional Review Board(s) or to authorities, if so required. Amendments and administrative changes will be signed by all signatories of the protocol.

All Investigators will acknowledge the receipt and confirm by their signature on the Amendment or Administrative Change Signature Sheet that they will adhere to the Amendment/Administrative Change. This sheet will be issued in duplicate and after signing, one will be filed in the Investigator Site File and one in the Study Master File.

12.6. Study Report and Publication Policy

After conclusion of the study, an integrated clinical and statistical study report shall be written by the Sponsor.

The respective EC and competent authority need to be notified about the end of the trial (last subject/subject out) or early termination of the trial.

China National Biotec Group (CNBG) is as much as possible dedicated to support process of free exchange of relevant scientific information. Any publication of the result of this trial must be consistent with the CNBG publication policy and regulations of the relevant regulatory authority. The rights of the investigator and of the sponsor with regard to publication of the results of this trial are described in the investigator contract. As a general rule, no trial results should be published prior to finalization of the Clinical Trial Report (CTR) and obtaining authorization from the relevant regulatory authority.

The present trial will be published in a clinical trial registry indicating the trial dates and indication as well as the number of sites and location. The subject identity should be

kept confidential.

CONFIDENTIAL

13. REFERENCES

[1] Vaccine Administration Law of the People's Republic of China, National People's Congress of the People's Republic of China, 1 December, 2019

[2] Good Clinical Practice (GCP), NMPA, 6 August, 2003

[3] Technical Guidelines for Clinical Trials of Vaccines, NMPA, 3 December 2004

[4] Technical Guiding Principles for Clinical Research of Prophylactic COVID-19 Vaccines (Tentative), NMPA, 14 August 2020

[5] Guidelines for Writing Basic Contents of Vaccine Clinical Study Reports, NMPA, 12 October 2005

[6] Guiding Principles of Adverse Event Grading Standards for Clinical Trials of Vaccines for Prevention, NMPA, 31 December 2019. https://www.nmpa.gov.cn/xxgk/ggtg/qtggtg/20191231111901460.html

[7] Measures for the Administration of Drug Registration (Bureau Order No.27): State Administration of Market Supervision, 30 March 2020

[8] Guidelines for Ethical Review of Drug Clinical Trials, NMPA, 2 November 2010

[9] Guidelines for the Management of Biological Sample Analysis Laboratories in Drug Clinical Trials (Tentative) NMPA, 2011

[10] Guiding Principles for Quality Management of Vaccine Clinical Trials (Tentative), NMPA, 31 October 2013

[11] Regulations on the Administration of Drug Clinical Trial Institutions, NMPA, National Health Commission, 1 December 2019

[12] Regulations on the Management of Serious Adverse Event Reporting in Vaccine Clinical Trials (Tentative), NMPA, 17 January 2014

[13] Guidelines on Biostatistics for Drug Clinical Trials, NMPA, 1 June 2016

[14] Guidelines for Planning and Reporting Data Management and Statistical Analysis of Drug Clinical Trials, NMPA, 27 July 2016

[15] Standards and Procedures for Rapid Reporting of Safety Data during Drug Clinical Trials, NMPA, 27 April 2018

[16] ICH E6 (R2), Integrated Addendum to Good Clinical Practice, dated 9 November 2016

[17] ICH E3, Structure and Content of Clinical Summary Report, 30 November 1995

[18] ICH E9, Statistical Principles of Clinical Trials, 5 Feb 1998

[19] "Identification of Abnormal Reaction to Vaccination", National Health

Commission, 1 Dec 2008

[20] Good Practice for Vaccination, National Health Commission, 6 December 2016

[21] Good Practice for the Management of Vaccine Storage and Transportation (2017 Edition), National Health Commission, 15 December 2017

[22] Al Kaabi N, Zhang Y, Xia S. Effect of 2 Inactivated SARS-CoV-2 Vaccines on Symptomatic COVID-19 Infection in Adults: A Randomized Clinical Trial. JAMA. 2021 May 26. doi: 10.1001/jama.2021.8565. Epub ahead of print. PMID: 34037666.

[23] The Development Safety Update Report (DSUR): Harmonizing the Format andContent for Periodic Safety Reporting During Clinical Trials: Report of CIOMS Working Group VII, Geneva 2007. https://cioms.ch/shop/product/development-safety-update-report-dsur-harmonizing-format-contentperiodic-safety-report-clinical-trials-report-cioms-working-group-vii.

[24] Priority List of Adverse Events of Special Interest: COVID-19. SPEAC. 25 May 2020. https://brightoncollaboration.us/wpcontent/uploads/2020/06/SPEAC_D2.3_V2.0_COVID-19_20200525_public.pdf.

CONFIDENTIAL

[25] Coronavirus disease (COVID-2019) situation reports. WHO. https://www.who.int/emergencies/diseases/novel-coronavirus-2019/situation-reports/

[26] Draft landscape and tracker of COVID-19 candidate vaccines. https://www.who.int/publications/m/item/draft-landscape-of-covid-19-candidate-vaccines

[27] Latest Situation On COVID-19 as of 24:00 on Sep 19th. China NHC website. http://www.nhc.gov.cn/xcs/yqtb/202109/110e53f7872c4d6b96cf0be8f9d4faa2.shtml

[28] Guan WJ, Ni ZY, Hu Y, et al. Clinical characteristics of 2019 novel coronavirus infection in China. medRxiv. Published February 9, 2020. Accessed February 23, 2020. https://www.medrxiv.org/content/10.1101/2020.02.06.20020974v1

[29] Zhou, P. et al. A pneumonia outbreak associated with a new coronavirus of probable bat origin. Nature https:// doi.org/10.1038/s41586-020-2012-7 (2020).

[30] Wrapp D, Wang NW, Corbett KS, et al. Cryo-EM Structure of the 2019-nCoV Spike in the Prefusion Conformation. bioRxiv. Published February 15, 2020. Accessed February 23, 2020.

[31] Zhang Y, Zeng G, Pan H, et al. Safety, tolerability, and immunogenicity of an

inactivated SARS-CoV-2 vaccine in healthy adults aged 18-59 years: a randomised, double-blind, placebo-controlled, phase 1/2 clinical trial. Lancet Infect Dis. 2021 Feb;21(2):181-192. doi: 10.1016/S1473-3099(20)30843-4.

[32] Novavax COVID-19 Vaccine Demonstrates 89.3% Efficacy in UK Phase 3 Trial. https://ir.novavax.com/news-releases/news-release-details/novavax-covid-19-vaccine-demonstrates-893-efficacy-uk-phase-3

[33] Shilong Yang, Yan Li, Lianpan Dai, et al. Safety and immunogenicity of a recombinant tandem-repeat dimeric RBD protein vaccine against COVID-19 in adults: pooled analysis of two randomized, double-blind, placebo-controlled, phase 1 and 2 trials. medRxiv 2020.12.20.20248602; doi: https://doi.org/10.1101/2020.12.20.20248602

[34] EMA recommends COVID-19 Vaccine AstraZeneca for authorisation in the EU. https://www.ema.europa.eu/en/news/ema-recommends-covid-19-vaccine-astrazeneca-authorisation-eu

[35] Voysey M, Clemens SAC, Madhi SA, et al. Safety and efficacy of the ChAdOx1 nCoV-19 vaccine (AZD1222) against SARS-CoV-2: an interim analysis of four randomised controlled trials in Brazil, South Africa, and the UK. Lancet. 2021, 397(10269):99-111. doi: 10.1016/S0140-6736(20)32661-1.

CONFIDENTIAL

[36] Zhu FC, et al. Immunogenicity and safety of a recombinant adenovirus type-5-vectored COVID-19 vaccine in healthy adults aged 18 years or older: a randomised, double-blind, placebo-controlled, phase 2 trial. Lancet. 2020, 396(10249):479-488. doi: 10.1016/S0140-6736(20)31605-6.

[37] Baden LR, El Sahly HM, Essink B,et al. Efficacy and Safety of the mRNA-1273 SARS-CoV-2 Vaccine. N Engl J Med.2020 Dec 30:NEJMoa2035389. doi: 10.1056/NEJMoa2035389. Epub ahead of print. PMID: 33378609

[38] Polack FP, Thomas SJ, Kitchin N, et al. Safety and Efficacy of the BNT162b2 mRNA Covid-19 Vaccine. N Engl J Med. 2020,383(27):2603-2615. doi: 10.1056/NEJMoa2034577.

[39] Technical Guidelines for Inoculation of SARS-COV-2 Vaccine (First Edition). National Health Commission.

Appendix

Case Definition

Suspected cases:

Comprehensive judgment based on epidemiological history and clinical symptoms:

Have any of the epidemiological history, and have two or more A symptoms, or have one or more B symptoms; or

with imaging features of COVID-19

If there is no clear epidemiological history, they should have two or more A symptoms or one or more B symptoms and detectable SARS-CoV-2 specific IgM; or have two or more A symptoms and One or more B symptoms; with imaging features of COVID-19

① Epidemiological history

A. Long-term residence or stay in the affected area for more than 7 days is deemed to have an epidemiological history; or

B. History of travel or residence in the community where the case was reported within 14 days before the onset of illness; or

CONFIDENTIAL

C. In contact with SARS-CoV-2 infected or asymptomatic infected persons within 14 days before the onset; or

D. Cluster cases (2 or more cases of fever and/or respiratory symptoms occurred in a small area such as home, office, school, etc. within 2 weeks).

② Clinical symptoms

Symptoms A (last for at least 2 days): fever (axillary temperature ≥37.5℃); chills; sore throat; fatigue; nasal congestion or runny nose; body pain, muscle pain; headache; nausea or vomiting; diarrhea.

Symptoms B: Cough (last for at least 2 days); newly developed taste or smell disorders (last for at least 2 days); shortness of breath or difficulty breathing;

③ Imaging features of COVID-19

Confirmed cases:

On the basis of the determination of the suspected case, there’s also a COVID-19 PCR test result.

Differential diagnosis:

Encourage any possible pathogenic differential diagnosis.

Clinical Classifications of Confirmed COVID-19 Cases:

Mild

The clinical symptoms are mild, and there is no imaging characteristics of pneumonia.

Moderate

Showing fever, respiratory symptoms, and imaging characteristics of pneumonia.

Severe

Meet any of the following criteria:

1. Respiratory distress (RR≥30 breaths/min);
2. Oxygen saturatio n≤93% at rest;
3. Arterial partial pressure of oxygen (PaO2)/ fraction of inspired oxygen (FiO2)≦300mmHg (1mmHg=0.133kPa);
4. The clinical symptoms progressively worsened, and the chest imaging showed >50% obvious lesion progression within 24-48 hours.

CONFIDENTIAL

Critical

Meet one of the following criteria:

1. Respiratory failure and requiring mechanical ventilation;
2. Shock;
3. With other organ failure that requires ICU care;
4. Death

CONSORT 2010 checklist of information to include when reporting a randomised trial*

Section/Topic	Item No	Checklist item	Reported on page No
**Title and abstract**			
	1a	Identification as a randomised trial in the title	Page 1
	1b	Structured summary of trial design, methods, results, and conclusions (for specific guidance see CONSORT for abstracts)	Page 2
**Introduction**			
Background and objectives	2a	Scientific background and explanation of rationale	Pages 3, 4
	2b	Specific objectives or hypotheses	Page 4
**Methods**			
Trial design	3a	Description of trial design (such as parallel, factorial) including allocation ratio	Pages 14,16
	3b	Important changes to methods after trial commencement (such as eligibility criteria), with reasons	NA
Participants	4a	Eligibility criteria for participants	Page 15
	4b	Settings and locations where the data were collected	Page 17
Interventions	5	The interventions for each group with sufficient details to allow replication, including how and when they were actually administered	Page 17
Outcomes	6a	Completely defined pre-specified primary and secondary outcome measures, including how and when they were assessed	Page 17,18
	6b	Any changes to trial outcomes after the trial commenced, with reasons	NA
Sample size	7a	How sample size was determined	Page 19,20
	7b	When applicable, explanation of any interim analyses and stopping guidelines	NA
Randomisation:			
Sequence generation	8a	Method used to generate the random allocation sequence	Page 16
	8b	Type of randomisation; details of any restriction (such as blocking and block size)	Page 16
Allocation concealment mechanism	9	Mechanism used to implement the random allocation sequence (such as sequentially numbered containers), describing any steps taken to conceal the sequence until interventions were assigned	Page 16
Implementation	10	Who generated the random allocation sequence, who enrolled participants, and who assigned participants to interventions	Page 16
Blinding	11a	If done, who was blinded after assignment to interventions (for example, participants, care providers, those	Page 16

		assessing outcomes) and how	
	11b	If relevant, description of the similarity of interventions	Page 16
Statistical methods	12a	Statistical methods used to compare groups for primary and secondary outcomes	Pages 19, 20
	12b	Methods for additional analyses, such as subgroup analyses and adjusted analyses	Pages 19, 20
**Results**			
Participant flow (a diagram is strongly recommended)	13a	For each group, the numbers of participants who were randomly assigned, received intended treatment, and were analysed for the primary outcome	Page 5
	13b	For each group, losses and exclusions after randomisation, together with reasons	Figure 1
Recruitment	14a	Dates defining the periods of recruitment and follow-up	Pages 4, 14 and 17
	14b	Why the trial ended or was stopped	NA
Baseline data	15	A table showing baseline demographic and clinical characteristics for each group	Table 1
Numbers analysed	16	For each group, number of participants (denominator) included in each analysis and whether the analysis was by original assigned groups	Page 5 Figure 1
Outcomes and estimation	17a	For each primary and secondary outcome, results for each group, and the estimated effect size and its precision (such as 95% confidence interval)	Pages 5-11
	17b	For binary outcomes, presentation of both absolute and relative effect sizes is recommended	NA
Ancillary analyses	18	Results of any other analyses performed, including subgroup analyses and adjusted analyses, distinguishing pre-specified from exploratory	Pages 10-11
Harms	19	All important harms or unintended effects in each group (for specific guidance see CONSORT for harms)	NA
**Discussion**			
Limitations	20	Trial limitations, addressing sources of potential bias, imprecision, and, if relevant, multiplicity of analyses	Pages 14
Generalisability	21	Generalisability (external validity, applicability) of the trial findings	Pages 11-14
Interpretation	22	Interpretation consistent with results, balancing benefits and harms, and considering other relevant evidence	Pages 11-14
**Other information**			
Registration	23	Registration number and name of trial registry	Pages 2, 14
Protocol	24	Where the full trial protocol can be accessed, if available	Page 15
Funding	25	Sources of funding and other support (such as supply of drugs), role of funders	Pages 15, 24

*We strongly recommend reading this statement in conjunction with the CONSORT 2010 Explanation and Elaboration for important clarifications on all the items. If relevant, we also recommend reading CONSORT extensions for cluster randomised trials, non-inferiority and equivalence trials, non-pharmacological treatments, herbal interventions, and pragmatic trials. Additional extensions are forthcoming: for those and for up to date references relevant to this checklist, see www.consort-statement.org.
