# Supplementary material for: Safety and immunogenicity of a hybrid-type vaccine booster in BBIBP-CorV recipients in a randomized phase 2 trial
Source: Nat Commun. 2022 Jun 27;13:3654. doi: 10.1038/s41467-022-31379-0 (PMC9237056; doi:10.1038/s41467-022-31379-0)
Supplement: Supplementary file 3 — Reporting Summary [file 41467_2022_31379_MOESM3_ESM.pdf]

nature portfolio

Corresponding author(s): Yun Tao Zhang; Xiao Ming Yang; Guizhen Wu; Qi Ming Li

Last updated by author(s): May 23, 2022

Reporting Summary

Nature Portfolio wishes to improve the reproducibility of the work that we publish. This form provides structure for consistency and transparency in reporting. For further information on Nature Portfolio policies, see our Editorial Policies and the Editorial Policy Checklist.

Statistics

For all statistical analyses, confirm that the following items are present in the figure legend, table legend, main text, or Methods section.

n/a	Confirmed	
☐	☒	The exact sample size (*n*) for each experimental group/condition, given as a discrete number and unit of measurement
☐	☒	A statement on whether measurements were taken from distinct samples or whether the same sample was measured repeatedly
☐	☒	The statistical test(s) used AND whether they are one- or two-sided<br>*Only common tests should be described solely by name; describe more complex techniques in the Methods section.*
☒	☐	A description of all covariates tested
☐	☒	A description of any assumptions or corrections, such as tests of normality and adjustment for multiple comparisons
☐	☒	A full description of the statistical parameters including central tendency (e.g. means) or other basic estimates (e.g. regression coefficient) AND variation (e.g. standard deviation) or associated estimates of uncertainty (e.g. confidence intervals)
☐	☒	For null hypothesis testing, the test statistic (e.g. *F*, *t*, *r*) with confidence intervals, effect sizes, degrees of freedom and *P* value noted<br>*Give P values as exact values whenever suitable.*
☒	☐	For Bayesian analysis, information on the choice of priors and Markov chain Monte Carlo settings
☐	☒	For hierarchical and complex designs, identification of the appropriate level for tests and full reporting of outcomes
☒	☐	Estimates of effect sizes (e.g. Cohen's *d*, Pearson's *r*), indicating how they were calculated

Our web collection on statistics for biologists contains articles on many of the points above.

Software and code

Policy information about availability of computer code

Data collection	Data collected in the electronic case report form, and carried out by using Clinical Trial Data Management System.
Data analysis	All statistical analyses were carried out using SAS software (version 9.4). Sample size was calculated by using Power Analysis and Sample Size (PASS 15.0) software. No custom codes were developed in the study.

For manuscripts utilizing custom algorithms or software that are central to the research but not yet described in published literature, software must be made available to editors and reviewers. We strongly encourage code deposition in a community repository (e.g. GitHub). See the Nature Portfolio guidelines for submitting code & software for further information.

Data

Policy information about availability of data

All manuscripts must include a data availability statement. This statement should provide the following information, where applicable:

- Accession codes, unique identifiers, or web links for publicly available datasets
- A description of any restrictions on data availability
- For clinical datasets or third party data, please ensure that the statement adheres to our policy

The study protocol is available in the Supplementary Information file. The individual participant data will be shared after de-identification. The clinical trial is still ongoing, and the data will be available from one month to one year after the completion of the study. Researchers who provide a scientifically sound proposal will be allowed access to the individual participant data. Proposals should be directed to the corresponding author Qi Ming Li (liqiming189@163.com). The proposals will be reviewed and approved by the funder, investigator, and collaborators on the basis of scientific merit. To gain access, data requestors will need to sign a data access agreement.

Field-specific reporting

Please select the one below that is the best fit for your research. If you are not sure, read the appropriate sections before making your selection.

☒ Life sciences ☐ Behavioural & social sciences ☐ Ecological, evolutionary & environmental sciences

For a reference copy of the document with all sections, see nature.com/documents/nr-reporting-summary-flat.pdf

Life sciences study design

All studies must disclose on these points even when the disclosure is negative.

Sample size	The sample size of participants was determined by Power Analysis and Sample Size (PASS15.0) software using the expected difference between groups, predefined noninferiority margin, intended power, significance level and estimated dropout rate. Assuming that the 4-fold rise rate after booster vaccination achieve 85%, 208 participants in each arm will be required to have 80% power to conclude non-inferiority with margin of -10% and one-sided significance level of 2.5% using Miettinen and Nurminen method. If equal GMT after booster immunization is assumed, and standard deviation of GMT after log10 transformation is considered to be 0.7, 250 subjects in each arm will be required to have 80% power to conclude non-inferiority with margin of 2/3 and one-sided significance level of 2.5%. Then, considering about 15%~20% drop-out rate, 600 participants are required in each of the three groups (4-6-month, 7-9-month and >9-month groups), and 1800 subjects in total are planned to enroll.
Data exclusions	No data were excluded from the analysis.
Replication	This paper reported the interim results of a clinical trial, and there was no attempt at replication of the study findings.
Randomization	For allocation of the participants, a randomization list was created by the stratified blocked randomization method using SAS software (version 9.4). Eligible participants were stratified according to the prime-boost intervals, i.e., 4-6 months, 7-9 months and >9 months. In each stratum, participants were randomly assigned in a ratio of 1:1 to either heterologous or homologous booster groups using a block randomization method with a block size of 10. A vaccine randomization list was also generated using SAS software with a randomization block size of 10. Then, participant and vaccine randomization lists were inputted into the interactive web response system (IWRS), and participants were vaccinated according to the randomization number and vaccine number obtained from IWRS.
Blinding	The trial was double-blind to avoid introducing bias by having randomization and masking process conducted by independent personnel who were not involved in the study. Participants, investigators, and other staff remained blinded to individual treatment assignments during the trial.

Reporting for specific materials, systems and methods

We require information from authors about some types of materials, experimental systems and methods used in many studies. Here, indicate whether each material, system or method listed is relevant to your study. If you are not sure if a list item applies to your research, read the appropriate section before selecting a response.

Materials & experimental systems

n/a	Involved in the study	
☒	☐	Antibodies
☐	☒	Eukaryotic cell lines
☒	☐	Palaeontology and archaeology
☒	☐	Animals and other organisms
☐	☒	Human research participants
☐	☒	Clinical data
☒	☐	Dual use research of concern

Methods

n/a	Involved in the study	
☒	☐	ChIP-seq
☒	☐	Flow cytometry
☒	☐	MRI-based neuroimaging

Eukaryotic cell lines

Policy information about cell lines

Cell line source(s)	Vero cells from National Institute for Food and Drug Control (NIFDC), Beijing, China
Authentication	The Vero cell line was not authenticated.
Mycoplasma contamination	The Vero cell line has been tested negative for mycoplasma contamination.
Commonly misidentified lines (See ICLAC register)	No commonly misidentified cell lines were used in the study.

March 2021

Human research participants

Policy information about studies involving human research participants

Population characteristics	The demographic characteristics were broadly similar between heterologous and homologous boosting groups (Table 1 and Supplementary Table 1). The participants in two groups had similar age, sex, race, height and weight distributions. The majority of the participants were Bangladeshis, Indian or Pakistanis, and more men than women participated in the trial. The baseline antibody levels were statistically similar between the participants in heterologous and homologous boosting groups.
Recruitment	Three groups of healthy adults aged ≥18 years who had received two doses of BBIBP-CorV 4-6 months, 7-9 months and >9 months before, respectively, were recruited from the United Arab Emirates. The subjects were recruited by telephone or participated in the trial by themselves. At the research site, the subjects were given an informed consent, and were well told of the study protocol, including study purpose, procedure, duration, risks and benefits, etc. Under the condition of voluntary participation, the subjects and research doctors signed an informed consent form together. Written informed consent was obtained from all participants before the screening. Participants were enrolled after undergoing a health screening by inquiry, medical history review and physical examination. Confirmed, suspected or asymptomatic COVID-19 cases, individuals with a history of SARS or MERS infections, and those vaccinated with any other COVID-19 vaccines were excluded. The detailed inclusion and exclusion criteria are are listed in the study protocol (Supplementary Note 3). Eligible participants were stratified according to the prime-boost intervals, i.e., 4-6 months, 7-9 months and >9 months. In each stratum, participants were randomly assigned in a ratio of 1:1 to either heterologous or homologous booster groups using a block randomization method with a block size of 10. During the study, double-blind RCT method was adopted to avoid the influence of self-selection bias or other selection biases on the results.
Ethics oversight	The trial protocol was reviewed and approved by Abu Dhabi Health Research and Technology Ethics Committee.

Note that full information on the approval of the study protocol must also be provided in the manuscript.

Clinical data

Policy information about clinical studies

All manuscripts should comply with the ICMJE guidelines for publication of clinical research and a completed CONSORT checklist must be included with all submissions.

Clinical trial registration	This trial was registered with ClinicalTrials.gov (NCT05069129).
Study protocol	The protocol has been submitted and provided in the Supplementary Information file of the paper.
Data collection	Between Oct 23 and Nov 8, 2021, we recruited 1833 participants, and these participants were classified into three groups with different prime-boosting intervals, i.e., 4-6 months, 7-9 months and >9 months. For each group, participants were randomly assigned to receive either a heterologous boost of NVSI-06-08 or a homologous boost of BBIBP-CorV. After booster vaccination, participants were observed at study site for 30 min to identify immediate adverse reactions. Solicited adverse events (AEs) were recorded for 7 days and unsolicited AEs for 30 days post-vaccination. Serious adverse events (SAEs) and adverse events of special interest (AESIs) were collected up to 6 months after booster immunization. Nasopharyngeal swabs were collected from all the participants for PCR tests prior to booster vaccination. After the vaccination, only for the subjects who showed any symptoms of COVID-19 or went to hospital for treatment, nasopharyngeal swabs were collected and PCR tests were conducted. Blood samples were collected before booster vaccination, and on 15 days, 30 days, 3 months, 6 months, 9 months and 12 months post-boost. Vaccination and blood collection were conducted at SKMC Center for Diabetes & Endocrinology, Abu Dhabi, UAE. The data were collected in accordance with local regulations and ICH-GCP relevant standards.
Outcomes	The primary immunogenicity outcome was the neutralizing response on 15 days and 30 days after booster vaccination, by evaluation of the geometric mean titers (GMTs) of neutralizing antibodies and the corresponding 4-fold rise rate (i.e., post-/pre-boost ≥4) against SARS-CoV-2 prototype strain. Neutralizing antibody titers were measured using live-virus neutralization assay. The secondary immunogenicity outcome was geometric mean concentrations (GMCs) of IgG antibodies and the corresponding 4-fold rise rate against SARS-CoV-2 prototype strain. IgG antibodies were measured using a magnetic particle-based chemiluminescence enzyme immunoassay kit. The safety outcome was occurrence and severity of any adverse reactions within 30 days post-boost. As an exploratory study, the immunogenicity of booster vaccination against SARS-CoV-2 variants of concerns (VOCs), including Omicron, was also evaluated by the GMTs of neutralizing antibodies in a subset of participants from 7-9-month group. Neutralizing antibody titers against the VOCs, including Alpha, Beta, Delta and Omicron, were measured using live-virus neutralization assay.
